# Relationship between sex and cardiovascular mortality in chronic kidney disease: A systematic review and meta-analysis

**DOI:** 10.1371/journal.pone.0254554

**Published:** 2021-07-12

**Authors:** Sultana Shajahan, Janaki Amin, Jacqueline K. Phillips, Cara M. Hildreth

**Affiliations:** 1 Department of Biomedical Science, Faculty of Medicine, Health and Human Sciences, Macquarie University, Sydney, Australia; 2 Department of Health Systems and Populations, Faculty of Medicine, Health and Human Sciences, Macquarie University, Sydney, Australia; Universita degli Studi di Perugia, ITALY

## Abstract

Chronic kidney disease (CKD) is a significant health challenge associated with high cardiovascular mortality risk. Historically, cardiovascular mortality risk has been found to higher in men than women in the general population. However, recent research has highlighted that this risk may be similar or even higher in women than men in the CKD population. To address the inconclusive and inconsistent evidence regarding this relationship between sex and cardiovascular mortality within CKD patients, a systematic review and meta-analysis of articles published between January 2004 and October 2020 using PubMed/Medline, EMBASE, Scopus and Cochrane databases was performed. Forty-eight studies were included that reported cardiovascular mortality among adult men relative to women with 95% confidence intervals (CI) or provided sufficient data to calculate risk estimates (RE). Random effects meta-analysis of reported and calculated estimates revealed that male sex was associated with elevated cardiovascular mortality in CKD patients (RE 1.13, CI 1.03–1.25). Subsequent subgroup analyses indicated higher risk in men in studies based in the USA and in men receiving haemodialysis or with non-dialysis-dependent CKD. Though men showed overall higher cardiovascular mortality risk than women, the increased risk was marginal, and appropriate risk awareness is necessary for both sexes with CKD. Further research is needed to understand the impact of treatment modality and geographical distribution on sex differences in cardiovascular mortality in CKD.

## Introduction

Chronic kidney disease (CKD) is a significant global health issue, resulting in premature death, reduced quality of life and substantial financial burden for patients [1]. In 2016, CKD was the 16th leading cause of death worldwide [2] and has been projected to be the 5th leading cause of death by 2040 [3]. The primary cause of mortality in CKD patients is cardiovascular disease, namely heart failure, myocardial infarction, sudden cardiac arrest and stroke [4].

Cardiovascular mortality is associated with several risk factors, including biological sex, age, smoking and obesity [5]. The effect of biological sex on cardiovascular mortality in CKD patients is unclear. Historically, the consensus has been that the life-long risk for cardiovascular mortality is higher in men than women within the general population [6]. However, few studies have focussed on sex differences in cardiovascular outcomes in the CKD population. A meta-analysis of renal function and the association of sex with cardiovascular mortality found that men had a higher mortality risk across all levels of renal function compared with women [7]. These findings are consistent with analyses of the European Renal Association-European Dialysis and Transplant Association Registry [8]. However, other population studies show that the hazard ratio (HR) for cardiovascular-specific (e.g. myocardial infarction; MI) and all-cause cardiovascular mortality is higher in women than men [9]. Moreover, more recent data from 2018 suggests that women with CKD have a higher annual risk of cardiovascular hospitalisations and death than men [10]. Thus the collective evidence regarding the role of sex on cardiovascular mortality among CKD patients is inconsistent and inconclusive.

To address this, we conducted a systematic review and meta-analysis of the literature published between 2004 and 2020. We sought to determine the effect of sex on cardiovascular mortality among CKD patients and identify if a need exists for risk stratification and sex-specific guidelines regarding cardiovascular mortality risk among CKD patients.

## Materials and methods

### Search strategies

This review was conducted per the Preferred Reporting Items for Systematic Reviews and Meta-Analyses (PRISMA) guidelines ([11]; see S1 Checklist). A systematic search of the literature published between January 2004 and October 2020 was conducted using PubMed/Medline, EMBASE, Scopus and the Cochrane library. A date limit was introduced based on the guidelines for Kidney Disease Outcomes Quality Initiative [12], which aimed to set a standard for the definition of CKD in the early 2000s, utilising eGFR as a measure of kidney disease progression. Combinations of appropriate Medical Subject Headings and keywords were used to comprehensively search the selected databases, with syntax amended for each database: (chronic renal insufficiency OR chronic kidney failure OR (chronic kidney adj (disease or insufficiency)) OR (chronic renal adj (disease or insufficiency)) OR end-stage renal disease OR uremia OR uraemia) AND (cardiovascular disease mortality OR (cardiovascular adj (mortality or death or event* or complication* or outcome*)) OR heart disease mortality) AND (sex factors OR male or female OR sex distribution OR sex characteristics OR sex ratio OR men OR women OR men or women or male* or female* or sex or gender or “sex difference*”). The search was limited to English. Additional details are provided in S1 Data Reference lists from relevant reviews were also screened for pertinent studies.

### Study selection and eligibility criteria

Studies were included if they met the following criteria:

- non-interventional cohort study (prospective/retrospective) of adult patients (aged > 18 years) with any stage of CKD, including dialysis patients, and CKD defined as an eGFR < 60 mL/min per 1·73 m^2^ (Stages 3–5 CKD).
- reported rate/risk estimates of cardiovascular mortality of men relative to women as HRs or RRs with 95% confidence intervals or contained enough data to calculate risk estimates.
- contained patient population data collected in or after 2004.
- had a follow-up of at least one year.

If a group of studies drew participants from the same cohort, the study with more comprehensive data and longer follow-up duration was selected.

To reduce any confounding variables that may affect the association between CKD and cardiovascular mortality, we excluded studies if:

- the patient population exclusively contained type 1 and 2 diabetes mellitus patients.
- study participants had any infection, carcinoma, acute kidney injury, surgical interventions (e.g., kidney transplant or coronary artery bypass surgery).
- study participants received any non-conventional drug treatments (e.g., chemotherapy).

Reviews, comments, letters to editors or unpublished studies were also excluded.

After removing all duplicates, records identified through database searches and other sources were screened by SS for eligibility by screening titles and abstracts. The full texts of the screened records were then independently reviewed against eligibility criteria by three reviewers, SS (100%), CMH (2%) and JKP (2%), where the percentage denotes the proportion of the studies screened by each reviewer. Disagreement regarding any study was resolved by discussion between the three reviewers.

### Quality assessment

The Newcastle-Ottawa Scale (NOS) was used to assess the risk of bias for cohort studies (S2 Data) [13]. Since the primary objectives of the included studies differed from the research question of this systematic review, we adapted the NOS to assess the quality of data relevant to the research question. We therefore used three items to assess study quality: (i) selection of participants (including three domains); (ii) comparability of study results (including three domains); and (iii) outcome (including four domains). Each domain had a rating of “yes,” “no”, or “unclear.” If there was adequate data against a domain in the included study and met the criteria, it was classified as low risk of bias (yes). Conversely, a domain was classified as high risk of bias if adequate information was not available (no) or not enough data was available to make an assessment (unclear). “Yes” was scored as “1”, and “no” or “unclear” was scored as “0.” Scores were tallied up to calculate the final cumulative score. A study was considered high quality if the cumulative score was ≥ 4, and low quality if <4. Three reviewers assessed quality (SS (100%), JA (6.6%) and CMH (4.44%) and any disagreement was resolved through discussion.

### Data extraction and data items

Data were extracted using an adapted form of the Data Extraction Template for systematic reviews (Cochrane Public Health Group; S3 Data). SS performed all data extraction with a proportion of duplicate data extraction performed by JA (6.6%) and CMH (4.44%), and any disagreements between reviewers resolved by discussion.

Data were collected for the number of men and women participants with CKD, CKD stage, mode of treatment, comorbidities, the average age of patients, length of follow-up and number of patients lost to follow-up. Outcome measures included rate/risk estimates of cardiovascular mortality of men relative to women as HRs and RRs with 95% CI and p-values, where cardiovascular death was defined as death due to any cardiovascular disease, such as myocardial infarction, heart failure, stroke, sudden cardiac arrest, and atrial fibrillation. Where rate estimates were not available, the absolute number of cardiovascular deaths stratified by sex were collected to calculate relative rates with 95% CI and p-values. Information was also collected for any adjustments for confounders, such as age, diabetes, hypertension, and obesity.

Additional data, where available, were also collected for: (i) sex-stratified cardiovascular mortality risk relative to a non-CKD population and (ii) cause-specific cardiovascular mortality, namely heart failure, myocardial infarction, and stroke. An effort was made to contact study authors in the case of missing data.

### Statistical analysis

Extracted and calculated effect estimates hazard ratios (HR) and rate ratios (RR) were combined in the meta-analysis to calculate the overall risk estimate. A random-effects model was used for analysis to account for the variation of real effects across studies [14]. The overall estimate was denoted to be a risk estimate (RE). Heterogeneity among risk estimates was measured using the I^2^ statistic. The degree of heterogeneity was assessed from the I^2^ statistic using the following thresholds for interpretation: (1) 0% to 30%: marginal heterogeneity; (2) 30% to 50%: moderate heterogeneity; (3) 50% to 75%: substantial heterogeneity; and (4) 75% to 100%: represents considerable heterogeneity [14].

Exploratory subgroup meta-analyses were conducted on several variables that may have contributed to heterogeneity: quality of studies (high vs. low); geographical distribution (China vs. Taiwan vs. Korea vs. Japan vs. Europe vs. United States vs. Australia and New Zealand vs. Africa); sample size (<100 vs. 100–999 vs. >1000); length of follow-up in years (<2 vs. 2–5 vs. >5); male to female ratio in the study sample (0.4–0.8 vs. 0.9–1.1 vs. 1.2–2.8); and dialysis modality (haemodialysis (HD), peritoneal dialysis (PD), no dialysis). An additional subgroup analysis was conducted based on adjustment for confounders (adjusted vs. unadjusted) for reported HR from included studies.

A 95% CI with no overlap with the null effect value (1) were interpreted to demonstrate a statistically significant difference between sexes in risk of cardiovascular mortality. Publication bias was assessed via a funnel plot with pseudo-95% CI and the log of the RR as θ. All analyses were performed using the ‘meta’ package in STATA version 16 and Revman 5.

## Results

### Search result

**Fig 1:**
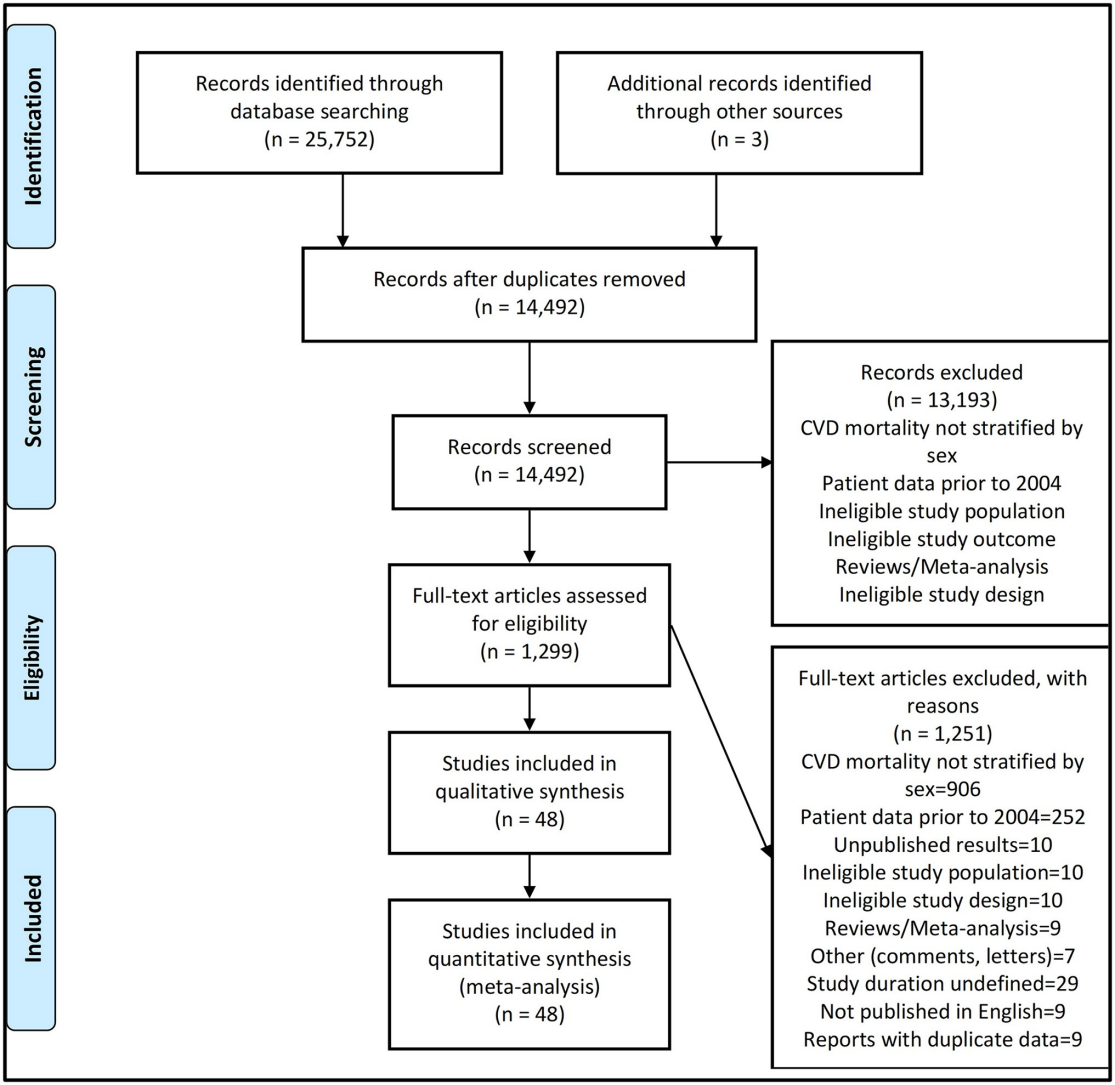
Flow diagram of study selection and search results. n, number of studies. CVD, Cardiovascular disease.

A systematic search of the literature identified a total of 14,492 studies (25,752 studies before removal of duplicate studies) with title and abstract screening identifying a total of 1,299 eligible studies (see Fig 1). A full-text review of eligible studies was evaluated against the eligibility criteria, yielding 57 studies. S1 Table details the excluded studies and reasons for exclusion. Of the 57 studies, nine identified studies contained data from the same study cohort. After careful evaluation, the studies with the most comprehensive data and longer follow-up time were included. The final review included a total of 48 studies.

### Characteristics of included studies

Characteristics of the included studies are detailed in Table 1. The total number of subjects across all the studies were 99,822, which comprised 51,069 men and 48,753 women. There was a higher proportion of men than women across all the studies (average ratio: 1.3; range: 0.4 [15] to 2.8 [16]), with the age of subjects typically ranging from 52–73 years, except for one study [17] which had a comparatively younger study population (average age: 36 years). All studies were published between 2010 and 2020, had an average duration of follow-up of 3.75 years (range: 1.28 [18] to 11 [19] years) and variable sample size (62 [20] to 38,377 [21] subjects).

**Table 1:** Summary of study characteristics.

Author/country	Stage of CKD	Dialysis Modality	Sample size (ratio men: women)	Mean age	Follow-up yr.	Baseline comorbidities of study sample (%)	Study Factor	Outcome Factor	NOS score
**Wang 2020** [45]**/China**	ESRD	HD	205 (1.6)	59.0	3.0	HTN (90); DM (29.3)	Biomarkers soluble suppression of tumorigenicity 2 (sST2) and N-terminal pro-brain natriuretic peptide (NT-proBNP)	all-cause and cardiovascular mortality	3
**Zhang 2020** [44]**/China**	ESRD	HD	252 (1.3)	57.1	6.1	HTN (31.3), Coronary Heart Disease (6.7), DM (12.3), Cerebral infarction (14.3), Cerebral haemorrhage (3.2)	Plasma Trimethylamine-N-Oxide (TMAO)	cardiovascular and all-cause mortality	2
**Yu 2020** [19]**/China**	ESRD	HD	358 (1.1)	74.0	11	DM (50.3), HTN (95.8), CHF (13.7), Stroke (12), Arrhythmia (8.1), CHD (3.9), Peripheral vascular disease (0.8), Cancer (4.2)	Impact of vascular access	cardiovascular and all-cause mortality	6
**Lee 2020** [59]**/Taiwan**	3–5	No	472 (1.1)	66.8	2.0	DM (36.70); HTN (75.80); Coronary artery disease (19.6); Cerebrovascular disease (8.1); CHF (12)	Upstroke time	Cardiovascular and all-cause mortality	4
**Tsai 2020** [55]**/Taiwan**	ESRD	PD	133 (0.8)	56.0	6.4	DM (21.05), HTN (84.96)	Heart rhythm complexity	cardiovascular mortality	3
**Simsek 2020** [60]**/Turkey**	3,4	No	191 (0.7)	66.3	6.3	DM (19.40), HTN (57.10), Previous coronary heart disease (7.30), Dyslipidaemia (66.00)	N-terminal pro-brain natriuretic peptide (NT-proBNP) level	Mortality	5
**Toyama 2019** [16]**/Japan**	ESRD	HD	286 (2.8)	70.5	2.0	DM (34.62); HTN (77.98); Dyslipidaemia (49.30); Prior myocardial infarction (11.89)	stress myocardial perfusion single-photon emission computed tomography (SPECT)	cardiovascular/cerebrovascular events	3
**Mizuiri 2019** [47]**/Japan**	ESRD	HD	353 (2.0)	68.0	3.0	DM (40.2)	hypomagnesemia	All-cause and cardiovascular mortality	3
**Chen 2019** [58]**/Taiwan**	3–5	No	568 (1.5)	66.0	2.7	DM (57.57); HTN (85.56); CAD (79); Cerebrovascular disease (59)	aortic arch calcification (AoAC) and cardio-thoracic ratio (CTR)	all-cause and cardiovascular mortality	5
**Yadav 2019** [46]**/The Netherlands**	ESRD	HD	199 (1.5)	70.0	4.0	DM (39); HTN (79); CVD (55)	digital brachial index	Survival rates	3
**Cano-Megias 2019** [57]**/Spain**	4,5	HD	137 (1.2)	61.5	10.0	DM (27.7); HTN (89); CAD (20.1); Cerebrovascular disease (11.3); Previous cardiologic event (26.3)	coronary artery calcification (CaC)	Overall and cardiovascular mortality	3
Wu 2019 [28]/China	ESRD*	HD	169 (1.20)	60.2	7.0	NA	baseline serum magnesium level	mortality	4
Saglimbene 2019 [29]/Europe and South America	ESRD	HD	8110 (1.40)	63.1	2.7	HTN (85), DM (32), HF (19.1), MI (11.60), stroke (8.8), pulmonary disease (11.6), gastrointestinal disease (21.7)	n-3 polyunsaturated fatty acid dietary intake	mortality	6
Gong 2018 [48]/China	ESRD*	PD	98 (0.96)	52.5	6.0	DM (21.4), History of CVD (7.1)	elevated serum sclerostin levels	mortality	3
Yayar 2018 [30]/Turkey	ESRD*	HD	82 (0.78)	57.9	4.0	DM (26.8), History of CVD (35.4)	serum hepcidin-25 & sub-clinic atherosclerosis	mortality	5
Kawagoe 2018 [31]/Japan	ESRD*	HD	1310 (1.40)	67.9	2.0	Not Reported	N-terminal-pro-B-type-natriuretic peptide	mortality	5
Kon 2018 [62]/Japan	3–5	Not clear	27,362 (1.20)	70.2	5.0	Stroke (19.6), Heart disease (29.2)	baseline eGFR	5-year all-cause & cardiovascular mortality	3
Navaneethan 2018 [21]/US	3–5	NA	38,377 (0.80)	71.7	4.5	HTN (87.2), DM (29.8), CAD (24.2), HF (7.8), Cerebrovascular disease (10.4), PVC (3.7)	high-density lipoprotein cholesterol	mortality	4
Zhang 2017 [25]/China	ESRD*	HD	414 (1.60)	61.8	1.9	DM (22.9), HTN (94), CVD (9.2)	soluble suppression of tumorigenicity 2	mortality	4
Wu 2017 [49]/Taiwan	ESRD*	PD	190 (0.80)	52.6	4.6	DM (15.3), CVD (21.1)	chest X-ray-detected aortic arch calcification	mortality	6
Peng 2017 [50]/China	ESRD*	PD	345 (1.40)	52.8	2.1	DM (23.5), HTN (43.2), CVD (20)	prognostic nutritional index	cardiovascular disease mortality	3
Jeng 2017 [32]/Taiwan	ESRD*	HD	136 (1.20)	60.3	5.6	DM (50), HTN (72.1), CVD (53.4)	proinflammatory monocytes levels	all-cause & cardiovascular mortality	4
Antunovic 2017 [20]/ Montenegro	ESRD	HD	62 (0.90)	57.8	2.0	DM (8.1), HTN (32.3)	high-sensitive troponin I	mortality	3
Isla 2016 [17]/South Africa	ESRD	HD & PD	340 (1.10)	36.1	3.1	DM (10.3), HTN (25.9) HIV positive (3.1)	causes and predictors	mortality	4
Lu 2016 [33]/Taiwan	ESRD*	HD	154 (0.90)	69.1	4.2	DM (55.9), HTN (68.8)	number of endothelial progenitor cells	cardiovascular and all-cause mortality	4
Merle 2016 [22]/France	ESRD*	HD	1983 (1.60)	67.9	2.0	DM (37.7), HTN (79.1), CVD (54.6)	low parathyroid hormone (PTH) status	mortality	4
Chen 2015 [37]/China	ESRD*	HD	110 (1.40)	55.2	3.5	DM (57.3)	aortic artery calcification, cardiac valve calcification	mortality	3
Flythe 2015 [36]/US	ESRD*	HD	10,758 (1.20)	61.0	3.0	DM (59.1), Heart Failure (43.1), CAD (13.4)	post-dialysis weights above and below the prescribed target weight	mortality	5
Tsai 2015 [35]/Taiwan	ESRD*	HD	444 (0.87)	61.6	4.3	DM (32.7), CVD (20.9)	site of peripheral artery occlusive disease	all-cause & cardiovascular mortality	6
Oh 2015 [51]/Korea	ESRD*	PD	335 (1.60)	53.5	1.8	DM (41.8), HTN (48.1), Coronary arterial disease (11.3), Peripheral arterial disease (7.5)	3 biomarkers (N-terminal-pro-B-type-natriuretic peptide, Cardiac troponin T and high-sensitivity C-reactive protein)	mortality	4
Ulusoy 2015 [34]/Turkey	ESRD	HD	238 (1.40)	60.3	2.0	DM (36.3), HTN (30.9), Peripheral vascular disease (6.3)	tenascin-C levels	cardiac mortality	4
Yoshitomi 2014 [61]/Japan	3–5	NA	320 (2.10)	70.0	2.5	HTN (94), DM (51), History of CVD (19), Dyslipidaemia (73), History of IHD (19)	ankle-brachial blood pressure index	cardiovascular events and mortality	1
Okamoto 2014 [56]/Japan	ESRD*	HD, PD	126 (1.30)	67.0	5.0	DM (52.4)	visceral fat area	mortality	2
Li 2014 [38]/China	ESRD*	HD	278 (1.20)		1.8	DM (33.8), HTN (91.1), History of CVD (30.6)	pulmonary hypertension	cardiovascular mortality and events	5
Honneger Bloch 2014 [39]/New Zealand	ESRD*	HD	238 (1.10)	63.0	2.0	DM (64), History of MI (67)	high sensitivity troponin T	mortality	4
Oh 2014 [24]/Korea	ESRD	HD	864 (1.50)	59.7	1.5	DM (56.3), HTN (48)	3 biomarkers (N-terminal-pro-B-type-natriuretic peptide, Cardiac troponin T and high-sensitivity C-reactive protein)	mortality	3
Avramovski 2014 [26]/Macedonia	ESRD*	HD	80 (2.00)	59.3	2.6	DM (20), HTN (46.2)	aortic pulse wave velocity	all-cause and cardiovascular mortality	3
Arsov 2014 [42]/ Macedonia, Germany, Sweden	ESRD*	HD	169 (1.60)	56.0	3.0	DM (24), HTN (18), CVD (18)	skin autofluorescence and release of heart-type fatty acid binding protein in plasma	overall & CVD mortality	3
Lim 2013 [40]/Taiwan	ESRD*	HD	248 (1.00)	65.0	4.9	DM (51.2), HTN (75.4)	serum oxidized albumin	all-cause & cardiovascular mortality	4
Li 2013 [52]/China	ESRD*	PD	66 (0.89)	62.1	3.5	Non-diabetic, History of CVD (9.1)	insulin resistance	cardiovascular morbidity and mortality	4
Genovesi 2013 [27]/Italy	ESRD*	HD	122 (1.80)	69.8	3.9	Ischaemic heart disease (37.7), DM (27.1), HTN (84.4), Dilated cardiomyopathy (41.8), Valvular heart disease (23.8), Dyslipidaemia (18), Ischaemic cerebral disease (14.8), Atrial fibrillation (41.8)	various risk factors	total mortality & sudden cardiac death	3
den Hoedt 2013 [41]/ Netherlands, Canada, Norway	ESRD	HD	714 (1.70)	64.1	3.0	DM (24), History of CVD (44)	online hemodiafiltration versus low-flux haemodialysis	all-cause & CV morbidity and mortality	4
Murthy 2012 [18]/US	CKD Stages 3–5	Not clear	866 (1.00)	71.1	1.3	DM (44.8), HTN (91.2), Dyslipidaemia (70), Recent MI < = 30 days (18.9), Remote MI >30 days (22.6), Cerebrovascular disease (8), Peripheral vascular disease (9.1)	vasodilator function	mortality	3
An 2012 [54]/China	ESRD*	PD	138 (1.40)	53.0	3.2	DM (23.9), CAD (29.1), Cerebrovascular disease (7.7), PVD (2.6)	neutrophil to lymphocyte ratio	cardiovascular & all-cause mortality	6
Wu 2012 [15]/Taiwan	ESRD*	HD	112 (0.44)	72.6	2.8	DM (62.5), HTN (56.3)	Serum free p-cresyl sulphate levels	all-cause and CV mortality	2
Lee 2012 [53]/Taiwan	ESRD*	PD	415 (1.30)	55.8	2.9	DM (47.2), CVD (34.9)	prevalence of aortic arch calcification	mortality	6
Kakiya 2012 [43]/Japan	ESRD*	HD	494 (1.70)	60.9	4.2	Diabetic Nephropathy (22.3), HTN (86.2), Pre-existing CVD (33.6)	serum adrenal androgen dehydroepiandrosterone sulphate levels	mortality	4
Ogawa 2010 [23]/Japan	ESRD*	HD	401 (2.10)	61.5	4	DM (33.2)	aortic arch calcification score	all-cause & cardiovascular mortality	3

HD = Haemodialysis, PD = Peritoneal dialysis, ESRD = End-Stage Renal Disease, DM = Diabetes mellitus, HTN = Hypertension, CVD = Cardiovascular disease, PVD = Peripheral vascular disease, HF = Heart failure, MI = Myocardial infarction, CAD = Coronary artery disease. NOS = Newcastle-Ottawa Scale.

*Patients received dialysis therapy and thus assumed to have ESRD.

The majority of the studies reported results for end-stage renal disease (ESRD) patients on dialysis: 30 studies included subjects exclusively on HD [15, 16, 19, 20, 22–47], eight studies included subjects on PD [48–55], three studies included both HD and PD [17, 18, 56], and six studies reported their subjects were not on dialysis [21, 57–61]. Six studies defined their subjects as having CKD stages 3 to 5, [18, 21, 58, 59, 61, 62], while only one study described their subjects as CKD stages 3 and 4 [60]. In addition to this, most studies (33/48) reported comorbidity with diabetes mellitus and hypertension. Of the remaining studies, 15 studies did not report any comorbidity [23, 28, 30, 31, 35–37, 39, 41, 48, 49, 53, 54, 56, 62] and only one excluded diabetes as part of their patient selection criteria [52].

The majority of included studies were conducted in Asia (Japan, China, Taiwan; 30 in total). Among the remaining 18 studies: ten were undertaken in Europe (France, Italy, Macedonia, Sweden, Germany, Montenegro, The Netherlands, Spain and Turkey) [20, 22, 26, 27, 30, 34, 42, 46, 57, 60], three in the United States [18, 21, 36], one in Australia and New Zealand [39], one in South Africa [17], and two included more than one geographical region [29, 41].

Forty-four of the included studies were hospital/clinic-based, two were registry-based studies [21, 62], and one study was based on a previous clinical trial [60]. Thirty of the 48 studies had accounted for all study participants during follow-up [16, 19, 23, 25, 28–33, 35, 38–43, 45–47, 49–55, 58–60]; however, the remaining 18 studies did not clearly describe follow up method or status for all patients.

### Risk of bias

**Fig 2:**
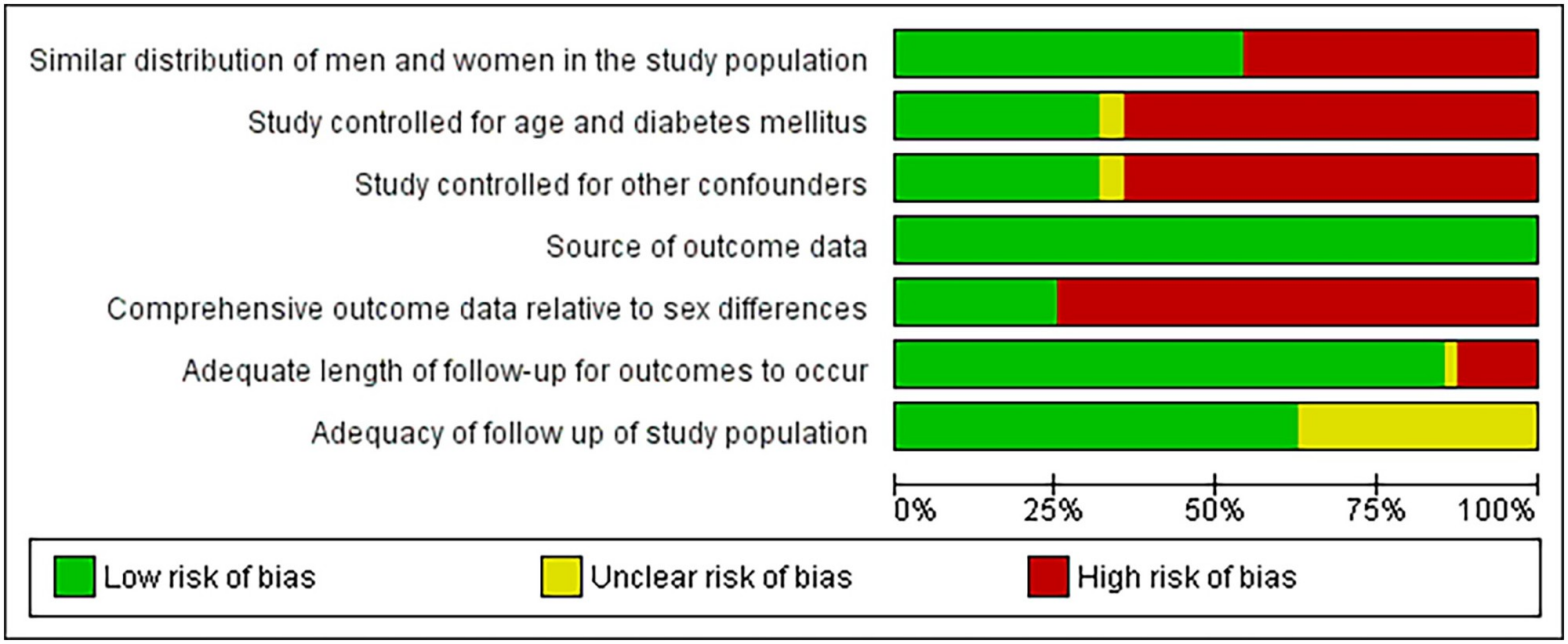
Risk of bias graph. Review authors’ judgements about each risk of bias item as presented as percentages across all included studies.

**Fig 3:**
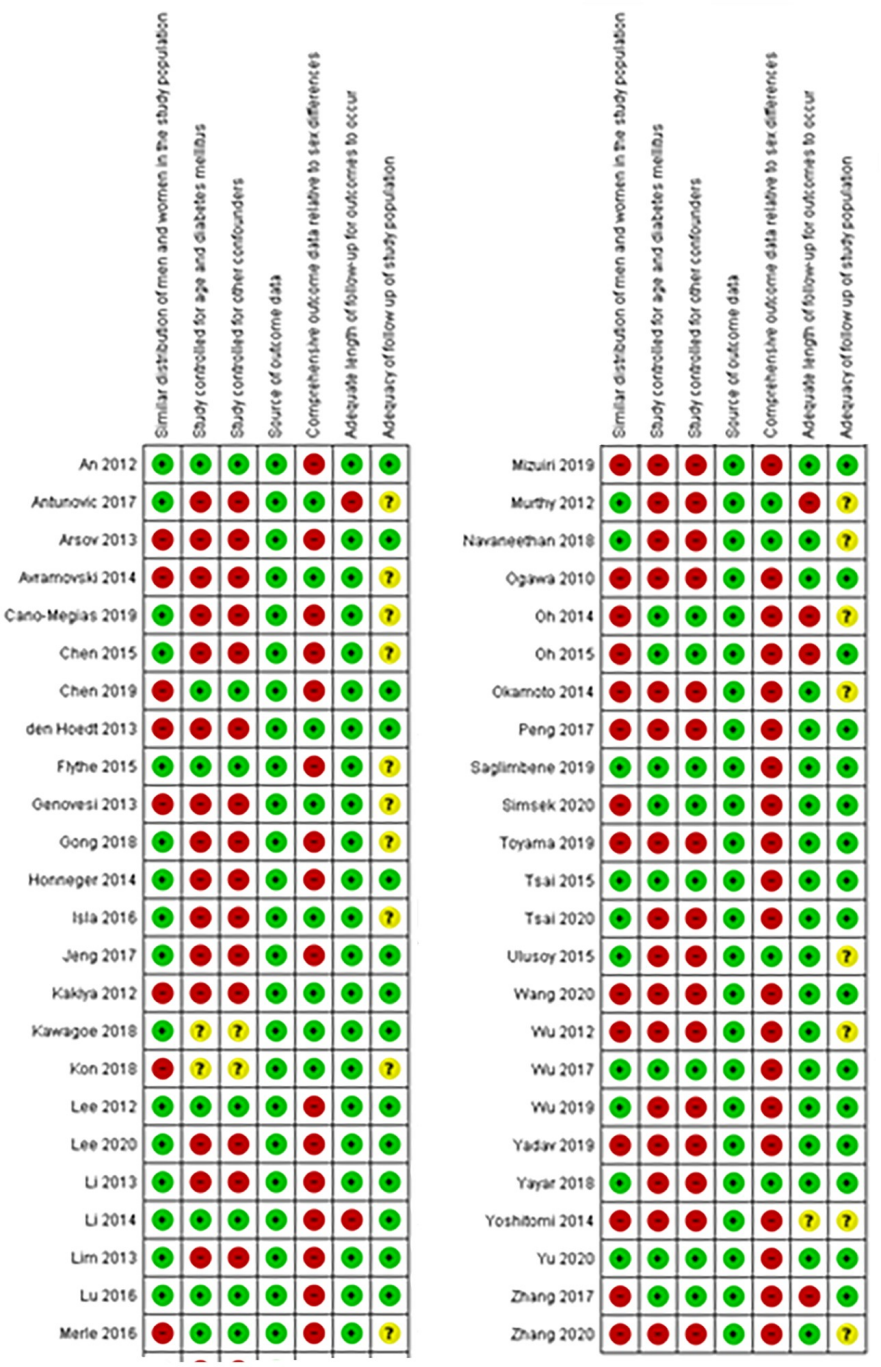
Risk of bias summary. Review authors’ judgements about each risk of bias item for each included study.

Presented in Fig 2 is a summary of the quality assessment of included studies, with review authors’ judgements about each risk of bias item for each included study in Fig 3. Additional information regarding the risk of bias for each study is provided in S2 Table.

In total, 28 studies had a cumulative quality score ≥ four [17, 19, 21, 22, 25, 28–36, 38–41, 43, 49, 51–54, 58–60], and were thus considered high quality. The most frequent limitations observed were a lack of comprehensive sex-stratified data, unequal distribution of men and women in the study population, lack of adjustment for age and diabetes, lack of adjustment for other confounders and covariates, e.g., history of CVD, hypertension and haemoglobin level, and a lack of information regarding patient follow-up (see Figs 2 and 3). The outcome data source was consistent across studies, mainly through patient medical records or data linkage to mortality registries.

### Sex and overall cardiovascular mortality

The HR of men for cardiovascular mortality relative to women with 95% CI were reported in 38 out of the 48 studies, with 23 unadjusted and 15 adjusted HRs (Table 2). The remaining ten studies reported the number/proportion of cardiovascular deaths stratified by sex (Table 3), which were used to calculate the rate ratio (RR). 23 out of the 48 included studies reported a higher cardiovascular mortality rate in men than women, with risk estimates ranging from 1.16 to 3.51 (Tables 2 and 3) [18–20, 25, 30, 35, 37, 38, 42, 45, 55, 57, 58, 60–62]. However, 18 of the 48 studies showed that men had a lower risk of cardiovascular mortality compared to women, with risk estimates ranging from 0.29 to 0.93 (Tables 2 and 3) [23, 24, 34, 39, 44, 46, 47, 49, 53, 54, 59].

**Table 2:** Summary of reported risk estimates of male sex for cardiovascular mortality.

Study	Unadjusted				Adjusted				
	HR	95% CI Lower Limit	95% CI Upper Limit	P-value	HR	95% CI Lower Limit	95% CI Upper limit	P-value	Adjusted Variables
**Wang 2020**	1.36	0.46	4.05	0.58	-	-	-	-	n/a
**Zhang 2020**	0.93	0.49	1.75	0.81	-	-	-	-	n/a
**Yu 2020**	-	-	-	-	1.49	0.75	2.95	0.25	Age, dialysis vintage in months, diastolic BP, CHF, CHD, Arrhythmia, Arrhythmia with a pacemaker, Previous stroke, DM, Primary gout, Systemic vasculitis or lupus nephritis, Cancer, Vascular access types, others
**Lee 2020**	0.86	0.52	1.42	0.56	-	-	-	-	n/a
**Tsai 2020**	1.21	0.50	2.29	0.67	-	-	-	-	n/a
**Simsek 2020**	-	-	-	-	2.79	1.38	5.62	0.00	Previous CHD, Anaemia, HTN, Urinary albumin to creatinine ratio >30 mg/g, Log N-terminal pro-brain natriuretic peptide, eGFR
**Toyama 2019**	1.04	0.52	12.51	0.36	-	-	-	-	n/a
**Mizuiri 2019**	0.83	0.46	1.54	0.54	-	-	-	-	n/a
**Chen 2019**	1.81	0.96	3.42	0.07	1.23	0.61	2.49	0.57	Age, Chest Thoracic Ratio, Aortic Calcification score
**Yadav 2019**	0.88	0.50	1.59	0.68	-	-	-	-	n/a
**Cano-Megias 2019**	1.19	0.57	2.50	0.65	-	-	-	-	n/a
Wu [28]	1.03	0.46	2.30	0.95	-	-	-	-	n/a
Saglimbene [29]	-	-	-	-	1.16	0.98	1.37	0.08	Age, DM, MI, education, smoker, vascular access type, body mass index, albumin, Charlson comorbidity score, phosphorus level, calcium level, haemoglobin, KT/V (index to quantify haemodialysis treatment adequacy), fibre daily intake, energy intake
Gong [48]	0.69	0.29	1.64	0.40	-	-	-	-	n/a
Zhang [25]	-	-	-	-	2.77	1.11	6.94	0.03	Age, DM, coronary heart disease, dialysis vintage, vascular access, sST2, NT-proBNP, hs-cTnT, hs-CRP, haemoglobin, serum albumin, leukocyte count, serum urea, serum creatinine, uric acid, body mass index, systolic BP, diastolic BP
Wu [49]	-	-	-	-	0.40	0.05	3.02	0.38	Age, DM, duration of PD, CVD, MBP, BMI, albumin, phosphorous, HDL, aortic arch calcification
Peng [50]	0.70	0.34	1.42	0.32	-	-	-	-	n/a
Jeng [32]	0.96	0.51	1.79	0.89	-	-	-	-	n/a
Isla [17]	0.83	0.39	1.78	-	-	-	-	-	n/a
Lu [33]	-	-	-	-	1.28	0.61	2.66	-	Age, DM, HTN, endothelial progenitor cells, current smoker, dialysis efficiency, haemoglobin, Harrell’s concordance
Merle [22]	-	-	-	-	1.05	0.67	1.64	0.84	Age, DM, hypertension, smoking, prevalent cardiovascular events (cerebrovascular disease, ischemic heart disease, heart failure, and peripheral artery disease)
Chen [37]	3.51	1	12.31	0.05	-	-	-	-	n/a
Flythe [36]	-	-	-	-	1.19	1.04	1.35	-	Age, DM, race, CAD, HF, vascular access type, albumin, phosphorus level, haemoglobin, equilibrated Kt/V, dialytic vintage, prescribed treatment time (minutes), intradialytic weight gain, post-dialysis weight, pre-dialysis systolic BP, missed treatments
Tsai [35]	1.13	0.75	1.70	0.54	1.87	1.11	3.16	0.02	Age, DM, CVD, BP, albumin, triglyceride cholesterol, Kt/v, cardiomegaly, Ca-P product, peripheral arterial occlusion disease
Oh [51]	0.68	0.32	1.45	0.32	0.69	0.32	1.49	0.34	Age, white blood cell count
Yoshitomi [61]	2.82	0.95	12.09	0.06	-	-	-	-	n/a
Okamoto [56]	1.19	0.42	3.34	0.33	-	-	-	-	n/a
Li [38]	2.25	0.99	5.10	0.05	2.06	0.89	4.75	0.09	Age, DM, CVD, pulmonary hypertension, duration of HD, pre-HD BP, serum phosphorus, urea reduction ratio, systolic dysfunction
Honneger Bloch [39]	0.66	0.35	1.24	0.20	-	-	-	-	n/a
Oh [24]	1.09	0.50	2.34	0.83	0.62	0.18	2.10	0.44	Age, DM, HTN
Arsov [42]	2.44	1.05	5.64	0.04	-	-	-	-	n/a
Lim [40]	0.95	0.58	1.54	0.83	-	-	-	-	n/a
Li [52]	1.31	0.68	2.54	0.42	-	-	-	-	n/a
Murthy [18]	1.71	1.11	2.63	0.01	-	-	-	-	n/a
An [54]	-	-	-	-	0.29	0.09	0.90	0.03	Age, diabetic nephropathy, history of CVD, albumin level, neutrophil to lymphocyte ratio
Wu [15]	0.71	0.27	1.83	0.47	-	-	-	-	n/a
Lee [53]	-	-	-	-	0.55	0.25	1.21	>0.05	Age, DM, CVD, smoking, lipid-lowering therapy calcium phosphorous product, albumin, log hs-CRP, baseline aortic arch calcification
Ogawa [23]	0.59	0.25	1.37	0.22	-	-	-	-	n/a

**Table 3:** Summary of additional data from studies not reporting risk estimates.

Study	Men			Women			RR of men for cardiovascular mortality	95% (CI) lower limit	95% CI upper limit	*P*-value
	CVD deaths (*n*)	Sample size (*n*)	Rate of CVD death in/1000 person-years	CVD deaths (*n*)	Sample size (*n*)	Rate of CVD death/1000 person-years				
Yayar [30]	13	36	361.11	8	46	173.91	2.08	0.96	4.46	0.06
Kawagoe [31]	29	770	37.66	25	540	46.29	0.81	0.48	1.37	0.44
Kon [62]	31	14,810	2.09	12	12,509	0.95	2.18	1.11	4.24	0.02
Navaneethan [21]	1851	15,112	122.49	1817	19,597	92.71	1.30	1.20	1.36	<0.01
Antunovic [20]	6	30	200.00	4	32	125.00	1.60	0.50	5.11	0.43
Ulusoy [34]	20	140	142.86	20	98	204.08	0.66	0.37	1.15	0.15
Avramovski [26]	10	53	188.68	7	27	259.25	0.73	0.31	1.69	0.46
Genovesi [27]	13	79	164.56	7	43	162.79	1.01	0.43	2.34	0.98
den Hoedt [41]	50	445	112.36	24	269	89.21	1.26	0.79	2.00	0.33
Kakiya [43]	22	313	70.29	13	181	71.82	0.98	0.50	1.89	0.95

**Fig 4:**
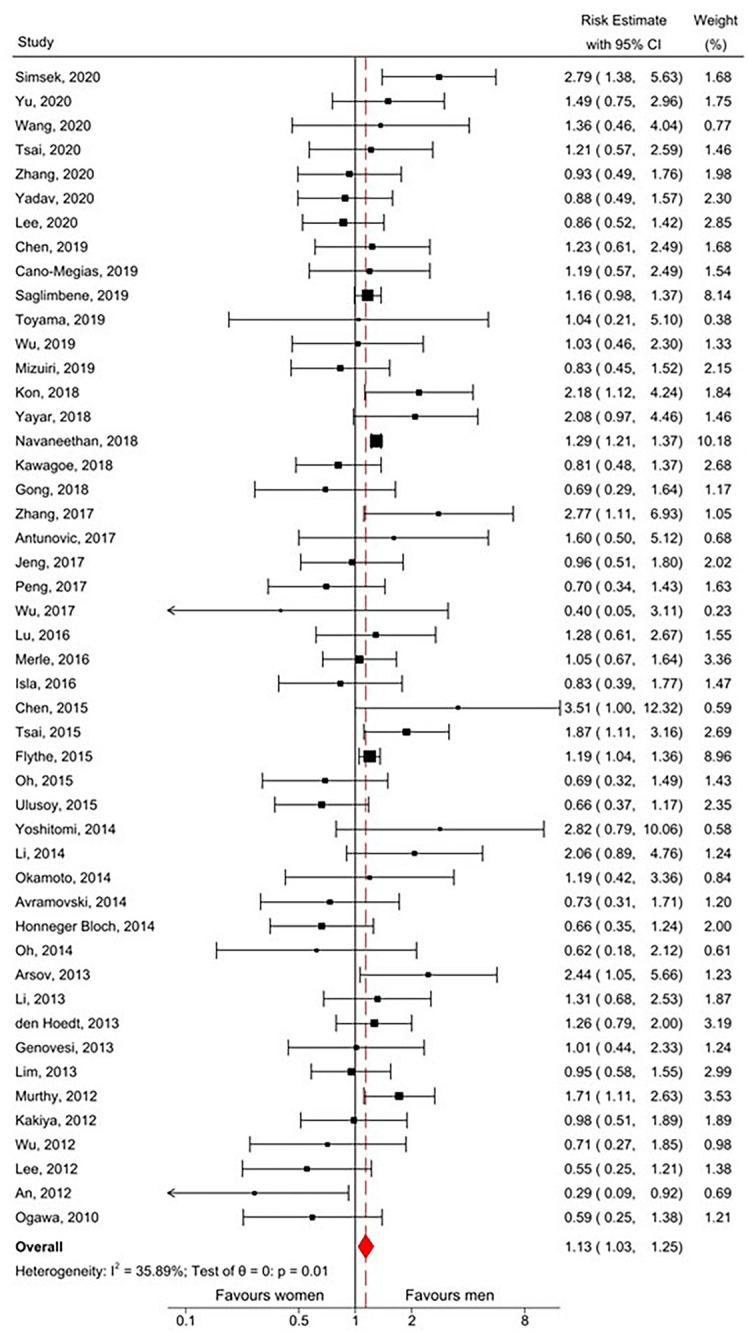
Forest plot of meta-analysis of risk estimates of all included studies. CI, confidence interval. I^2^, Measure of heterogeneity. θ, Risk estimate. p, p-value.

After combining the reported and calculated risk estimates (HR/RR) in a random-effects meta-analysis, the overall risk estimate indicated that men with CKD were marginally at higher risk of cardiovascular mortality compared to women with CKD (RE 1.13, 95%CI 1.03–1.25), with moderate heterogeneity (I^2^ = 35.89%; see Fig 4).

### Subgroup analysis

Table 4 summarises the results of the subgroup analyses. There was no statistically significant difference in risk estimate between any of the subgroups. When restricted to the 28 high-quality studies alone, risk was elevated for men (RE 1.18 95%CI 1.08, 1.27) with very low heterogeneity between studies (I^2^ = 0%).

**Table 4:** Risk estimate (RE) of cardiovascular disease, stratified by study characteristics.

Subgroup	Studies (*n*)	RE (95% CI)	I^2^ (%)
Overall	48	1.13 (1.03, 1.25)	35.89
Sample size			
>1000	6	1.23 (1.15, 1.33)	17.31
100–999	37	1.09 (0.94, 1.26)	33.40
<100	5	1.18 (0.77, 1.81)	24.32
Length of follow-up (years)			
>5 years	8	1.20 (0.90, 1.59)	20.19
2–5	35	1.10 (0.99, 1.22)	35.13
<2	5	1.41 (0.84, 2.37)	54.80
Men: women ratio			
1.2–2.8	30	1.09 (0.98, 1.21)	8.17
0.9–1.1	12	1.16 (0.93, 1.44)	25.65
0.4–0.8	6	1.41 (1.01, 1.96)	25.60
Geographical distribution			
Europe	12	1.20 (0.97, 1.48)	37.30
China	11	1.18 (0.85, 1.63)	41.17
Taiwan	9	1.10 (0.87, 1.38)	9.59
Japan	8	1.07 (0.76, 1.50)	34.22
Korea	3	0.62 (0.37, 1.02)	0.00
United States	3	1.28 (1.21, 1.35)	0.00
Australia and New Zealand	1	0.66 (0.35, 1.24)	-
Africa	1	0.83 (0.39, 1.77)	-
Study quality			
High	28	1.18 (1.08, 1.27)	0.00
Low	20	1.04 (0.86, 1.26)	42.32
Source of risk estimate			
HR	38	1.13 (1.03, 1.25)	35.93
RR	10	1.16 (0.93, 1.44)	42,70
By dialysis modality			
HD	30	1.14(1.05, 1.24)	0.00
PD	8	0.76 (0.55, 1.05)	13.09
Both HD, PD	3	1.30 (0.80, 2.13)	34.89
No dialysis	6	1.34 (1.00, 1.80)	45.27
Adjustment for other risk factors (only includes studies where HR was reported)			
Adjusted	15	1.21 (0.97, 1.50)	60.50
Unadjusted	23	1.03 (0.88, 1.21)	12.13

Note: I^2^ = I^2^ statistic for the measurement of heterogeneity among risk estimates.

**Fig 5:**
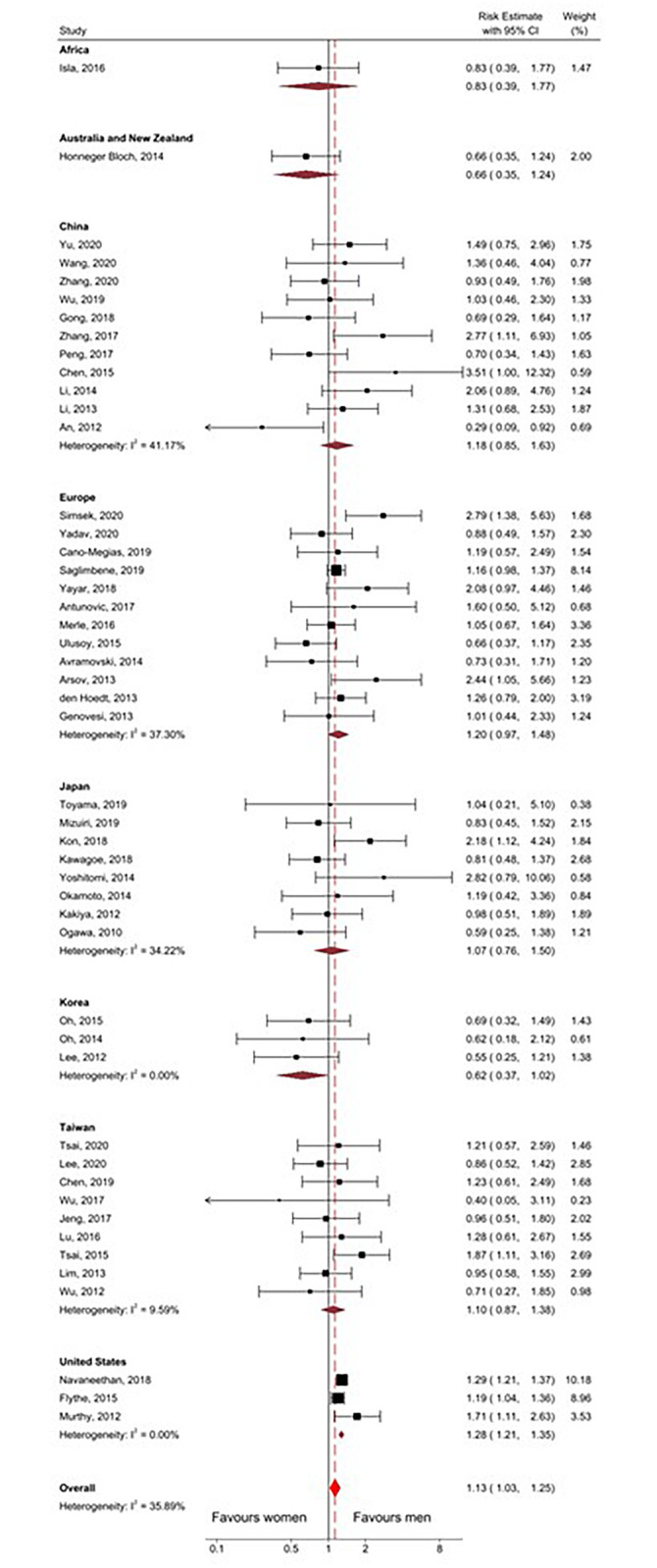
Forest plot of subgroup analysis by country of risk estimates of all included studies. CI, confidence interval. I^2^, Measure of heterogeneity.

**Fig 6:**
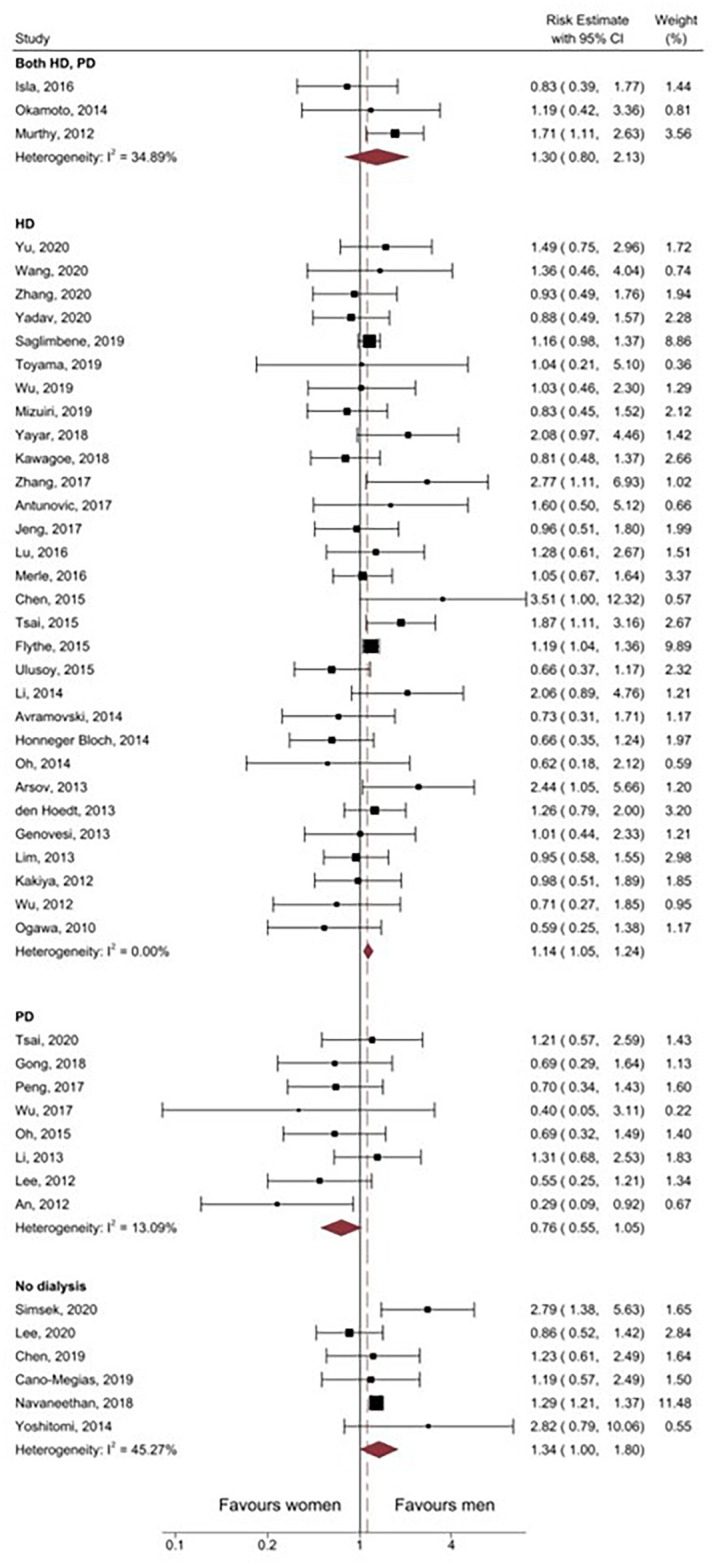
Forest plot of subgroup analysis by dialysis modality of risk estimates of all included studies. CI, confidence interval. I^2^, Measure of heterogeneity. HD, Haemodialysis. PD, Peritoneal dialysis.

Subgroup analysis by measure of association (HR versus RR) revealed that there was no meaningful difference in estimate of sex effect on the method of calculation [HR 1.13 (95%CI 1.03–1.25) vs. RR 1.16 (95%CI 0.93–1.44)]. Subgroup analysis by adjusted versus unadjusted HRs revealed that the association did not reach statistical significance for either group [adjusted HR 1.21 (95%CI 0.97–1.50) vs. unadjusted HR 1.03 (95%CI 0.88–1.21)]. The direction of association varied between countries with the highest increased risk for men reported in studies from the United States (RE 1.28 95%CI 1.21–1.35). In all Korean studies, the point estimate for risk indicated greater mortality in women, although this did not reach statistical significance (RE 0.62, 95%CI 0.37–1.02; Fig 5). Analysis stratified by male-to-female ratio showed that CVD mortality was only greater in studies which included more women (M: F ratio 0.4–0.8; RE 1.43 95%CI 1.15–1.78). Additional analysis by dialysis modality suggested that peritoneal dialysis was associated with lower cardiovascular mortality risk among men; however, this did not reach statistical significance (RE 0.76, 95% CI 0.55–1.05; Fig 6).

### Publication bias

**Fig 7:**
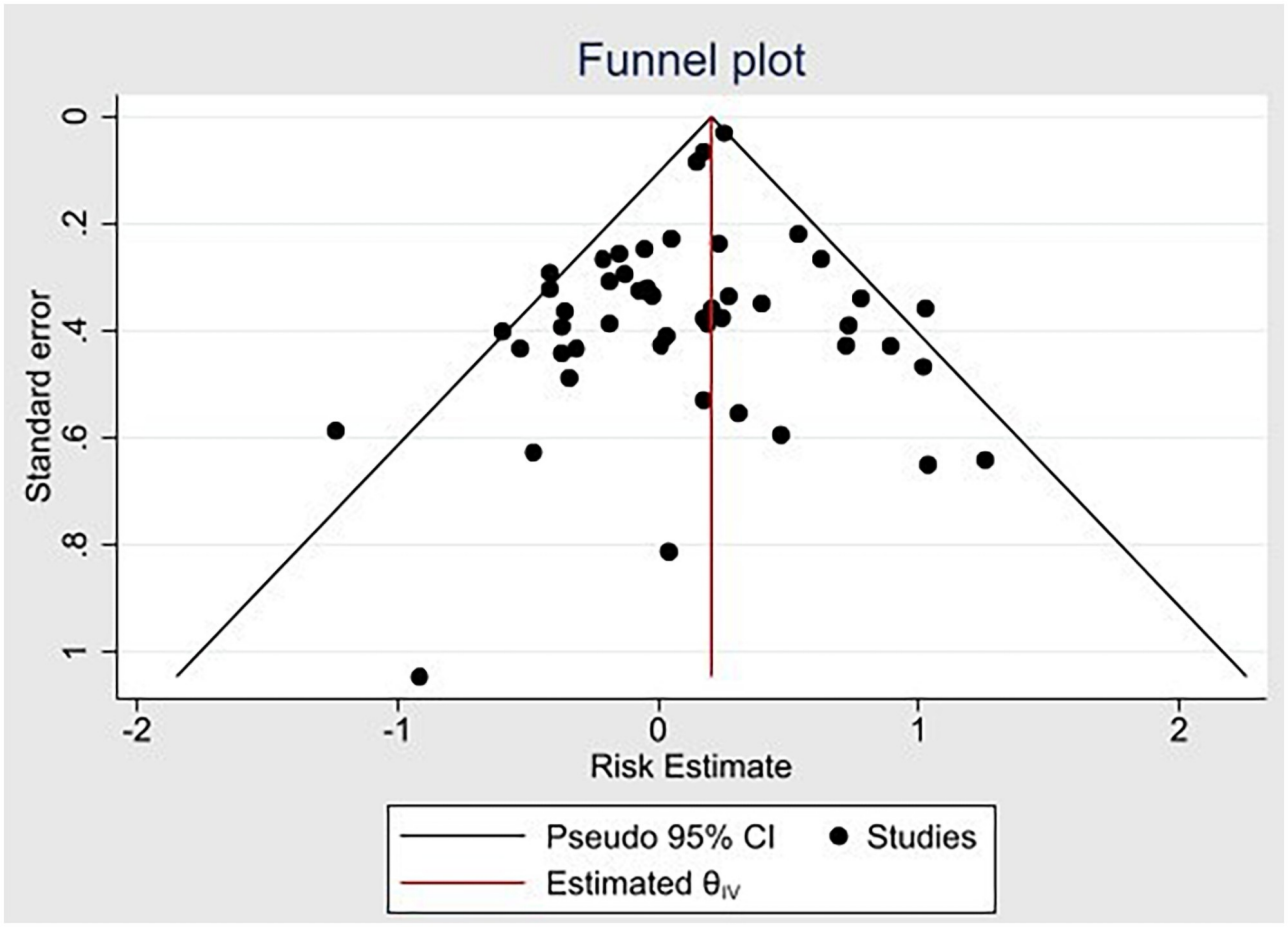
Funnel plot showing publication bias across all studies. CI, confidence interval. Estimated θ_IV_, Estimated effect-size line.

Fig 7 shows the funnel plot (with pseudo 95% CI) used to assess publication bias, with the y-axis representing standard error of effect estimate and x-axis representing individual study effect estimates. Scattering of the effect estimates demonstrates a wide range of standard errors, and the symmetrical shape shows that there was no significant publication bias.

## Discussion

This systematic review and meta-analysis provide a comprehensive analysis of the association of sex and cardiovascular mortality among CKD patients with patient data collected from 2004 onwards. Data from 48 original publications encompassing a large total study population and lengthy follow-up time showed men were at marginally higher risk of cardiovascular mortality than women among the CKD population, with borderline significance. Though this significance was lost when considering only calculated risk ratios, combining the two groups (pre-calculated and calculated) showed borderline significance. Subgroup analysis of only adjusted rates (HR) also showed a marginally higher risk in men, though the association was not statistically significant.

Contrary to the combined result, subgroup analysis by male-to-female ratio showed a comparable risk of cardiovascular mortality among men and women when the distribution of men and women were primarily equal or favoured male participant, suggesting that a higher proportion of either men or women in the study population may bias the overall association. Country-based subgroup analysis indicated that men with CKD in Korea may have less risk of cardiovascular mortality compared to women with CKD; however, the association was not statistically significant. Several studies have shown that although myocardial infarction incidence is higher in Korean men in the overall Korean population, in-hospital mortality for MI is higher in women [63–65]. For our meta-analysis, there were only three studies conducted in Korea with all patient recruitment and follow-up completed by the end of 2012/early 2013. It would be important to consider if this relationship exists currently and when conducted on a large study population. Contrasting the observations from the Korean studies, our analysis clearly supports the assertion that male patients with CKD from the United States are at elevated risk of cardiovascular mortality. This subgroup analysis included two very large-scale studies (>10000 subjects each) [21, 36] and one moderately sized study (100–999) [18], which on balance had an even ratio of male to female participants and included both dialysis-dependent and non-dialysis-dependent CKD patients. The reasons underlying this clearly elevated risk are unknown; however, may relate to known co-morbidities such as hypertension, diabetes, or coronary artery disease, which were prevalent in these studies. Finally, subgroup analysis by dialysis modality revealed that men had higher cardiovascular mortality risk when considering only haemodialysis or non-dialysis-dependent patients whereas there was equivalent risk when only peritoneal dialysis patients are considered. Peritoneal dialysis, relative to haemodialysis, is associated with an increased risk of mortality [66, 67] and therefore further research is required to understand why our subgroup analysis demonstrated that there was no difference in cardiovascular mortality risk when patients received peritoneal dialysis i.e., was risk actually reduced in male patients receiving peritoneal dialysis relative to haemodialysis or increased in female patients.

Historically, reports of cardiovascular mortality risk within the general population have been much higher in men, with age-adjusted cardiovascular mortality reported to be as much as 80% higher in men [6, 68, 69]. However, this increased cardiovascular protection in women within the general population cannot be extrapolated to women with CKD. Our findings suggest that given that cardiovascular risk was only marginally higher in men, the protective effect of female sex is significantly reduced in women with CKD. In support of this, previous meta-analyses looking at the association of sex with cardiovascular mortality among both the general population and CKD cohorts showed that although men in both groups had higher cardiovascular mortality at all levels of eGFR, the slope of the risk relationship for cardiovascular mortality rose more rapidly in women than in men with decreasing eGFR [7]. Similarly, in a cohort study looking at incident adult dialysis patients, women receiving dialysis had a higher cardiovascular mortality risk than women in the general population (eGFR > 90) [8]. Future studies are required to comprehensively compare the relative risk of cardiovascular mortality for men and women with CKD relative to the general population.

There were several strengths to this systematic review: 1. the literature search included several large databases with the search criteria designed to identify as many relevant articles as possible; 2. a proportion of the study selection, data extraction and quality assessment were conducted in duplicate by separate reviewers to reduce reporting bias; 3. none of the included studies addressed sex differences specifically in their primary study objectives and, therefore, there was less potential for selection and publication bias with respect to our study question. Because the included studies’ primary study objectives were different from our research question, we adapted the Risk of Bias tool to evaluate our research question’s quality of data rather than the reported outcomes in the original study; and finally, 4. our study was widely representative of the ESRD disease population, allowing us to determine whether sex differences in cardiovascular mortality exist based on dialysis modality.

There are some limitations to our study that need to be taken into consideration. Notably, most studies did not report sex-stratified proportions of cardiovascular deaths or sex-stratified cardiovascular mortality rates. To overcome this pre-calculated HR from these studies were used in the meta-analysis. In the ten studies where source data was available, RR was calculated, and subgroup analysis based on the measure of association (HR versus RR) conducted to assess whether the method of measure of association contributed to differences in study outcome. This analysis showed similar results regardless of the method of assessment. Cause-specific cardiovascular mortality was only reported in three studies [21, 27, 43], and no subgroup analysis on the cause of cardiovascular death could be performed. In addition, there was some heterogeneity among risk/rate estimates from the included studies, possibly due to some bias at the study level. Possible sources of bias included unequal distribution of men and women and variation in follow-up time. However, subgroup analysis by variables was done to assess these sources of heterogeneity.

In conclusion, the findings from this systematic review and meta-analysis, covering study populations with substantial follow-up, show that male sex is marginally associated with higher cardiovascular mortality among CKD patients. Although men with CKD showed overall higher cardiovascular mortality risk than women with CKD, the increased risk was minimal, and appropriate risk awareness is necessary for both sexes to reduce exposure to cardiovascular risk factors, facilitate early diagnosis of complications, and ensure better cardiovascular health outcomes for both sexes.

## Supporting information

S1 Checklist PRISMA checklist. (PDF)

S1 Data Pubmed/medline search strategy. (DOCX)

S2 Data Newcastle-Ottawa quality assessment scale. (PDF)

S3 Data Cochrane data extraction template. (DOCX)

S1 Table List of excluded studies with reasons for exclusion. (DOCX)

S2 Table Risk of bias summary for individual studies. (DOCX)
