# Supplementary material for: Relationship between sex and cardiovascular mortality in chronic kidney disease: A systematic review and meta-analysis
Source: PLoS One. 2021 Jul 12;16(7):e0254554. doi: 10.1371/journal.pone.0254554 (PMC8274915; doi:10.1371/journal.pone.0254554)
Supplement: S1 Data — (DOCX) [file pone.0254554.s002.docx]

**S1 Data. PubMed/Medline search strategy**

**PubMed Search Strategy**

(("Renal Insufficiency, Chronic"[Mesh]) OR ("Uremia"[Majr:NoExp]) OR ("chronic kidney disease"[All Fields]) OR ("chronic renal disease"[All Fields]) OR ("chronic kidney insufficiency"[All Fields]) OR ("chronic renal insufficiency"[All Fields]))

AND

(("Cardiovascular Diseases/mortality"[Mesh]) OR ("cardiovascular death"[All Fields]) OR ("cardiovascular mortality"[All Fields]) OR ("cardiovascular event*"[All Fields]) OR ("cardiovascular outcome*"[All Fields]) OR ("cardiovascular complication*"[All Fields]))

AND

(("Sex Factors"[Mesh]) OR ("Male"[Mesh]) OR ("Female"[Mesh]) OR ("Men"[Mesh]) OR ("Women"[Mesh]) OR ("Sex Distribution"[Mesh]))

**Medline Search Strategy**

1. renal insufficiency, chronic/ or kidney failure, chronic/

2. (chronic kidney adj (disease or insufficiency)).ti,ab.

3. chronic renal disease.ti,ab.

4. chronic renal insufficiency.ti,ab.

5. end-stage renal disease.ti,ab.

6. uraemia.ti,ab.

7. uremia.ti,ab.

8. Uremia/

9. 1 or 2 or 3 or 4 or 5 or 6 or 7 or 8

10. Cardiovascular Diseases/mo [Mortality]

11. (cardiovascular adj (mortality or death or event* or complication* or outcome*)).ti,ab.

12. Heart Diseases/mo [Mortality]

13. 10 or 11 or 12

14. Sex Factors/

15. Male/ or Female/

16. Sex Distribution/ or Sex Characteristics/ or Sex Ratio/

17. Men/ or Women/

18. (men or women or male* or female* or sex or gender or "sex difference*").mp

19. 14 or 15 or 16 or 17 or 18

20. 9 and 13 and 19

21. limit 20 to english language
