# Supplementary material for: Relationship between sex and cardiovascular mortality in chronic kidney disease: A systematic review and meta-analysis
Source: PLoS One. 2021 Jul 12;16(7):e0254554. doi: 10.1371/journal.pone.0254554 (PMC8274915; doi:10.1371/journal.pone.0254554)
Supplement: S3 Data — (DOCX) [file pone.0254554.s004.docx]

**S3 Data. Cochrane Data Extraction Template**

**Cochrane Public Health Group Data Extraction and Assessment Template (*modify* to suit your review)**

| **Study ID:** | **Report ID :** | Date form completed: |
| --- | --- | --- |
| First author: | Year of study: | Data extractor: |
| Citation: | | |

**1. General Information**

| Publication type Journal Article ⬜ Abstract ⬜ Other (specify e.g. book chapter)___________________ | |
| --- | --- |
| Country of study: | |
| Funding source of study: | Potential conflict of interest from funding? Y / N / unclear |

**2. Study Eligibility**

| **Study Characteristics** | | | | **Page/ Para/ Figure #** |
| --- | --- | --- | --- | --- |
| **Type of study**  (Review authors to add/remove designs based on criteria specified in protocol) | ⬜ Randomised Controlled Trial (RCT)  ⬜ Cluster Randomised Controlled Trial (cluster RCT) | | ⬜ Controlled Before and After (CBA) study   - Contemporaneous data collection - Comparable control site - At least 2 x intervention and 2 x control clusters |  |
|  | ⬜ Interrupted Time Series (ITS)   - At least 3 time points before and 3 after the intervention - Clearly defined intervention point | | ⬜ Other design (specify): |  |
|  | ⬜ A process evaluation of an included study design | | *Does the study design meet the criteria for inclusion?*  Yes ⬜ No ⬜ 🡪**Exclude** Unclear ⬜ |  |
|  | Description in text: | | |  |
| **Participants**  (Review authors insert inclusion criteria as defined in Protocol) | Describe the participants included: | | |  |
|  | Are participants defined as a group having specific social or cultural characteristics? | Yes ⬜ No ⬜ Unclear ⬜  Details: | |  |
|  | How is the geographic boundary defined? | Details:  Specific location (e.g. state / country): | |  |
|  | *Do the participants meet the criteria for inclusion?* | Yes ⬜ No ⬜ 🡪**Exclude** Unclear ⬜ | |  |

| **Types of intervention**  (Review authors insert inclusion criteria as defined in Protocol) | Strategies included in the intervention | |  | |  |
| --- | --- | --- | --- | --- | --- |
|  | Focus of the intervention | |  | |  |
|  | *Does the intervention meet the criteria for inclusion?* | | Yes ⬜ No ⬜ 🡪**Exclude** Unclear ⬜ | |  |
| **Duration of intervention** | Start date: | Stop date: | | Intervention duration: |  |
|  | *Is the duration of intervention adequate for inclusion?* | | Yes ⬜ No ⬜ 🡪**Exclude** Unclear ⬜ | |  |
| **Types of outcome measures**  (Review authors insert inclusion criteria as defined in Protocol) | List outcomes: | |  | |  |
|  | Outcome measured at a population level or individual level? | | Details: | |  |
|  | *Do the outcome measures meet the criteria for inclusion?* | | Yes ⬜ No ⬜ 🡪**Exclude** Unclear ⬜ | |  |

**Summary of Assessment for Inclusion**

| **Include in review ⬜ Exclude from review ⬜** | |
| --- | --- |
| Independently assessed, and then compared? Yes ⬜ No ⬜ | Differences resolved Yes ⬜ No ⬜ |
| Request further details? Yes ⬜ No ⬜ | Contact details of authors: |
| **Notes:** | |

DO NOT PROCEED IF PAPER EXCLUDED FROM REVIEW

**3. Study details**

| **Study intention** | **Descriptions as stated in the report/paper** | **Page/ Para/ Figure #** |
| --- | --- | --- |
| Aim of intervention | *What was the problem that this intervention was designed to address?* |  |
| Aim of study | *What was the study designed to assess? Are these clearly stated?* |  |
| Equity pointer: Social context of the study | *e.g. was study conducted in a particular setting that might target/exclude specific population s? See also Inclusion/exclusion criteria under Methods, below.* |  |
| Start and end date of the study | *Identify which elements of planning of the intervention should be included* |  |
| Total study duration |  |  |

| **Methods** | **Descriptions as stated in the report/paper** | **Page/ Para/ Figure #** |
| --- | --- | --- |
| Method/s of recruitment of participants  *(How were potential participants approached and invited to participate? Where were participants recruited from? Does this differ from the intervention setting?)* |  |  |
| Inclusion/exclusion criteria for participation in study |  |  |
| Representativeness of sample: Are participants in the study likely to be representative of the target population? |  |  |
| Total number of intervention groups |  |  |
| Assumed risk estimate  (*e. .baseline or population risk noted in Background)* | *References:* |  |
| Sample size calculation:  What assumptions were made?  Were these assumptions appropriate? | *(Yes/No/Unclear)* |  |
| What was the unit of randomisation?  Allocation by individuals or cluster/groups |  |  |
| What was the unit of analysis?  **Is this the same as the unit of randomisation?** | *(Yes/No/Unclear)* |  |
| Statistical methods used and appropriateness of these methods | *(Check with your statistician if unsure about appropriateness)* |  |

**Results**

| **Participants**  *Include if relevant* | **Include information for each group (i.e. intervention and controls) under study** | **Page/ Para/ Figure #** |
| --- | --- | --- |
| 1. What percentage of selected individuals agreed to participate? |  |  |
| 1. Total number randomised (or total pop. at start of study for NRCTs) |  |  |
| 1. Number allocated to each intervention group (no. of individuals) |  |  |
| 1. For cluster trials, number of clusters, number of people per cluster |  |  |
| 1. Where there any significant baseline imbalances? | Yes ⬜ No ⬜ Unclear ⬜  Details: |  |
| 1. Number and reason for (and sociodemographic differences of) withdrawals and exclusions for each intervention group |  |  |
| 1. Were patients who entered the study adequately accounted for? |  |  |
| 1. What percentage of patients completed the study? |  |  |
| 1. What percentage of participants received the allocated intervention or exposure of interest? |  |  |
| 1. Is the analysis performed by intervention allocation status (intention to treat) rather than the actual intervention received? Have any attempts been made to impute missing data? |  |  |
| 1. Age (median, mean and range if possible) |  |  |
| 1. Sex |  |  |
| 1. Race/Ethnicity |  |  |
| 1. Principal health problem (incl. stage of illness) |  |  |
| 1. Diagnostic criteria |  |  |
| 1. Co-morbidity |  |  |
| 1. Other sociodemographics (eg. Educational level, literacy level, soci-economic status, first language. Also consider possible proxies for these e.g. low baseline nutritional status ) |  |  |
| 1. PROGRESS categories reported at baseline (indicate letters of those reported: Place of residence, race, occupation, gender, religion, education, SES, social capital) |  |  |
|  |  |  |
| **Subgroups** | *Enter a description of any participant subgroups from this paper to be analysed in the review.* |  |

**Intervention Group 1***(copy and paste table for each Intervention group)*

| **Group name:** | *(State brief name for this intervention group.)* | **Page/ Para/ Figure #** |
| --- | --- | --- |
| Details of intervention or control condition *(Include if relevant in sufficient detail for replication)* | | |
| - Setting *eg multicentre, university teaching hospitals, rural, metropolitan, school, workplace, community, GP clinic, etc.* |  |  |
| - Theoretical basis (include key references) |  |  |
| - Content (list the strategies intended and delivered) |  |  |
| - Did the intervention include strategies to address diversity/disadvantage? | *Enter a description of any relevant strategies* |  |
| - Delivery (eg. Stages (sequential or simultaneous), timing, frequency, duration, intensity, fidelity – process indicators) |  |  |
| - Providers (who, number, education/training in intervention delivery, ethnicity etc. if potentially relevant to acceptance and uptake by participants |  |  |
| - Co-interventions |  |  |
| Duration of intervention |  |  |
| Duration of follow-up |  |  |
| Was sustainability discussed by the authors? Was is a consideration in study development? |  |  |
| Economic variables ie costs of the intervention, and changes in other (eg health care) costs as result of intervention^^[[1]](#footnote-1)^♠^ | Yes ⬜ 🡪**List in Outcome section if appropriate**  No ⬜ Unclear ⬜  Details: |  |
| Other economic information (from a societal, non-healthcare view – e.g. lost wages, time) | Yes ⬜  No ⬜  Details: |  |
| Resource requirements to replicate intervention (e.g. staff numbers, hours of implementation, equipment?) |  |  |
| Subgroups | *Enter a description of any intervention subgroups from this report to be analysed in the review.* |  |
| What are the moderators/mediators of changes stated in the study? |  |  |
| Do the authors describe any political or organisational context? | *List relevant dot points* |  |
| Were any partnerships referred to? | *List these as dot points* |  |
| Was a process evaluation conducted? | *What components were included in the process evaluation? (eg. dose, frequency, consistency, implemented as intended etc)* |  |
| Control/comparison (what information is provided about what the control or comparison group received?) | *Enter a description of what was provided for the control group, if applicable* |  |

**Outcomes**

*(This table is set up for 2 outcome measure to save spaces, copy and paste table as often as required*)

| **Question** | **Outcome 1** | **Page/ Para/ Figure #** | **Outcome 2** | **Page/ Para/ Figure #** |
| --- | --- | --- | --- | --- |
| Is there an analytic framework applied (e.g. logic model, conceptual framework)? |  |  |  |  |
| Outcome definition (with diagnostic criteria if relevant) |  |  |  |  |
| Type of outcome: Is this a modifiable variable (Community level, neighbourhood level, individual level) or desired health outcome |  |  |  |  |
| Time points measured |  |  |  |  |
| Time points reported |  |  |  |  |
| Is there adequate latency for the outcome to be observed? |  |  |  |  |
| Is the measure repeated on the same individuals or redrawn from the population / community for each time point? |  |  |  |  |
| Unit of measurement (if relevant) |  |  |  |  |
| For scales – upper and lower limits and indicate whether high or low score is good |  |  |  |  |
| How is the measure applied? Telephone survey, mail survey, in person by trained assessor, routinely collected data, other |  |  |  |  |
| How is the outcome reported? Self or study assessor |  |  |  |  |
| Is this outcome/tool validated? |  |  |  |  |
| …And has it been used as validated? |  |  |  |  |
| Is it a reliable outcome measure? |  |  |  |  |
| Is there adequate power for this outcome? |  |  |  |  |
|  |  |  |  |  |
| Were PROGRESS categories analysed by outcome? Indicate the letters of those that outcomes were analysed by (place of residence, race, occupation, gender, religion, education, SES, social capital) |  |  |  |  |

**Results**

*Copy and paste the appropriate table for each outcome and subgroup at each timepoint, including baseline*

**For RCT/CCT**

**Dichotomous outcome** page/para/fig

| Comparison |  | | | |  |
| --- | --- | --- | --- | --- | --- |
| Outcome |  | | | |  |
| Subgroup |  | | | |  |
| Timepoint |  | | | |  |
| **Results** | **Intervention** | | **Comparison** | |  |
|  | Events | No. participants | Events | No. participants |  |
|  |  |  |  |  |  |
| No. of missing participants and reasons |  | |  | |  |
| Any other results reported |  | | | |  |
| Reanalysis required? (specify -  (e.g. correlation adjustment) |  | | | |  |
| Reanalysis possible? | *yes/no/unclear* | | | |  |
| Reanalysed results |  | | | |  |

**For RCT/CCT**

**Continuous outcome** page/para/fig

| Comparison |  | | | | | |  |
| --- | --- | --- | --- | --- | --- | --- | --- |
| Outcome |  | | | | | |  |
| Subgroup |  | | | | | |  |
| Timepoint |  | | | | | |  |
| Post-intervention or change from baseline? |  | | | | | |  |
| **Results** | **Intervention** | | | **Comparison** | | |  |
|  | Mean | SD (or other variance) | No. participants | Mean | SD (or other variance) | No. participants |  |
|  |  |  |  |  |  |  |  |
| No. missing participants and reasons |  | | |  | | |  |
| Any other results reported |  | | | | | |  |
| Reanalysis required? (specify) |  | | | | | |  |
| Reanalysis possible? | *yes/no/unclear* | | | | | |  |
| Reanalysed results |  | | | | | |  |

**For RCT/CCT**

**Generic inverse variance method**

Page/para/figure

| Comparison |  | | | |  |
| --- | --- | --- | --- | --- | --- |
| Outcome |  | | | |  |
| Subgroup |  | | | |  |
| Timepoint |  | | | |  |
| Results | Effect estimate | SE (or other variance) | Intervention no. | Control no. |  |
|  |  |  |  |  |  |
| No. missing participants and reasons |  | | | |  |
| Any other results reported |  | | | |  |
| Reanalysis required? (specify) |  | | | |  |
| Reanalysis possible? | *yes/no/unclear* | | | |  |
| Reanalysed results |  | | | |  |

**For CBA**

Page/para/fig

| Comparison |  | |  |
| --- | --- | --- | --- |
| Assignment | How were control and treatment groups selected?? Is there likely to be an effect if these were the opposite way? | |  |
|  | Contemporaneous data collection? | |  |
| Outcome |  | |  |
| Subgroup |  | |  |
| Timepoint |  | |  |
| Post-intervention or change from baseline? |  | |  |
|  | **Intervention** | **Comparison** |  |
| No. participants  measured |  |  |  |
| No. missing participants and reasons |  |  |  |
| Baseline result (with variance measure) |  |  |  |
| Post-intervention results (with variance measure) |  |  |  |
| Change (Post – baseline) (with variance measure) |  |  |  |
| Difference in change (intervention – control) (with variance measure) |  | |  |
| Any other results reported |  | |  |
| Reanalysis required? (specify) |  | |  |
| Reanalysis possible? | *yes/no/unclear* | |  |
| Reanalysed results |  | |  |

**For ITS**

**Generic inverse variance method** Page/para/fig

| Comparison |  | | | | |  |
| --- | --- | --- | --- | --- | --- | --- |
| Outcome |  | | | | |  |
| Subgroup |  | | | | |  |
| Length of timepoints measured |  | | | | |  |
| Snapshot or interval measured |  | | | | |  |
| No. participants measured |  | | | | |  |
| No. missing participants and reasons |  | | | | |  |
|  | Pre-intervention | | | Post-intervention | |  |
| No. of timepoints measured |  | | |  | |  |
| Mean value (with variance measure) |  | | |  | |  |
| Difference in means (post – pre) |  | | | | |  |
| Percent relative change |  | | | | |  |
| Result reported by authors (with variance measure) |  | | | | |  |
| Reanalysis required? (specify) |  | | | | |  |
| Reanalysis possible? | *yes/no/unclear* | | | | |  |
| Individual time point results |  | | | | |  |
| Read from figure? | *yes/no* | | | | |  |
| Reanalysed results | Change in level | SE | Change in slope | | SE |  |
|  |  |  |  | |  |  |

**Other relevant information**

| Were outcomes relating to harms/unintended effects of the intervention described? Include any data for these in the outcomes tables above |  |  |  |
| --- | --- | --- | --- |
| Potential for author conflict *ie. evidence that author or data collectors would benefit if results favoured the intervention under study or the control* |  |  |  |
| Key conclusions of the study authors |  |  |  |
| Could the inclusion of this study potentially bias the generalisability of the review? Equity pointer: Remember to consider whether disadvantaged populations may have been excluded from the study. |  | | |
| Is there potential for differences in relative effects between advantaged and disadvantaged populations? (e.g. are children from lower income families less likely to wear bicycle helmets) |  | | |
| Are interventions likely to be aimed at the disadvantaged? (e.g. school meals aimed at poor children). |  | | |
| Issues affecting directness  (*Note any aspects of population, intervention, etc. that affect this study’s direct applicability to the review question)* |  | | |
| References to other relevant studies |  | | |
| Additional notes by review authors |  | | |
| Correspondence required for further study information (from whom, what and when) |  | | |

**Adapted data extraction form with Risk of Bias tool (Newcastle-Ottawa Scale)**

| 1. General Information | | |
| --- | --- | --- |
|  | Study ID (e.g. Smith 2001) |  |
|  | Report title (title of paper/ abstract/ report) |  |
|  | First Author |  |
|  | Year of study |  |
|  | Date form completed |  |
|  | Data extractor |  |
|  | Publication Type |  |
|  | Country of Study |  |
|  | Funding source |  |
|  | Potential conflict of interest from funding Y/N/Unclear |  |
|  | Notes: |  |
| 2. Study Eligibility | | |
| Inclusion criteria | |  |
| *Type of participants* | |  |
|  | Patients population (CKD/Dialysis/Renal Impairment/Decreased GFR <60ml…) |  |
|  | Both sexes |  |
|  | Aged 18 and older |  |
|  | Stage of CKD (Any) |  |
|  | Patient data collected after 2004 |  |
| *Type of studies* | |  |
|  | Non-interventional cohort study (prospective/retrospective)/ Cross-sectional/ Case Control, Observational study/ Control arm of RCT with appropriate sex stratification/ Systematic Review/ Meta-analysis/ Grey literature - ANZDATA |  |
| *Type of outcomes* | |  |
|  | Cardiovascular mortality (overall and/or cause-specific) stratified by sex/gender |  |
|  | Follow-up duration > 1 year |  |
|  | Full-text available |  |
|  | Publication Language English |  |
|  | Published after 2004 |  |
|  | Exclusion criteria |  |
| *Type of participants* | |  |
|  | Studies looking exclusively at type 1 and type 2 diabetes mellitus |  |
|  | Following conditions associated with the study population at the beginning of the study: 1. Infection 2. Carcinoma 3. Acute Kidney Injury 4. Kidney transplant recipients 5. Surgical interventions or non-conventional drug treatments |  |
|  | Age less than 18 years |  |
|  | Patient data was collected before 2004 |  |
| *Type of studies* | |  |
|  | Review |  |
|  | Others: letters, author's comments/replies |  |
|  | Studies which derived data from ANZDATA |  |
|  | Interventional studies (surgical intervention e.g. CABG, PCI etc. or non-conventional drug trials with no control group) |  |
| *Type of outcome* | |  |
|  | Did not report CV mortality stratified by sex in CKD patients |  |
|  | No results published |  |
| 3. Population and setting | | |
|  | Population description  Source/setting of the population  (e.g. country, urban, rural, particular ethnic  group)  20. Method/s of recruitment of  participants  Notes: |  |
| 4. Methods | | |
|  | Aim of study |  |
|  | Design |  |
|  | Sampling technique (inclusion, exclusion criteria) |  |
|  | Study duration |  |
|  | Notes: |  |
| 5. Participants | | |
|  | Description of condition (CKD/Dialysis etc.) |  |
|  | Total number of participants with CKD (analysed for cardiovascular mortality) |  |
|  | *Males with CKD* |  |
|  | *Females with CKD* |  |
|  | Stage of CKD (GFR ml/min) |  |
|  | Stage 1 >90 |  |
|  | Stage 2 60 -89 |  |
|  | Stage 3 30-59 |  |
|  | Stage 4 15-29 |  |
|  | Stage 5/ ESRD  <15 |  |
|  | Dialysis (Y/N) |  |
|  | If Dialysis (Y), did the whole cohort receive dialysis? |  |
|  | Average Age in Years of the population of interest |  |
|  | Length of follow-up (years) avg. |  |
|  | Number of lost to follow-up |  |
|  | Baseline comorbidities in CKD patients (%) |  |
|  | Notes: |  |
| 6. Outcomes | |  |
|  | Measure of cardiovascular mortality stratified by sex (overall) |  |
|  | Definition of CV mortality in the study |  |
|  | Cause-specific cardiovascular mortality stratified by sex (heart failure/atrial fibrillation/myocardial infarction/stroke, sudden cardiac arrest etc.) |  |
|  | How data was collected |  |
|  | Notes: |  |
| 7. Results and Findings | | |
|  | Cardiovascular mortality stratified by sex | |
|  | Number |  |
|  | Total |  |
|  | *Male* |  |
|  | *Female* |  |
|  | Proportion of CVD deaths % |  |
|  | *Male* |  |
|  | *Female* |  |
|  | Mortality rate (as reported in the study) |  |
|  | *Male* |  |
|  | *Female* |  |
|  | Mortality rate (per 1000 person years) |  |
|  | *Male* |  |
|  | *Female* |  |
|  | *Male vs Female* Hazard Ratio for CV mortality (95% CI) |  |
|  | *Univariate/Unadjusted* |  |
|  | *95% CI* |  |
|  | *P-value* |  |
|  | *Multivariate/Adjusted* |  |
|  | *95% CI* |  |
|  | *P-value* |  |
|  | Sex-Stratified hazard ratio relative to the reference cell category of eGFR >90 |  |
|  | *GFR category as defined in the study (when applicable)* |  |
|  | *Male* |  |
|  | *95% CI* |  |
|  | *P-value* |  |
|  | *Female* |  |
|  | *95% CI* |  |
|  | *P-value* |  |
|  | Risk Ratio |  |
|  | *Male* |  |
|  | *Female* |  |
|  | Odds Ratio |  |
|  | *Male* |  |
|  | *Female* |  |
|  | Statistical methods used |  |
|  | Adjustments |  |
|  | Other form of measurement |  |
|  | *Male* |  |
|  | *Female* |  |
|  | Cause-specific mortality stratified by sex | |
|  | Method of measurement |  |
|  | *Heart Failure* |  |
|  | *Male* |  |
|  | *Female* |  |
|  | *Atrial Fibrillation* |  |
|  | *Male* |  |
|  | *Female* |  |
|  | *Myocardial Infraction* |  |
|  | *Male* |  |
|  | *Female* |  |
|  | *Stroke* |  |
|  | *Male* |  |
|  | *Female* |  |
|  | *Other cause* |  |
|  | *Male* |  |
|  | *Female* |  |
|  | All-cause mortality | |
|  | *Male* |  |
|  | *Female* |  |
|  | *Male vs Female HR* |  |
|  | *Univariate/Unadjusted* |  |
|  | *95% CI* |  |
|  | *P-value* |  |
|  | *Multivariate/Adjusted* |  |
|  | *95% CI* |  |
|  | *P-value* |  |
|  | Notes: |  |
| 8. Risk of bias (Quality Assessment) adapted from the Newcastle-Ottawa Scale (NOS) | | |
| A. Selection | | |
|  | 1. Representativeness of the number of men and women in the CKD cohort  a) Similar distribution of men and women in the study population *  b) mostly men or women  c) no description |  |
| B. Comparability | | |
|  | 1) Study controls for age and/or diabetes mellitus * |  |
|  | 2) Study controls for other confounders * |  |
|  | 3) Study does not adjust for any confounders |  |
| C. Outcome | | |
|  | 1) Assessment of outcome a) Patient medical records * b) record linkage * d) no description |  |
|  | 2) Comprehensive cardiovascular mortality data relative to sex differences (e.g. absolute mortality rates, number of deaths in men and women with due to cardiovascular causes etc.)  a) Comprehensive data was reported *  b) Comprehensive data was not reported |  |
|  | 3) Length of follow-up a) at least 2 years if >60% of the patients had end-stage CKD * b) at least 5 years if <60% of the study population had end-stage CKD * c) Does not satisfy above length of follow-up |  |
|  | 4) Adequacy of follow up a) complete F/U - all subjects accounted for * b) subjects lost to F/U unlikely to introduce bias - small number lost - > 20 % F/U or description provided of those lost) * c) F/U rate < 20% and no description  d) no statement |  |
|  | Notes: |  |
| 9. Conclusion | | |
|  | Conclusions by the authors regarding sex differences in CV mortality in the study population |  |
|  | Notes by review author |  |

1. ♠ Costs associated with the intervention can be linked with provider or participant outcomes in an economic evaluation (depends on the type of economic evaluation) [↑](#footnote-ref-1)
