# Supplementary material for: Relationship between sex and cardiovascular mortality in chronic kidney disease: A systematic review and meta-analysis
Source: PLoS One. 2021 Jul 12;16(7):e0254554. doi: 10.1371/journal.pone.0254554 (PMC8274915; doi:10.1371/journal.pone.0254554)
Supplement: S1 Table — (DOCX) [file pone.0254554.s005.docx]

**S1 Table. List of excluded studies with reasons for exclusion**

| No. | Authors | Title | Year published | Reason for exclusion |
| --- | --- | --- | --- | --- |
| 1 | McKercher, C., Chan, H. W., Clayton, P. A., McDonald, S. and Jose, M. D. | Dialysis outcomes of elderly Indigenous and non-Indigenous Australians | 2014 | CVD mortality not stratified by sex |
| 2 | Neuen, B. L., Leather, N., Greenwood, A. M., Gunnarsson, R., Cho, Y. and Mantha, M. L. | Neutrophil-lymphocyte ratio predicts cardiovascular and all-cause mortality in hemodialysis patients | 2016 | CVD mortality not stratified by sex |
| 3 | Roberts, M. A., Polkinghorne, K. R., McDonald, S. P. and Ierino, F. L. | Secular trends in cardiovascular mortality rates of patients receiving dialysis compared with the general population | 2011 | CVD mortality not stratified by sex |
| 4 | Ali, O. M., Sayed, A. A., Mohammed, W. S., Mohammed, R. R. | Cardiovascular system affection and its relation to first-year mortality in patients initiating maintenance hemodialysis | 2020 | CVD mortality not stratified by sex |
| 5 | Chen, Z., Zhang, X., Han, F., Xie, X., Hua, Z., Huang, X., Lindholm, B., Haarhaus, M., Stenvinkel, P., Qureshi, A. R., Chen, J. | High alkaline phosphatase and low intact parathyroid hormone associate with worse clinical outcome in peritoneal dialysis patients | 2020 | CVD mortality not stratified by sex |
| 6 | Chiu, H., Wu, P. Y., Huang, J. C., Tu, H. P., Lin, M. Y., Chen, S. C., Chang, J. M. | There is a U shaped association between non high density lipoprotein cholesterol with overall and cardiovascular mortality in chronic kidney disease stage 3-5 | 2020 | CVD mortality not stratified by sex |
| 7 | Feng, X., Wen, Y., Peng, F. F., Wang, N., Zhan, X., Wu, X. | Association between aminotransferase/alanine aminotransferase ratio and cardiovascular disease mortality in patients on peritoneal dialysis: A multi-center retrospective study | 2020 | CVD mortality not stratified by sex |
| 8 | Harada, T., Sorimachi, H., Obokata, M., Sunaga, H., Ishida, H., Ito, K., Ogawa, T., Ando, Y., Kurabayashi, M., Negishi, K. | The relationship between antecedent creatinine decreases and outcomes in patients undergoing hemodialysis | 2020 | CVD mortality not stratified by sex |
| 9 | Dratch, A., Kleine, C. E., Streja, E. Soohoo, M., Park, C., Hsiung, J. T., Rhee, C. M., Obi, Y., Molnar, M. Z., Kovesdy, C. P., Kalantar-Zadeh, K. | Mean Corpuscular Volume and Mortality in Incident Hemodialysis Patients | 2019 | CVD mortality not stratified by sex |
| 10 | Feldreich, T., Nowak, C., Fall, T.,, Carlsson, A. C., Carrero, J. J., Ripsweden, J., Qureshi, A. R., Heimburger, O., Barany, P., Stenvinkel, P., Vuilleumier, N., Kalra, P. A., Green, D., Arnlov, J. | Circulating proteins as predictors of cardiovascular mortality in end-stage renal disease | 2019 | CVD mortality not stratified by sex |
| 11 | Gunthner, R., Hanssen, H., Hauser, C., Angermann, S., Lorenz, G., Kemmner, S., Matschkal, J., Braunisch, M. C., Kuchle, C., Renders, L., Moog, P., Wassertheurer, S., Baumann, M., Hammes, H. P., Mayer, C. C., Haller, B., Stryeck, S., Madl, T., Carbajo-Lozoya, J., Heemann, U. Kotliar, K., Schmaderer, C. | Impaired Retinal Vessel Dilation Predicts Mortality in End-Stage Renal Disease | 2019 | CVD mortality not stratified by sex |
| 12 | Jahn, L., Kramann, R., Marx, N., Floege, J., Becker, M., Schlieper, G. | Speckle Tracking Echocardiography and All-Cause and Cardiovascular Mortality Risk in Chronic Kidney Disease Patients | 2019 | CVD mortality not stratified by sex |
| 13 | Kalousova, M., Dusilova-Sulkova, S., Kubena, A. A., Zakiyanov, O., Tesar, V., Zima, T. | Sclerostin levels predict cardiovascular mortality in long-term hemodialysis patients: A prospective observational cohort study | 2019 | CVD mortality not stratified by sex |
| 14 | Kido, R., Akizawa, T., Fukuhara, S. | Haemoglobin concentration and survival of haemodialysis patients before and after experiencing cardiovascular disease: a cohort study from Japanese dialysis outcomes and practice pattern study (J-DOPPS) | 2019 | CVD mortality not stratified by sex |
| 15 | Lasfar, L. B., Guedri, Y., Zellama, D., El Meknassi, I., Azzebi, A., Mrabet, S., Fradi, A., Toumi, S., Sabri, F., Amor, S. B., Sahtout, W., Achour, A. | Long-term clinical outcomes of peritoneal dialysis patients: 10-year experience of a single unit from Tunisia | 2019 | CVD mortality not stratified by sex |
| 16 | Lu, W., Pang, W. F., Jin, L., Li, H., Chow, K. M., Kwan, B. C., Leung, C. B., Li, P. K., Szeto, C. C. | Peritoneal protein clearance predicts mortality in peritoneal dialysis patients | 2019 | CVD mortality not stratified by sex |
| 17 | Matschkal, J., Mayer, C. C., Sarafidis, P. A., Lorenz, G., Braunisch, M. C., Guenthner, R., Angermann, S., Steubl, D., Kemmner, S., Bachmann, Q., Hauser, C., Nerl, L., Baumann, M., Mann, J. F., Moog, P., Kuechle, C., Renders, L., Heemann, U., Wassertheurer, S., Schmaderer, C. | Comparison of 24-hour and Office Pulse Wave Velocity for Prediction of Mortality in Hemodialysis Patients | 2019 | CVD mortality not stratified by sex |
| 18 | Modi, Z. J., Lu, Y., Ji, N., Kapke, A., Selewski, D. T., Dietrich, X., Abbott, K., Nallamothu, B. K., Schaubel, D. E., Saran, R., Gipson, D. S. | Risk of Cardiovascular Disease and Mortality in Young Adults With End-stage Renal Disease: An Analysis of the US Renal Data System | 2019 | CVD mortality not stratified by sex |
| 19 | Nemcsik, J., Batta, D., Tabak, A., Korosi, B., Cseprekal, O., Egresits, J. Tisler, A. | Integrated central blood pressure-aortic stiffness risk categories and cardiovascular mortality in endstage renal disease | 2019 | CVD mortality not stratified by sex |
| 20 | Petreski, T., Ekart, R., Hojs, R., Bevc, S. | Asymptomatic hyperuricemia and cardiovascular mortality in patients with chronic kidney disease who progress to hemodialysis | 2019 | CVD mortality not stratified by sex |
| 21 | Sarafidis, P. A.,Loutradis, C., Mayer, C. C., Karpetas, A., Pagkopoulou, E. Bikos, A., Faitatzidou, D., Wassertheurer, S., Schmaderer, C., Liakopoulos, V., Papagianni, A., London, G. | Weak within-individual association of blood pressure and pulse wave velocity in hemodialysis is related to adverse outcomes | 2019 | CVD mortality not stratified by sex |
| 22 | See, E. J., Hedley, J., Agar, J. W. M., Hawley, C. M., Johnson, D. W., Kelly, P. J., Lee, V. W., Mac, K. Polkinghorne, K. R., Rabindranath, K. S., Sud, K., Webster, A. C. | Patient survival on haemodiafiltration and haemodialysis: a cohort study using the Australia and New Zealand Dialysis and Transplant Registry | 2019 | CVD mortality not stratified by sex |
| 23 | Toida, T., Sato, Y., Komatsu, H., Kitamura, K., Fujimoto, S. | Pre- and Postdialysis Uric Acid Difference and Risk of Long-Term All-Cause and Cardiovascular Mortalities in Japanese Hemodialysis Patients; Miyazaki Dialysis Cohort Study | 2019 | CVD mortality not stratified by sex |
| 24 | Wang, Z., Yu, D., Cai, Y., Zhao, B., Zhang, X., Zhao, Z. | Optimal cut-off threshold in pulse pressure predicting cardiovascular death among newly diagnosed end-stage renal disease patients: A prospective cohort study | 2019 | CVD mortality not stratified by sex |
| 25 | Wu, L., Cai, K., Luo, Q., Wang, L. Hong, Y. | Baseline Serum Magnesium Level and Its Variability in Maintenance Hemodialysis Patients: Associations with Mortality | 2019 | CVD mortality not stratified by sex |
| 26 | Wu, M., Wu, H., Huang, X., Ye, H. Huang, F., Yu, X. Yang, X. | Associations between serum mineral metabolism parameters and mortality in patients on peritoneal dialysis | 2019 | CVD mortality not stratified by sex |
| 27 | Wu, P. P., Hsieh, Y. P., Kor, C. T., Chiu, P. F. | Association between albumin-globulin ratio and mortality in patients with chronic kidney disease | 2019 | CVD mortality not stratified by sex |
| 28 | Yan, Z., Yu, D., Cai, Y., Shang, J., Qin, R., Xiao, J., Zhao, B., Zhao, Z., Simmons, D. | Triglyceride Glucose Index Predicting Cardiovascular Mortality in Chinese Initiating Peritoneal Dialysis: A Cohort Study | 2019 | CVD mortality not stratified by sex |
| 29 | Lee, Y. J., Okuda, Y., Sy, J., Lee, Y. K., Obi, Y., Cho, S., Chen, J. L. T., Jin, A., Rhee, C. M., Kalantar-Zadeh, K., Streja, E. | Ultrafiltration Rate, Residual Kidney Function, and Survival Among Patients Treated With Reduced-Frequency Hemodialysis | 2019 | CVD mortality not stratified by sex |
| 30 | Lin, T., Xia, X., Yu, J., Qiu, Y., Yi, C., Lin, J., Mao, H., Yang, X., Huang, F. | The predictive study of the relation between elevated low-density lipoprotein cholesterol to high-density lipoprotein cholesterol ratio and mortality in peritoneal dialysis | 2019 | CVD mortality not stratified by sex |
| 31 | Peng, F., Sun, L., Chen, T., Zhu, Y., Zhou, W., Li, P., Chen, Y., Zhuang, Y., Huang, Q., Long, H. | Albumin-globulin ratio and mortality in patients on peritoneal dialysis: A retrospective study | 2019 | CVD mortality not stratified by sex |
| 32 | Ren, M., Sheng, Q., Xie, X., Zhang, X., Han, F., Chen, J. | Geriatric nutritional risk index is associated with mortality in peritoneal dialysis patients | 2019 | CVD mortality not stratified by sex |
| 33 | Saglimbene, V. M., Wong, G., Teixeira-Pinto, A., Ruospo, M., Garcia-Larsen, V., Palmer, S. C. Natale, P., Campbell, K., Carrero, J. J., Stenvinkel, P. Gargano, L., Murgo, A. M., Johnson, D. W., Tonelli, M. Gelfman, R., Celia, E., Ecder, T., Bernat, A. G., Del Castillo, D., Timofte, D., Torok, M., Bednarek-Skublewska, A., Dulawa, J., Stroumza, P., Hansis, M., Fabricius, E., Felaco, P., Wollheim, C., Hegbrant, J., Craig, J. C., Strippoli, G. F. M. | Dietary Patterns and Mortality in a Multinational Cohort of Adults Receiving Hemodialysis | 2019 | CVD mortality not stratified by sex |
| 34 | Su, W. Y., Wu, P. Y., Huang, J. C., Chen, S. C., Chang, J. M. | Increased proteinuria is associated with increased aortic arch calcification, cardio-thoracic ratio, rapid renal progression and increased overall and cardiovascular mortality in chronic kidney disease | 2019 | CVD mortality not stratified by sex |
| 35 | Thang, L. V., Loc, N. D., Kien, N. T. Dung, N. H., Quyen, D. B. Q., Tuan, N. M., Ha, D. M., Kien, T. Q., Dung, N. T. T., Van, D. T., Van Duc, N., Ha, N. T. T., Toan, P. Q., Nghia, V. X. | Interleukin 6 is a better predictor of 5-year cardiovascular mortality than high-sensitivity C-reactive protein in hemodialysis patients using reused low-flux dialyzers | 2019 | CVD mortality not stratified by sex |
| 36 | Tsai, C. H., Lin, L. Y., Lin, Y. H., Tsai, I. J., Huang, J. W. | Abdominal aorta calcification predicts cardiovascular but not non-cardiovascular outcome in patients receiving peritoneal dialysis: A prospective cohort study | 2019 | CVD mortality not stratified by sex |
| 37 | Tsuruya, K., Kanda, E., Nomura, T., Iseki, K., Hirakata, H. | Postdialysis blood pressure is a better predictor of mortality than predialysis blood pressure in Japanese hemodialysis patients: the Japan Dialysis Outcomes and Practice Patterns Study | 2019 | CVD mortality not stratified by sex |
| 38 | Wen, Y., Zhan, X., Wang, N., Peng, F., Feng, X., Wu, X. | Monocyte/Lymphocyte Ratio and Cardiovascular Disease Mortality in Peritoneal Dialysis Patients | 2019 | CVD mortality not stratified by sex |
| 39 | Yu, J., Lin, T., Huang, N., Xia, X., Li, J., Qiu, Y., Yang, X., Mao, H., Huang, F. | Plasma fibrinogen and mortality in patients undergoing peritoneal dialysis: A prospective cohort study | 2019 | CVD mortality not stratified by sex |
| 40 | Zeng, M., Liu, Y., Liu, F., Peng, Y., Sun, L., Xiao, L. | J-shaped association of platelet-to-lymphocyte ratio with 5-year mortality among patients with chronic kidney disease in a prospective cohort study | 2019 | CVD mortality not stratified by sex |
| 41 | Zeng, M., Liu, Y., Liu, F., Peng, Y., Sun, L., Xiao, L. | Association between albumin-to-globulin ratio and long-term mortality in patients with chronic kidney disease: a cohort study | 2019 | CVD mortality not stratified by sex |
| 42 | Zeng, Y., Chen, Z., Chen, Q., Zhan, X., Long, H., Peng, F., Zhang, F., Feng, X., Zhou, Q., Liu, L., Peng, X., Evergreen Tree Nephrology, Association, Guo, G., Zhang, Y., Wang, Z., Wen, Y., Li, J. Liang, J. | Neutrophil to Lymphocyte Ratio Predicts Adverse Cardiovascular Outcome in Peritoneal Dialysis Patients Younger than 60 Years Old | 2019 | CVD mortality not stratified by sex |
| 43 | Junrong Tong , Manyi Liu, Hong Li, Zhengmao Luo, Xianyang Zhong, Jun Huang, Riguang Liu, Feng He, Junzhou Fu | Mortality and associated risk factors in dialysis patients with cardiovascular disease | 2016 | CVD mortality not stratified by sex |
| 44 | Aalten, J., Hoogeveen, E. K., Roodnat, J. I., Weimar, W., Borm, G. F., De Fijter, J. W. and Hoitsma, A. J. | Associations between pre-kidney-transplant risk factors and post-transplant cardiovascular events and death | 2008 | CVD mortality not stratified by sex |
| 45 | Abbott, K. C., Trespalacios, F. C. and Agodoa, L. Y. | Arteriovenous fistula use and heart disease in long-term elderly hemodialysis patients: analysis of United States Renal Data System Dialysis Morbidity and Mortality Wave II | 2003 | CVD mortality not stratified by sex |
| 46 | Abbott, K. C., Trespalacios, F. C., Agodoa, L. Y., Taylor, A. J. and Bakris, G. L. | beta-Blocker use in long-term dialysis patients: association with hospitalized heart failure and mortality | 2004 | CVD mortality not stratified by sex |
| 47 | Abdallah, E., Waked, E., Nabil, M. and El-Bendary, O. | Adiponectin and cardiovascular outcomes among hemodialysis patients | 2012 | CVD mortality not stratified by sex |
| 48 | Abedini, S., Meinitzer, A., Holme, I., Marz, W., Weihrauch, G., Fellstrom, B., Jardine, A. and Holdaas, H. | Asymmetrical dimethylarginine is associated with renal and cardiovascular outcomes and all-cause mortality in renal transplant recipients | 2010 | CVD mortality not stratified by sex |
| 49 | Abuzeid, W., Iwanochko, R. M., Wang, X., Kim, S. J., Husain, M. and Lee, D. S. | Prognostic impact of SPECT-MPI after renal transplantation | 2017 | CVD mortality not stratified by sex |
| 50 | Adragao, T., Pires, A., Branco, P., Castro, R., Oliveira, A., Nogueira, C., Bordalo, J., Curto, J. D. and Prata, M. M. | Ankle-brachial index, vascular calcifications and mortality in dialysis patients | 2012 | CVD mortality not stratified by sex |
| 51 | Afkarian, M., Katz, R., Bansal, N., Correa, A., Kestenbaum, B., Himmelfarb, J., de Boer, I. H. and Young, B. | Diabetes, Kidney Disease, and Cardiovascular Outcomes in the Jackson Heart Study | 2016 | CVD mortality not stratified by sex |
| 52 | Afshinnia, F., Ayazi, P. and Chadow, H. L. | Glomerular filtration rate on admission independently predicts short-term in-hospital mortality after acute myocardial infarction | 2006 | CVD mortality not stratified by sex |
| 53 | Agarwal, R. | Blood pressure and mortality among hemodialysis patients | 2010 | CVD mortality not stratified by sex |
| 54 | Agarwal, R. | Ambulatory blood pressure monitoring trumps estimated glomerular filtration rate in predicting cardiovascular risk in low-risk populations | 2013 | CVD mortality not stratified by sex |
| 55 | Agrinier, N., Thilly, N., Briancon, S., Juilliere, Y., Mertes, P. M., Villemot, J. P., Alla, F. and Zannad, F. | Prognostic factors associated with 15-year mortality in patients with hospitalized systolic HF: Results of the observational community-based EPICAL cohort study | 2017 | CVD mortality not stratified by sex |
| 56 | Ahmed, A., Rich, M. W., Sanders, P. W., Perry, G. J., Bakris, G. L., Zile, M. R., Love, T. E., Aban, I. B. and Shlipak, M. G. | Chronic kidney disease associated mortality in diastolic versus systolic heart failure: a propensity matched study | 2007 | CVD mortality not stratified by sex |
| 57 | Ahmed, S., Cannon, C. P., Giugliano, R. P., Murphy, S. A., Morrow, D. A., Antman, E. M., Braunwald, E. and Gibson, C. M. | The independent and combined risk of diabetes and non-endstage renal impairment in non-ST-segment elevation acute coronary syndromes | 2008 | CVD mortality not stratified by sex |
| 58 | Airy, M., Schold, J. D., Jolly, S. E., Arrigain, S., Bansal, N., Winkelmayer, W. C., Nally, J. V. and Navaneethan, S. D. | Cause-Specific Mortality in Patients with Chronic Kidney Disease and Atrial Fibrillation | 2018 | CVD mortality not stratified by sex |
| 59 | Akashi, N., Sakakura, K., Watanabe, Y., Noguchi, M., Taniguchi, Y., Yamamoto, K., Wada, H., Momomura, S. I. and Fujita, H. | The comparison of clinical outcomes in patients with acute myocardial infarction and advanced chronic kidney disease on chronic hemodialysis versus off hemodialysis | 2018 | CVD mortality not stratified by sex |
| 60 | Akdag, I., Yilmaz, Y., Kahvecioglu, S., Bolca, N., Ercan, I., Ersoy, A. and Gullulu, M. | Clinical value of the malnutrition-inflammation-atherosclerosis syndrome for long-term prediction of cardiovascular mortality in patients with end-stage renal disease: a 5-year prospective study | 2008 | CVD mortality not stratified by sex |
| 61 | Aker, S., Bantis, C., Reis, P., Kuhr, N., Schwandt, C., Grabensee, B., Heering, P. and Ivens, K. | Influence of interleukin-6 G-174C gene polymorphism on coronary artery disease, cardiovascular complications and mortality in dialysis patients | 2009 | CVD mortality not stratified by sex |
| 62 | Alam, A., Palumbo, A., Mucsi, I., Barre, P. E. and Sniderman, A. D. | Elevated troponin I levels but not low grade chronic inflammation is associated with cardiac-specific mortality in stable hemodialysis patients | 2013 | CVD mortality not stratified by sex |
| 63 | Alderson, H. V., Ritchie, J. P., Middleton, R., Larsson, A., Larsson, T. E. and Kalra, P. A. | FGF-23 and Osteoprotegerin but not Fetuin-A are associated with death and enhance risk prediction in non-dialysis chronic kidney disease stages 3-5 | 2016 | CVD mortality not stratified by sex |
| 64 | Alderson, H. V., Ritchie, J. P., Pagano, S., Middleton, R. J., Pruijm, M., Vuilleumier, N. and Kalra, P. A. | The Associations of Blood Kidney Injury Molecule-1 and Neutrophil Gelatinase-Associated Lipocalin with Progression from CKD to ESRD | 2016 | CVD mortality not stratified by sex |
| 65 | AlJaroudi, W., Campagnoli, T., Fughhi, I., Wassouf, M., Ali, A. and Doukky, R. | Prognostic value of heart rate response during regadenoson stress myocardial perfusion imaging in patients with end stage renal disease | 2016 | CVD mortality not stratified by sex |
| 66 | Almeida, F. A. A., Machado, F. C., Moura Jr, J. A. and Guimarães, A. C. | Global and cardiovascular mortality and risk factors in patients under hemodialysis treatment | 2010 | CVD mortality not stratified by sex |
| 67 | Amabile, N., Guerin, A. P., Tedgui, A., Boulanger, C. M. and London, G. M. | Predictive value of circulating endothelial microparticles for cardiovascular mortality in end-stage renal failure: a pilot study | 2012 | CVD mortality not stratified by sex |
| 68 | Amaral, T. L. M., Amaral, C. A., Miranda Filho, A. L. and Monteiro, G. T. R. | Trends and multiple causes of death due to chronic renal failure in a municipality in the Brazilian Amazon | 2018 | CVD mortality not stratified by sex |
| 69 | An, W. S. and Son, Y. K. | Vascular calcification on plain radiographs is associated with carotid intima media thickness, malnutrition and cardiovascular events in dialysis patients: A prospective observational study | 2013 | CVD mortality not stratified by sex |
| 70 | Anaya, P., Blomquist, G. A., Davenport, D. L., Monier-Faugere, M. C., Sorrell, V. L. and Malluche, H. H. | Coronary artery calcification in CKD-5D patients is tied to adverse cardiac function and increased mortality | 2016 | CVD mortality not stratified by sex |
| 71 | Ani, C. and Ovbiagele, B. | Relation of baseline presence and severity of renal disease to long-term mortality in persons with known stroke | 2010 | CVD mortality not stratified by sex |
| 72 | Antonelou, M. H., Georgatzakou, H. T., Tzounakas, V. L., Velentzas, A. D., Kokkalis, A. C., Kriebardis, A. G. and Papassideri, I. S. | Blood modifications associated with end stage renal disease duration, progression and cardiovascular mortality: a 3-year follow-up pilot study | 2014 | CVD mortality not stratified by sex |
| 73 | Arase, H., Yamada, S., Yotsueda, R., Taniguchi, M., Yoshida, H., Tokumoto, M., Nakano, T., Tsuruya, K. and Kitazono, T. | Modified creatinine index and risk for cardiovascular events and all-cause mortality in patients undergoing hemodialysis: The Q-Cohort study | 2018 | CVD mortality not stratified by sex |
| 74 | Arikan, H., Koc, M., Tuglular, S., Ozener, C. and Akoglu, E. | Elevated plasma levels of PAI-1 predict cardiovascular events and cardiovascular mortality in prevalent peritoneal dialysis patients | 2009 | CVD mortality not stratified by sex |
| 75 | Aronson, D. and Burger, A. J. | The relationship between transient and persistent worsening renal function and mortality in patients with acute decompensated heart failure | 2010 | CVD mortality not stratified by sex |
| 76 | Artunc, F., Nowak, A., Mueller, C., Breidthardt, T., Twerenbold, R., Wagner, R., Peter, A., Haering, H. U., Ebmeyer, S. and Friedrich, B. | Plasma concentrations of the vasoactive peptide fragments mid-regional pro-adrenomedullin, C-terminal pro-endothelin 1 and copeptin in hemodialysis patients: Associated factors and prediction of mortality | 2014 | CVD mortality not stratified by sex |
| 77 | Astor, B. C., Hallan, S. I., Miller, E. R., 3rd, Yeung, E. and Coresh, J. | Glomerular filtration rate, albuminuria, and risk of cardiovascular and all-cause mortality in the US population | 2008 | CVD mortality not stratified by sex |
| 78 | Astor, B. C., Shafi, T., Hoogeveen, R. C., Matsushita, K., Ballantyne, C. M., Inker, L. A. and Coresh, J. | Novel markers of kidney function as predictors of ESRD, cardiovascular disease, and mortality in the general population | 2012 | CVD mortality not stratified by sex |
| 79 | Astor, B. C., Yi, S., Hiremath, L., Corbin, T., Pogue, V., Wilkening, B., Peterson, G., Lewis, J., Lash, J. P., Van Lente, F., Gassman, J., Wang, X., Bakris, G., Appel, L. J. and Contreras, G. | N-terminal prohormone brain natriuretic peptide as a predictor of cardiovascular disease and mortality in blacks with hypertensive kidney disease: the African American Study of Kidney Disease and Hypertension (AASK) | 2008 | CVD mortality not stratified by sex |
| 80 | Astrup, A. S., Tarnow, L., Rossing, P., Pietraszek, L., Riis Hansen, P. and Parving, H. H. | Improved prognosis in type 1 diabetic patients with nephropathy: a prospective follow-up study | 2005 | CVD mortality not stratified by sex |
| 81 | Ates, K., Nergizoglu, G., Keven, K., Sen, A., Kutlay, S., Erturk, S., Duman, N., Karatan, O. and Ertug, A. E. | Effect of fluid and sodium removal on mortality in peritoneal dialysis patients | 2001 | CVD mortality not stratified by sex |
| 82 | Atkinson, P., Chiu, D. Y., Sharma, R., Kalra, P. R., Ward, C., Foley, R. N., Venning, M. C., Waldek, S., O'Donoghue, D. J. and Kalra, P. A. | Predictive value of myocardial and coronary imaging in the long-term outcome of potential renal transplant recipients | 2011 | CVD mortality not stratified by sex |
| 83 | Avramovski, P., Avramovska, M. and Sikole, A. | B-flow imaging estimation of carotid and femoral atherosclerotic plaques: vessel walls rheological damage or strong predictor of cardiovascular mortality in chronic dialysis patients | 2016 | CVD mortality not stratified by sex |
| 84 | Awan, A. A., Niu, J., Pan, J. S., Erickson, K. F., Mandayam, S., Winkelmayer, W. C., Navaneethan, S. D. and Ramanathan, V. | Trends in the causes of death among kidney transplant recipients in the United States (1996-2014) | 2018 | CVD mortality not stratified by sex |
| 85 | Ayer, A., Mills, C., Donovan, C., Christenson, R. H., Ganz, P. and Dubin, R. F. | Associations of microvascular dysfunction with cardiovascular outcomes: The cardiac, endothelial function and arterial stiffness in ESRD (CERES) cohort | 2019 | CVD mortality not stratified by sex |
| 86 | Baber, U., Stone, G. W., Weisz, G., Moreno, P., Dangas, G., Maehara, A., Mintz, G. S., Cristea, E., Fahy, M., Xu, K., Lansky, A. J., Wennerblom, B., Mathey, D. G., Templin, B., Zhang, Z., Serruys, P. W. and Mehran, R. | Coronary plaque composition, morphology, and outcomes in patients with and without chronic kidney disease presenting with acute coronary syndromes | 2012 | CVD mortality not stratified by sex |
| 87 | Backholer, K., Hirakawa, Y., Tonkin, A., Giles, G., Magliano, D. J., Colagiuri, S., Harris, M., Mitchell, P., Nelson, M., Shaw, J. E., Simmons, D., Simons, L., Taylor, A., Harding, J., Gopinath, B. and Woodward, M. | Development of an Australian cardiovascular disease mortality risk score using multiple imputation and recalibration from national statistics | 2017 | CVD mortality not stratified by sex |
| 88 | Badiou, S., Cristol, J. P., Jaussent, I., Terrier, N., Morena, M., Maurice, F., Leray-Moragues, H., Rivory, J. P., Chalabi, L., Delcourt, C., Canaud, B. and Dupuy, A. M. | Fine-tuning of the prediction of mortality in hemodialysis patients by use of cytokine proteomic determination | 2008 | CVD mortality not stratified by sex |
| 89 | Baek, S. D., Baek, C. H., Kim, J. S., Kim, S. M., Kim, J. H. and Kim, S. B. | Does stage III chronic kidney disease always progress to end-stage renal disease? A ten-year follow-up study | 2012 | CVD mortality not stratified by sex |
| 90 | Bagheri, N., Taziki, O. and Falaknazi, K. | C- Reactive protein, cardiac troponin T and low albumin are predictors of mortality in hemodialysis patients | 2009 | CVD mortality not stratified by sex |
| 91 | Bai, K., Pan, Y., Lu, F., Zhao, Y., Wang, F. and Zhang, L. | Cognitive function and 3-year mortality in the very elderly Chinese population with chronic kidney disease | 2018 | CVD mortality not stratified by sex |
| 92 | Bajaj, A., Damrauer, S. M., Anderson, A. H., Xie, D., Budoff, M. J., Go, A. S., He, J., Lash, J. P., Ojo, A., Post, W. S., Rahman, M., Reilly, M. P., Saleheen, D., Townsend, R. R., Chen, J. and Rader, D. J. | Lipoprotein(a) and Risk of Myocardial Infarction and Death in Chronic Kidney Disease: Findings From the CRIC Study (Chronic Renal Insufficiency Cohort) | 2017 | CVD mortality not stratified by sex |
| 93 | Banerjee, A., Fauchier, L., Vourc'h, P., Andres, C. R., Taillandier, S., Halimi, J. M. and Lip, G. Y. H. | A prospective study of estimated glomerular filtration rate and outcomes in patients with atrial fibrillation: the Loire Valley Atrial Fibrillation Project | 2014 | CVD mortality not stratified by sex |
| 94 | Bansal, N., McCulloch, C. E., Rahman, M., Kusek, J. W., Anderson, A. H., Xie, D., Townsend, R. R., Lora, C. M., Wright, J., Go, A. S. and et al. | Blood pressure and risk of all-cause mortality in advanced chronic kidney disease and hemodialysis the chronic renal insufficiency cohort study | 2015 | CVD mortality not stratified by sex |
| 95 | Bao, W., Wang, F. and Tang, W. | Aortic-brachial stiffness mismatch and mortality in peritoneal dialysis patients | 2019 | CVD mortality not stratified by sex |
| 96 | Bargnoux, A. S., Morena, M., Jaussent, I., Maurice, F., Chalabi, L., Leray-Moragues, H., Terrier, N., Dupuy, A. M., Badiou, S., Canaud, B. and Cristol, J. P. | A combined index of cardiac biomarkers as a risk factor for early cardiovascular mortality in hemodialysis patients | 2013 | CVD mortality not stratified by sex |
| 97 | Barr, E. L., Reutens, A., Magliano, D. J., Wolfe, R., Lu, Z. X., Sikaris, K. A., Tanamas, S. K., Atkins, R., Chadban, S., Shaw, J. E. and Polkinghorne, K. R. | Cystatin C estimated glomerular filtration rate and all-cause and cardiovascular disease mortality risk in the general population: AusDiab study | 2017 | CVD mortality not stratified by sex |
| 98 | Barreto, D. V., Barreto, F. C., Liabeuf, S., Temmar, M., Lemke, H. D., Tribouilloy, C., Choukroun, G., Vanholder, R. and Massy, Z. A. | Plasma interleukin-6 is independently associated with mortality in both hemodialysis and pre-dialysis patients with chronic kidney disease | 2010 | CVD mortality not stratified by sex |
| 99 | Barreto, F. C., Barreto, D. V., Liabeuf, S., Meert, N., Glorieux, G., Temmar, M., Choukroun, G., Vanholder, R. and Massy, Z. A. | Serum indoxyl sulfate is associated with vascular disease and mortality in chronic kidney disease patients | 2009 | CVD mortality not stratified by sex |
| 100 | Baumann, M., Wassertheurer, S., Suttmann, Y., Burkhardt, K. and Heemann, U. | Aortic pulse wave velocity predicts mortality in chronic kidney disease stages 2-4 | 2014 | CVD mortality not stratified by sex |
| 101 | Bavanandan, S., Ajayi, S., Fentum, B., Paul, S. K., Carr, S. J. and Robinson, T. G. | Cardiac baroreceptor sensitivity: a prognostic marker in predialysis chronic kidney disease patients? | 2005 | CVD mortality not stratified by sex |
| 102 | Beaubien, E. R., Pylypchuk, G. B., Akhtar, J. and Biem, H. J. | Value of corrected QT interval dispersion in identifying patients initiating dialysis at increased risk of total and cardiovascular mortality | 2002 | CVD mortality not stratified by sex |
| 103 | Beberashvili, I., Katkov, A., Sinuani, I., Azar, A., Shapiro, G., Feldman, L., Gorelik, O., Stav, K. and Efrati, S. | Serum Obestatin: A Biomarker of Cardiovascular and All-Cause Mortality in Hemodialysis Patients | 2018 | CVD mortality not stratified by sex |
| 104 | Beberashvili, I., Sinuani, I., Azar, A., Kadoshi, H., Shapiro, G., Feldman, L., Sandbank, J. and Averbukh, Z. | Increased basal nitric oxide amplifies the association of inflammation with all-cause and cardiovascular mortality in prevalent hemodialysis patients | 2013 | CVD mortality not stratified by sex |
| 105 | Beberashvili, I., Sinuani, I., Azar, A., Kadoshi, H., Shapiro, G., Feldman, L., Sandbank, J. and Averbukh, Z. | Decreased IGF-1 levels potentiate association of inflammation with all-cause and cardiovascular mortality in prevalent hemodialysis patients | 2013 | CVD mortality not stratified by sex |
| 106 | Beberashvili, I., Sinuani, I., Azar, A., Kadoshi, H., Shapiro, G., Feldman, L., Sandbank, J. and Averbukh, Z. | Low serum concentration of obestatin as a predictor of mortality in maintenance hemodialysis patients | 2013 | CVD mortality not stratified by sex |
| 107 | Beberashvili, I., Sinuani, I., Azar, A., Shapiro, G., Feldman, L., Doenyas-Barak, K., Stav, K. and Efrati, S. | Interaction between acyl-ghrelin and BMI predicts clinical outcomes in hemodialysis patients | 2017 | CVD mortality not stratified by sex |
| 108 | Beddhu, S., Allen-Brady, K., Cheung, A. K., Horne, B. D., Bair, T., Muhlestein, J. B. and Anderson, J. L. | Impact of renal failure on the risk of myocardial infarction and death | 2002 | CVD mortality not stratified by sex |
| 109 | Beddhu, S., Nigwekar, S. U., Ma, X. and Greene, T. | Associations of resting heart rate with insulin resistance, cardiovascular events and mortality in chronic kidney disease | 2009 | CVD mortality not stratified by sex |
| 110 | Bellizzi, V., Chiodini, P., Cupisti, A., Viola, B. F., Pezzotta, M., De Nicola, L., Minutolo, R., Barsotti, G., Piccoli, G. B. and Di Iorio, B. | Very low-protein diet plus ketoacids in chronic kidney disease and risk of death during end-stage renal disease: a historical cohort controlled study | 2015 | CVD mortality not stratified by sex |
| 111 | Bevc, S., Purg, D., Knehtl, M., Hren, M., Turnšek, N., Hojs, N., Zorman, T., Dvoršak, B., Ekart, R. and Hojs, R. | Ankle-Brachial Index and Long-Term (10 Years) Survival of Nondiabetic Hemodialysis Patients | 2016 | CVD mortality not stratified by sex |
| 112 | Bevc, S., Purg, D., Turnšek, N., Hren, M., Hojs, N., Zorman, T., Pečovnik-Balon, B., Dvoršak, B., Ekart, R. and Hojs, R. | Ankle-Brachial index and cardiovascular mortality in nondiabetic hemodialysis patients | 2013 | CVD mortality not stratified by sex |
| 113 | Bhatti, S., Hakeem, A., Dhanalakota, S., Palani, G., Husain, Z., Jacobsen, G. and Ananthasubramaniam, K. | Prognostic value of regadenoson myocardial single-photon emission computed tomography in patients with different degrees of renal dysfunction | 2014 | CVD mortality not stratified by sex |
| 114 | Bian, X., Liu, N., Bai, Y., Zheng, L., He, P., Su, X., Du, F., Yang, X. and Li, D. | Association of leptin with mortality in patients on maintenance hemodialysis: a prospective study | 2014 | CVD mortality not stratified by sex |
| 115 | Biesenbach, G., Loipl, J., Schmekal, B. and Janko, O. | Different risk factors and causes for early death after initiating dialysis in diabetic and non-diabetic patients | 2007 | CVD mortality not stratified by sex |
| 116 | Bittencourt, M. S., Hulten, E. A., Ghoshhajra, B., Abbara, S., Murthy, V. L., Divakaran, S., Nasir, K., Gowdak, L. H., Riella, L. V., Chiumiento, M., Hoffmann, U., Di Carli, M. F. and Blankstein, R. | Incremental prognostic value of kidney function decline over coronary artery disease for cardiovascular event prediction after coronary computed tomography | 2015 | CVD mortality not stratified by sex |
| 117 | Blacher, J., Safar, M. E., Guerin, A. P., Pannier, B., Marchais, S. J. and London, G. M. | Aortic pulse wave velocity index and mortality in end-stage renal disease | 2003 | CVD mortality not stratified by sex |
| 118 | Block, G. A., Klassen, P. S., Lazarus, J. M., Ofsthun, N., Lowrie, E. G. and Chertow, G. M. | Mineral metabolism, mortality, and morbidity in maintenance hemodialysis | 2004 | CVD mortality not stratified by sex |
| 119 | Boger, C. A., Gotz, A., Stubanus, M., Banas, B., Deinzer, M., Kruger, B., Holmer, S. R., Schmitz, G., Riegger, G. A. and Kramer, B. K. | C-reactive protein as predictor of death in end-stage diabetic nephropathy: role of peripheral arterial disease | 2005 | CVD mortality not stratified by sex |
| 120 | Bolignano, D., Lennartz, S., Leonardis, D., D'Arrigo, G., Tripepi, R., Emrich, I. E., Mallamaci, F., Fliser, D., Heine, G. and Zoccali, C. | High estimated pulmonary artery systolic pressure predicts adverse cardiovascular outcomes in stage 2-4 chronic kidney disease | 2015 | CVD mortality not stratified by sex |
| 121 | Bonato, F. O., Watanabe, R., Lemos, M. M., Cassiolato, J. L., Wolf, M. and Canziani, M. E. | Asymptomatic Ventricular Arrhythmia and Clinical Outcomes in Chronic Kidney Disease: A Pilot Study | 2016 | CVD mortality not stratified by sex |
| 122 | Boriani, G., Laroche, C., Diemberger, I., Fantecchi, E., Popescu, M. I., Rasmussen, L. H., Sinagra, G., Petrescu, L., Tavazzi, L., Maggioni, A. P. and Lip, G. Y. | Asymptomatic atrial fibrillation: clinical correlates, management, and outcomes in the EORP-AF Pilot General Registry | 2015 | CVD mortality not stratified by sex |
| 123 | Bostom, A. G., Carpenter, M. A., Kusek, J. W., Levey, A. S., Hunsicker, L., Pfeffer, M. A., Selhub, J., Jacques, P. F., Cole, E., Gravens-Mueller, L. and et al. | Homocysteine-lowering and cardiovascular disease outcomes in kidney transplant recipients: primary results from the Folic Acid for Vascular Outcome Reduction in Transplantation trial | 2011 | CVD mortality not stratified by sex |
| 124 | Bozic, M., Mendez-Barbero, N., Gutierrez-Munoz, C., Betriu, A., Egido, J., Fernandez, E., Martin-Ventura, J. L., Valdivielso, J. M. and Blanco-Colio, L. M. | Combination of biomarkers of vascular calcification and sTWEAK to predict cardiovascular events in chronic kidney disease | 2018 | CVD mortality not stratified by sex |
| 125 | Brandenburg, V. M., Schlieper, G., Heussen, N., Holzmann, S., Busch, B., Evenepoel, P., Vanholder, R., Meijers, B., Meert, N., Fassbender, W. J., Floege, J., Jahnen-Dechent, W. and Ketteler, M. | Serological cardiovascular and mortality risk predictors in dialysis patients receiving sevelamer: a prospective study | 2010 | CVD mortality not stratified by sex |
| 126 | Brantsma, A. H., Bakker, S. J., Hillege, H. L., de Zeeuw, D., de Jong, P. E. and Gansevoort, R. T. | Cardiovascular and renal outcome in subjects with K/DOQI stage 1-3 chronic kidney disease: the importance of urinary albumin excretion | 2008 | CVD mortality not stratified by sex |
| 127 | Bray, B. D., Boyd, J., Daly, C., Donaldson, K., Doyle, A., Fox, J. G., Innes, A., Khan, I., Peel, R. K., Severn, A., Shilliday, I., Simpson, K., Stewart, G. A., Traynor, J. and Metcalfe, W. | Vascular access type and risk of mortality in a national prospective cohort of haemodialysis patients | 2012 | CVD mortality not stratified by sex |
| 128 | Brenner, B. M., Cooper, M. E., de Zeeuw, D., Keane, W. F., Mitch, W. E., Parving, H. H., Remuzzi, G., Snapinn, S. M., Zhang, Z. and Shahinfar, S. | Effects of losartan on renal and cardiovascular outcomes in patients with type 2 diabetes and nephropathy | 2001 | CVD mortality not stratified by sex |
| 129 | Bruch, C., Fischer, C., Sindermann, J., Stypmann, J., Breithardt, G. and Gradaus, R. | Comparison of the prognostic usefulness of N-terminal pro-brain natriuretic Peptide in patients with heart failure with versus without chronic kidney disease | 2008 | CVD mortality not stratified by sex |
| 130 | Bruch, C., Reinecke, H., Rothenburger, M., Scheld, H. H., Whalley, G. A., Stypmann, J., Breithardt, G., Wichter, T. and Gradaus, R. | Transmitral flow patterns and the presence of chronic kidney disease provide independent and incremental prognostic information in patients with heart failure and systolic dysfunction | 2007 | CVD mortality not stratified by sex |
| 131 | Bruch, C., Reinecke, H., Stypmann, J., Rothenburger, M., Schmid, C., Breithardt, G., Wichter, T. and Gradaus, R. | N-terminal pro-brain natriuretic peptide, kidney disease and outcome in patients with chronic heart failure | 2006 | CVD mortality not stratified by sex |
| 132 | Brunelli, S. M., Sibbel, S., Do, T. P., Cooper, K. and Bradbury, B. D. | Facility Dialysate Calcium Practices and Clinical Outcomes Among Patients Receiving Hemodialysis: A Retrospective Observational Study | 2015 | CVD mortality not stratified by sex |
| 133 | Buccianti, G., Baragetti, I., Bamonti, F., Furiani, S., Dorighet, V. and Patrosso, C. | Plasma homocysteine levels and cardiovascular mortality in patients with end-stage renal disease | 2004 | CVD mortality not stratified by sex |
| 134 | Busch, M., Fleck, C., Wolf, G. and Stein, G. | Asymmetrical (ADMA) and symmetrical dimethylarginine (SDMA) as potential risk factors for cardiovascular and renal outcome in chronic kidney disease - possible candidates for paradoxical epidemiology? | 2006 | CVD mortality not stratified by sex |
| 135 | Byungsu Yoo, B. S., Son, J. W., Kim, J. Y., Ahn, M. S., Lee, S. H. and Yoon, J. H. | Gender difference of obesity paradox in systolic heart failure | 2017 | CVD mortality not stratified by sex |
| 136 | Cabrera, C., Brunelli, S. M., Rosenbaum, D., Anum, E., Ramakrishnan, K., Jensen, D. E., Stalhammar, N. O. and Stefansson, B. V. | A retrospective, longitudinal study estimating the association between interdialytic weight gain and cardiovascular events and death in hemodialysis patients | 2015 | CVD mortality not stratified by sex |
| 137 | Cai, K., Luo, Q., Dai, Z., Zhu, B., Fei, J., Xue, C. and Wu, D. | Hypomagnesemia Is Associated with Increased Mortality among Peritoneal Dialysis Patients | 2016 | CVD mortality not stratified by sex |
| 138 | Campbell, R. C., Sui, X., Filippatos, G., Love, T. E., Wahle, C., Sanders, P. W. and Ahmed, A. | Association of chronic kidney disease with outcomes in chronic heart failure: a propensity-matched study | 2009 | CVD mortality not stratified by sex |
| 139 | Cannata-Andia, J. B., Fernandez-Martin, J. L., Locatelli, F., London, G., Gorriz, J. L., Floege, J., Ketteler, M., Ferreira, A., Covic, A., Rutkowski, B., Memmos, D., Bos, W. J., Teplan, V., Nagy, J., Tielemans, C., Verbeelen, D., Goldsmith, D., Kramar, R., Martin, P. Y., Wuthrich, R. P., Pavlovic, D., Benedik, M., Sanchez, J. E., Martinez-Camblor, P., Naves-Diaz, M., Carrero, J. J. and Zoccali, C. | Use of phosphate-binding agents is associated with a lower risk of mortality | 2013 | CVD mortality not stratified by sex |
| 140 | Carrero, J. J., Kyriazis, J., Sonmez, A., Tzanakis, I., Qureshi, A. R., Stenvinkel, P., Saglam, M., Stylianou, K., Yaman, H., Taslipinar, A., Vural, A., Gok, M., Yenicesu, M., Daphnis, E. and Yilmaz, M. I. | Prolactin levels, endothelial dysfunction, and the risk of cardiovascular events and mortality in patients with CKD | 2012 | CVD mortality not stratified by sex |
| 141 | Carrero, J. J., Ortiz, A., Qureshi, A. R., Martin-Ventura, J. L., Barany, P., Heimburger, O., Marron, B., Metry, G., Snaedal, S., Lindholm, B., Egido, J., Stenvinkel, P. and Blanco-Colio, L. M. | Additive effects of soluble TWEAK and inflammation on mortality in hemodialysis patients | 2009 | CVD mortality not stratified by sex |
| 142 | Carrero, J. J., Qureshi, A. R., Axelsson, J., Yilmaz, M. I., Rehnmark, S., Witt, M. R., Barany, P., Heimburger, O., Suliman, M. E., Alvestrand, A., Lindholm, B. and Stenvinkel, P. | Clinical and biochemical implications of low thyroid hormone levels (total and free forms) in euthyroid patients with chronic kidney disease | 2007 | CVD mortality not stratified by sex |
| 143 | Chamberlain, A. M., Alonso, A., Gersh, B. J., Manemann, S. M., Killian, J. M., Weston, S. A., Byrne, M. and Roger, V. L. | Multimorbidity and the risk of hospitalization and death in atrial fibrillation: A population-based study | 2017 | CVD mortality not stratified by sex |
| 144 | Chang, J. F., Hsu, S. P., Pai, M. F., Yang, J. Y., Chen, H. Y., Wu, H. Y. and Peng, Y. S. | High soluble vascular cell adhesion molecule-1 concentrations predict long-term mortality in hemodialysis patients | 2013 | CVD mortality not stratified by sex |
| 145 | Chang, J. M., Chen, S. C., Huang, J. C., Su, H. M. and Chen, H. C. | Anemia and left ventricular hypertrophy with renal function decline and cardiovascular events in chronic kidney disease | 2014 | CVD mortality not stratified by sex |
| 146 | Chang, T. I., Streja, E., Soohoo, M., Kim, T. W., Rhee, C. M., Kovesdy, C. P., Kashyap, M. L., Vaziri, N. D., Kalantar-Zadeh, K. and Moradi, H. | Association of Serum Triglyceride to HDL Cholesterol Ratio with All-Cause and Cardiovascular Mortality in Incident Hemodialysis Patients | 2017 | CVD mortality not stratified by sex |
| 147 | Chang, Y. K., Hsu, C. C., Hwang, S. J., Chen, P. C., Huang, C. C., Li, T. C. and Sung, F. C. | A comparative assessment of survival between propensity score-matched patients with peritoneal dialysis and hemodialysis in Taiwan | 2012 | CVD mortality not stratified by sex |
| 148 | Charytan, D. M., Skali, H., Shah, N. R., Veeranna, V., Cheezum, M. K., Taqueti, V. R., Kato, T., Bibbo, C. R., Hainer, J., Dorbala, S., Blankstein, R. and Di Carli, M. F. | Coronary flow reserve is predictive of the risk of cardiovascular death regardless of chronic kidney disease stage | 2018 | CVD mortality not stratified by sex |
| 149 | Charytan, D. M., Solomon, S. D., Ivanovich, P., Remuzzi, G., Cooper, M. E., McGill, J. B., Parving, H. H., Parfrey, P., Singh, A. K., Burdmann, E. A., Levey, A. S., Eckardt, K. U., McMurray, J. J. V., Weinrauch, L. A., Liu, J., Claggett, B., Lewis, E. F. and Pfeffer, M. A. | Metformin use and cardiovascular events in patients with type 2 diabetes and chronic kidney disease | 2019 | CVD mortality not stratified by sex |
| 150 | Chen, D. Y., Wang, S. H., Mao, C. T., Tsai, M. L., Lin, Y. S., Chou, C. C., Wen, M. S., Wang, C. C., Hsieh, I. C., Hung, K. C. and Chen, T. H. | Sitagliptin and cardiovascular outcomes in diabetic patients with chronic kidney disease and acute myocardial infarction: A nationwide cohort study | 2015 | CVD mortality not stratified by sex |
| 151 | Chen, F. A., Yang, C. Y., Yang, W. C., Chen, J. Y., Ng, Y. Y., Li, S. Y., Liu, W. S., Cheng, S. T., Wang, Y. J. and Lin, C. C. | Ankle-brachial index is a powerful predictor of renal outcome and cardiovascular events in patients with chronic kidney disease | 2012 | CVD mortality not stratified by sex |
| 152 | Chen, H. H., Wu, C. J., Chen, Y. C., Tsai, C. S., Lin, F. J. and Yeh, H. I. | Metabolic syndrome is associated with severe coronary artery disease and poor cardiac outcome in end-stage renal disease patients with acute coronary syndrome | 2006 | CVD mortality not stratified by sex |
| 153 | Chen, H. Y., Chiu, Y. L., Hsu, S. P., Pai, M. F., Yang, J. Y. and Peng, Y. S. | Fetuin A/nutritional status predicts cardiovascular outcomes and survival in hemodialysis patients | 2014 | CVD mortality not stratified by sex |
| 154 | Chen, H. Y., Tsai, W. C., Chiu, Y. L., Hsu, S. P., Pai, M. F., Yang, J. Y. and Peng, Y. S. | Triglyceride to high-density lipoprotein cholesterol ratio predicts cardiovascular outcomes in prevalent dialysis patients | 2015 | CVD mortality not stratified by sex |
| 155 | Chen, H. Y., Wei, F., Wang, L. H., Wang, Z., Meng, J., Yu, H. B., Zhang, R. N., Sun, G. J., Jiang, A. L. and Wang, L. | Abnormal ankle-brachial index and risk of cardiovascular or all-cause mortality in patients with chronic kidney disease: a meta-analysis | 2017 | CVD mortality not stratified by sex |
| 156 | Chen, J. B., Cheng, B. C., Yang, C. H. and Hua, M. S. | An association between time-varying serum albumin level and the mortality rate in maintenance haemodialysis patients: a five-year clinical cohort study | 2016 | CVD mortality not stratified by sex |
| 157 | Chen, L. P., Chiang, C. K., Peng, Y. S., Hsu, S. P., Lin, C. Y., Lai, C. F. and Hung, K. Y. | Relationship between periodontal disease and mortality in patients treated with maintenance hemodialysis | 2011 | CVD mortality not stratified by sex |
| 158 | Chen, P. C., Huang, J. C., Chen, S. C., Wu, P. Y., Lee, J. J., Chiu, Y. W., Chang, J. M., Chen, H. C. and Huang, Y. L. | Association of type 2 diabetes mellitus and ratio of transmitral E wave velocity to early diastole mitral velocity with cardiovascular events in chronic kidney disease | 2017 | CVD mortality not stratified by sex |
| 159 | Chen, R., Kumar, S., Timmis, A., Feder, G., Yaqoob, M. M. and Hemingway, H. | Comparison of the relation between renal impairment, angiographic coronary artery disease, and long-term mortality in women versus men | 2006 | CVD mortality not stratified by sex |
| 160 | Chen, S. C., Chang, J. M., Hwang, S. J., Tsai, J. C., Liu, W. C., Wang, C. S., Lin, T. H., Su, H. M. and Chen, H. C. | Ankle brachial index as a predictor for mortality in patients with chronic kidney disease and undergoing haemodialysis | 2010 | CVD mortality not stratified by sex |
| 161 | Chen, S. C., Chang, J. M., Liu, W. C., Huang, J. C., Tsai, J. C., Lin, M. Y., Su, H. M., Hwang, S. J. and Chen, H. C. | Echocardiographic parameters are independently associated with increased cardiovascular events in patients with chronic kidney disease | 2012 | CVD mortality not stratified by sex |
| 162 | Chen, S. C., Chang, J. M., Tsai, J. C., Hsu, P. C., Lin, T. H., Su, H. M., Voon, W. C., Hwang, S. J. and Chen, H. C. | A new systolic parameter defined as the ratio of brachial pre-ejection period to brachial ejection time predicts overall and cardiovascular mortality in hemodialysis patients | 2010 | CVD mortality not stratified by sex |
| 163 | Chen, S. C., Chang, J. M., Tsai, J. C., Lin, T. H., Hsu, P. C., Su, H. M., Voon, W. C., Hwang, S. J. and Chen, H. C. | A systolic parameter defined as the ratio of brachial pre-ejection period to brachial ejection time predicts cardiovascular events in patients with chronic kidney disease | 2010 | CVD mortality not stratified by sex |
| 164 | Chen, S. C., Chang, J. M., Tsai, Y. C., Huang, J. C., Chen, L. I., Su, H. M., Hwang, S. J. and Chen, H. C. | Ratio of transmitral E-wave velocity to early diastole mitral annulus velocity with cardiovascular and renal outcomes in chronic kidney disease | 2013 | CVD mortality not stratified by sex |
| 165 | Chen, S. C., Chang, J. M., Tsai, Y. C., Tsai, J. C., Su, H. M., Hwang, S. J. and Chen, H. C. | Association of interleg BP difference with overall and cardiovascular mortality in hemodialysis | 2012 | CVD mortality not stratified by sex |
| 166 | Chen, S. C., Huang, J. C., Tsai, Y. C., Chen, L. I., Su, H. M., Chang, J. M. and Chen, H. C. | Body Mass Index, Left Ventricular Mass Index and Cardiovascular Events in Chronic Kidney Disease | 2016 | CVD mortality not stratified by sex |
| 167 | Chen, S. C., Huang, J. C., Tsai, Y. C., Hsiu-Chin Mai, R. N., Jui-Hsin Chen, R. N., Kuo, P. L., Chang, J. M., Hwang, S. J. and Chen, H. C. | Heart Rate Variability Change Before and After Hemodialysis is Associated with Overall and Cardiovascular Mortality in Hemodialysis | 2016 | CVD mortality not stratified by sex |
| 168 | Chen, S. C., Hung, C. C., Tsai, Y. C., Huang, J. C., Kuo, M. C., Lee, J. J., Chiu, Y. W., Chang, J. M., Hwang, S. J. and Chen, H. C. | Association of cholesterol levels with mortality and cardiovascular events among patients with CKD and different amounts of proteinuria | 2013 | CVD mortality not stratified by sex |
| 169 | Chen, S. C., Lee, M. Y., Huang, J. C., Shih, M. C. P., Chang, J. M. and Chen, H. C. | Association of ankle-brachial index and aortic arch calcification with overall and cardiovascular mortality in hemodialysis | 2016 | CVD mortality not stratified by sex |
| 170 | Chen, S. C., Su, H. M., Huang, J. C., Chang, K., Tsai, Y. C., Chen, L. I., Chang, J. M., Hwang, S. J. and Chen, H. C. | Association of P-Wave Dispersion with Overall and Cardiovascular Mortality in Hemodialysis Patients | 2015 | CVD mortality not stratified by sex |
| 171 | Chen, T. K., Appel, L. J., Grams, M. E., Tin, A., Choi, M. J., Lipkowitz, M. S., Winkler, C. A. and Estrella, M. M. | APOL1 Risk Variants and Cardiovascular Disease: Results From the AASK (African American Study of Kidney Disease and Hypertension) | 2017 | CVD mortality not stratified by sex |
| 172 | Chen, Y. H., Hung, S. C. and Tarng, D. C. | Length polymorphism in heme oxygenase-1 and cardiovascular events and mortality in hemodialysis patients | 2013 | CVD mortality not stratified by sex |
| 173 | Cheung, A. K., Sarnak, M. J., Yan, G., Berkoben, M., Heyka, R., Kaufman, A., Lewis, J., Rocco, M., Toto, R., Windus, D. and et al. | Cardiac diseases in maintenance hemodialysis patients: results of the HEMO Study | 2004 | CVD mortality not stratified by sex |
| 174 | Cheung, C. L., Sahni, S., Cheung, B. M., Sing, C. W. and Wong, I. C. | Vitamin K intake and mortality in people with chronic kidney disease from NHANES III | 2015 | CVD mortality not stratified by sex |
| 175 | Chiu, D., Abidin, N., Johnstone, L., Chong, M., Kataria, V., Sewell, J., Sinha, S., Kalra, P. A. and Green, D. | Novel Approach to Cardiovascular Outcome Prediction in Haemodialysis Patients | 2016 | CVD mortality not stratified by sex |
| 176 | Chiu, D. Y., Kalra, P. A., Sinha, S. and Green, D. | Association of serum sodium levels with all-cause and cardiovascular mortality in chronic kidney disease: Results from a prospective observational study | 2016 | CVD mortality not stratified by sex |
| 177 | Choi, C. Y., Park, J. S., Yoon, K. T., Gil, H. W., Lee, E. Y. and Hong, S. Y. | Intra-dialytic hypertension is associated with high mortality in hemodialysis patients | 2017 | CVD mortality not stratified by sex |
| 178 | Choi, J. S., Kim, C. S., Bae, E. H., Ma, S. K., Ahn, Y. K., Jeong, M. H., Kim, Y. J., Cho, M. C., Kim, C. J. and Kim, S. W. | Predicting outcomes after myocardial infarction by using the Chronic Kidney Disease Epidemiology Collaboration equation in comparison with the Modification of Diet in Renal Disease study equation: results from the Korea Acute Myocardial Infarction Registry | 2012 | CVD mortality not stratified by sex |
| 179 | Chonchol, M., Greene, T., Zhang, Y., Hoofnagle, A. N. and Cheung, A. K. | Low Vitamin D and High Fibroblast Growth Factor 23 Serum Levels Associate with Infectious and Cardiac Deaths in the HEMO Study | 2016 | CVD mortality not stratified by sex |
| 180 | Chou, M. T., Wang, J. J., Sun, Y. M., Sheu, M. J., Chu, C. C., Weng, S. F., Chio, C. C., Kan, W. C. and Chien, C. C. | Epidemiology and mortality among dialysis patients with acute coronary syndrome: Taiwan National Cohort Study | 2013 | CVD mortality not stratified by sex |
| 181 | Chowdhury, E. K., Langham, R. G., Ademi, Z., Owen, A., Krum, H., Wing, L. M., Nelson, M. R. and Reid, C. M. | Rate of change in renal function and mortality in elderly treated hypertensive patients | 2015 | CVD mortality not stratified by sex |
| 182 | Chowdhury, M. M., Makris, G. C., Tarkin, J. M., Joshi, F. R., Hayes, P. D., Rudd, J. H. F. and Coughlin, P. A. | Lower limb arterial calcification (LLAC) scores in patients with symptomatic peripheral arterial disease are associated with increased cardiac mortality and morbidity | 2017 | CVD mortality not stratified by sex |
| 183 | Chronic Kidney Disease Prognosis, C., Matsushita, K., van der Velde, M., Astor, B. C., Woodward, M., Levey, A. S., de Jong, P. E., Coresh, J. and Gansevoort, R. T. | Association of estimated glomerular filtration rate and albuminuria with all-cause and cardiovascular mortality in general population cohorts: a collaborative meta-analysis | 2010 | CVD mortality not stratified by sex |
| 184 | Chuang, Y. W., Yu, T. M., Huang, S. T., Sun, K. T., Lo, Y. C., Fu, P. K., Lee, B. J., Chen, C. H., Lin, C. L. and Kao, C. H. | Young-adult polycystic kidney disease is associated with major cardiovascular complications | 2018 | CVD mortality not stratified by sex |
| 185 | Cimbaljevic, S., Suvakov, S., Matic, M., Pljesa-Ercegovac, M., Pekmezovic, T., Radic, T., Coric, V., Damjanovic, T., Dimkovic, N., Markovic, R., Savic-Radojevic, A. and Simic, T. | Association of GSTO1 and GSTO2 polymorphism with risk of end-stage renal disease development and patient survival | 2016 | CVD mortality not stratified by sex |
| 186 | Claes, K. J., Heye, S., Bammens, B., Kuypers, D. R., Meijers, B., Naesens, M., Vanrenterghem, Y. and Evenepoel, P. | Aortic calcifications and arterial stiffness as predictors of cardiovascular events in incident renal transplant recipients | 2013 | CVD mortality not stratified by sex |
| 187 | Cohen, S. D., Kimmel, P. L., Neff, R., Agodoa, L. and Abbott, K. C. | Association of incident gout and mortality in dialysis patients | 2008 | CVD mortality not stratified by sex |
| 188 | Coll, M., Ferrer-Costa, C., Pich, S., Allegue, C., Rodrigo, E., Fernández-Fresnedo, G., Barreda, P., Mates, J., De Francisco, A. L. M., Ortega, I., Iglesias, A., Campuzano, O., Salas, E., Arias, M. and Brugada, R. | Role of genetic and electrolyte abnormalities in prolonged QTc interval and sudden cardiac death in end-stage renal disease patients | 2018 | CVD mortality not stratified by sex |
| 189 | Collado, S., Coll, E., Nicolau, C., Azqueta, M., Pons, M., Cruzado, J. M., de la Torre, B., Deulofeu, R., Mojal, S., Pascual, J. and Cases, A. | Serum osteoprotegerin in prevalent hemodialysis patients: associations with mortality, atherosclerosis and cardiac function | 2017 | CVD mortality not stratified by sex |
| 190 | Cooper, B. A., Branley, P., Bulfone, L., Collins, J. F., Craig, J. C., Fraenkel, M. B., Harris, A., Johnson, D. W., Kesselhut, J., Li, J. J. and et al. | A randomized, controlled trial of early versus late initiation of dialysis | 2010 | CVD mortality not stratified by sex |
| 191 | Cooper, C. J., Murphy, T. P., Cutlip, D. E., D'Agostino, R., Jamerson, K., Matsumoto, A. H., Jaff, M. R., Michael, S., Solomon, S., Cohen, D. and et al. | A randomized multicenter clinical trial of renal artery stenting in preventing cardiovascular and renal events: results of the CORAL study | 2013 | CVD mortality not stratified by sex |
| 192 | Cordeiro, A. C., Amparo, F. C., Oliveira, M. A., Amodeo, C., Smanio, P., Pinto, I. M., Lindholm, B., Stenvinkel, P. and Carrero, J. J. | Epicardial fat accumulation, cardiometabolic profile and cardiovascular events in patients with stages 3-5 chronic kidney disease | 2015 | CVD mortality not stratified by sex |
| 193 | Cordeiro, A. C., Moraes, A. A., Cerutti, V., Franca, F., Quiroga, B., Amodeo, C., Picotti, J. C., Dutra, L. V., Rodrigues, G. D., Amparo, F. C., Lindholm, B. and Carrero, J. J. | Clinical determinants and prognostic significance of the electrocardiographic strain pattern in chronic kidney disease patients | 2014 | CVD mortality not stratified by sex |
| 194 | Couchoud, C., Labeeuw, M., Moranne, O., Allot, V., Esnault, V., Frimat, L. and Stengel, B. | A clinical score to predict 6-month prognosis in elderly patients starting dialysis for end-stage renal disease | 2009 | CVD mortality not stratified by sex |
| 195 | Couchoud, C., Moranne, O., Frimat, L., Labeeuw, M., Allot, V. and Stengel, B. | Associations between comorbidities, treatment choice and outcome in the elderly with end-stage renal disease | 2007 | CVD mortality not stratified by sex |
| 196 | Cozzolino, M., Biondi, M. L., Banfi, E., Riser, B. L., Mehmeti, F., Cusi, D. and Gallieni, M. | CCN2 (CTGF) gene polymorphism is a novel prognostic risk factor for cardiovascular outcomes in hemodialysis patients | 2010 | CVD mortality not stratified by sex |
| 197 | Cseprekal, O., Egresits, J., Tabak, A., Nemcsik, J., Jarai, Z., Babos, L., Fodor, E., Farkas, K., Godina, G., Karpathi, K. I., Kerkovits, L., Marton, A., Nemcsik-Bencze, Z., Nemeth, Z., Sallai, L., Kiss, I. and Tisler, A. | The significance of micro- and macrovascular biomarkers on cardiovascular outcome in chronic kidney disease: a prospective cohort study | 2016 | CVD mortality not stratified by sex |
| 198 | Dad, T., Tighiouart, H., Joseph, A., Bostom, A., Carpenter, M., Hunsicker, L., Kusek, J. W., Pfeffer, M., Levey, A. S. and Weiner, D. E. | Aspirin Use and Incident Cardiovascular Disease, Kidney Failure, and Death in Stable Kidney Transplant Recipients: A Post Hoc Analysis of the Folic Acid for Vascular Outcome Reduction in Transplantation (FAVORIT) Trial | 2016 | CVD mortality not stratified by sex |
| 199 | Dalrymple, L. S., Katz, R., Kestenbaum, B., Shlipak, M. G., Sarnak, M. J., Stehman-Breen, C., Seliger, S., Siscovick, D., Newman, A. B. and Fried, L. | Chronic kidney disease and the risk of end-stage renal disease versus death | 2011 | CVD mortality not stratified by sex |
| 200 | Damman, K., Andersen, K., Belohlavek, J., Lefkowitz, M. P., Rouleau, J. L., Solomon, S. D., Swedberg, K., Zile, M., Packer, M. and McMurray, J. J. V. | Angiotensin receptor neprilysin inhibition and renal function and in heart failure: results from PARADIGM-HF | 2015 | CVD mortality not stratified by sex |
| 201 | Dasgupta, I., Thomas, G. N., Clarke, J., Sitch, A., Martin, J., Bieber, B., Hecking, M., Karaboyas, A., Pisoni, R., Port, F., Robinson, B. and Rayner, H. | Associations between hemodialysis facility practices to manage fluid volume and intradialytic hypotension and patient outcomes | 2019 | CVD mortality not stratified by sex |
| 202 | David, S., John, S. G., Jefferies, H. J., Sigrist, M. K., Kumpers, P., Kielstein, J. T., Haller, H. and McIntyre, C. W. | Angiopoietin-2 levels predict mortality in CKD patients | 2012 | CVD mortality not stratified by sex |
| 203 | de Bie, M. K., Koopman, M. G., Gaasbeek, A., Dekker, F. W., Maan, A. C., Swenne, C. A., Scherptong, R. W., van Dessel, P. F., Wilde, A. A., Schalij, M. J., Rabelink, T. J. and Jukema, J. W. | Incremental prognostic value of an abnormal baseline spatial QRS-T angle in chronic dialysis patients | 2013 | CVD mortality not stratified by sex |
| 204 | de Roij van Zuijdewijn, C. L., Hansildaar, R., Bots, M. L., Blankestijn, P. J., van den Dorpel, M. A., Grooteman, M. P., Kamp, O., ter Wee, P. M. and Nube, M. J. | Eccentric Left Ventricular Hypertrophy and Sudden Death in Patients with End-Stage Kidney Disease | 2015 | CVD mortality not stratified by sex |
| 205 | de Roij van Zuijdewijn, C. L., ter Wee, P. M., Chapdelaine, I., Bots, M. L., Blankestijn, P. J., van den Dorpel, M. A., Nube, M. J. and Grooteman, M. P. | A Comparison of 8 Nutrition-Related Tests to Predict Mortality in Hemodialysis Patients | 2015 | CVD mortality not stratified by sex |
| 206 | De Roij Van Zuijdewijn, C. L. M., Grooteman, M. P. C., Bots, M. L., Blankestijn, P. J., Steppan, S., Büchel, J., Groenwold, R. H. H., Brandenburg, V., Van Den Dorpel, M. A., Ter Wee, P. M., Nubé, M. J. and Vervloet, M. G. | Serum magnesium and sudden death in European hemodialysis patients | 2015 | CVD mortality not stratified by sex |
| 207 | Del Fabbro, P., Luthi, J. C., Carrera, E., Michel, P., Burnier, M. and Burnand, B. | Anemia and chronic kidney disease are potential risk factors for mortality in stroke patients: a historic cohort study | 2010 | CVD mortality not stratified by sex |
| 208 | DeLoach, S. S., Joffe, M. M., Mai, X., Goral, S. and Rosas, S. E. | Aortic calcification predicts cardiovascular events and all-cause mortality in renal transplantation | 2009 | CVD mortality not stratified by sex |
| 209 | Demirci, C., Aşcı, G., Demirci, M. S., Özkahya, M., Töz, H., Duman, S., Sipahi, S., Erten, S., Tanrısev, M. and Ok, E. | Impedance ratio: a novel marker and a powerful predictor of mortality in hemodialysis patients | 2016 | CVD mortality not stratified by sex |
| 210 | Deo, R., Shou, H., Soliman, E. Z., Yang, W., Arkin, J. M., Zhang, X., Townsend, R. R., Go, A. S., Shlipak, M. G. and Feldman, H. I. | Electrocardiographic Measures and Prediction of Cardiovascular and Noncardiovascular Death in CKD | 2016 | CVD mortality not stratified by sex |
| 211 | Desideri, G., Panichi, V., Paoletti, S., Grassi, D., Bigazzi, R., Beati, S., Bernabini, G., Rosati, A., Ferri, C., Taddei, S. and et al. | Soluble CD40 ligand is predictive of combined cardiovascular morbidity and mortality in patients on haemodialysis at a relatively short-term follow-up | 2011 | CVD mortality not stratified by sex |
| 212 | Di Angelantonio, E., Chowdhury, R., Sarwar, N., Aspelund, T., Danesh, J. and Gudnason, V. | Chronic kidney disease and risk of major cardiovascular disease and non-vascular mortality: prospective population based cohort study | 2010 | CVD mortality not stratified by sex |
| 213 | Di Micco, L., Salvi, P., Bellasi, A., Sirico, M. L. and Di Iorio, B. | Subendocardial viability ratio predicts cardiovascular mortality in chronic kidney disease patients | 2013 | CVD mortality not stratified by sex |
| 214 | Dierkes, J., Domrose, U., Westphal, S., Ambrosch, A., Bosselmann, H. P., Neumann, K. H. and Luley, C. | Cardiac troponin T predicts mortality in patients with end-stage renal disease | 2000 | CVD mortality not stratified by sex |
| 215 | Diez, J. J., Estrada, P., Bajo, M. A., Fernandez-Reyes, M. J., Grande, C., del Peso, G., Heras, M., Molina, A., Iglesias, P., Sanchez-Villanueva, R. and Selgas, R. | High stable serum adiponectin levels are associated with a better outcome in prevalent dialysis patients | 2009 | CVD mortality not stratified by sex |
| 216 | Dobre, M., Brateanu, A., Rashidi, A. and Rahman, M. | Electrocardiogram abnormalities and cardiovascular mortality in elderly patients with CKD | 2012 | CVD mortality not stratified by sex |
| 217 | Dong, K., Huang, X., Zhang, Q., Yu, Z., Ding, J. and Song, H. | A lower baseline glomerular filtration rate predicts high mortality and newly cerebrovascular accidents in acute ischemic stroke patients | 2017 | CVD mortality not stratified by sex |
| 218 | Dos Reis Santos, I., Danaga, A. R., De Carvalho Aguiar, I., Oliveira, E. F., Dias, I. S., Urbano, J. J., Martins, A. A., Ferraz, L. M., Fonseca, N. T., Fernandes, V. and et al. | Cardiovascular risk and mortality in end-stage renal disease patients undergoing dialysis: sleep study, pulmonary function, respiratory mechanics, upper airway collapsibility, autonomic nervous activity, depression, anxiety, stress and quality of life: a prospective, double blind, randomized controlled clinical trial | 2013 | CVD mortality not stratified by sex |
| 219 | Doukky, R., Fughhi, I., Campagnoli, T., Wassouf, M. and Ali, A. | The prognostic value of regadenoson SPECT myocardial perfusion imaging in patients with end-stage renal disease | 2017 | CVD mortality not stratified by sex |
| 220 | Doulgerakis, D., Moyssakis, I., Kapelios, C. J., Eleftheriadou, I., Chorepsima, S., Michail, S. and Tentolouris, N. | Cardiac Autonomic Neuropathy Predicts All-Cause and Cardiovascular Mortality in Patients With End-Stage Renal Failure: A 5-Year Prospective Study | 2017 | CVD mortality not stratified by sex |
| 221 | Dousdampanis, P., Trigka, K. and Fourtounas, C. | Hypomagnesemia, chronic kidney disease and cardiovascular mortality: Pronounced association but unproven causation | 2014 | CVD mortality not stratified by sex |
| 222 | Dragovic, J. T., Popovic, J., Djuric, P., Bulatovic, A., Jankovic, A., Buzadzic, I. and Dimkovic, N. | The 5A Allele of the MMP3-Gene Promoter Polymorphism Is a Risk Factor for Poor Outcome of Hemodialysis Patients | 2017 | CVD mortality not stratified by sex |
| 223 | Dratch, A., Kleine, C. E., Streja, E., Soohoo, M., Park, C., Hsiung, J. T., Rhee, C. M., Obi, Y., Molnar, M. Z., Kovesdy, C. P. and Kalantar-Zadeh, K. | Mean Corpuscular Volume and Mortality in Incident Hemodialysis Patients | 2019 | CVD mortality not stratified by sex |
| 224 | Drawz, P. E., Babineau, D. C., Brecklin, C., He, J., Kallem, R. R., Soliman, E. Z., Xie, D., Appleby, D., Anderson, A. H. and Rahman, M. | Heart rate variability is a predictor of mortality in chronic kidney disease: A report from the CRIC study: CRIC study investigators | 2014 | CVD mortality not stratified by sex |
| 225 | Drechsler, C., Delgado, G., Wanner, C., Blouin, K., Pilz, S., Tomaschitz, A., Kleber, M. E., Dressel, A., Willmes, C., Krane, V., Kramer, B. K., Marz, W., Ritz, E., van Gilst, W. H., van der Harst, P. and de Boer, R. A. | Galectin-3, Renal Function, and Clinical Outcomes: Results from the LURIC and 4D Studies | 2015 | CVD mortality not stratified by sex |
| 226 | Drechsler, C., Evenepoel, P., Vervloet, M. G., Wanner, C., Ketteler, M., Marx, N., Floege, J., Dekker, F. W. and Brandenburg, V. M. | High levels of circulating sclerostin are associated with better cardiovascular survival in incident dialysis patients: results from the NECOSAD study | 2015 | CVD mortality not stratified by sex |
| 227 | Drechsler, C., Grootendorst, D. C., Pilz, S., Tomaschitz, A., Krane, V., Dekker, F., Marz, W., Ritz, E. and Wanner, C. | Wasting and sudden cardiac death in hemodialysis patients: a post hoc analysis of 4D (Die Deutsche Diabetes Dialyse Studie) | 2011 | CVD mortality not stratified by sex |
| 228 | Drechsler, C., Kalim, S., Wenger, J. B., Suntharalingam, P., Hod, T., Thadhani, R. I., Karumanchi, S. A., Wanner, C. and Berg, A. H. | Protein carbamylation is associated with heart failure and mortality in diabetic patients with end-stage renal disease | 2015 | CVD mortality not stratified by sex |
| 229 | Drechsler, C., Krane, V., Winkler, K., Dekker, F. W. and Wanner, C. | Changes in adiponectin and the risk of sudden death, stroke, myocardial infarction, and mortality in hemodialysis patients | 2009 | CVD mortality not stratified by sex |
| 230 | Drechsler, C., Meinitzer, A., Pilz, S., Krane, V., Tomaschitz, A., Ritz, E., Marz, W. and Wanner, C. | Homoarginine, heart failure, and sudden cardiac death in haemodialysis patients | 2011 | CVD mortality not stratified by sex |
| 231 | Drechsler, C., Pilz, S., Obermayer-Pietsch, B., Verduijn, M., Tomaschitz, A., Krane, V., Espe, K., Dekker, F., Brandenburg, V., März, W., Ritz, E. and Wanner, C. | Vitamin D deficiency is associated with sudden cardiac death, combined cardiovascular events, and mortality in haemodialysis patients | 2010 | CVD mortality not stratified by sex |
| 232 | Drechsler, C., Verduijn, M., Pilz, S., Krediet, R. T., Dekker, F. W., Wanner, C., Ketteler, M., Boeschoten, E. W. and Brandenburg, V. | Bone alkaline phosphatase and mortality in dialysis patients | 2011 | CVD mortality not stratified by sex |
| 233 | Dubin, R. F., Deo, R., Bansal, N., Anderson, A. H., Yang, P., Go, A. S., Keane, M., Townsend, R., Porter, A., Budoff, M., Malik, S., He, J., Rahman, M., Wright, J., Cappola, T., Kallem, R., Roy, J., Sha, D. and Shlipak, M. G. | Associations of conventional echocardiographic measures with incident heart failure and mortality: The chronic renal insufficiency cohort | 2017 | CVD mortality not stratified by sex |
| 234 | Edner, M., Benson, L., Dahlstrom, U. and Lund, L. H. | Association between renin-angiotensin system antagonist use and mortality in heart failure with severe renal insufficiency: a prospective propensity score-matched cohort study | 2015 | CVD mortality not stratified by sex |
| 235 | Eisenga, M. F., Nolte, I. M., van der Meer, P., Bakker, S. J. L. and Gaillard, C. | Association of different iron deficiency cutoffs with adverse outcomes in chronic kidney disease | 2018 | CVD mortality not stratified by sex |
| 236 | Ekart, R., Bevc, S., Hojs, N. and Hojs, R. | Derived Subendocardial Viability Ratio and Cardiovascular Events in Patients with Chronic Kidney Disease | 2019 | CVD mortality not stratified by sex |
| 237 | El Amrani, M. and El Kabbaj, D. | Isolated diastolic hypotension in hemodialysis: Risk factor for novel cardiovascular complications and all-cause mortality | 2019 | CVD mortality not stratified by sex |
| 238 | El Hadj Othmane, T., Nemcsik, J., Fekete, B. C., Deák, G., Egresits, J., Fodor, E., Logan, A. G., Németh, Z. K., Járai, Z., Szabó, T., Szathmári, M., Kiss, I. and Tislér, A. | Arterial stiffness in hemodialysis: Which parameter to measure to predict cardiovascular mortality? | 2009 | CVD mortality not stratified by sex |
| 239 | El Said, H. W., Mohamed, O. M., El Said, T. W. and El Serwi, A. B. | Central obesity and risks of cardiovascular events and mortality in prevalent hemodialysis patients | 2017 | CVD mortality not stratified by sex |
| 240 | Elsayed, E. T., Nassra, R. A. and Naga, Y. S. | Peroxisome proliferator-activated receptor-γ–coactivator 1α (PGC-1α) gene expression in chronic kidney disease patients on hemodialysis: relation to hemodialysis-related cardiovascular morbidity and mortality | 2017 | CVD mortality not stratified by sex |
| 241 | Elsharif, M. E. | Mortality rate of patients with end stage renal disease on regular hemodialysis: a single center study | 2011 | CVD mortality not stratified by sex |
| 242 | Engelbertz, C., Reinecke, H., Breithardt, G., Schmieder, R. E., Fobker, M., Fischer, D., Schmitz, B., Pinnschmidt, H. O., Wegscheider, K., Pavenstadt, H. and Brand, E. | Two-year outcome and risk factors for mortality in patients with coronary artery disease and renal failure: The prospective, observational CAD-REF Registry | 2017 | CVD mortality not stratified by sex |
| 243 | Ertas, G., Kozdag, G., Emre, E., Vural, A., Akbulut, T., Ural, D. and Goktekin, O. | Renal function has an effect on cardiovascular mortality in patients with dilated cardiomyopathy | 2012 | CVD mortality not stratified by sex |
| 244 | Esteve-Pastor, M. A., Rivera-Caravaca, J. M., Roldan-Rabadan, I., Roldan, V., Muniz, J., Rana-Miguez, P., Ruiz-Ortiz, M., Cequier, A., Bertomeu-Martinez, V., Badimon, L., Anguita, M., Lip, G. Y. H. and Marin, F. | Relation of Renal Dysfunction to Quality of Anticoagulation Control in Patients with Atrial Fibrillation: The FANTASIIA Registry | 2018 | CVD mortality not stratified by sex |
| 245 | Etter, C., Straub, Y., Hersberger, M., Raz, H. R., Kistler, T., Kiss, D., Wuthrich, R. P., Gloor, H. J., Aerne, D., Wahl, P., Klaghofer, R. and Ambuhl, P. M. | Pregnancy-associated plasma protein-A is an independent short-time predictor of mortality in patients on maintenance haemodialysis | 2010 | CVD mortality not stratified by sex |
| 246 | Fabbian, F., Gallerani, M., Pala, M., De Giorgi, A., Salmi, R., Dentali, F., Ageno, W. and Manfredini, R. | Association between in-hospital mortality and renal dysfunction in 186,219 patients hospitalized for acute stroke in the Emilia-Romagna region of Italy | 2014 | CVD mortality not stratified by sex |
| 247 | Fahrleitner-Pammer, A., Herberth, J., Browning, S. R., Obermayer-Pietsch, B., Wirnsberger, G., Holzer, H., Dobnig, H. and Malluche, H. H. | Bone markers predict cardiovascular events in chronic kidney disease | 2008 | CVD mortality not stratified by sex |
| 248 | Fan, Q., Yan, Y., Gu, L., He, L., Chen, N., Jiang, G., Yuan, L., Xue, J., Zhang, Y., Ma, J. and et al. | Prognostic Value of the Delivery Dialysis Dose on Twice-Weekly Hemodialysis Patients | 2017 | CVD mortality not stratified by sex |
| 249 | Fan, Y., Jin, X., Jiang, M. and Fang, N. | Elevated serum alkaline phosphatase and cardiovascular or all-cause mortality risk in dialysis patients: A meta-Analysis | 2017 | CVD mortality not stratified by sex |
| 250 | Fang, W., Yang, X., Bargman, J. M. and Oreopoulos, D. G. | Association between pulse pressure and mortality in patients undergoing peritoneal dialysis | 2009 | CVD mortality not stratified by sex |
| 251 | Fang, Y. W., Leu, J. G., Tsai, M. H. and Liou, H. H. | Higher intra-dialysis serum phosphorus reduction ratio as a predictor of mortality in patients on long-term hemodialysis | 2019 | CVD mortality not stratified by sex |
| 252 | Farias, M. A., McClellan, W., Soucie, J. M. and Mitch, W. E. | A prospective comparison of methods for determining if cardiovascular disease is a predictor of mortality in dialysis patients | 1994 | CVD mortality not stratified by sex |
| 253 | Farshid, A., Pathak, R., Shadbolt, B., Arnolda, L. and Talaulikar, G. | Diastolic function is a strong predictor of mortality in patients with chronic kidney disease | 2013 | CVD mortality not stratified by sex |
| 254 | Feldreich, T., Nowak, C., Fall, T., Carlsson, A. C., Carrero, J. J., Ripsweden, J., Qureshi, A. R., Heimburger, O., Barany, P., Stenvinkel, P., Vuilleumier, N., Kalra, P. A., Green, D. and Arnlov, J. | Circulating proteins as predictors of cardiovascular mortality in end-stage renal disease | 2019 | CVD mortality not stratified by sex |
| 255 | Feng, S. J., Li, H. and Wang, S. X. | Lower Hydrogen Sulfide Is Associated with Cardiovascular Mortality, Which Involves cPKCbetaII/Akt Pathway in Chronic Hemodialysis Patients | 2015 | CVD mortality not stratified by sex |
| 256 | Feng, Y., Li, Z., Liu, J., Sun, F., Ma, L., Shen, Y. and Zhou, Y. | Association of short-term blood pressure variability with cardiovascular mortality among incident hemodialysis patients | 2018 | CVD mortality not stratified by sex |
| 257 | Fenske, W., Wanner, C., Allolio, B., Drechsler, C., Blouin, K., Lilienthal, J. and Krane, V. | Copeptin levels associate with cardiovascular events in patients with ESRD and type 2 diabetes mellitus | 2011 | CVD mortality not stratified by sex |
| 258 | Fensterseifer, D. M., Karohl, C., Schvartzman, P., Costa, C. A. and Veronese, F. J. | Coronary calcification and its association with mortality in haemodialysis patients | 2009 | CVD mortality not stratified by sex |
| 259 | Fernandez-Laso, V., Sastre, C., Valdivielso, J. M., Betriu, A., Fernandez, E., Egido, J., Martin-Ventura, J. L. and Blanco-Colio, L. M. | Soluble TWEAK and Major Adverse Cardiovascular Events in Patients with CKD | 2016 | CVD mortality not stratified by sex |
| 260 | Ferreira, J. P., Girerd, N., Pellicori, P., Duarte, K., Girerd, S., Pfeffer, M. A., McMurray, J. J., Pitt, B., Dickstein, K., Jacobs, L. and et al. | Renal function estimation and Cockroft-Gault formulas for predicting cardiovascular mortality in population-based, cardiovascular risk, heart failure and post-myocardial infarction cohorts: the Heart 'OMics' in AGEing (HOMAGE) and the high-risk myocardial infarction database initiatives | 2016 | CVD mortality not stratified by sex |
| 261 | Fick, G. M., Johnson, A. M., Hammond, W. S. and Gabow, P. A. | Causes of death in autosomal dominant polycystic kidney disease | 1995 | CVD mortality not stratified by sex |
| 262 | Filippatos, G. S., Ahmed, M. I., Gladden, J. D., Mujib, M., Aban, I. B., Love, T. E., Sanders, P. W., Pitt, B., Anker, S. D. and Ahmed, A. | Hyperuricaemia, chronic kidney disease, and outcomes in heart failure: potential mechanistic insights from epidemiological data | 2011 | CVD mortality not stratified by sex |
| 263 | Fischer, M. J., Ho, P. M., McDermott, K., Lowy, E. and Parikh, C. R. | Chronic kidney disease is associated with adverse outcomes among elderly patients taking clopidogrel after hospitalization for acute coronary syndrome | 2013 | CVD mortality not stratified by sex |
| 264 | Fischer, M. J., Kimmel, P. L., Greene, T., Gassman, J. J., Wang, X., Brooks, D. H., Charleston, J., Dowie, D., Thornley-Brown, D., Cooper, L. A., Bruce, M. A., Kusek, J. W., Norris, K. C. and Lash, J. P. | Elevated depressive affect is associated with adverse cardiovascular outcomes among African Americans with chronic kidney disease | 2011 | CVD mortality not stratified by sex |
| 265 | Fitzpatrick, J., Sozio, S. M., Jaar, B. G., McAdams-DeMarco, M. A., Estrella, M. M., Tereshchenko, L. G., Monroy-Trujillo, J. M. and Parekh, R. S. | Association of Abdominal Adiposity with Cardiovascular Mortality in Incident Hemodialysis | 2018 | CVD mortality not stratified by sex |
| 266 | Flores Gama, C., Rosales, L. M., Ouellet, G., Dou, Y., Thijssen, S., Usvyat, L., Zhang, H., Kuntsevich, V., Levin, N. W. and Kotanko, P. | Plasma Gelsolin and Its Association with Mortality and Hospitalization in Chronic Hemodialysis Patients | 2017 | CVD mortality not stratified by sex |
| 267 | Flueckiger, P., Pastan, S., Goyal, A., McClellan, W. W. and Patzer, R. E. | Associations of ECG interval prolongations with mortality among ESRD patients evaluated for renal transplantation | 2014 | CVD mortality not stratified by sex |
| 268 | Flythe, J. E., Inrig, J. K., Shafi, T., Chang, T. I., Cape, K., Dinesh, K., Kunaparaju, S. and Brunelli, S. M. | Association of intradialytic blood pressure variability with increased all-cause and cardiovascular mortality in patients treated with long-term hemodialysis | 2013 | CVD mortality not stratified by sex |
| 269 | Flythe, J. E., Kimmel, S. E. and Brunelli, S. M. | Rapid fluid removal during dialysis is associated with cardiovascular morbidity and mortality | 2011 | CVD mortality not stratified by sex |
| 270 | Foley, R. N. and Collins, A. J. | A novel model of cardiovascular risk based on kidney function | 2011 | CVD mortality not stratified by sex |
| 271 | Foley, R. N., Gilbertson, D. T., Murray, T. and Collins, A. J. | Long interdialytic interval and mortality among patients receiving hemodialysis | 2011 | CVD mortality not stratified by sex |
| 272 | Foley, R. N., Herzog, C. A. and Collins, A. J. | Blood pressure and long-term mortality in United States hemodialysis patients: USRDS Waves 3 and 4 Study | 2002 | CVD mortality not stratified by sex |
| 273 | Foley, R. N., Murray, A. M., Li, S., Herzog, C. A., McBean, A. M., Eggers, P. W. and Collins, A. J. | Chronic kidney disease and the risk for cardiovascular disease, renal replacement, and death in the United States Medicare population, 1998 to 1999 | 2005 | CVD mortality not stratified by sex |
| 274 | Foley, R. N., Parfrey, P. S., Harnett, J. D., Kent, G. M., Murray, D. C. and Barre, P. E. | Hypoalbuminemia, cardiac morbidity, and mortality in end-stage renal disease | 1996 | CVD mortality not stratified by sex |
| 275 | Formanowicz, D., Wanic-Kossowska, M., Pawliczak, E., Radom, M. and Formanowicz, P. | Usefulness of serum interleukin-18 in predicting cardiovascular mortality in patients with chronic kidney disease-systems and clinical approach | 2015 | CVD mortality not stratified by sex |
| 276 | Formiga, F., Moreno-Gonzalez, R., Chivite, D., Casado, J., Escrihuela-Vidal, F. and Corbella, X. | Clinical characteristics and one-year mortality according to admission renal function in patients with a first acute heart failure hospitalization | 2018 | CVD mortality not stratified by sex |
| 277 | Formiga, F., Moreno-Gonzalez, R., Chivite, D., Yun, S., Franco, J., Ariza-Solé, A. and Corbella, X. | Sex differences in 1-year mortality risks in older patients experiencing a first acute heart failure hospitalization | 2019 | CVD mortality not stratified by sex |
| 278 | Fortier, C., Mac-Way, F., Desmeules, S., Marquis, K., De Serres, S. A., Lebel, M., Boutouyrie, P. and Agharazii, M. | Aortic-brachial stiffness mismatch and mortality in dialysis population | 2015 | CVD mortality not stratified by sex |
| 279 | Fox, C. S., Matsushita, K., Woodward, M., Bilo, H. J., Chalmers, J., Heerspink, H. J., Lee, B. J., Perkins, R. M., Rossing, P., Sairenchi, T., Tonelli, M., Vassalotti, J. A., Yamagishi, K., Coresh, J., de Jong, P. E., Wen, C. P. and Nelson, R. G. | Associations of kidney disease measures with mortality and end-stage renal disease in individuals with and without diabetes: a meta-analysis | 2012 | CVD mortality not stratified by sex |
| 280 | Freisinger, E., Sehner, S., Malyar, N. M., Suling, A., Reinecke, H. and Wegscheider, K. | Nationwide Routine-Data Analysis of Sex Differences in Outcome of Acute Myocardial Infarction | 2018 | CVD mortality not stratified by sex |
| 281 | Friedman, A. N., Yu, Z., Denski, C., Tamez, H., Wenger, J., Thadhani, R., Li, Y. and Watkins, B. | Fatty acids and other risk factors for sudden cardiac death in patients starting hemodialysis | 2013 | CVD mortality not stratified by sex |
| 282 | Frostegård, A. G., Hua, X., Su, J., Carrero, J. J., Heimbürger, O., Bárány, P., Stenvinkel, P. and Frostegård, J. | Immunoglobulin (Ig)M antibodies against oxidized cardiolipin but not native cardiolipin are novel biomarkers in haemodialysis patients, associated negatively with mortality | 2013 | CVD mortality not stratified by sex |
| 283 | Fujii, H., Kim, J. I., Yoshiya, K., Nishi, S. and Fukagawa, M. | Clinical characteristics and cardiovascular outcomes of hemodialysis patients with atrial fibrillation: A prospective follow-up study | 2011 | CVD mortality not stratified by sex |
| 284 | Fukasawa, H., Ishibuchi, K., Kaneko, M., Niwa, H., Yasuda, H., Kumagai, H. and Furuya, R. | Red Blood Cell Distribution Width Is Associated With All-Cause and Cardiovascular Mortality in Hemodialysis Patients | 2017 | CVD mortality not stratified by sex |
| 285 | Fukasawa, H., Kaneko, M., Niwa, H., Matsuyama, T., Yasuda, H., Kumagai, H. and Furuya, R. | Lower thigh muscle mass is associated with all-cause and cardiovascular mortality in elderly hemodialysis patients | 2017 | CVD mortality not stratified by sex |
| 286 | Fukuma, S., Yamaguchi, T., Hashimoto, S., Nakai, S., Iseki, K., Tsubakihara, Y. and Fukuhara, S. | Erythropoiesis-stimulating agent responsiveness and mortality in hemodialysis patients: Results from a cohort study from the dialysis registry in Japan | 2012 | CVD mortality not stratified by sex |
| 287 | Fukuoka, K., Nakao, K., Morimoto, H., Nakao, A., Takatori, Y., Arimoto, K., Taki, M., Wada, J. and Makino, H. | Glycated albumin levels predict long-term survival in diabetic patients undergoing haemodialysis | 2008 | CVD mortality not stratified by sex |
| 288 | Furuhashi, T., Moroi, M., Joki, N., Hase, H., Minakawa, M., Masai, H., Kunimasa, T., Fukuda, H. and Sugi, K. | Predictors of cardiovascular events in hemodialysis patients after stress myocardial perfusion imaging | 2013 | CVD mortality not stratified by sex |
| 289 | Furuhashi, T., Moroi, M., Joki, N., Hase, H., Minakawa, M., Masai, H., Kunimasa, T., Fukuda, H. and Sugi, K. | Prediction of cardiovascular events in pre-dialysis chronic kidney disease patients with normal SPECT myocardial perfusion imaging | 2014 | CVD mortality not stratified by sex |
| 290 | Furuya, F., Shimura, H., Takahashi, K., Akiyama, D., Motosugi, A., Ikegishi, Y., Haraguchi, K. and Kobayashi, T. | Skin autofluorescence is a predictor of cardiovascular disease in chronic kidney disease patients | 2015 | CVD mortality not stratified by sex |
| 291 | Galil, A. G., Pinheiro, H. S., Chaoubah, A., Costa, D. M. and Bastos, M. G. | Chronic kidney disease increases cardiovascular unfavourable outcomes in outpatients with heart failure | 2009 | CVD mortality not stratified by sex |
| 292 | Ganesh, S. K., Stack, A. G., Levin, N. W., Hulbert-Shearon, T. and Port, F. K. | Association of elevated serum PO(4), Ca x PO(4) product, and parathyroid hormone with cardiac mortality risk in chronic hemodialysis patients | 2001 | CVD mortality not stratified by sex |
| 293 | Gao, B., Wu, S., Wang, J., Yang, C., Chen, S., Hou, J., Li, J., Yang, Y., He, K., Zhao, M., Chen, M. and Zhang, L. | Clinical features and long-term outcomes of diabetic kidney disease – A prospective cohort study from China | 2019 | CVD mortality not stratified by sex |
| 294 | Gao, S., Xu, J., Zhang, S. and Jin, J. | Meta-Analysis of the Association between Fibroblast Growth Factor 23 and Mortality and Cardiovascular Events in Hemodialysis Patients | 2019 | CVD mortality not stratified by sex |
| 295 | Garimella, P. S., Katz, R., Patel, K. V., Kritchevsky, S. B., Parikh, C. R., Ix, J. H., Fried, L. F., Newman, A. B., Shlipak, M. G., Harris, T. B. and Sarnak, M. J. | Association of Serum Erythropoietin With Cardiovascular Events, Kidney Function Decline, and Mortality: The Health Aging and Body Composition Study | 2016 | CVD mortality not stratified by sex |
| 296 | Genovesi, S., Rossi, E., Gallieni, M., Stella, A., Badiali, F., Conte, F., Pasquali, S., Bertoli, S., Ondei, P., Bonforte, G., Pozzi, C., Rebora, P., Valsecchi, M. G. and Santoro, A. | Warfarin use, mortality, bleeding and stroke in haemodialysis patients with atrial fibrillation | 2015 | CVD mortality not stratified by sex |
| 297 | Genovesi, S., Vincenti, A., Rossi, E., Pogliani, D., Acquistapace, I., Stella, A. and Valsecchi, M. G. | Atrial Fibrillation and Morbidity and Mortality in a Cohort of Long-term Hemodialysis Patients | 2008 | CVD mortality not stratified by sex |
| 298 | Gheorghiade, M., Böhm, M., Greene, S. J., Fonarow, G. C., Lewis, E. F., Zannad, F., Solomon, S. D., Baschiera, F., Botha, J., Hua, T. A. and et al. | Effect of aliskiren on postdischarge mortality and heart failure readmissions among patients hospitalized for heart failure: the ASTRONAUT randomized trial | 2013 | CVD mortality not stratified by sex |
| 299 | Giamouzis, G., Sui, X., Love, T. E., Butler, J., Young, J. B. and Ahmed, A. | A propensity-matched study of the association of cardiothoracic ratio with morbidity and mortality in chronic heart failure | 2008 | CVD mortality not stratified by sex |
| 300 | Go, A. S., Chertow, G. M., Fan, D., McCulloch, C. E. and Hsu, C. Y. | Chronic kidney disease and the risks of death, cardiovascular events, and hospitalization | 2004 | CVD mortality not stratified by sex |
| 301 | Gohda, T., Maruyama, S., Kamei, N., Yamaguchi, S., Shibata, T., Murakoshi, M., Horikoshi, S., Tomino, Y., Ohsawa, I., Gotoh, H., Nojiri, S. and Suzuki, Y. | Circulating TNF Receptors 1 and 2 Predict Mortality in Patients with End-stage Renal Disease Undergoing Dialysis | 2017 | CVD mortality not stratified by sex |
| 302 | Gowdak, L. H., de Paula, F. J., Cesar, L. A., Filho, E. E., Ianhez, L. E., Krieger, E. M., Ramires, J. A. and De Lima, J. J. | Diabetes and coronary artery disease impose similar cardiovascular morbidity and mortality on renal transplant candidates | 2007 | CVD mortality not stratified by sex |
| 303 | Gracia-Iguacel, C., Gonzalez-Parra, E., Egido, J., Lindholm, B., Mahillo, I., Carrero, J. J. and Ortiz, A. | Cortisol levels are associated with mortality risk in hemodialysis patients | 2014 | CVD mortality not stratified by sex |
| 304 | Green, D., Ritchie, J. P., Abidin, N., New, D. I. and Kalra, P. A. | The association of ECG and echocardiographic abnormalities with sudden cardiac death in a dialysis patient cohort | 2014 | CVD mortality not stratified by sex |
| 305 | Gregg, L. P., Adams-Huet, B., Li, X., Colbert, G., Jain, N., de Lemos, J. A. and Hedayati, S. S. | Effect Modification of Chronic Kidney Disease on the Association of Circulating and Imaging Cardiac Biomarkers With Outcomes | 2017 | CVD mortality not stratified by sex |
| 306 | Gregg, L. P., Tio, M. C., Li, X., Adams-Huet, B., Lemos, J. A. D. and Hedayati, S. S. | Association of Monocyte Chemoattractant Protein-1 with Death and Atherosclerotic Events in Chronic Kidney Disease | 2018 | CVD mortality not stratified by sex |
| 307 | Grooteman, M. P., van den Dorpel, M. A., Bots, M. L., Penne, E. L., van der Weerd, N. C., Mazairac, A. H., den Hoedt, C. H., van der Tweel, I., Lévesque, R., Nubé, M. J. and et al. | Effect of online hemodiafiltration on all-cause mortality and cardiovascular outcomes | 2012 | CVD mortality not stratified by sex |
| 308 | Grzegorzewska, A. E., Niepolski, L., Swiderska, M. K., Mostowska, A., Stolarek, I., Warchol, W., Figlerowicz, M. and Jagodzinski, P. P. | ENHO, RXRA, and LXRA polymorphisms and dyslipidaemia, related comorbidities and survival in haemodialysis patients | 2018 | CVD mortality not stratified by sex |
| 309 | Guerrero-Riscos, M. A., Montes-Delgado, R., Seda-Guzman, M. and Praena-Fernandez, J. M. | Erythropoietin resistance and survival in non-dialysis patients with stage 4-5 chronic kidney disease and heart disease | 2012 | CVD mortality not stratified by sex |
| 310 | Hamada, S. and Gulliford, M. C. | Multiple risk factor control, mortality and cardiovascular events in type 2 diabetes and chronic kidney disease: A population-based cohort study | 2018 | CVD mortality not stratified by sex |
| 311 | Hamaguchi, S., Tsuchihashi-Makaya, M., Kinugawa, S., Yokota, T., Ide, T., Takeshita, A. and Tsutsui, H. | Chronic kidney disease as an independent risk for long-term adverse outcomes in patients hospitalized with heart failure in Japan. Report from the Japanese Cardiac Registry of Heart Failure in Cardiology (JCARE-CARD) | 2009 | CVD mortality not stratified by sex |
| 312 | Hanada, S., Ando, R., Naito, S., Kobayashi, N., Wakabayashi, M., Hata, T. and Sasaki, S. | Assessment and significance of abdominal aortic calcification in chronic kidney disease | 2010 | CVD mortality not stratified by sex |
| 313 | Hanatani, S., Izumiya, Y., Onoue, Y., Tanaka, T., Yamamoto, M., Ishida, T., Yamamura, S., Kimura, Y., Araki, S., Arima, Y., Nakamura, T., Fujisue, K., Takashio, S., Sueta, D., Sakamoto, K., Yamamoto, E., Kojima, S., Kaikita, K. and Tsujita, K. | Non-invasive testing for sarcopenia predicts future cardiovascular events in patients with chronic kidney disease | 2018 | CVD mortality not stratified by sex |
| 314 | Hasegawa, M., Ishii, J., Kitagawa, F., Kanayama, K., Takahashi, H., Ozaki, Y. and Yuzawa, Y. | Prognostic value of highly sensitive troponin T on cardiac events in patients with chronic kidney disease not on dialysis | 2013 | CVD mortality not stratified by sex |
| 315 | Hasegawa, M., Ishii, J., Kitagawa, F., Takahashi, H., Sugiyama, K., Tada, M., Kanayama, K., Takahashi, K., Hayashi, H., Koide, S., Nakai, S., Ozaki, Y. and Yuzawa, Y. | Plasma Neutrophil Gelatinase-Associated Lipocalin as a Predictor of Cardiovascular Events in Patients with Chronic Kidney Disease | 2016 | CVD mortality not stratified by sex |
| 316 | Hasegawa, M., Ishii, J., Kitagawa, F., Takahashi, K., Hayashi, H., Koide, S., Tomita, M., Takahashi, H., Ozaki, Y. and Yuzawa, Y. | Urinary neutrophil gelatinase-associated lipocalin as a predictor of cardiovascular events in patients with chronic kidney disease | 2015 | CVD mortality not stratified by sex |
| 317 | Hashemi, A., Nourbakhsh, S., Asgari, S., Mirbolouk, M., Azizi, F. and Hadaegh, F. | Blood pressure components and incident cardiovascular disease and mortality events among Iranian adults with chronic kidney disease during over a decade long follow-up: A prospective cohort study | 2018 | CVD mortality not stratified by sex |
| 318 | Hassan, H. C., Howlin, K., Jefferys, A., Spicer, S. T., Aravindan, A. N., Suryanarayanan, G., Hall, B. M., Cleland, B. D., Wong, J. K., Suranyi, M. G. and Makris, A. | High-sensitivity troponin as a predictor of cardiac events and mortality in the stable dialysis population | 2014 | CVD mortality not stratified by sex |
| 319 | Hassan, S. B., El-demery, A. B., Ahmed, A. I. and Abukhalil, R. E. | Soluble TWEAK and cardiovascular morbidity and mortality in chronic kidney disease patients | 2012 | CVD mortality not stratified by sex |
| 320 | Havel, M., Kaminek, M., Metelkova, I., Budikova, M., Henzlova, L., Koranda, P., Zadrazil, J. and Kincl, V. | Prognostic value of myocardial perfusion imaging and coronary artery calcium measurements in patients with end-stage renal disease | 2015 | CVD mortality not stratified by sex |
| 321 | Hayano, J., Yasuma, F., Watanabe, E., Carney, R. M., Stein, P. K., Blumenthal, J. A., Arsenos, P., Gatzoulis, K. A., Takahashi, H., Ishii, H., Kiyono, K., Yamamoto, Y., Yoshida, Y., Yuda, E. and Kodama, I. | Blunted cyclic variation of heart rate predicts mortality risk in post-myocardial infarction, end-stage renal disease, and chronic heart failure patients | 2017 | CVD mortality not stratified by sex |
| 322 | Hayashi, T., Kimura, T., Yasuda, K., Sasaki, K., Obi, Y., Nagayama, H., Ohno, M., Uematsu, K., Tamai, T., Nishide, T., Rakugi, H. and Isaka, Y. | Early nephrology referral 6 months before dialysis initiation can reduce early death but does not improve long-term cardiovascular outcome on dialysis | 2016 | CVD mortality not stratified by sex |
| 323 | Hayashi, T., Kimura, T., Yasuda, K., Sasaki, K., Obi, Y., Rakugi, H. and Isaka, Y. | Cardiac troponin T elevation at dialysis initiation is associated with all-cause and cardiovascular mortality on dialysis in patients without diabetic nephropathy | 2017 | CVD mortality not stratified by sex |
| 324 | Hebert, K., Dias, A., Delgado, M. C., Franco, E., Tamariz, L., Steen, D., Trahan, P., Major, B. and Arcement, L. M. | Epidemiology and survival of the five stages of chronic kidney disease in a systolic heart failure population | 2010 | CVD mortality not stratified by sex |
| 325 | Hee, L., Nguyen, T., Whatmough, M., Descallar, J., Chen, J., Kapila, S., French, J. K. and Thomas, L. | Left atrial volume and adverse cardiovascular outcomes in unselected patients with and without CKD | 2014 | CVD mortality not stratified by sex |
| 326 | Heine, G. H., Ulrich, C., Seibert, E., Seiler, S., Marell, J., Reichart, B., Krause, M., Schlitt, A., Köhler, H. and Girndt, M. | CD14++CD16+ monocytes but not total monocyte numbers predict cardiovascular events in dialysis patients | 2008 | CVD mortality not stratified by sex |
| 327 | Helve, S., Laine, M., Sinisalo, J., Helantera, I., Hanninen, H., Lammintausta, O., Lehtonen, J., Finne, P. and Nieminen, T. | Even mild reversible myocardial perfusion defects predict mortality in patients evaluated for kidney transplantation | 2018 | CVD mortality not stratified by sex |
| 328 | Herselman, M., Esau, N., Kruger, J. M., Labadarios, D. and Moosa, M. R. | Relationship between body mass index and mortality in adults on maintenance hemodialysis: a systematic review | 2010 | CVD mortality not stratified by sex |
| 329 | Hinderliter, A., Padilla, R. L., Gillespie, B. W., Levin, N. W., Kotanko, P., Kiser, M., Finkelstein, F., Rajagopalan, S. and Saran, R. | Association of carotid intima-media thickness with cardiovascular risk factors and patient outcomes in advanced chronic kidney disease: the RRI-CKD study | 2015 | CVD mortality not stratified by sex |
| 330 | Hirata, Y., Sugiyama, S., Yamamoto, E., Matsuzawa, Y., Akiyama, E., Kusaka, H., Fujisue, K., Kurokawa, H., Matsubara, J., Sugamura, K., Maeda, H., Iwashita, S., Jinnouchi, H., Matsui, K. and Ogawa, H. | Endothelial function and cardiovascular events in chronic kidney disease | 2014 | CVD mortality not stratified by sex |
| 331 | Hitsumoto, T. | Clinical Usefulness of the Cardio-Ankle Vascular Index as a Predictor of Primary Cardiovascular Events in Patients With Chronic Kidney Disease | 2018 | CVD mortality not stratified by sex |
| 332 | Hocher, B., Ziebig, R., Altermann, C., Krause, R., Asmus, G., Richter, C. M., Slowinski, T., Sinha, P. and Neumayer, H. H. | Different impact of biomarkers as mortality predictors among diabetic and nondiabetic patients undergoing hemodialysis | 2003 | CVD mortality not stratified by sex |
| 333 | Holme, I., Fayyad, R., Faergeman, O., Kastelein, J. J., Olsson, A. G., Tikkanen, M. J., Larsen, M. L., Lindahl, C., Holdaas, H. and Pedersen, T. R. | Cardiovascular outcomes and their relationships to lipoprotein components in patients with and without chronic kidney disease: results from the IDEAL trial | 2010 | CVD mortality not stratified by sex |
| 334 | Hong, D., Wu, S., Pu, L., Wang, F., Wang, J., Wang, Z., Gao, H., Zhang, Y., Deng, F., Li, G., He, Q. and Wang, L. | Abdominal aortic calcification is not superior over other vascular calcification in predicting mortality in hemodialysis patients: A retrospective observational study | 2013 | CVD mortality not stratified by sex |
| 335 | Hoppe, K., Schwermer, K., Olewicz-Gawlik, A., Klysz, P., Kawka, A., Baum, E., Sikorska, D., Ścigacz, K., Roszak, M., Lindholm, B., Pawlaczyk, K. and Oko, A. | Dialysis vintage and cardiovascular injury as factors influencing long-term survival in peritoneal dialysis and hemodialysis | 2017 | CVD mortality not stratified by sex |
| 336 | Hoshino, J., Nagai, K., Kai, H., Saito, C., Ito, Y., Asahi, K., Kondo, M., Iseki, K., Iseki, C., Okada, H., Kashihara, N., Narita, I., Wada, T., Combe, C., Pisoni, R. L., Robinson, B. M. and Yamagata, K. | A nationwide prospective cohort study of patients with advanced chronic kidney disease in Japan: The reach-J CKD cohort study | 2018 | CVD mortality not stratified by sex |
| 337 | Hov, G. G., Aasarod, K. I., Sagen, E. and Asberg, A. | Arginine, dimethylated arginine and homoarginine in relation to cardiovascular risk in patients with moderate chronic kidney disease | 2015 | CVD mortality not stratified by sex |
| 338 | Hsieh, Y. P., Chang, C. C., Kor, C. T., Yang, Y., Wen, Y. K. and Chiu, P. F. | The Predictive Role of Red Cell Distribution Width in Mortality among Chronic Kidney Disease Patients | 2016 | CVD mortality not stratified by sex |
| 339 | Hsieh, Y. P., Chang, C. C., Kor, C. T., Yang, Y., Wen, Y. K. and Chiu, P. F. | Mean Corpuscular Volume and Mortality in Patients with CKD | 2017 | CVD mortality not stratified by sex |
| 340 | Hsu, C. W., Yen, T. H., Chen, K. H., Lin-Tan, D. T., Lin, J. L., Weng, C. H. and Huang, W. H. | Effect of blood cadmium level on mortality in patients undergoing maintenance hemodialysis | 2015 | CVD mortality not stratified by sex |
| 341 | Hsu, H. J., Yen, C. H., Hsu, K. H., Lee, C. C., Chang, S. J., Wu, I. W., Sun, C. Y., Chou, C. C., Yu, C. C., Hsieh, M. F., Chen, C. Y., Hsu, C. Y., Weng, C. H., Tsai, C. J. and Wu, M. S. | Association between cold dialysis and cardiovascular survival in hemodialysis patients | 2012 | CVD mortality not stratified by sex |
| 342 | Hsu, W. L., Li, S. Y., Liu, J. S., Huang, P. H., Lin, S. J., Hsu, C. C., Lin, Y. P. and Tarng, D. C. | High Uric Acid Ameliorates Indoxyl Sulfate-Induced Endothelial Dysfunction and Is Associated with Lower Mortality among Hemodialysis Patients | 2017 | CVD mortality not stratified by sex |
| 343 | Hu, W. S., Lee, J. H., Tsai, M. K. and Wen, C. P. | A novel cardiovascular death prediction model for Chinese individuals: A prospective cohort study of 381,963 study participants | 2017 | CVD mortality not stratified by sex |
| 344 | Huang, C. L., Jong, Y. S., Wu, Y. W., Wang, W. J., Hsieh, A. R., Chao, C. L., Chen, W. J. and Yang, W. S. | Association of plasma thrombospondin-1 level with cardiovascular disease and mortality in hemodialysis patients | 2015 | CVD mortality not stratified by sex |
| 345 | Huang, J. C., Chen, S. C., Su, H. M., Chang, J. M., Hwang, S. J. and Chen, H. C. | Performance of the Framingham risk score in patients receiving hemodialysis | 2013 | CVD mortality not stratified by sex |
| 346 | Huang, J. C., Lin, H. Y. H., Lim, L. M., Chen, S. C., Chang, J. M., Hwang, S. J., Tsai, J. C., Hung, C. C. and Chen, H. C. | Body mass index, mortality, and gender difference in advanced chronic kidney disease | 2015 | CVD mortality not stratified by sex |
| 347 | Huang, J. C., Tsai, Y. C., Wu, P. Y., Lee, J. J., Chen, S. C., Chiu, Y. W., Hsu, Y. L., Chang, J. M. and Chen, H. C. | Independent Association of Overhydration with All-Cause and Cardiovascular Mortality Adjusted for Global Left Ventricular Longitudinal Systolic Strain and E/E' Ratio in Maintenance Hemodialysis Patients | 2018 | CVD mortality not stratified by sex |
| 348 | Huang, S. T., Yu, T. M., Ke, T. Y., Wu, M. J., Chuang, Y. W., Li, C. Y., Chiu, C. W., Lin, C. L., Liang, W. M., Chou, T. C. and Kao, C. H. | Syncope and collapse are associated with an increased risk of cardiovascular disease and mortality in patients undergoing dialysis | 2018 | CVD mortality not stratified by sex |
| 349 | Huang, W. Y., Weng, W. C., Peng, T. I., Chien, Y. Y., Wu, C. L., Lee, M., Hung, C. C. and Chen, K. H. | Association of hyponatremia in acute stroke stage with three-year mortality in patients with first-ever ischemic stroke | 2012 | CVD mortality not stratified by sex |
| 350 | Hughes, J., Green, D., Chiu, D. Y. Y., Abidin, N. and Kalra, P. A. | The Association of Echocardiographic Peak Systolic Strain Rate with Cardiovascular Outcomes in Haemodialysis Patients | 2018 | CVD mortality not stratified by sex |
| 351 | Hung, S. C., Hsu, T. W., Lin, Y. P. and Tarng, D. C. | Decoy receptor 3, a novel inflammatory marker, and mortality in hemodialysis patients | 2012 | CVD mortality not stratified by sex |
| 352 | Hung, Y. M. and Lee, S. S. J. | Analysis of early and late mortality of chronic hemodialysis patients in a hemodialysis center of Southern Taiwan | 2003 | CVD mortality not stratified by sex |
| 353 | Hur, S. H., Won, K. B., Kim, I. C., Bae, J. H., Choi, D. J., Ahn, Y. K., Park, J. S., Kim, H. S., Choi, R. K., Choi, D., Kim, J. H., Han, K. R., Park, H. S., Choi, S. Y., Yoon, J. H., Gwon, H. C., Rha, S. W., Jang, W., Bae, J. W., Hwang, K. K., Lim, D. S., Jung, K. T., Oh, S. K., Lee, J. H., Shin, E. S. and Kim, K. S. | Comparison of 2-year clinical outcomes between diabetic versus nondiabetic patients with acute myocardial infarction after 1-month stabilization: Analysis of the prospective registry of DIAMOND (DIabetic acute myocardial infarctiON Disease) in Korea: an observational registry study | 2016 | CVD mortality not stratified by sex |
| 354 | Hutton, H. L., Levin, A., Gill, J., Djurdjev, O., Tang, M. and Barbour, S. J. | Cardiovascular risk is similar in patients with glomerulonephritis compared to other types of chronic kidney disease: a matched cohort study | 2017 | CVD mortality not stratified by sex |
| 355 | Hwang, H. S., Kim, S. Y., Hong, Y. A., Cho, W. K., Chang, Y. K., Shin, S. J., Yang, C. W., Kim, S. Y. and Yoon, H. E. | Clinical impact of coexisting retinopathy and vascular calcification on chronic kidney disease progression and cardiovascular events | 2016 | CVD mortality not stratified by sex |
| 356 | Hwang, H. S., Park, M. W., Yoon, H. E., Chang, Y. K., Yang, C. W., Kim, S. Y., Cho, J. S., Kim, C. J., Park, G. M., Park, C. S., Choi, Y. S., Koh, Y. S., Lee, J. M., Shin, D. I., Seo, S. M., Jeon, D. S., Moon, K. W., Yoo, K. D., Kim, H. Y., Kim, D. B., Park, H. J., Kim, P. J., Chang, K., Chung, W. S., Seung, K. B., Jeong, M. H., Her, S. H. and Ahn, Y. | Clinical significance of chronic kidney disease and atrial fibrillation on morbidity and mortality in patients with acute myocardial infarction | 2014 | CVD mortality not stratified by sex |
| 357 | Ignjatovic, A. M., Cvetkovic, T. P., Pavlovic, R. M., Dordevic, V. M., Milosevic, Z. G., Dordevic, V. B., Pavlovic, D. D., Stojanovic, I. R. and Bogdanovic, D. | Endothelial dysfunction, inflammation and malnutrition markers as predictors of mortality in dialysis patients: multimarker approach | 2013 | CVD mortality not stratified by sex |
| 358 | Iimori, S., Mori, Y., Akita, W., Takada, S., Kuyama, T., Ohnishi, T., Shikuma, S., Ishigami, J., Tajima, M., Asai, T. and et al. | Effects of sevelamer hydrochloride on mortality, lipid abnormality and arterial stiffness in hemodialyzed patients: a propensity-matched observational study | 2012 | CVD mortality not stratified by sex |
| 359 | Iimori, S., Naito, S., Noda, Y., Nishida, H., Kihira, H., Yui, N., Okado, T., Sasaki, S., Uchida, S. and Rai, T. | Anaemia management and mortality risk in newly visiting patients with chronic kidney disease in Japan: The CKD-ROUTE study | 2015 | CVD mortality not stratified by sex |
| 360 | Inaguma, D., Koide, S., Takahashi, K., Hayashi, H., Hasegawa, M. and Yuzawa, Y. | Association between resting heart rate just before starting the first dialysis session and mortality: A multicentre prospective cohort study | 2018 | CVD mortality not stratified by sex |
| 361 | Inaguma, D., Murata, M., Tanaka, A. and Shinjo, H. | Relationship between mortality and speed of eGFR decline in the 3 months prior to dialysis initiation | 2017 | CVD mortality not stratified by sex |
| 362 | Inaguma, D., Tanaka, A. and Shinjo, H. | Physical function at the time of dialysis initiation is associated with subsequent mortality | 2017 | CVD mortality not stratified by sex |
| 363 | Inoue, T., Ogawa, T., Ishida, H., Ando, Y. and Nitta, K. | Aortic arch calcification evaluated on chest X-ray is a strong independent predictor of cardiovascular events in chronic hemodialysis patients | 2012 | CVD mortality not stratified by sex |
| 364 | Inrig, J. K., Oddone, E. Z., Hasselblad, V., Gillespie, B., Patel, U. D., Reddan, D., Toto, R., Himmelfarb, J., Winchester, J. F., Stivelman, J. and et al. | Association of intradialytic blood pressure changes with hospitalization and mortality rates in prevalent ESRD patients | 2007 | CVD mortality not stratified by sex |
| 365 | Ishii, H., Takahashi, H., Ito, Y., Aoyama, T., Kamoi, D., Sakakibara, T., Umemoto, N., Kumada, Y., Suzuki, S. and Murohara, T. | The Association of Ankle Brachial Index, Protein-Energy Wasting, and Inflammation Status with Cardiovascular Mortality in Patients on Chronic Hemodialysis | 2017 | CVD mortality not stratified by sex |
| 366 | Ishimura, E., Okuno, S., Kono, K., Fujino-Kato, Y., Maeno, Y., Kagitani, S., Tsuboniwa, N., Nagasue, K., Maekawa, K., Yamakawa, T., Inaba, M. and Nishizawa, Y. | Glycemic control and survival of diabetic hemodialysis patients--importance of lower hemoglobin A1C levels | 2009 | CVD mortality not stratified by sex |
| 367 | Isshiki, K., Nishio, T., Isono, M., Makiishi, T., Shikano, T., Tomita, K., Nishio, T., Kanasaki, M., Maegawa, H. and Uzu, T. | Glycated Albumin Predicts the Risk of Mortality in Type 2 Diabetic Patients on Hemodialysis: Evaluation of a Target Level for Improving Survival | 2014 | CVD mortality not stratified by sex |
| 368 | Ix, J. H., Shlipak, M. G., Sarnak, M. J., Beck, G. J., Greene, T., Wang, X., Kusek, J. W., Collins, A. J., Levey, A. S. and Menon, V. | Fetuin-A is not associated with mortality in chronic kidney disease | 2007 | CVD mortality not stratified by sex |
| 369 | Jaar, B. G., Coresh, J., Plantinga, L. C., Fink, N. E., Klag, M. J., Levey, A. S., Levin, N. W., Sadler, J. H., Kliger, A. and Powe, N. R. | Comparing the risk for death with peritoneal dialysis and hemodialysis in a national cohort of patients with chronic kidney disease | 2005 | CVD mortality not stratified by sex |
| 370 | Jamison, R. L., Hartigan, P., Kaufman, J. S., Goldfarb, D. S., Warren, S. R., Guarino, P. D. and Gaziano, J. M. | Effect of homocysteine lowering on mortality and vascular disease in advanced chronic kidney disease and end-stage renal disease: a randomized controlled trial | 2007 | CVD mortality not stratified by sex |
| 371 | Janda, K., Krzanowski, M., Dumnicka, P., Kusnierz-Cabala, B., Miarka, P. and Sulowicz, W. | Peritoneal solute transport rate as an independent risk factor for total and cardiovascular mortality in a population of peritoneal dialysis patients | 2014 | CVD mortality not stratified by sex |
| 372 | Janda, K., Krzanowski, M., Gajda, M., Dumnicka, P., Jasek, E., Fedak, D., Pietrzycka, A., Kuzniewski, M., Litwin, J. A. and Sulowicz, W. | Vascular effects of advanced glycation end-products: content of immunohistochemically detected AGEs in radial artery samples as a predictor for arterial calcification and cardiovascular risk in asymptomatic patients with chronic kidney disease | 2015 | CVD mortality not stratified by sex |
| 373 | Jaroszyński, A., Jaroszyńska, A., Zaborowski, T., Drelich-Zbroja, A., Zapolski, T. and Dabrowski, W. | Serum heat shock protein 27 levels predict cardiac mortality in hemodialysis patients 11 Medical and Health Sciences 1102 Cardiorespiratory Medicine and Haematology | 2018 | CVD mortality not stratified by sex |
| 374 | Jin, H., Shin, J. Y., Lee, S. H., Song, J. H., Kim, M. J. and Lee, S. W. | Abdominal obesity and mortality in continuous ambulatory peritoneal dialysis patients | 2015 | CVD mortality not stratified by sex |
| 375 | Johansson, M., Gao, S. A., Friberg, P., Annerstedt, M., Carlstrom, J., Ivarsson, T., Jensen, G., Ljungman, S., Mathillas, O., Nielsen, F. D. and Strombom, U. | Baroreflex effectiveness index and baroreflex sensitivity predict all-cause mortality and sudden death in hypertensive patients with chronic renal failure | 2007 | CVD mortality not stratified by sex |
| 376 | Johnson, D. W., Dent, H., Hawley, C. M., McDonald, S. P., Rosman, J. B., Brown, F. G., Bannister, K. and Wiggins, K. J. | Association of dialysis modality and cardiovascular mortality in incident dialysis patients | 2009 | CVD mortality not stratified by sex |
| 377 | Johnson, D. W., Wiggins, K. J., Armstrong, K. A., Campbell, S. B., Isbel, N. M. and Hawley, C. M. | Elevated white cell count at commencement of peritoneal dialysis predicts overall and cardiac mortality | 2005 | CVD mortality not stratified by sex |
| 378 | Joki, N., Hase, H., Kawano, Y., Nakamura, S., Nakajima, K., Hatta, T., Nishimura, S., Moroi, M., Nakagawa, S., Kasai, T., Kusuoka, H., Takeishi, Y., Momose, M., Takehana, K., Nanasato, M., Yoda, S., Nishina, H., Matsumoto, N. and Nishimura, T. | Myocardial perfusion imaging for predicting cardiac events in Japanese patients with advanced chronic kidney disease: 1-year interim report of the J-ACCESS 3 investigation | 2014 | CVD mortality not stratified by sex |
| 379 | Jung, H. Y., Kim, S. H., Jang, H. M., Lee, S., Kim, Y. S., Kang, S. W., Yang, C. W., Kim, N. H., Choi, J. Y., Cho, J. H., Kim, C. D., Park, S. H. and Kim, Y. L. | Individualized prediction of mortality using multiple inflammatory markers in patients on dialysis | 2018 | CVD mortality not stratified by sex |
| 380 | Jung, J. H., Chae, Y. J., Lee, D. H., Cho, Y. I., Ko, M. M., Park, S. K. and Kim, W. | Changes in whole blood viscosity during hemodialysis and mortality in patients with end-stage renal disease | 2017 | CVD mortality not stratified by sex |
| 381 | Kacso, I. M., Potra, A. R., Bondor, C. I., Moldovan, D., Rusu, C., Patiu, I. M., Racasan, S., Orasan, R., Vladutiu, D., Spanu, C., Rusu, A., Nita, C., Moldovan, R., Ghigolea, B. and Kacso, G. | Adiponectin predicts cardiovascular events in diabetes dialysis patients | 2015 | CVD mortality not stratified by sex |
| 382 | Kalantar-Zadeh, K., Block, G., Humphreys, M. H., McAllister, C. J. and Kopple, J. D. | A Low, Rather than a High, Total Plasma Homocysteine Is an Indicator of Poor Outcome in Hemodialysis Patients | 2004 | CVD mortality not stratified by sex |
| 383 | Kalantar-Zadeh, K., Kopple, J. D., Kilpatrick, R. D., McAllister, C. J., Shinaberger, C. S., Gjertson, D. W. and Greenland, S. | Association of morbid obesity and weight change over time with cardiovascular survival in hemodialysis population | 2005 | CVD mortality not stratified by sex |
| 384 | Kalantar-Zadeh, K., Regidor, D. L., Kovesdy, C. P., Van Wyck, D., Bunnapradist, S., Horwich, T. B. and Fonarow, G. C. | Fluid retention is associated with cardiovascular mortality in patients undergoing long-term hemodialysis | 2009 | CVD mortality not stratified by sex |
| 385 | Kalim, S., Clish, C. B., Wenger, J., Elmariah, S., Yeh, R. W., Deferio, J. J., Pierce, K., Deik, A., Gerszten, R. E., Thadhani, R. and Rhee, E. P. | A plasma long-chain acylcarnitine predicts cardiovascular mortality in incident dialysis patients | 2013 | CVD mortality not stratified by sex |
| 386 | Kalousová, M., Jáchymová, M., Muravská, A., Kuběna, A. A., Dusilová-Sulková, S., Tesař, V. and Zima, T. | Cys327Cys polymorphism of the PAPP-A gene (pregnancy associated plasma protein A) is related to mortality of long term hemodialysis patients | 2014 | CVD mortality not stratified by sex |
| 387 | Kalousová, M., Zima, T., Krane, V., März, W., Wanner, C., Tesař, V. and Drechsler, C. | Pregnancy-associated plasma protein A associates with cardiovascular events in diabetic hemodialysis patients | 2014 | CVD mortality not stratified by sex |
| 388 | Kamiura, N., Yamamoto, K., Okada, S., Sakai, M. and Fujimori, A. | Calcification of the thoracic aorta determined by three-dimensional computed tomography predicts cardiovascular complications in patients undergoing hemodialysis | 2014 | CVD mortality not stratified by sex |
| 389 | Kanbay, M., Afsar, B., Siriopol, D., Unal, H. U., Karaman, M., Saglam, M., Gezer, M., Tas, A., Eyileten, T., Guler, A. K., Aydin, I., Oguz, Y., Tarim, K., Covic, A. and Yilmaz, M. I. | Endostatin in chronic kidney disease: Associations with inflammation, vascular abnormalities, cardiovascular events and survival | 2016 | CVD mortality not stratified by sex |
| 390 | Kanbay, M., Yilmaz, M. I., Apetrii, M., Saglam, M., Yaman, H., Unal, H. U., Gok, M., Caglar, K., Oguz, Y., Yenicesu, M., Cetinkaya, H., Eyileten, T., Acikel, C., Vural, A. and Covic, A. | Relationship between serum magnesium levels and cardiovascular events in chronic kidney disease patients | 2012 | CVD mortality not stratified by sex |
| 391 | Kasama, S., Toyama, T., Sato, M., Sano, H., Ueda, T., Sasaki, T., Nakahara, T., Higuchi, T., Tsushima, Y. and Kurabayashi, M. | Prognostic value of myocardial perfusion single photon emission computed tomography for major adverse cardiac cerebrovascular and renal events in patients with chronic kidney disease: results from first year of follow-up of the Gunma-CKD SPECT multicenter study | 2016 | CVD mortality not stratified by sex |
| 392 | Kato, A., Takita, T., Furuhashi, M., Maruyama, Y., Kumagai, H. and Hishida, A. | Blood monocyte count is a predictor of total and cardiovascular mortality in hemodialysis patients | 2008 | CVD mortality not stratified by sex |
| 393 | Kato, A., Takita, T., Furuhashi, M., Maruyama, Y., Miyajima, H. and Kumagai, H. | Brachial-ankle pulse wave velocity and the cardio-ankle vascular index as a predictor of cardiovascular outcomes in patients on regular hemodialysis | 2012 | CVD mortality not stratified by sex |
| 394 | Katzenellenbogen, J. M., Sanfilippo, F. M., Hobbs, M. S., Briffa, T. G., Ridout, S. C., Knuiman, M. W., Dimer, L., Taylor, K. P., Thompson, P. L. and Thompson, S. C. | Aboriginal to non-Aboriginal differentials in 2-year outcomes following non-fatal first-ever acute MI persist after adjustment for comorbidity | 2012 | CVD mortality not stratified by sex |
| 395 | Kawaguchi, T., Tong, L., Robinson, B. M., Sen, A., Fukuhara, S., Kurokawa, K., Canaud, B., Lameire, N., Port, F. K. and Pisoni, R. L. | C-reactive protein and mortality in hemodialysis patients: the Dialysis Outcomes and Practice Patterns Study (DOPPS) | 2011 | CVD mortality not stratified by sex |
| 396 | Kaysen, G. A., Johansen, K. L., Chertow, G. M., Dalrymple, L. S., Kornak, J., Grimes, B., Dwyer, T., Chassy, A. W. and Fiehn, O. | Associations of Trimethylamine N-Oxide With Nutritional and Inflammatory Biomarkers and Cardiovascular Outcomes in Patients New to Dialysis | 2015 | CVD mortality not stratified by sex |
| 397 | Kazmi, W. H., Gilbertson, D. T., Obrador, G. T., Guo, H., Pereira, B. J., Collins, A. J. and Kausz, A. T. | Effect of comorbidity on the increased mortality associated with early initiation of dialysis | 2005 | CVD mortality not stratified by sex |
| 398 | Kerns, E. S., Kim, E. D., Meoni, L. A., Sozio, S. M., Jaar, B. G., Estrella, M. M., Parekh, R. S. and Bourjeily, G. | Obstructive Sleep Apnea Increases Sudden Cardiac Death in Incident Hemodialysis Patients | 2018 | CVD mortality not stratified by sex |
| 399 | Khilji, F., Baloch, M., Iqbal, J., Iqbal, Q. and Saleem, F. | Frequency, prognosis and risk factors among congestive heart failure in dialysis patients attending public hospitals of Quetta city, Pakistan | 2017 | CVD mortality not stratified by sex |
| 400 | Kim, C. S., Jin, D. C., Yun, Y. C., Bae, E. H., Ma, S. K. and Kim, S. W. | Relationship between serum uric acid and mortality among hemodialysis patients: Retrospective analysis of Korean end-stage renal disease registry data | 2017 | CVD mortality not stratified by sex |
| 401 | Kim, D., Shim, C. Y., Hong, G. R., Cho, I. J., Chang, H. J., Ha, J. W. and Chung, N. | Effect of End-Stage Renal Disease on Rate of Progression of Aortic Stenosis | 2016 | CVD mortality not stratified by sex |
| 402 | Kim, H. J., Lee, H., Kim, D. K., Oh, K. H., Kim, Y. S., Ahn, C., Han, J. S., Min, S. K., Min, S. I., Kim, H. C. and Joo, K. W. | Recurrent Vascular Access Dysfunction as a Novel Marker of Cardiovascular Outcome and Mortality in Hemodialysis Patients | 2016 | CVD mortality not stratified by sex |
| 403 | Kim, J. S. and Wen, Y. | A comparative study on mortality patterns among Koreans, Korean-Chinese and Chinese | 1999 | CVD mortality not stratified by sex |
| 404 | Kim, N. H., Kim, K. J., Choi, J., Lee, J., Bae, J. H., An, J. H., Kim, H. Y., Yoo, H. J., Seo, J. A., Kim, N. H., Choi, K. M., Baik, S. H. and Kim, S. G. | Dipeptidyl peptidase-4 inhibitor compared with sulfonylurea in combination with metformin: Cardiovascular and renal outcomes in a propensity-matched cohort study | 2019 | CVD mortality not stratified by sex |
| 405 | Kim, R. B., Morse, B. L., Djurdjev, O., Tang, M., Muirhead, N., Barrett, B., Holmes, D. T., Madore, F., Clase, C. M., Rigatto, C., Levin, A. and Can, P. I. | Advanced chronic kidney disease populations have elevated trimethylamine N-oxide levels associated with increased cardiovascular events | 2016 | CVD mortality not stratified by sex |
| 406 | Kim, S., Molnar, M. Z., Fonarow, G. C., Streja, E., Wang, J., Gillen, D. L., Mehrotra, R., Brunelli, S. M., Kovesdy, C. P., Kalantar-Zadeh, K. and Rhee, C. M. | Mean platelet volume and mortality risk in a national incident hemodialysis cohort | 2016 | CVD mortality not stratified by sex |
| 407 | Kim, S. J., Oh, H. J., Yoo, D. E., Shin, D. H., Lee, M. J., Kim, H. R., Park, J. T., Han, S. H., Yoo, T. H., Choi, K. H. and Kang, S. W. | Electrocardiographic left ventricular hypertrophy and outcome in hemodialysis patients | 2012 | CVD mortality not stratified by sex |
| 408 | Kim, T., Rhee, C. M., Streja, E., Soohoo, M., Obi, Y., Chou, J. A., Tortorici, A. R., Ravel, V. A., Kovesdy, C. P. and Kalantar-Zadeh, K. | Racial and Ethnic Differences in Mortality Associated with Serum Potassium in a Large Hemodialysis Cohort | 2017 | CVD mortality not stratified by sex |
| 409 | Kimata, N., Albert, J. M., Akiba, T., Yamazaki, S., Kawaguchi, Y., Fukuhara, S., Akizawa, T., Saito, A., Asano, Y., Kurokawa, K., Pisoni, R. L. and Port, F. K. | Association of mineral metabolism factors with all-cause and cardiovascular mortality in hemodialysis patients: The Japan dialysis outcomes and practice patterns study | 2007 | CVD mortality not stratified by sex |
| 410 | Kiran, V. R., Zhu, T. Y., Yip, T., Lui, S. L. and Lo, W. K. | Body mass index and mortality risk in Asian peritoneal dialysis patients in Hong Kong-impact of diabetes and cardiovascular disease status | 2014 | CVD mortality not stratified by sex |
| 411 | Klassen, P. S., Lowrie, E. G., Reddan, D. N., DeLong, E. R., Coladonato, J. A., Szczech, L. A., Lazarus, J. M. and Owen, W. F., Jr. | Association between pulse pressure and mortality in patients undergoing maintenance hemodialysis | 2002 | CVD mortality not stratified by sex |
| 412 | Kleine, C. E., Soohoo, M., Ranasinghe, O. N., Park, C., Marroquin, M. V., Obi, Y., Rhee, C. M., Moradi, H., Kovesdy, C. P., Kalantar-Zadeh, K. and Streja, E. | Association of Pre-End-Stage Renal Disease Hemoglobin with Early Dialysis Outcomes | 2018 | CVD mortality not stratified by sex |
| 413 | Ko, K. I., Park, K. S., Lee, M. J., Doh, F. M., Kim, C. H., Koo, H. M., Oh, H. J., Park, J. T., Han, S. H., Kang, S. W. and Yoo, T. H. | Increased dialysate MCP-1 is associated with cardiovascular mortality in peritoneal dialysis patients: a prospective observational study | 2014 | CVD mortality not stratified by sex |
| 414 | Koch, M., Kohnle, M., Trapp, R., Haastert, B., Rump, L. C. and Aker, S. | Comparable outcome of acute unplanned peritoneal dialysis and haemodialysis | 2012 | CVD mortality not stratified by sex |
| 415 | Kohara, M., Masuda, T., Shiizaki, K., Akimoto, T., Watanabe, Y., Honma, S., Sekiguchi, C., Miyazawa, Y., Kusano, E., Kanda, Y., Asano, Y., Kuro-O, M. and Nagata, D. | Association between circulating fibroblast growth factor 21 and mortality in end-stage renal disease | 2017 | CVD mortality not stratified by sex |
| 416 | Kollerits, B., Krane, V., Drechsler, C., Lamina, C., Marz, W., Ritz, E., Wanner, C. and Kronenberg, F. | Apolipoprotein A-IV concentrations and clinical outcomes in haemodialysis patients with type 2 diabetes mellitus--a post hoc analysis of the 4D Study | 2012 | CVD mortality not stratified by sex |
| 417 | Komatsu, M., Okazaki, M., Tsuchiya, K., Kawaguchi, H. and Nitta, K. | Aortic arch calcification predicts cardiovascular and all-cause mortality in maintenance hemodialysis patients | 2014 | CVD mortality not stratified by sex |
| 418 | Koo, H. M., Kim, C. H., Doh, F. M., Lee, M. J., Kim, E. J., Han, J. H., Han, J. S., Oh, H. J., Han, S. H., Yoo, T. H. and Kang, S. W. | The impact of low triiodothyronine levels on mortality is mediated by malnutrition and cardiac dysfunction in incident hemodialysis patients | 2013 | CVD mortality not stratified by sex |
| 419 | Kovesdy, C. P., Alrifai, A., Gosmanova, E. O., Lu, J. L., Canada, R. B., Wall, B. M., Hung, A. M., Molnar, M. Z. and Kalantar-Zadeh, K. | Age and Outcomes Associated with BP in Patients with Incident CKD | 2016 | CVD mortality not stratified by sex |
| 420 | Kovesdy, C. P., Lott, E. H., Lu, J. L., Malakauskas, S. M., Ma, J. Z., Molnar, M. Z. and Kalantar-Zadeh, K. | Hyponatremia, hypernatremia, and mortality in patients with chronic kidney disease with and without congestive heart failure | 2012 | CVD mortality not stratified by sex |
| 421 | Krane, V., Genser, B., Kleber, M. E., Drechsler, C., Marz, W., Delgado, G., Allolio, B., Wanner, C. and Fenske, W. | Copeptin associates with cause-specific mortality in patients with impaired renal function: results from the LURIC and the 4D study | 2017 | CVD mortality not stratified by sex |
| 422 | Krane, V., Krieter, D. H., Olschewski, M., Marz, W., Mann, J. F., Ritz, E. and Wanner, C. | Dialyzer membrane characteristics and outcome of patients with type 2 diabetes on maintenance hemodialysis | 2007 | CVD mortality not stratified by sex |
| 423 | Krane, V., Winkler, K., Drechsler, C., Lilienthal, J., März, W. and Wanner, C. | Association of LDL Cholesterol and Inflammation With Cardiovascular Events and Mortality in Hemodialysis Patients With Type 2 Diabetes Mellitus | 2009 | CVD mortality not stratified by sex |
| 424 | Krishnasamy, R., Isbel, N. M., Hawley, C. M., Pascoe, E. M., Burrage, M., Leano, R., Haluska, B. A., Marwick, T. H. and Stanton, T. | Left Ventricular Global Longitudinal Strain (GLS) Is a Superior Predictor of All-Cause and Cardiovascular Mortality When Compared to Ejection Fraction in Advanced Chronic Kidney Disease | 2015 | CVD mortality not stratified by sex |
| 425 | Krzanowski, M., Krzanowska, K., Dumnicka, P., Gajda, M., Woziwodzka, K., Fedak, D., Grodzicki, T., Litwin, J. A. and Sulowicz, W. | Elevated Circulating Osteoprotegerin Levels in the Plasma of Hemodialyzed Patients With Severe Artery Calcification | 2018 | CVD mortality not stratified by sex |
| 426 | Krzanowski, M., Krzanowska, K., Gajda, M., Dumnicka, P., Dziewierz, A., Woziwodzka, K., Litwin, J. A. and Sulowicz, W. | Pentraxin 3 as a new indicator of cardiovascular-related death in patients with advanced chronic kidney disease | 2017 | CVD mortality not stratified by sex |
| 427 | Kshirsagar, A. V., Craig, R. G., Moss, K. L., Beck, J. D., Offenbacher, S., Kotanko, P., Klemmer, P. J., Yoshino, M., Levin, N. W., Yip, J. K., Almas, K., Lupovici, E. M., Usvyat, L. A. and Falk, R. J. | Periodontal disease adversely affects the survival of patients with end-stage renal disease | 2009 | CVD mortality not stratified by sex |
| 428 | Kuo, H. L., Liu, Y. L., Liang, C. C., Chang, C. T., Wang, S. M., Liu, J. H., Lin, H. H., Wang, I. K., Yang, Y. F., Chou, C. Y. and Huang, C. C. | Prolonged QT interval is linked to all-cause and cardiac mortality in chronic peritoneal dialysis patients | 2017 | CVD mortality not stratified by sex |
| 429 | Kuo, K. L., Hung, S. C., Tseng, W. C., Tsai, M. T., Liu, J. S., Lin, M. H., Hsu, C. C. and Tarng, D. C. | Association of anemia and iron parameters with mortality among patients undergoing prevalent hemodialysis in Taiwan: The AIM-HD study | 2018 | CVD mortality not stratified by sex |
| 430 | Kuragano, T., Matsumura, O., Matsuda, A., Hara, T., Kiyomoto, H., Murata, T., Kitamura, K., Fujimoto, S., Hase, H., Joki, N., Fukatsu, A., Inoue, T., Itakura, I. and Nakanishi, T. | Association between hemoglobin variability, serum ferritin levels, and adverse events/mortality in maintenance hemodialysis patients | 2014 | CVD mortality not stratified by sex |
| 431 | Kuwahara, M., Hasumi, S., Mandai, S., Tanaka, T., Shikuma, S., Akita, W., Mori, Y. and Sasaki, S. | Rate of ankle-brachial index decline predicts cardiovascular mortality in hemodialysis patients | 2014 | CVD mortality not stratified by sex |
| 432 | Kuwahara, M., Takehara, E., Sasaki, Y., Azetsu, H., Kusaka, K., Shikuma, S. and Akita, W. | Effects of Cardiovascular Events on End-Stage Renal Disease and Mortality in Patients With Chronic Kidney Disease Before Dialysis | 2016 | CVD mortality not stratified by sex |
| 433 | Kuwamura, Y., Shoji, T., Okute, Y., Yamazaki, Y., Motoyama, K., Morioka, T., Mori, K., Fukumoto, S., Tsujimoto, Y., Shioi, A., Emoto, M. and Inaba, M. | Altered Serum n-6 Polyunsaturated Fatty Acid Profile and Risks of Mortality and Cardiovascular Events in a Cohort of Hemodialysis Patients | 2018 | CVD mortality not stratified by sex |
| 434 | Kuzniewski, M., Fedak, D., Dumnicka, P., Stepien, E., Kusnierz-Cabala, B., Cwynar, M. and Sulowicz, W. | Osteoprotegerin and osteoprotegerin/TRAIL ratio are associated with cardiovascular dysfunction and mortality among patients with renal failure | 2016 | CVD mortality not stratified by sex |
| 435 | Kyto, V., Sipila, J. and Rautava, P. | Gender and in-hospital mortality of ST-segment elevation myocardial infarction (from a multihospital nationwide registry study of 31,689 patients) | 2015 | CVD mortality not stratified by sex |
| 436 | Ladhani, M., Craig, J. C., Irving, M., Clayton, P. A. and Wong, G. | Obesity and the risk of cardiovascular and all-cause mortality in chronic kidney disease: A systematic review and meta-analysis | 2017 | CVD mortality not stratified by sex |
| 437 | Lahrach, H., Lebrazi, H., Saïle, R., Ghalim, N. and Ramdani, B. | Inflammation, cardiovascular risk and mortality among long term haemodialysis patients | 2012 | CVD mortality not stratified by sex |
| 438 | Lai, X., Zhang, A. H., Chen, S. Y., He, L., Su, C. Y., Fan, M. H. and Wang, T. | Outcomes of stage 1-5 chronic kidney disease in Mainland China | 2014 | CVD mortality not stratified by sex |
| 439 | Lajer, M., Tarnow, L., Jorsal, A., Teerlink, T., Parving, H. H. and Rossing, P. | Plasma concentration of asymmetric dimethylarginine (ADMA) predicts cardiovascular morbidity and mortality in type 1 diabetic patients with diabetic nephropathy | 2008 | CVD mortality not stratified by sex |
| 440 | Lam, C. S., Carson, P. E., Anand, I. S., Rector, T. S., Kuskowski, M., Komajda, M., McKelvie, R. S., McMurray, J. J., Zile, M. R., Massie, B. M. and et al. | Sex differences in clinical characteristics and outcomes in elderly patients with heart failure and preserved ejection fraction: the Irbesartan in Heart Failure with Preserved Ejection Fraction (I-PRESERVE) trial | 2012 | CVD mortality not stratified by sex |
| 441 | Langsford, D., Tang, M., Cheikh Hassan, H. I., Djurdjev, O., Sood, M. M. and Levin, A. | The Association between Biomarker Profiles, Etiology of Chronic Kidney Disease, and Mortality | 2017 | CVD mortality not stratified by sex |
| 442 | Lawson, C. A., Testani, J. M., Mamas, M., Damman, K., Jones, P. W., Teece, L. and Kadam, U. T. | Chronic kidney disease, worsening renal function and outcomes in a heart failure community setting: A UK national study | 2018 | CVD mortality not stratified by sex |
| 443 | Lee, M. J., Park, J. T., Han, S. H., Kim, Y. L., Kim, Y. S., Yang, C. W., Kim, N. H., Kang, S. W., Kim, H. J. and Yoo, T. H. | The atherogenic index of plasma and the risk of mortality in incident dialysis patients: Results from a nationwide prospective cohort in Korea | 2017 | CVD mortality not stratified by sex |
| 444 | Lee, M. J., Shin, D. H., Kim, S. J., Yoo, D. E., Ko, K. I., Koo, H. M., Kim, C. H., Doh, F. M., Oh, H. J., Park, J. T., Han, S. H., Yoo, T. H., Choi, K. H. and Kang, S. W. | Sagittal abdominal diameter is an independent predictor of all-cause and cardiovascular mortality in incident peritoneal dialysis patients | 2013 | CVD mortality not stratified by sex |
| 445 | Lee, S., Kang, E., Yoo, K. D., Choi, Y., Kim, D. K., Joo, K. W., Yang, S. H., Kim, Y. L., Kang, S. W., Yang, C. W., Kim, N. H., Kim, Y. S. and Lee, H. | Lower serum potassium associated with increased mortality in dialysis patients: A nationwide prospective observational cohort study in Korea | 2017 | CVD mortality not stratified by sex |
| 446 | Lee, T., Thamer, M., Zhang, Q., Zhang, Y. and Allon, M. | Reduced Cardiovascular Mortality Associated with Early Vascular Access Placement in Elderly Patients with Chronic Kidney Disease | 2016 | CVD mortality not stratified by sex |
| 447 | Lee, W. C., Fang, H. Y., Chen, H. C., Chen, C. J., Yang, C. H., Hang, C. L., Wu, C. J. and Fang, C. Y. | Anemia: A significant cardiovascular mortality risk after ST-segment elevation myocardial infarction complicated by the comorbidities of hypertension and kidney disease | 2017 | CVD mortality not stratified by sex |
| 448 | Lertdumrongluk, P., Rhee, C. M., Park, J., Lau, W. L., Moradi, H., Jing, J., Molnar, M. Z., Brunelli, S. M., Nissenson, A. R., Kovesdy, C. P. and Kalantar-Zadeh, K. | Association of serum phosphorus concentration with mortality in elderly and nonelderly hemodialysis patients | 2013 | CVD mortality not stratified by sex |
| 449 | Lesaffre, F., Wynckel, A., Nazeyrollas, P., Rieu, P. and Metz, D. | Echocardiography to predict adverse cardiac and vascular events in patients with severe chronic kidney disease (stage 4): a prospective study | 2013 | CVD mortality not stratified by sex |
| 450 | Levin, A., Rigatto, C., Brendan, B., Madore, F., Muirhead, N., Holmes, D., Clase, C. M., Tang, M. and Djurdjev, O. | Cohort profile: Canadian study of prediction of death, dialysis and interim cardiovascular events (CanPREDDICT) | 2013 | CVD mortality not stratified by sex |
| 451 | Li, C., Hu, D., Shi, X., Li, L., Yang, J., Song, L. and Ma, C. | A multicentre prospective evaluation of the impact of renal insufficiency on in-hospital and long-term mortality of patients with acute ST-elevation myocardial infarction | 2015 | CVD mortality not stratified by sex |
| 452 | Li, H., Lu, X., Xiong, R. and Wang, S. | High Neutrophil-to-Lymphocyte Ratio Predicts Cardiovascular Mortality in Chronic Hemodialysis Patients | 2017 | CVD mortality not stratified by sex |
| 453 | Li, T., Liu, J., An, S., Dai, Y. and Yu, Q. | Body mass index and mortality in patients on maintenance hemodialysis: A meta-analysis | 2014 | CVD mortality not stratified by sex |
| 454 | Li, W., Xu, R., Wang, Y., Shen, J., Li, Z., Yu, X. and Mao, H. | Association of body mass index and uncontrolled blood pressure with cardiovascular mortality in peritoneal dialysis patients | 2019 | CVD mortality not stratified by sex |
| 455 | Li, W. J., Chen, X. M., Nie, X. Y., Zhang, J., Cheng, Y. J., Lin, X. X. and Wu, S. H. | Cardiac troponin and C-reactive protein for predicting all-cause and cardiovascular mortality in patients with chronic kidney disease: a meta-analysis. Review | 2015 | CVD mortality not stratified by sex |
| 456 | Liabeuf, S., Barreto, D. V., Kretschmer, A., Barreto, F. C., Renard, C., Andrejak, M., Choukroun, G. and Massy, Z. | High circulating levels of large splice variants of tenascin-C is associated with mortality and cardiovascular disease in chronic kidney disease patients | 2011 | CVD mortality not stratified by sex |
| 457 | Liabeuf, S., Glorieux, G., Lenglet, A., Diouf, M., Schepers, E., Desjardins, L., Choukroun, G., Vanholder, R. and Massy, Z. A. | Does p-cresylglucuronide have the same impact on mortality as other protein-bound uremic toxins? | 2013 | CVD mortality not stratified by sex |
| 458 | Liabeuf, S., Lenglet, A., Desjardins, L., Neirynck, N., Glorieux, G., Lemke, H. D., Vanholder, R., Diouf, M., Choukroun, G. and Massy, Z. A. | Plasma beta-2 microglobulin is associated with cardiovascular disease in uremic patients | 2012 | CVD mortality not stratified by sex |
| 459 | Lim, C. C., Teo, B. W., Ong, P. G., Cheung, C. Y., Lim, S. C., Chow, K. Y., Meng, C. C., Lee, J., Tai, E. S., Wong, T. Y. and Sabanayagam, C. | Chronic kidney disease, cardiovascular disease and mortality: A prospective cohort study in a multi-ethnic Asian population | 2015 | CVD mortality not stratified by sex |
| 460 | Lima, H. N., Cabral, N. L., Franklin, J., Moro, C. H., Pecoits-Filho, R. and Goncalves, A. R. | Age dependent impact of estimated glomerular filtration rate on long-term survival after ischaemic stroke | 2012 | CVD mortality not stratified by sex |
| 461 | Lin, C., Zhang, Q., Zhang, H. and Lin, A. | Long-Term Effects of Low-Dose Spironolactone on Chronic Dialysis Patients: a Randomized Placebo-Controlled Study | 2016 | CVD mortality not stratified by sex |
| 462 | Lin, C. Y., Leu, J. G., Fang, Y. W. and Tsai, M. H. | Association of interleg difference of ankle brachial index with overall and cardiovascular mortality in chronic hemodialysis patients | 2015 | CVD mortality not stratified by sex |
| 463 | Lin, F. J., Zhang, X., Huang, L. S., Ji, G., Huang, H. D., Xie, Y., Jiang, G. R., Zhou, X. and Lu, W. | Impact of hemoglobin variability on cardiovascular mortality in maintenance hemodialysis patients | 2018 | CVD mortality not stratified by sex |
| 464 | Lin, J. H., Yen, T. H., Weng, C. H. and Huang, W. H. | Environmental NO2 level is associated with 2-year mortality in patients undergoing peritoneal dialysis | 2015 | CVD mortality not stratified by sex |
| 465 | Lin, M. Y., Cheng, L. J., Chiu, Y. W., Hsieh, H. M., Wu, P. H., Lin, Y. T., Wang, S. L., Jian, F. X., Hsu, C. C., Yang, S. A., Lee, H. L. and Hwang, S. J. | Effect of national pre-ESRD care program on expenditures and mortality in incident dialysis patients: A population-based study | 2018 | CVD mortality not stratified by sex |
| 466 | Lin, S. W., Weng, W. C., Huang, Y. H., Su, F. C., Peng, T. I., Chien, Y. Y., Wu, C. L., Lee, K. Y., Yu, Y. J., Zhu, J. X. and Huang, W. Y. | Association between renal dysfunction and 3-year mortality in patients with acute first-ever ischemic stroke | 2015 | CVD mortality not stratified by sex |
| 467 | Lin, T. H., Lai, W. T., Hsin, H. T., Li, A. H., Wang, C. L., Kuo, C. T., Hwang, J. J., Chiang, F. T. and Chang, S. C. | Effects of clopidogrel on mortality, cardiovascular and bleeding outcomes in patients with chronic kidney disease - data from Taiwan acute coronary syndrome full spectrum registry | 2013 | CVD mortality not stratified by sex |
| 468 | Lin, T. Y., Lim, P. S. and Hung, S. C. | Normal-weight obesity and clinical outcomes in nondiabetic chronic kidney disease patients: a cohort study | 2018 | CVD mortality not stratified by sex |
| 469 | Lin, T. Y., Peng, C. H., Hung, S. C. and Tarng, D. C. | Body composition is associated with clinical outcomes in patients with non-dialysis-dependent chronic kidney disease | 2018 | CVD mortality not stratified by sex |
| 470 | Lipsic, E., Asselbergs, F. W., van der Meer, P., Tio, R. A., Voors, A. A., van Gilst, W. H., Zijlstra, F. and van Veldhuisen, D. J. | Anaemia predicts cardiovascular events in patients with stable coronary artery disease | 2005 | CVD mortality not stratified by sex |
| 471 | Liu, J. H., Chen, C. C., Wang, S. M., Chou, C. Y., Liu, Y. L., Kuo, H. L., Lin, H. H., Wang, I. K., Yang, Y. F. and Huang, C. C. | Association between pulse pressure and 30-month all-cause mortality in peritoneal dialysis patients | 2008 | CVD mortality not stratified by sex |
| 472 | Liu, S. H., Li, Y. J., Wu, H. H., Lee, C. C., Lin, C. Y., Weng, C. H., Chen, Y. C., Chang, M. Y., Hsu, H. H., Fang, J. T., Hung, C. C., Yang, C. W. and Tian, Y. C. | High-sensitivity C-reactive protein predicts mortality and technique failure in peritoneal dialysis patients | 2014 | CVD mortality not stratified by sex |
| 473 | Liu, T., Liang, K. V., Rosenbaum, A., Stephenson, R., Pike, F., Weissfeld, L. and Unruh, M. L. | Peripheral vascular disease severity impacts health outcomes and health-related quality of life in maintenance hemodialysis patients in the HEMO Study | 2012 | CVD mortality not stratified by sex |
| 474 | Liu, X., Guo, Q., Feng, X., Wang, J., Wu, J., Mao, H., Huang, F., Yu, X. and Yang, X. | Alkaline phosphatase and mortality in patients on peritoneal dialysis | 2014 | CVD mortality not stratified by sex |
| 475 | Liu, Y., Lee, W. C., Cheng, B. C., Li, L. C., Lee, C. H., Chang, W. X. and Chen, J. B. | Association between the achievement of target range CKD-MBD markers and mortality in prevalent hemodialysis patients in Taiwan by Using the Kidney Disease: Improving Global Outcomes Clinical Guidelines | 2016 | CVD mortality not stratified by sex |
| 476 | Liu, Y., Zhu, J. G., Cheng, B. C., Liao, S. C., Lee, C. H., Chang, W. X. and Chen, J. B. | An association between time-varying serum alkaline phosphatase concentrations and mortality rate in patients undergoing peritoneal dialysis: a five-year cohort study | 2017 | CVD mortality not stratified by sex |
| 477 | Liu, Y. L., Liu, J. H., Wang, I. K., Ju, S. W., Yu, T. M., Chen, I. R., Liu, Y. C., Huang, C. M., Lin, S. Y., Chang, C. T. and Huang, C. C. | Association of inflammatory cytokines with mortality in peritoneal dialysis patients | 2017 | CVD mortality not stratified by sex |
| 478 | Liu, Y. W., Su, C. T., Sung, J. M., Wang, S. P., Su, Y. R., Yang, C. S., Tsai, L. M., Chen, J. H. and Tsai, W. C. | Association of left ventricular longitudinal strain with mortality among stable hemodialysis patients with preserved left ventricular ejection fraction | 2013 | CVD mortality not stratified by sex |
| 479 | Liu, Y. W., Tseng, C. C., Su, C. T., Chang, Y. T., Chen, J. Y., Chen, L. Y., Tsai, L. M., Chen, J. H., Wang, M. C. and Tsai, W. C. | The prognostic value of left ventricular global peak systolic longitudinal strain in chronic peritoneal dialysis patients | 2014 | CVD mortality not stratified by sex |
| 480 | Lofman, I., Szummer, K., Dahlstrom, U., Jernberg, T. and Lund, L. H. | Associations with and prognostic impact of chronic kidney disease in heart failure with preserved, mid-range, and reduced ejection fraction | 2017 | CVD mortality not stratified by sex |
| 481 | London, G. M., Guerin, A. P., Marchais, S. J., Metivier, F., Pannier, B. and Adda, H. | Arterial media calcification in end-stage renal disease: impact on all-cause and cardiovascular mortality | 2003 | CVD mortality not stratified by sex |
| 482 | Longenecker, J. C., Klag, M. J., Marcovina, S. M., Liu, Y. M., Jaar, B. G., Powe, N. R., Fink, N. E., Levey, A. S. and Coresh, J. | High lipoprotein(a) levels and small apolipoprotein(a) size prospectively predict cardiovascular events in dialysis patients | 2005 | CVD mortality not stratified by sex |
| 483 | Lorenz, G., Schmalenberg, M., Kemmner, S., Haller, B., Steubl, D., Pham, D., Schreiegg, A., Bachmann, Q., Schmidt, A., Haderer, S., Huber, M., Angermann, S., Gunthner, R., Braunisch, M., Hauser, C., Reichelt, A. L., Matschkal, J., Suttmann, Y., Moog, P., Stock, K., Kuchle, C., Thurmel, K., Renders, L., Bauer, A., Baumann, M., Heemann, U., Luppa, P. B. and Schmaderer, C. | Mortality prediction in stable hemodialysis patients is refined by YKL-40, a 40-kDa glycoprotein associated with inflammation | 2018 | CVD mortality not stratified by sex |
| 484 | Lorenzen, J., David, S., Bahlmann, F. H., Groot, K., Bahlmann, E., Kielstein, J. T., Haller, H. and Fliser, D. | Endothelial progenitor cells and cardiovascular events in patients with chronic kidney disease - A prospective follow-up study | 2010 | CVD mortality not stratified by sex |
| 485 | Losito, A., Del Vecchio, L., Del Rosso, G. and Locatelli, F. | Postdialysis Hypertension: Associated Factors, Patient Profiles, and Cardiovascular Mortality | 2016 | CVD mortality not stratified by sex |
| 486 | Losito, A., Del Vecchio, L., Del Rosso, G. and Malandra, R. | Blood pressure and cardiovascular mortality in dialysis patients with left ventricular systolic dysfunction | 2014 | CVD mortality not stratified by sex |
| 487 | Losito, A., Kalidas, K., Santoni, S., Errico, R. and Jeffery, S. | Association of the -159C/T polymorphism of the endotoxin receptor (CD14) with carotid artery disease and cardiovascular mortality in dialysis patients | 2005 | CVD mortality not stratified by sex |
| 488 | Lowbeer, C., Stenvinkel, P., Pecoits-Filho, R., Heimburger, O., Lindholm, B., Gustafsson, S. A. and Seeberger, A. | Elevated cardiac troponin T in predialysis patients is associated with inflammation and predicts mortality | 2003 | CVD mortality not stratified by sex |
| 489 | Lu, C. L., Leu, J. G., Liu, W. C., Zheng, C. M., Lin, Y. F., Shyu, J. F., Wu, C. C. and Lu, K. C. | Endothelial progenitor cells predict long-term mortality in hemodialysis patients | 2016 | CVD mortality not stratified by sex |
| 490 | Lu, J., Zhu, M., Liu, S., Zhu, M., Pang, H., Lin, X., Ni, Z., Qian, J., Cai, H. and Zhang, W. | The relationship between survival rate and intradialytic blood pressure changes in maintenance hemodialysis patients | 2017 | CVD mortality not stratified by sex |
| 491 | Lu, T. M., Chung, M. Y., Lin, C. C., Hsu, C. P. and Lin, S. J. | Asymmetric dimethylarginine and clinical outcomes in chronic kidney disease | 2011 | CVD mortality not stratified by sex |
| 492 | Lu, W., Pang, W. F., Jin, L., Li, H., Chow, K. M., Kwan, B. C. H., Leung, C. B., Li, P. K. T. and Szeto, C. C. | Peritoneal protein clearance predicts mortality in peritoneal dialysis patients | 2019 | CVD mortality not stratified by sex |
| 493 | Lu, X., Wang, S., Zhang, G., Xiong, R. and Li, H. | High Neutrophil-to-Lymphocyte Ratio is a Significant Predictor of Cardiovascular and All-Cause Mortality in Patients Undergoing Peritoneal Dialysis | 2018 | CVD mortality not stratified by sex |
| 494 | Lukowsky, L. R., Kheifets, L., Arah, O. A., Nissenson, A. R. and Kalantar-Zadeh, K. | Patterns and predictors of early mortality in incident hemodialysis patients: new insights | 2012 | CVD mortality not stratified by sex |
| 495 | Luo, Q., Xia, X., Li, B., Lin, Z., Yu, X. and Huang, F. | Serum uric acid and cardiovascular mortality in chronic kidney disease: A meta-analysis | 2019 | CVD mortality not stratified by sex |
| 496 | Luo, Y., Li, X., Li, J., Wang, X., Xu, Y., Qiao, Y., Hu, D. and Ma, Y. | Peripheral arterial disease, chronic kidney disease, and mortality: the Chinese Ankle Brachial Index Cohort Study | 2010 | CVD mortality not stratified by sex |
| 497 | Luthi, J. C., Flanders, W. D., Burnier, M., Burnand, B. and McClellan, W. M. | Anemia and chronic kidney disease are associated with poor outcomes in heart failure patients | 2006 | CVD mortality not stratified by sex |
| 498 | Madero, M., Sarnak, M. J., Wang, X., Sceppa, C. C., Greene, T., Beck, G. J., Kusek, J. W., Collins, A. J., Levey, A. S. and Menon, V. | Body mass index and mortality in CKD | 2007 | CVD mortality not stratified by sex |
| 499 | Maduell, F., Moreso, F., Pons, M., Ramos, R., Mora-Macia, J., Carreras, J., Soler, J., Torres, F., Campistol, J. M. and Martinez-Castelao, A. | High-efficiency postdilution online hemodiafiltration reduces all-cause mortality in hemodialysis patients | 2013 | CVD mortality not stratified by sex |
| 500 | Maduell, F., Varas, J., Ramos, R., Martin-Malo, A., Perez-Garcia, R., Berdud, I., Moreso, F., Canaud, B., Stuard, S., Gauly, A., Aljama, P. and Merello, J. I. | Hemodiafiltration Reduces All-Cause and Cardiovascular Mortality in Incident Hemodialysis Patients: A Propensity-Matched Cohort Study | 2017 | CVD mortality not stratified by sex |
| 501 | Mahmoodi, B. K., Matsushita, K., Woodward, M., Blankestijn, P. J., Cirillo, M., Ohkubo, T., Rossing, P., Sarnak, M. J., Stengel, B., Yamagishi, K., Yamashita, K., Zhang, L., Coresh, J., de Jong, P. E. and Astor, B. C. | Associations of kidney disease measures with mortality and end-stage renal disease in individuals with and without hypertension: a meta-analysis | 2012 | CVD mortality not stratified by sex |
| 502 | Mallamaci, F., Zoccali, C., Tripepi, G., Fermo, I., Benedetto, F. A., Cataliotti, A., Bellanuova, I., Malatino, L. S. and Soldarini, A. | Hyperhomocysteinemia predicts cardiovascular outcomes in hemodialysis patients | 2002 | CVD mortality not stratified by sex |
| 503 | Mann, J. F., Lonn, E. M., Yi, Q., Gerstein, H. C., Hoogwerf, B. J., Pogue, J., Bosch, J., Dagenais, G. R. and Yusuf, S. | Effects of vitamin E on cardiovascular outcomes in people with mild-to-moderate renal insufficiency: results of the HOPE study | 2004 | CVD mortality not stratified by sex |
| 504 | Marcais, C., Maucort-Boulch, D., Drai, J., Dantony, E., Carlier, M. C., Blond, E., Genet, L., Kuentz, F., Lataillade, D., Legrand, E., Moreau-Gaudry, X., Jean, G. and Fouque, D. | Circulating Klotho Associates With Cardiovascular Morbidity and Mortality During Hemodialysis | 2017 | CVD mortality not stratified by sex |
| 505 | Mark, P. B., Doyle, A., Blyth, K. G., Patel, R. K., Weir, R. A., Steedman, T., Foster, J. E., Dargie, H. J. and Jardine, A. G. | Vascular function assessed with cardiovascular magnetic resonance predicts survival in patients with advanced chronic kidney disease | 2008 | CVD mortality not stratified by sex |
| 506 | Marouga, A., Dalamaga, M., Kastania, A. N., Kroupis, C., Lagiou, M., Saounatsou, K., Dimas, K. and Vlahakos, D. V. | Circulating resistin is a significant predictor of mortality independently from cardiovascular comorbidities in elderly, non-diabetic subjects with chronic kidney disease | 2016 | CVD mortality not stratified by sex |
| 507 | Martin, R. S. S., Martin, L. C., Franco, R. J. S., Barretti, P., Caramori, J. C. T., Castro, J. H., Antunes, A. A., Zanati-Basan, S. G., Matsubara, B. B. and Martins, A. S. | Ventricular hypertrophy and cardiovascular mortality in hemodialysis patients with low educational level | 2012 | CVD mortality not stratified by sex |
| 508 | Maruyama, Y., Taniguchi, M., Kazama, J. J., Yokoyama, K., Hosoya, T., Yokoo, T., Shigematsu, T., Iseki, K. and Tsubakihara, Y. | A higher serum alkaline phosphatase is associated with the incidence of hip fracture and mortality among patients receiving hemodialysis in Japan | 2014 | CVD mortality not stratified by sex |
| 509 | Maruyama, Y., Yokoyama, K., Yokoo, T., Shigematsu, T., Iseki, K. and Tsubakihara, Y. | The Different Association between Serum Ferritin and Mortality in Hemodialysis and Peritoneal Dialysis Patients Using Japanese Nationwide Dialysis Registry | 2015 | CVD mortality not stratified by sex |
| 510 | März, W., Genser, B., Drechsler, C., Krane, V., Grammer, T. B., Ritz, E., Stojakovic, T., Scharnagl, H., Winkler, K., Holme, I. and et al. | Atorvastatin and low-density lipoprotein cholesterol in type 2 diabetes mellitus patients on hemodialysis | 2011 | CVD mortality not stratified by sex |
| 511 | Mason, N. A., Bailie, G. R., Satayathum, S., Bragg-Gresham, J. L., Akiba, T., Akizawa, T., Combe, C., Rayner, H. C., Saito, A., Gillespie, B. W. and et al. | HMG-coenzyme a reductase inhibitor use is associated with mortality reduction in hemodialysis patients | 2005 | CVD mortality not stratified by sex |
| 512 | Masuda, T., Murata, M., Honma, S., Iwazu, Y., Sasaki, N., Ogura, M., Onishi, A., Ando, Y., Muto, S., Shimada, K., Kario, K., Kusano, E. and Asano, Y. | Sleep-disordered breathing predicts cardiovascular events and mortality in hemodialysis patients | 2011 | CVD mortality not stratified by sex |
| 513 | Matias, P. J., Azevedo, A., Laranjinha, I., Navarro, D., Mendes, M., Ferreira, C., Amaral, T., Jorge, C., Aires, I., Gil, C. and Ferreira, A. | Lower serum magnesium is associated with cardiovascular risk factors and mortality in haemodialysis patients | 2014 | CVD mortality not stratified by sex |
| 514 | Matschkal, J., Mayer, C. C., Sarafidis, P. A., Lorenz, G., Braunisch, M. C., Guenthner, R., Angermann, S., Steubl, D., Kemmner, S., Bachmann, Q., Hauser, C., Nerl, L., Baumann, M., Mann, J. F., Moog, P., Kuechle, C., Renders, L., Heemann, U., Wassertheurer, S. and Schmaderer, C. | Comparison of 24-hour and Office Pulse Wave Velocity for Prediction of Mortality in Hemodialysis Patients | 2019 | CVD mortality not stratified by sex |
| 515 | Matsubara, K., Suliman, M. E., Qureshi, A. R., Axelsson, J., Martola, L., Heimburger, O., Barany, P., Stenvinkel, P. and Lindholm, B. | Bone mineral density in end-stage renal disease patients: association with wasting, cardiovascular disease and mortality | 2008 | CVD mortality not stratified by sex |
| 516 | Matsumoto, Y., Mori, Y., Kageyama, S., Arihara, K., Sugiyama, T., Ohmura, H., Yakushigawa, T., Sugiyama, H., Shimada, Y., Nojima, Y. and et al. | Spironolactone reduces cardiovascular and cerebrovascular morbidity and mortality in hemodialysis patients | 2014 | CVD mortality not stratified by sex |
| 517 | Matsuo, S., Nakajima, K., Takeishi, Y. and Nishimura, T. | Prognostic value of normal stress myocardial perfusion imaging and ventricular function in Japanese patients with chronic kidney disease: a study based on the J-ACCESS-3 database | 2018 | CVD mortality not stratified by sex |
| 518 | Matsushita, K., Coresh, J., Sang, Y., Chalmers, J., Fox, C., Guallar, E., Jafar, T., Jassal, S. K., Landman, G. W., Muntner, P., Roderick, P., Sairenchi, T., Schottker, B., Shankar, A., Shlipak, M., Tonelli, M., Townend, J., van Zuilen, A., Yamagishi, K., Yamashita, K., Gansevoort, R., Sarnak, M., Warnock, D. G., Woodward, M., Arnlov, J. and Consortium, C. K. D. P. | Estimated glomerular filtration rate and albuminuria for prediction of cardiovascular outcomes: a collaborative meta-analysis of individual participant data | 2015 | CVD mortality not stratified by sex |
| 519 | Matsushita, K., Sang, Y., Ballew, S. H., Astor, B. C., Hoogeveen, R. C., Solomon, S. D., Ballantyne, C. M., Woodward, M. and Coresh, J. | Cardiac and kidney markers for cardiovascular prediction in individuals with chronic kidney disease: the Atherosclerosis Risk in Communities study | 2014 | CVD mortality not stratified by sex |
| 520 | Mavrakanas, T. A., Sniderman, A. D., Barre, P. E. and Alam, A. | Serial versus single troponin measurements for the prediction of cardiovascular events and mortality in stable chronic haemodialysis patients | 2018 | CVD mortality not stratified by sex |
| 521 | Mayer, C. C., Matschkal, J., Sarafidis, P. A., Hagmair, S., Lorenz, G., Angermann, S., Braunisch, M. C., Baumann, M., Heemann, U., Wassertheurer, S. and Schmaderer, C. | Association of ambulatory blood pressure with all-cause and cardiovascular mortality in hemodialysis patients: Effects of heart failure and atrial fibrillation | 2018 | CVD mortality not stratified by sex |
| 522 | McClellan, W. M., Flanders, W. D., Langston, R. D., Jurkovitz, C. and Presley, R. | Anemia and renal insufficiency are independent risk factors for death among patients with congestive heart failure admitted to community hospitals: a population-based study | 2002 | CVD mortality not stratified by sex |
| 523 | McGovern, A. P., Rusholme, B., Jones, S., van Vlymen, J. N., Liyanage, H., Gallagher, H., Tomson, C. R., Khunti, K., Harris, K. and de Lusignan, S. | Association of chronic kidney disease (CKD) and failure to monitor renal function with adverse outcomes in people with diabetes: a primary care cohort study | 2013 | CVD mortality not stratified by sex |
| 524 | McMullan, C. J., Bakris, G. L., Phillips, R. A. and Forman, J. P. | Association of BP variability with mortality among African Americans with CKD | 2013 | CVD mortality not stratified by sex |
| 525 | McMullan, C. J., Lambers Heerspink, H. J., Parving, H. H., Dwyer, J. P., Forman, J. P. and de Zeeuw, D. | Visit-to-visit variability in blood pressure and kidney and cardiovascular outcomes in patients with type 2 diabetes and nephropathy: a post hoc analysis from the RENAAL study and the Irbesartan Diabetic Nephropathy Trial | 2014 | CVD mortality not stratified by sex |
| 526 | Mehrotra, R., Kermah, D. A., Salusky, I. B., Wolf, M. S., Thadhani, R. I., Chiu, Y. W., Martins, D., Adler, S. G. and Norris, K. C. | Chronic kidney disease, hypovitaminosis D, and mortality in the United States | 2009 | CVD mortality not stratified by sex |
| 527 | Mehta, N. N., Matthews, G. J., Krishnamoorthy, P., Shah, R., McLaughlin, C., Patel, P., Budoff, M., Chen, J., Wolman, M., Go, A., He, J., Kanetsky, P. A., Master, S. R., Rader, D. J., Raj, D., Gadegbeku, C. A., Shah, R., Schreiber, M., Fischer, M. J., Townsend, R. R., Kusek, J., Feldman, H. I., Foulkes, A. S. and Reilly, M. P. | Higher plasma CXCL12 levels predict incident myocardial infarction and death in chronic kidney disease: Findings fromthe chronic renal insufficiency cohort study | 2014 | CVD mortality not stratified by sex |
| 528 | Melloni, C., Cornel, J. H., Hafley, G., Neely, M. L., Clemmensen, P., Zamoryakhin, D., Prabhakaran, D., White, H. D., Fox, K. A., Ohman, E. M., Armstrong, P. W. and Roe, M. T. | Impact of chronic kidney disease on long-term ischemic and bleeding outcomes in medically managed patients with acute coronary syndromes: Insights from the TRILOGY ACS Trial | 2016 | CVD mortality not stratified by sex |
| 529 | Menon, V., Greene, T., Pereira, A. A., Wang, X., Beck, G. J., Kusek, J. W., Collins, A. J., Levey, A. S. and Sarnak, M. J. | Glycosylated hemoglobin and mortality in patients with nondiabetic chronic kidney disease | 2005 | CVD mortality not stratified by sex |
| 530 | Menon, V., Greene, T., Wang, X., Pereira, A. A., Marcovina, S. M., Beck, G. J., Kusek, J. W., Collins, A. J., Levey, A. S. and Sarnak, M. J. | C-reactive protein and albumin as predictors of all-cause and cardiovascular mortality in chronic kidney disease | 2005 | CVD mortality not stratified by sex |
| 531 | Menon, V., Sarnak, M. J., Greene, T., Wang, X., Pereira, A. A., Beck, G. J., Kusek, J. W., Selhub, J., Collins, A. J., Levey, A. S. and et al. | Relationship between homocysteine and mortality in chronic kidney disease | 2006 | CVD mortality not stratified by sex |
| 532 | Mercadal, L., Franck, J. E., Metzger, M., Urena Torres, P., de Cornelissen, F., Edet, S., Bechade, C., Vigneau, C., Drueke, T., Jacquelinet, C. and Stengel, B. | Hemodiafiltration Versus Hemodialysis and Survival in Patients With ESRD: The French Renal Epidemiology and Information Network (REIN) Registry | 2016 | CVD mortality not stratified by sex |
| 533 | Meuwese, C. L., Dekker, F. W., Lindholm, B., Qureshi, A. R., Heimburger, O., Barany, P., Stenvinkel, P. and Carrero, J. J. | Baseline levels and trimestral variation of triiodothyronine and thyroxine and their association with mortality in maintenance hemodialysis patients | 2012 | CVD mortality not stratified by sex |
| 534 | Mezue, K., Goyal, A., Pressman, G. S., Horrow, J. C. and Rangaswami, J. | Blood Pressure Variability Predicts Adverse Events and Cardiovascular Outcomes in Chronic Kidney Disease: A Post-Hoc Analysis of the SPRINT Trial | 2017 | CVD mortality not stratified by sex |
| 535 | Mielniczuk, L. M., Pfeffer, M. A., Lewis, E. F., Blazing, M. A., de Lemos, J. A., Mohanavelu, S., Rouleau, J., Fox, K., Pedersen, T. R. and Califf, R. M. | Acute decline in renal function, inflammation, and cardiovascular risk after an acute coronary syndrome | 2009 | CVD mortality not stratified by sex |
| 536 | Minami, Y., Kajimoto, K., Sato, N., Hagiwara, N. and Takano, T. | End-stage renal disease patients on chronic maintenance hemodialysis in a hospitalized acute heart failure cohort: Prevalence, clinical characteristics, therapeutic options, and mortality | 2016 | CVD mortality not stratified by sex |
| 537 | Miskulin, D. C., Tangri, N., Bandeen-Roche, K., Zhou, J., McDermott, A., Meyer, K. B., Ephraim, P. L., Michels, W. M., Jaar, B. G., Crews, D. C., Scialla, J. J., Sozio, S. M., Shafi, T., Wu, A. W., Cook, C. and Boulware, L. E. | Intravenous iron exposure and mortality in patients on hemodialysis | 2014 | CVD mortality not stratified by sex |
| 538 | Mitsuma, W., Matsubara, T., Hatada, K., Imai, S., Saito, N., Shimada, H. and Miyazaki, S. | Clinical characteristics of hemodialysis patients with atrial fibrillation: The RAKUEN (Registry of atrial fibrillation in chronic kidney disease under hemodialysis from Niigata) study | 2016 | CVD mortality not stratified by sex |
| 539 | Miura, M., Shiba, N., Nochioka, K., Takada, T., Takahashi, J., Kohno, H. and Shimokawa, H. | Urinary albumin excretion in heart failure with preserved ejection fraction: an interim analysis of the CHART 2 study | 2012 | CVD mortality not stratified by sex |
| 540 | Miura, S., Yoshihisa, A., Takiguchi, M., Shimizu, T., Nakamura, Y., Yamauchi, H., Iwaya, S., Owada, T., Miyata, M., Abe, S., Sato, T., Suzuki, S., Oikawa, M., Yamaki, T., Sugimoto, K., Kunii, H., Nakazato, K., Suzuki, H., Saitoh, S. I. and Takeishi, Y. | Association of Hypocalcemia with Mortality in Hospitalized Patients with Heart Failure and Chronic Kidney Disease | 2015 | CVD mortality not stratified by sex |
| 541 | Molnar, M. Z., Mehrotra, R., Duong, U., Kovesdy, C. P. and Kalantar-Zadeh, K. | Association of hemoglobin and survival in peritoneal dialysis patients | 2011 | CVD mortality not stratified by sex |
| 542 | Moradi, H., Streja, E., Kashyap, M. L., Vaziri, N. D., Fonarow, G. C. and Kalantar-Zadeh, K. | Elevated high-density lipoprotein cholesterol and cardiovascular mortality in maintenance hemodialysis patients | 2014 | CVD mortality not stratified by sex |
| 543 | Moroi, M., Tamaki, N., Nishimura, M., Haze, K., Nishimura, T., Kusano, E., Akiba, T., Sugimoto, T., Hase, H., Hara, K., Nakata, T., Kumita, S., Nagai, Y., Hashimoto, A., Momose, M., Miyakoda, K., Hasebe, N. and Kikuchi, K. | Association between abnormal myocardial fatty acid metabolism and cardiac-derived death among patients undergoing hemodialysis: Results from a cohort study in japan | 2013 | CVD mortality not stratified by sex |
| 544 | Mourad, A., Khoshdel, A., Carney, S., Gillies, A., Jones, B., Nanra, R. and Trevillian, P. | Haemodialysis-unresponsive blood pressure: cardiovascular mortality predictor? | 2005 | CVD mortality not stratified by sex |
| 545 | Movilli, E., Camerini, C., Gaggia, P., Zubani, R., Feller, P., Poiatti, P., Pola, A., Carli, O. and Cancarini, G. | Magnitude of end-dialysis overweight is associated with all-cause and cardiovascular mortality: a 3-year prospective study | 2013 | CVD mortality not stratified by sex |
| 546 | Muiesan, M. L., Ambrosioni, E., Costa, F. V., Leonetti, G., Pessina, A. C., Salvetti, M., Trimarco, B., Volpe, M., Pontremoli, R., Deferrari, G. and Rosei, E. A. | Sex differences in hypertension-related renal and cardiovascular diseases in Italy: the I-DEMAND study | 2012 | CVD mortality not stratified by sex |
| 547 | Nagashima, M., Hagiwara, N., Koyanagi, R., Yamaguchi, J., Takagi, A., Kawada-Watanabe, E., Shiga, T. and Ogawa, H. | Chronic kidney disease and long-term outcomes of myocardial infarction | 2013 | CVD mortality not stratified by sex |
| 548 | Nagata, M., Ninomiya, T., Kiyohara, Y., Murakami, Y., Irie, F., Sairenchi, T., Miura, K., Okamura, T. and Ueshima, H. | Prediction of cardiovascular disease mortality by proteinuria and reduced kidney function: Pooled analysis of 39,000 individuals from 7 cohort studies in Japan | 2013 | CVD mortality not stratified by sex |
| 549 | Naiman, N., Cheung, A. K. and Goldfarb-Rumyantzev, A. S. | Familiality of cardiovascular mortality in end-stage renal disease patients | 2009 | CVD mortality not stratified by sex |
| 550 | Nakagawa, N., Matsuki, M., Yao, N., Hirayama, T., Ishida, H., Kikuchi, K. and Hasebe, N. | Impact of metabolic disturbances and malnutrition-inflammation on 6-year mortality in Japanese patients undergoing hemodialysis | 2015 | CVD mortality not stratified by sex |
| 551 | Nakamura, K., Okamura, T., Hayakawa, T., Kadowaki, T., Kita, Y., Ohnishi, H., Saitoh, S., Sakata, K., Okayama, A. and Ueshima, H. | Chronic kidney disease is a risk factor for cardiovascular death in a community-based population in Japan: NIPPON DATA90 | 2006 | CVD mortality not stratified by sex |
| 552 | Nakamura, S., Nakata, H., Yoshihara, F., Kamide, K., Horio, T., Nakahama, H. and Kawano, Y. | Effect of early nephrology referral on the initiation of hemodialysis and survival in patients with chronic kidney disease and cardiovascular diseases | 2007 | CVD mortality not stratified by sex |
| 553 | Nakamura, S., Ogata, C., Aihara, N., Sasaki, O., Yoshihara, F., Nakahama, H., Inenaga, T., Kimura, G. and Kawano, Y. | QTc dispersion in haemodialysis patients with cardiac complications | 2005 | CVD mortality not stratified by sex |
| 554 | Nakayama, M., Sato, T., Miyazaki, M., Matsushima, M., Sato, H., Taguma, Y. and Ito, S. | Increased risk of cardiovascular events and mortality among non-diabetic chronic kidney disease patients with hypertensive nephropathy: the Gonryo study | 2011 | CVD mortality not stratified by sex |
| 555 | Nakayama, M., Sato, T., Sato, H., Yamaguchi, Y., Obara, K., Kurihara, I., Sato, K., Hotta, O., Seino, J., Miyata, M., Takeuchi, K., Nakayama, K., Matsushima, M., Otaka, T., Kinoshita, Y., Taguma, Y. and Ito, S. | Different clinical outcomes for cardiovascular events and mortality in chronic kidney disease according to underlying renal disease: the Gonryo study | 2010 | CVD mortality not stratified by sex |
| 556 | Nakayama, M., Ura, Y., Nagata, M., Okada, Y., Sumida, Y., Nishida, K., Ikeda, H. and Kaizu, Y. | Carotid artery calcification at the initiation of hemodialysis is a risk factor for cardiovascular events in patients with end-stage renal disease: a cohort study | 2011 | CVD mortality not stratified by sex |
| 557 | Naruse, H., Ishii, J., Takahashi, H., Kitagawa, F., Okuyama, R., Kawai, H., Muramatsu, T., Harada, M., Yamada, A., Motoyama, S., Matsui, S., Hayashi, M., Sarai, M., Watanabe, E., Izawa, H. and Ozaki, Y. | Prognostic value of combination of plasma D-dimer concentration and estimated glomerular filtration rate in predicting long-term mortality of patients with stable coronary artery disease | 2017 | CVD mortality not stratified by sex |
| 558 | Nauta, S. T., van Domburg, R. T., Nuis, R. J., Akkerhuis, M. and Deckers, J. W. | Decline in 20-year mortality after myocardial infarction in patients with chronic kidney disease: evolution from the prethrombolysis to the percutaneous coronary intervention era | 2013 | CVD mortality not stratified by sex |
| 559 | Navaneethan, S. D. and Beddhu, S. | Associations of serum uric acid with cardiovascular events and mortality in moderate chronic kidney disease | 2009 | CVD mortality not stratified by sex |
| 560 | Navaneethan, S. D., Schold, J. D., Arrigain, S., Kirwan, J. P. and Nally, J. V. | Body mass index and causes of death in chronic kidney disease | 2016 | CVD mortality not stratified by sex |
| 561 | Navaneethan, S. D., Schold, J. D., Jolly, S. E., Arrigain, S., Blum, M. F., Winkelmayer, W. C. and Nally, J. V., Jr. | Blood pressure parameters are associated with all-cause and cause-specific mortality in chronic kidney disease | 2017 | CVD mortality not stratified by sex |
| 562 | Naves-Diaz, M., Passlick-Deetjen, J., Guinsburg, A., Marelli, C., Fernandez-Martin, J. L., Rodriguez-Puyol, D. and Cannata-Andia, J. B. | Calcium, phosphorus, PTH and death rates in a large sample of dialysis patients from Latin America. The CORES Study | 2011 | CVD mortality not stratified by sex |
| 563 | Nemcsik, J., Cseprekal, O., Egresits, J., Kielstein, J., Kumpers, P., Lukasz, A., Tabak, A., Marton, A., Nemeth, Z. K., Jarai, Z., Godina, G., Sallai, L., Farkas, K., Kiss, I. and Tisler, A. | The role of laser Doppler flowmetry tests, serum angiopoietin-2, asymmetric and symmetric dimethylarginine to predict outcome in chronic kidney disease | 2017 | CVD mortality not stratified by sex |
| 564 | Ng, Y. H., Meyer, K. B., Kusek, J. W., Yan, G., Rocco, M. V., Kimmel, P. L., Benz, R. L., Beddhu, S., Dwyer, J. T., Toto, R. D., Eknoyan, G. and Unruh, M. L. | Hemodialysis timing, survival, and cardiovascular outcomes in the Hemodialysis (HEMO) Study | 2006 | CVD mortality not stratified by sex |
| 565 | Nishikawa, K., Takahashi, K., Yamada, R., Kinaga, T., Masato, M. and Yamamoto, M. | Influence of chronic kidney disease on hospitalization, chronic dialysis, and mortality in Japanese men: a longitudinal analysis | 2017 | CVD mortality not stratified by sex |
| 566 | Nishimura, M., Tokoro, T., Takatani, T., Sato, N., Hashimoto, T., Kobayashi, H. and Ono, T. | Circulating Aminoterminal Propeptide of Type III Procollagen as a Biomarker of Cardiovascular Events in Patients Undergoing Hemodialysis | 2019 | CVD mortality not stratified by sex |
| 567 | Nishizawa, Y., Koyama, H. and Inaba, M. | AGEs and cardiovascular diseases in patients with end-stage renal diseases | 2012 | CVD mortality not stratified by sex |
| 568 | Noce, A., Canale, M. P., Capria, A., Rovella, V., Tesauro, M., Splendiani, G., Annicchiarico-Petruzzelli, M., Manzuoli, M., Simonetti, G. and Di Daniele, N. | Coronary artery calcifications predict long term cardiovascular events in non diabetic Caucasian hemodialysis patients | 2015 | CVD mortality not stratified by sex |
| 569 | Norris, K., Bourgoigne, J., Gassman, J., Hebert, L., Middleton, J., Phillips, R. A., Randall, O., Rostand, S., Sherer, S., Toto, R. D. and et al. | Cardiovascular outcomes in the African American Study of Kidney Disease and Hypertension (AASK) Trial | 2006 | CVD mortality not stratified by sex |
| 570 | Ntr | To what extent does high dose hemodiafiltration compared to high flux hemodialysis reduce risk of death in end stage kidney disease patients | 2018 | CVD mortality not stratified by sex |
| 571 | Nube, M. J., Peters, S. A. E., Blankestijn, P. J., Canaud, B., Davenport, A., Grooteman, M. P. C., Asci, G., Locatelli, F., Maduell, F., Morena, M., Ok, E., Torres, F. and Bots, M. L. | Mortality reduction by post-dilution online-haemodiafiltration: a cause-specific analysis | 2017 | CVD mortality not stratified by sex |
| 572 | Obermayr, R. P., Temml, C., Gutjahr, G., Kainz, A., Klauser-Braun, R., Fugger, R. and Oberbauer, R. | Body mass index modifies the risk of cardiovascular death in proteinuric chronic kidney disease | 2009 | CVD mortality not stratified by sex |
| 573 | Obi, Y., Hamano, T., Wada, A. and Tsubakihara, Y. | Vitamin D receptor activator use and cause-specific death among dialysis patients: A nationwide cohort study using coarsened exact matching | 2017 | CVD mortality not stratified by sex |
| 574 | Obi, Y., Nguyen, D. V., Streja, E., Rivara, M. B., Rhee, C. M., Lau, W. L., Chen, Y., Kovesdy, C. P., Mehrotra, R. and Kalantar-Zadeh, K. | Development and Validation of a Novel Laboratory-Specific Correction Equation for Total Serum Calcium and Its Association With Mortality Among Hemodialysis Patients | 2017 | CVD mortality not stratified by sex |
| 575 | Obi, Y., Park, C., Soohoo, M., Sumida, K., Hamano, T., Rhee, C. M., Kovesdy, C. P., Kalantar-Zadeh, K. and Streja, E. | Association of Pre-ESRD Serum Calcium With Post-ESRD Mortality Among Incident ESRD Patients: A Cohort Study | 2018 | CVD mortality not stratified by sex |
| 576 | Obokata, M., Negishi, K., Sunaga, H., Ishida, H., Ito, K., Ogawa, T., Iso, T., Ando, Y. and Kurabayashi, M. | Association Between Circulating Ketone Bodies and Worse Outcomes in Hemodialysis Patients | 2017 | CVD mortality not stratified by sex |
| 577 | Odutayo, A., Wong, C. X., Hsiao, A. J., Hopewell, S., Altman, D. G. and Emdin, C. A. | Atrial fibrillation and risks of cardiovascular disease, renal disease, and death: systematic review and meta-analysis | 2016 | CVD mortality not stratified by sex |
| 578 | Ogata, H., Kumasawa, J., Fukuma, S., Mizobuchi, M., Kinugasa, E., Fukagawa, M., Fukuhara, S. and Akizawa, T. | The cardiothoracic ratio and all-cause and cardiovascular disease mortality in patients undergoing maintenance hemodialysis: results of the MBD-5D study | 2017 | CVD mortality not stratified by sex |
| 579 | Ohtake, T., Ishioka, K., Honda, K., Oka, M., Maesato, K., Mano, T., Ikee, R., Moriya, H., Hidaka, S. and Kobayashi, S. | Impact of coronary artery calcification in hemodialysis patients: Risk factors and associations with prognosis | 2010 | CVD mortality not stratified by sex |
| 580 | Ok, E. S., Asci, G., Toz, H., Ritz, E., Kircelli, F., Sever, M. S., Ozkahya, M., Sipahi, S., Dheir, H., Bozkurt, D. and et al. | Glycated hemoglobin predicts overall and cardiovascular mortality in non-diabetic hemodialysis patients | 2014 | CVD mortality not stratified by sex |
| 581 | Okada, T., Nakao, T., Matsumoto, H., Shino, T., Nagaoka, Y., Tomaru, R. and Wada, T. | Association between markers of glycemic control, cardiovascular complications and survival in type 2 diabetic patients with end-stage renal disease | 2007 | CVD mortality not stratified by sex |
| 582 | Omae, K., Ogawa, T., Yoshikawa, M., Sakura, H. and Nitta, K. | Use of Beta-Blockers on Maintenance Dialysis Patients and Ischemic Cerebral and Cardiovascular Deaths: An Examination Using Propensity Score | 2018 | CVD mortality not stratified by sex |
| 583 | Omersa, D., Lainscak, M., Erzen, I. and Farkas, J. | Mortality and readmissions in heart failure: an analysis of 36,824 elderly patients from the Slovenian national hospitalization database | 2016 | CVD mortality not stratified by sex |
| 584 | Ono, K., Tsuchida, A., Kawai, H., Matsuo, H., Wakamatsu, R., Maezawa, A., Yano, S., Kawada, T. and Nojima, Y. | Ankle-brachial blood pressure index predicts all-cause and cardiovascular mortality in hemodialysis patients | 2003 | CVD mortality not stratified by sex |
| 585 | Onuigbo, M., Onuigbo, N., Bellasi, A., Russo, D. and Di Iorio, B. R. | Penultimate pulse wave velocity, better than baseline pulse wave velocity, predicted mortality in Italian ESRD cohort study - a case for daily hemodialysis for ESRD patients with accelerated pulse wave velocity changes | 2013 | CVD mortality not stratified by sex |
| 586 | Opelz, G. and Dohler, B. | Association of HLA mismatch with death with a functioning graft after kidney transplantation: a collaborative transplant study report | 2012 | CVD mortality not stratified by sex |
| 587 | O'Seaghdha, C. M., Tin, A., Yang, Q., Katz, R., Liu, Y., Harris, T., Astor, B., Coresh, J., Fox, C. S., Kao, W. H. and Shlipak, M. G. | Association of a cystatin C gene variant with cystatin C levels, CKD, and risk of incident cardiovascular disease and mortality | 2014 | CVD mortality not stratified by sex |
| 588 | O'Shaughnessy, M. M., Liu, S., Montez-Rath, M. E., Lafayette, R. A. and Winkelmayer, W. C. | Cause of kidney disease and cardiovascular events in a national cohort of US patients with end-stage renal disease on dialysis: A retrospective analysis | 2019 | CVD mortality not stratified by sex |
| 589 | Otani-Takei, N., Masuda, T., Akimoto, T., Honma, S., Watanabe, Y., Shiizaki, K., Miki, T., Kusano, E., Asano, Y., Kuro-O, M. and Nagata, D. | Association between Serum Soluble Klotho Levels and Mortality in Chronic Hemodialysis Patients | 2015 | CVD mortality not stratified by sex |
| 590 | Otsubo, S., Kitamura, M., Wakaume, T., Yajima, A., Ishihara, M., Takasaki, M., Ueda, S., Sugimoto, H., Otsubo, K., Kimata, N., Akiba, T. and Nitta, K. | Association of peripheral artery disease and long-term mortality in hemodialysis patients | 2012 | CVD mortality not stratified by sex |
| 591 | Ou, S. M., Chen, Y. T., Hung, S. C., Shih, C. J., Lin, C. H., Chiang, C. K. and Tarng, D. C. | Association of estimated glomerular filtration rate with all-cause and cardiovascular mortality: The role of malnutrition-inflammation-cachexia syndrome | 2016 | CVD mortality not stratified by sex |
| 592 | Ovbiagele, B. | Chronic kidney disease and risk of death during hospitalization for stroke | 2011 | CVD mortality not stratified by sex |
| 593 | Owaki, A., Inaguma, D., Tanaka, A., Shinjo, H., Inaba, S. and Kurata, K. | Evaluation of the Relationship between the Serum Alkaline Phosphatase Level at Dialysis Initiation and All-Cause Mortality: A Multicenter, Prospective Study | 2017 | CVD mortality not stratified by sex |
| 594 | Palau, V., Riera, M., Duran, X., Valdivielso, J. M., Betriu, A., Fernandez, E., Pascual, J. and Soler, M. J. | Circulating ADAMs are associated with renal and cardiovascular outcomes in chronic kidney disease patients | 2018 | CVD mortality not stratified by sex |
| 595 | Palmer, S. C., Ruospo, M., Wong, G., Craig, J. C., Petruzzi, M., De Benedittis, M., Ford, P., Johnson, D. W., Tonelli, M., Natale, P., Saglimbene, V., Pellegrini, F., Celia, E., Gelfman, R., Leal, M. R., Torok, M., Stroumza, P., Bednarek-Skublewska, A., Dulawa, J., Frantzen, L., Ferrari, J. N., Del Castillo, D., Bernat, A. G., Hegbrant, J., Wollheim, C., Gargano, L., Bots, C. P., Strippoli, G. F. and Investigators, O. D. S. | Dental Health and Mortality in People With End-Stage Kidney Disease Treated With Hemodialysis: A Multinational Cohort Study | 2015 | CVD mortality not stratified by sex |
| 596 | Palomino, H. L., Rifkin, D. E., Anderson, C., Criqui, M. H., Whooley, M. A. and Ix, J. H. | 24-hour urine phosphorus excretion and mortality and cardiovascular events | 2013 | CVD mortality not stratified by sex |
| 597 | Pan, Y., Jing, J., Chen, W., Wang, Y. and He, Y. | Association between impaired renal function and stroke outcome in patients with versus without atrial fibrillation | 2018 | CVD mortality not stratified by sex |
| 598 | Panaghiu, L., Veisa, G., Covic, A., Alexa, I. D., Arsenescu, C. and Covic, M. | Risk of sudden death in patients with chronic renal failure and hemodialysis | 2004 | CVD mortality not stratified by sex |
| 599 | Pang, P. S., Teerlink, J. R., Voors, A. A., Ponikowski, P., Greenberg, B. H., Filippatos, G., Felker, G. M., Davison, B. A., Cotter, G., Kriger, J. and et al. | Use of High-Sensitivity Troponin T to Identify Patients With Acute Heart Failure at Lower Risk for Adverse Outcomes. An Exploratory Analysis From the RELAX-AHF Trial | 2016 | CVD mortality not stratified by sex |
| 600 | Paniagua, R., Amato, D., Vonesh, E., Correa-Rotter, R., Ramos, A., Moran, J. and Mujais, S. | Effects of increased peritoneal clearances on mortality rates in peritoneal dialysis: aDEMEX, a prospective, randomized, controlled trial | 2002 | CVD mortality not stratified by sex |
| 601 | Panichi, V., Bigazzi, R., Paoletti, S., Mantuano, E., Beati, S., Marchetti, V., Bernabini, G., Grazi, G., Giusti, R., Rosati, A., Migliori, M., Betti, G., Pasquariello, A., Panicucci, E., Barsotti, G. and Bellasi, A. | Impact of calcium, phosphate, PTH abnormalities and management on mortality in hemodialysis: Results from the RISCAVID study | 2010 | CVD mortality not stratified by sex |
| 602 | Panichi, V., Rizza, G. M., Paoletti, S., Bigazzi, R., Aloisi, M., Barsotti, G., Rindi, P., Donati, G., Antonelli, A., Panicucci, E., Tripepi, G., Tetta, C. and Palla, R. | Chronic inflammation and mortality in haemodialysis: effect of different renal replacement therapies. Results from the RISCAVID study | 2008 | CVD mortality not stratified by sex |
| 603 | Papademetriou, V., Zaheer, M., Doumas, M., Lovato, L., Applegate, W. B., Tsioufis, C., Mottle, A., Punthakee, Z. and Cushman, W. C. | Cardiovascular Outcomes in Action to Control Cardiovascular Risk in Diabetes: impact of Blood Pressure Level and Presence of Kidney Disease | 2016 | CVD mortality not stratified by sex |
| 604 | Park, C. H., Kang, E. W., Park, J. T., Han, S. H., Yoo, T. H., Kang, S. W. and Chang, T. I. | Association of serum lipid levels over time with survival in incident peritoneal dialysis patients | 2017 | CVD mortality not stratified by sex |
| 605 | Park, J., Rhee, C. M., Sim, J. J., Kim, Y. L., Ricks, J., Streja, E., Vashistha, T., Tolouian, R., Kovesdy, C. P. and Kalantar-Zadeh, K. | A comparative effectiveness research study of the change in blood pressure during hemodialysis treatment and survival | 2013 | CVD mortality not stratified by sex |
| 606 | Park, J. T., Yoo, T. H., Kim, J. K., Oh, H. J., Kim, S. J., Yoo, D. E., Lee, M. J., Shin, D. H., Han, S. H., Han, D. S. and Kang, S. W. | Leptin/adiponectin ratio is an independent predictor of mortality in nondiabetic peritoneal dialysis patients | 2013 | CVD mortality not stratified by sex |
| 607 | Park, W. Y., Koh, E. S., Kim, S. H., Kim, Y. O., Jin, D. C., Song, H. C., Choi, E. J., Kim, Y. L., Kim, Y. S., Kang, S. W., Kim, N. H., Yang, C. W. and Kim, Y. K. | Serum Gamma-Glutamyltransferase Levels Predict Clinical Outcomes in Hemodialysis Patients | 2015 | CVD mortality not stratified by sex |
| 608 | Patel, K. K., Shah, S. Y., Arrigain, S., Jolly, S., Schold, J. D., Navaneethan, S. D., Griffin, B. P., Nally, J. V. and Desai, M. Y. | Characteristics and Outcomes of Patients With Aortic Stenosis and Chronic Kidney Disease | 2019 | CVD mortality not stratified by sex |
| 609 | Patel, R. K., Jardine, A. G., Mark, P. B., Cunningham, A. F., Steedman, T., Powell, J. R., McQuarrie, E. P., Stevens, K. K., Dargie, H. J. and Jardine, A. G. | Association of left atrial volume with mortality among ESRD patients with left ventricular hypertrophy referred for kidney transplantation | 2010 | CVD mortality not stratified by sex |
| 610 | Pei, J., Tang, W., Li, L. X., Su, C. Y. and Wang, T. | Heart rate variability predicts mortality in peritoneal dialysis patients | 2015 | CVD mortality not stratified by sex |
| 611 | Pena de la Vega, L., Miller, R. S., Benda, M. M., Grill, D. E., Johnson, M. G., McCarthy, J. T. and McBane, R. D., 2nd | Association of heparin-dependent antibodies and adverse outcomes in hemodialysis patients: a population-based study | 2005 | CVD mortality not stratified by sex |
| 612 | Peng, F., Li, Z., Yi, C., Guo, Q., Yang, R., Long, H., Huang, F., Yu, X. and Yang, X. | Platelet index levels and cardiovascular mortality in incident peritoneal dialysis patients: a cohort study | 2017 | CVD mortality not stratified by sex |
| 613 | Peng, F., Li, Z., Zhong, Z., Luo, Q., Guo, Q., Huang, F., Yu, X. and Yang, X. | An increasing of red blood cell distribution width was associated with cardiovascular mortality in patients on peritoneal dialysis | 2014 | CVD mortality not stratified by sex |
| 614 | Peng, Y., Chen, F., Huang, F. Y., Xia, T. L., Huang, B. T., Chai, H., Wang, P. J., Zuo, Z. L., Liu, W., Zhang, C., Gui, Y. Y., Chen, M. and Huang, D. J. | Body Composition and Mortality in Coronary Artery Disease With Mild Renal Insufficiency in Chinese Patients | 2017 | CVD mortality not stratified by sex |
| 615 | Peralta, C. A., Lee, A., Odden, M. C., Lopez, L., Zeki Al Hazzouri, A., Neuhaus, J. and Haan, M. N. | Association between chronic kidney disease detected using creatinine and cystatin C and death and cardiovascular events in elderly Mexican Americans: the Sacramento Area Latino Study on Aging | 2013 | CVD mortality not stratified by sex |
| 616 | Perkins, R. M., Tang, X., Bengier, A. C., Kirchner, H. L. and Bucaloiu, I. D. | Variability in estimated glomerular filtration rate is an independent risk factor for death among patients with stage 3 chronic kidney disease | 2012 | CVD mortality not stratified by sex |
| 617 | Perkovic, V., Levin, A., Wheeler, D., Koitka-Weber, A., Mattheus, M., George, J., Von Eynatten, M. and Wanner, C. | Effects of empagliflozin on cardiovascular outcomes across kdigo risk categories: results from the EMPA-REG outcome® trial | 2017 | CVD mortality not stratified by sex |
| 618 | Perry, R. J., Griffiths, W., Dextraze, P., Solomon, R. J. and Trebbin, W. M. | Elevated nicotine levels in patients undergoing hemodialysis. A role in cardiovascular mortality and morbidity? | 1984 | CVD mortality not stratified by sex |
| 619 | Peters, S. A., Bots, M. L., Canaud, B., Davenport, A., Grooteman, M. P., Kircelli, F., Locatelli, F., Maduell, F., Morena, M., Nubé, M. J. and et al. | Haemodiafiltration and mortality in end-stage kidney disease patients: a pooled individual participant data analysis from four randomized controlled trials | 2016 | CVD mortality not stratified by sex |
| 620 | Petreski, T., Bevc, S., Ekart, R. and Hojs, R. | Hyperuricemia and long-term survival in patients with chronic kidney disease undergoing hemodialysis | 2017 | CVD mortality not stratified by sex |
| 621 | Petreski, T., Ekart, R., Hojs, R. and Bevc, S. | Asymptomatic hyperuricemia and cardiovascular mortality in patients with chronic kidney disease who progress to hemodialysis | 2019 | CVD mortality not stratified by sex |
| 622 | Petrović, D. and Stojimirović, B. | Cardiovascular morbidity and mortality in patients treated with hemodialysis - Epidemiological analysis | 2008 | CVD mortality not stratified by sex |
| 623 | Petrovic, D. and Stojimirovic, B. B. | Cardiac troponins: outcome predictors in hemodialysis patients | 2009 | CVD mortality not stratified by sex |
| 624 | Pfeffer, M. A., Burdmann, E. A., Chen, C. Y., Cooper, M. E., de Zeeuw, D., Eckardt, K. U., Feyzi, J. M., Ivanovich, P., Kewalramani, R., Levey, A. S. and et al. | A trial of darbepoetin alfa in type 2 diabetes and chronic kidney disease | 2009 | CVD mortality not stratified by sex |
| 625 | Plantinga, L. C., Fink, N. E., Finkelstein, F. O., Powe, N. R. and Jaar, B. G. | Association of peritoneal dialysis clinic size with clinical outcomes | 2009 | CVD mortality not stratified by sex |
| 626 | Poesen, R., Viaene, L., Verbeke, K., Augustijns, P., Bammens, B., Claes, K., Kuypers, D., Evenepoel, P. and Meijers, B. | Cardiovascular disease relates to intestinal uptake of p-cresol in patients with chronic kidney disease | 2014 | CVD mortality not stratified by sex |
| 627 | Port, F. K., Wolfe, R. A., Hulbert-Shearon, T. E., McCullough, K. P., Ashby, V. B. and Held, P. J. | High dialysis dose is associated with lower mortality among women but not among men | 2004 | CVD mortality not stratified by sex |
| 628 | Porter, A., Fischer, M. J., Wang, X., Brooks, D., Bruce, M., Charleston, J., Cleveland, W. H., Dowie, D., Faulkner, M., Gassman, J., Hiremath, L., Kendrick, C., Kusek, J. W., Norris, K. C., Thornley-Brown, D., Greene, T. and Lash, J. P. | Quality of life and outcomes in African Americans with CKD | 2014 | CVD mortality not stratified by sex |
| 629 | Postorino, M., Marino, C., Tripepi, G. and Zoccali, C. | Gammaglutamyltransferase in ESRD as a predictor of all-cause and cardiovascular mortality: another facet of oxidative stress burden | 2008 | CVD mortality not stratified by sex |
| 630 | Postorino, M., Marino, C., Tripepi, G. and Zoccali, C. | Abdominal obesity and all-cause and cardiovascular mortality in end-stage renal disease | 2009 | CVD mortality not stratified by sex |
| 631 | Postorino, M., Marino, C., Tripepi, G. and Zoccali, C. | Abdominal obesity modifies the risk of hypertriglyceridemia for all-cause and cardiovascular mortality in hemodialysis patients | 2011 | CVD mortality not stratified by sex |
| 632 | Poulikakos, D., Hnatkova, K., Banerjee, D. and Malik, M. | Association of QRS-T angle and heart rate variability with major cardiac events and mortality in hemodialysis patients | 2018 | CVD mortality not stratified by sex |
| 633 | Pressman, G. S., Seetha Rammohan, H. R., Romero-Corral, A., Fumo, P., Figueredo, V. M. and Gorcsan, J., 3rd | Echocardiographic strain and mortality in Black Americans with end-stage renal disease on hemodialysis | 2015 | CVD mortality not stratified by sex |
| 634 | Proietti, M., Raparelli, V., Laroche, C., Dan, G. A., Janion, M., Popescu, R., Sinagra, G., Vijgen, J., Boriani, G., Maggioni, A. P., Tavazzi, L. and Lip, G. Y. H. | Adverse outcomes in patients with atrial fibrillation and peripheral arterial disease: A report from the EURObservational research programme pilot survey on atrial fibrillation | 2017 | CVD mortality not stratified by sex |
| 635 | Pun, P. H., Horton, J. R. and Middleton, J. P. | Dialysate calcium concentration and the risk of sudden cardiac arrest in hemodialysis patients | 2013 | CVD mortality not stratified by sex |
| 636 | Pun, P. H., Smarz, T. R., Honeycutt, E. F., Shaw, L. K., Al-Khatib, S. M. and Middleton, J. P. | Chronic kidney disease is associated with increased risk of sudden cardiac death among patients with coronary artery disease | 2009 | CVD mortality not stratified by sex |
| 637 | Quiroga, B., Verdalles, U., Reque, J., Garcia de Vinuesa, S., Goicoechea, M. and Luno, J. | Cardiovascular events and mortality in chronic kidney disease (stages I-IV) | 2013 | CVD mortality not stratified by sex |
| 638 | Quiroga, B., Villaverde, M., Abad, S., Vega, A., Reque, J. and Lopez-Gomez, J. M. | Diastolic dysfunction and high levels of new cardiac biomarkers as risk factors for cardiovascular events and mortality in hemodialysis patients | 2013 | CVD mortality not stratified by sex |
| 639 | Racki, S., Zaputovic, L., Mavric, Z., Vujicic, B. and Dvornik, S. | C-reactive protein is a strong predictor of mortality in hemodialysis patients | 2006 | CVD mortality not stratified by sex |
| 640 | Rahman, M., Hsu, J. Y., Desai, N., Hsu, C. Y., Anderson, A. H., Appel, L. J., Chen, J., Cohen, D. L., Drawz, P. E., He, J., Qiang, P., Ricardo, A. C., Steigerwalt, S., Weir, M. R., Wright, J. T., Zhang, X. and Townsend, R. R. | Central blood pressure and cardiovascular outcomes in chronic kidney disease | 2018 | CVD mortality not stratified by sex |
| 641 | Raikou, V. D. and Kyriaki, D. | Mortality and low serum bicarbonate level in patients on hemodiafiltration versus peritoneal dialysis | 2018 | CVD mortality not stratified by sex |
| 642 | Rakhit, D. J., Marwick, T. H., Armstrong, K. A., Johnson, D. W., Leano, R. and Isbel, N. M. | Effect of aggressive risk factor modification on cardiac events and myocardial ischaemia in patients with chronic kidney disease | 2006 | CVD mortality not stratified by sex |
| 643 | Raman, M., Green, D., Middleton, R. J. and Kalra, P. A. | Comparing the impact of older age on outcome in chronic kidney disease of different etiologies: a prospective cohort study | 2018 | CVD mortality not stratified by sex |
| 644 | Rambod, M., Heine, G. H., Seiler, S., Dominic, E. A., Rogacev, K. S., Dwivedi, R., Ramezani, A., Wing, M. R., Amdur, R. L., Fliser, D. and Raj, D. S. | Association of vascular endothelial factors with cardiovascular outcome and mortality in chronic kidney disease patients: a 4-year cohort study | 2014 | CVD mortality not stratified by sex |
| 645 | Refaat, H., Sany, D., Mohab, A. and Ezzat, H. | Comparing Dialysis Modality and Cardiovascular Mortality in Patients on Hemodialysis and Peritoneal Dialysis | 2016 | CVD mortality not stratified by sex |
| 646 | Rhee, C. M., Kovesdy, C. P., Ravel, V. A., Streja, E., Brunelli, S. M., Soohoo, M., Sumida, K., Molnar, M. Z., Brent, G. A., Nguyen, D. V. and Kalantar-Zadeh, K. | Association of Glycemic Status During Progression of Chronic Kidney Disease With Early Dialysis Mortality in Patients With Diabetes | 2017 | CVD mortality not stratified by sex |
| 647 | Rhee, J. J., Zheng, Y., Montez-Rath, M. E., Chang, T. I. and Winkelmayer, W. C. | Associations of Glycemic Control With Cardiovascular Outcomes Among US Hemodialysis Patients With Diabetes Mellitus | 2017 | CVD mortality not stratified by sex |
| 648 | Ricardo, A. C., Athavale, A., Chen, J., Hampole, H., Garside, D., Marucha, P. and Lash, J. P. | Periodontal disease, chronic kidney disease and mortality: results from the third National Health and Nutrition Examination Survey | 2015 | CVD mortality not stratified by sex |
| 649 | Ricardo, A. C., Goh, V., Chen, J., Cedillo-Couvert, E., Kapella, M., Prasad, B., Parvathaneni, S., Knutson, K. and Lash, J. P. | Association of Sleep Duration, Symptoms, and Disorders with Mortality in Adults with Chronic Kidney Disease | 2017 | CVD mortality not stratified by sex |
| 650 | Ricks, J., Molnar, M. Z., Kovesdy, C. P., Shah, A., Nissenson, A. R., Williams, M. and Kalantar-Zadeh, K. | Glycemic control and cardiovascular mortality in hemodialysis patients with diabetes: A 6-year cohort study | 2012 | CVD mortality not stratified by sex |
| 651 | Ridker, P. M., MacFadyen, J. G., Glynn, R. J., Koenig, W., Libby, P., Everett, B. M., Lefkowitz, M., Thuren, T. and Cornel, J. H. | Inhibition of Interleukin-1beta by Canakinumab and Cardiovascular Outcomes in Patients With Chronic Kidney Disease | 2018 | CVD mortality not stratified by sex |
| 652 | Ritchie, C., Ekundayo, O. J., Muchimba, M., Campbell, R. C., Frank, S. J., Liu, B., Aban, I. B. and Ahmed, A. | Effects of diabetes mellitus in patients with heart failure and chronic kidney disease: a propensity-matched study of multimorbidity in chronic heart failure | 2009 | CVD mortality not stratified by sex |
| 653 | Ritchie, J., Assi, L. K., Burmeister, A., Hoefield, R., Cockwell, P. and Kalra, P. A. | Association of Serum Ig Free Light Chains with Mortality and ESRD among Patients with Nondialysis-Dependent CKD | 2015 | CVD mortality not stratified by sex |
| 654 | Roberts, J. L. | Analysis and outcome of 1063 patients trained for home hemodialysis | 1976 | CVD mortality not stratified by sex |
| 655 | Roberts, M. A., Srivastava, P. M., Macmillan, N., Hare, D. L., Ratnaike, S., Sikaris, K. and Ierino, F. L. | B-type natriuretic peptides strongly predict mortality in patients who are treated with long-term dialysis | 2008 | CVD mortality not stratified by sex |
| 656 | Robinson, B. M., Tong, L., Zhang, J., Wolfe, R. A., Goodkin, D. A., Greenwood, R. N., Kerr, P. G., Morgenstern, H., Li, Y., Pisoni, R. L., Saran, R., Tentori, F., Akizawa, T., Fukuhara, S. and Port, F. K. | Blood pressure levels and mortality risk among hemodialysis patients in the Dialysis Outcomes and Practice Patterns Study | 2012 | CVD mortality not stratified by sex |
| 657 | Robinson, P., McEwan, P., Ong, A. C. M., Orskov, B., Sandford, R., Scolari, F., Walz, G., Bennet-Wilton, H. and O'Reilly, K. | Assessing the long term outcomes of autosomal dominant polycystic kidney disease (ADPKD) using the ADPKD outcomes model: a UK case study | 2015 | CVD mortality not stratified by sex |
| 658 | Rocco, M., Daugirdas, J., Greene, T., Lockridge, R., Chan, C., Pierratos, A., Lindsay, R., Larive, B., Chertow, G., Beck, G. and et al. | Mortality during extended follow-up in the frequent hemodialysis network nocturnal trial | 2014 | CVD mortality not stratified by sex |
| 659 | Rodríguez-Osorio, L., de la Piedra, C., Rubert, M., Martín-Fernández, M., González Casaus, M. L., Gracia-Iguacel, C., Egido, J., Villa-Bellosta, R. and González Parra, E. | Differences between 2nd and 3rd generation seric parathormone determination methods on mortality in haemodialysis patients | 2017 | CVD mortality not stratified by sex |
| 660 | Rogacev, K. S., Seiler, S., Zawada, A. M., Reichart, B., Herath, E., Roth, D., Ulrich, C., Fliser, D. and Heine, G. H. | CD14++CD16+ monocytes and cardiovascular outcome in patients with chronic kidney disease | 2011 | CVD mortality not stratified by sex |
| 661 | Rong, R., Zhou, Q., Lin, J., Huang, N., Li, W., Qiu, Y., Yu, X. and Mao, H. | Maintained Folic Acid Supplementation Reduces the Risk of Mortality in Continuous Ambulatory Peritoneal Dialysis Patients | 2018 | CVD mortality not stratified by sex |
| 662 | Rothuizen, T. C., Ocak, G., Verschuren, J. J., Dekker, F. W., Rabelink, T. J., Jukema, J. W. and Rotmans, J. I. | Candidate Gene Analysis of Mortality in Dialysis Patients | 2015 | CVD mortality not stratified by sex |
| 663 | Roumeliotis, A. K., Roumeliotis, S. K., Panagoutsos, S. A., Tsetsos, F., Georgitsi, M., Manolopoulos, V., Paschou, P. and Passadakis, P. S. | Association of ALOX12 gene polymorphism with all-cause and cardiovascular mortality in diabetic nephropathy | 2018 | CVD mortality not stratified by sex |
| 664 | Roumeliotis, S., Roumeliotis, A., Panagoutsos, S., Giannakopoulou, E., Papanas, N., Manolopoulos, V. G., Passadakis, P. and Tavridou, A. | Matrix Gla protein T-138C polymorphism is associated with carotid intima media thickness and predicts mortality in patients with diabetic nephropathy | 2017 | CVD mortality not stratified by sex |
| 665 | Rufino, J. M., Garcia, C., Vega, N., Macia, M., Hernandez, D., Rodriguez, A., Maceira, B. and Lorenzo, V. | Current peritoneal dialysis compared with haemodialysis: medium-term survival analysis of incident dialysis patients in the Canary Islands in recent years | 2011 | CVD mortality not stratified by sex |
| 666 | Ruospo, M., Palmer, S. C., Wong, G., Craig, J. C., Petruzzi, M., De Benedittis, M., Ford, P., Johnson, D. W., Tonelli, M., Natale, P., Saglimbene, V., Pellegrini, F., Celia, E., Gelfman, R., Leal, M. R., Torok, M., Stroumza, P., Bednarek-Skublewska, A., Dulawa, J., Frantzen, L., Del Castillo, D., Schon, S., Bernat, A. G., Hegbrant, J., Wollheim, C., Gargano, L., Bots, C. P., Strippoli, G. F. and Investigators, O. | Periodontitis and early mortality among adults treated with hemodialysis: a multinational propensity-matched cohort study | 2017 | CVD mortality not stratified by sex |
| 667 | Rusu, C., Racasan, S., Moldovan, D., Kacso, I. M., Potra, A., Bondor, C. I., Patiu, I. M., Vladutiu, D. and Caprioara, M. G. | Soluble CD40 ligand in haemodialysis patients: survival impact and cardiovascular prognostic role | 2017 | CVD mortality not stratified by sex |
| 668 | Ryu, D. R., Park, J. T., Chung, J. H., Song, E. M., Roh, S. H., Lee, J. M., An, H. R., Yu, M., Pyun, W. B., Shin, G. J., Kim, S. J., Kang, D. H. and Choi, K. B. | A more appropriate cardiac troponin T level that can predict outcomes in end-stage renal disease patients with acute coronary syndrome | 2011 | CVD mortality not stratified by sex |
| 669 | Saglimbene, V., Palmer, S., Scardapane, M., Craig, J. C., Ruospo, M., Natale, P., Gargano, L., Leal, M., Bednarek-Skublewska, A., Dulawa, J., Ecder, T., Stroumza, P., Marco Murgo, A., Schon, S., Wollheim, C., Hegbrant, J. and Strippoli, G. F. | Depression and all-cause and cardiovascular mortality in patients on haemodialysis: a multinational cohort study | 2017 | CVD mortality not stratified by sex |
| 670 | Sahin, O. Z., Asci, G., Kircelli, F., Yilmaz, M., Duman, S., Ozkahya, M., Dogan, C., Odabas, A. R., Cirit, M. and Ok, E. | The impact of low serum sodium level on mortality depends on glycemic control | 2012 | CVD mortality not stratified by sex |
| 671 | Saji, N., Sato, T., Sakuta, K., Aoki, J., Kobayashi, K., Matsumoto, N., Uemura, J., Shibazaki, K. and Kimura, K. | Chronic kidney disease is an independent predictor of adverse clinical outcomes in patients with recent small subcortical infarcts | 2014 | CVD mortality not stratified by sex |
| 672 | Sakaguchi, Y., Fujii, N., Shoji, T., Hayashi, T., Rakugi, H. and Isaka, Y. | Hypomagnesemia is a significant predictor of cardiovascular and non-cardiovascular mortality in patients undergoing hemodialysis | 2014 | CVD mortality not stratified by sex |
| 673 | Sakaguchi, Y., Fujii, N., Shoji, T., Hayashi, T., Rakugi, H., Iseki, K., Tsubakihara, Y. and Isaka, Y. | Magnesium modifies the cardiovascular mortality risk associated with hyperphosphatemia in patients undergoing hemodialysis: A cohort study | 2014 | CVD mortality not stratified by sex |
| 674 | Sakao, Y., Ojima, T., Yasuda, H., Hashimoto, S., Hasegawa, T., Iseki, K., Tsubakihara, Y. and Kato, A. | Serum Creatinine Modifies Associations between Body Mass Index and Mortality and Morbidity in Prevalent Hemodialysis Patients | 2016 | CVD mortality not stratified by sex |
| 675 | Salvador-Gonzalez, B., Gil-Terron, N., Cerain-Herrero, M. J., Subirana, I., Guell-Miro, R., Rodriguez-Latre, L. M., Cunillera-Puertolas, O., Elosua, R., Grau, M., Vila, J., Pascual-Benito, L., Mestre-Ferrer, J., Ramos, R., Baena-Diez, J. M., Soler-Vila, M., Alonso-Bes, E., Ruiperez-Guijarro, L., Alvarez-Funes, V., Freixes-Villaro, E., Rodriguez-Pascual, M. and Martinez-Castelao, A. | Estimated Glomerular Filtration Rate, Cardiovascular Events and Mortality Across Age Groups Among Individuals Older Than 60 Years in Southern Europe | 2018 | CVD mortality not stratified by sex |
| 676 | Sampaio, M. S., Molnar, M. Z., Kovesdy, C. P., Mehrotra, R., Mucsi, I., Sim, J. J., Krishnan, M., Nissenson, A. R. and Kalantar-Zadeh, K. | Association of pretransplant serum phosphorus with posttransplant outcomes | 2011 | CVD mortality not stratified by sex |
| 677 | Sanchez-Villanueva, R., Estrada, P., del Peso, G., Grande, C., Diez, J. J., Iglesias, P., Gonzalez, E., Aguilar-Rodriguez, A., Selgas, R., Bajo, M. A., Grupo de Estudios Peritoneales de Madrid de, R. and del, I. | Repeated analysis of estimated insulin resistance using the HOMAIR index in nondiabetic patients on peritoneal dialysis and its relationship with cardiovascular disease and mortality | 2013 | CVD mortality not stratified by sex |
| 678 | Sandesara, P. B., O'Neal, W. T., Tahhan, A. S., Hayek, S. S., Lee, S. K., Khambhati, J., Topel, M. L., Hammadah, M., Alkhoder, A., Ko, Y. A., Gafeer, M. M., Beshiri, A., Murtagh, G., Kim, J. H., Wilson, P., Shaw, L., Epstein, S. E., Sperling, L. S. and Quyyumi, A. A. | Comparison of the Association Between High-Sensitivity Troponin I and Adverse Cardiovascular Outcomes in Patients With Versus Without Chronic Kidney Disease | 2018 | CVD mortality not stratified by sex |
| 679 | Santos, P. W., He, J., Tuffaha, A. and Wetmore, J. B. | Clinical characteristics and risk factors associated with mortality in calcific uremic arteriolopathy | 2017 | CVD mortality not stratified by sex |
| 680 | Sarafidis, P. A., Loutradis, C., Karpetas, A., Tzanis, G., Piperidou, A., Koutroumpas, G., Raptis, V., Syrgkanis, C., Liakopoulos, V., Efstratiadis, G., London, G. and Zoccali, C. | Ambulatory Pulse Wave Velocity Is a Stronger Predictor of Cardiovascular Events and All-Cause Mortality Than Office and Ambulatory Blood Pressure in Hemodialysis Patients | 2017 | CVD mortality not stratified by sex |
| 681 | Sato, H., Nagasawa, T., Saito, A. and Miyazaki, M. | Risk of cardiovascular mortality predicted by the serum calcium level and calcification score at the initiation of dialysis | 2018 | CVD mortality not stratified by sex |
| 682 | Sato, H., Takeuchi, Y., Matsuda, K., Saito, A., Kagaya, S., Fukami, H., Ojima, Y. and Nagasawa, T. | Evaluation of the Predictive Value of the Serum Calcium-Magnesium Ratio for All-Cause and Cardiovascular Mortality in Incident Dialysis Patients | 2017 | CVD mortality not stratified by sex |
| 683 | Sato, Y., Fujimoto, S., Toida, T., Nakagawa, H., Yamashita, Y., Iwakiri, T., Fukuda, A. and Iwatsubo, S. | Apoprotein B/Apoprotein A-1 Ratio and Mortality among Prevalent Dialysis Patients | 2016 | CVD mortality not stratified by sex |
| 684 | Sato, Y., Hayashi, T., Joki, N. and Fujimoto, S. | Association of Lead aVR T-wave Amplitude With Cardiovascular Events or Mortality Among Prevalent Dialysis Patients | 2017 | CVD mortality not stratified by sex |
| 685 | Sattar, A., Argyropoulos, C., Weissfeld, L., Younas, N., Fried, L., Kellum, J. A. and Unruh, M. | All-cause and cause-specific mortality associated with diabetes in prevalent hemodialysis patients | 2012 | CVD mortality not stratified by sex |
| 686 | Saunders, M. R., Ricardo, A. C., Chen, J., Chin, M. H. and Lash, J. P. | Association between insurance status and mortality in individuals with albuminuria: an observational cohort study | 2016 | CVD mortality not stratified by sex |
| 687 | Schlackow, I., Kent, S., Herrington, W., Emberson, J., Haynes, R., Reith, C., Wanner, C., Fellstrom, B., Gray, A., Landray, M. J., Baigent, C. and Mihaylova, B. | A policy model of cardiovascular disease in moderate-to-advanced chronic kidney disease | 2017 | CVD mortality not stratified by sex |
| 688 | Schlieper, G., Westenfeld, R., Kruger, T., Cranenburg, E. C., Magdeleyns, E. J., Brandenburg, V. M., Djuric, Z., Damjanovic, T., Ketteler, M., Vermeer, C., Dimkovic, N., Floege, J. and Schurgers, L. J. | Circulating nonphosphorylated carboxylated matrix gla protein predicts survival in ESRD | 2011 | CVD mortality not stratified by sex |
| 689 | Schmaderer, C., Braunisch, M. C., Suttmann, Y., Lorenz, G., Pham, D., Haller, B., Angermann, S., Matschkal, J., Renders, L., Baumann, M., Braun, J. R., Heemann, U. and Kuchle, C. | Reduced Mortality in Maintenance Haemodialysis Patients on High versus Low Dialysate Magnesium: A Pilot Study | 2017 | CVD mortality not stratified by sex |
| 690 | Schneider, C., Coll, B., Jick, S. S. and Meier, C. R. | Doubling of serum creatinine and the risk of cardiovascular outcomes in patients with chronic kidney disease and type 2 diabetes mellitus: a cohort study | 2016 | CVD mortality not stratified by sex |
| 691 | Schrauben, S. J., Jepson, C., Hsu, J. Y., Wilson, F. P., Zhang, X., Lash, J. P., Robinson, B. M., Townsend, R. R., Chen, J., Fogelfeld, L., Kao, P., Landis, J. R., Rader, D. J., Hamm, L. L., Anderson, A. H. and Feldman, H. I. | Insulin resistance and chronic kidney disease progression, cardiovascular events, and death: Findings from the chronic renal insufficiency cohort study 11 Medical and Health Sciences 1103 Clinical Sciences | 2019 | CVD mortality not stratified by sex |
| 692 | Schuett, K., Savvaidis, A., Maxeiner, S., Lysaja, K., Jankowski, V., Schirmer, S. H., Dimkovic, N., Boor, P., Kaesler, N., Dekker, F. W., Floege, J., Marx, N. and Schlieper, G. | Clot Structure: A Potent Mortality Risk Factor in Patients on Hemodialysis | 2017 | CVD mortality not stratified by sex |
| 693 | Schwaiger, J. P., Neyer, U., Sprenger-Mahr, H., Kollerits, B., Mundle, M., Langle, M. and Kronenberg, F. | A simple score predicts future cardiovascular events in an inception cohort of dialysis patients | 2006 | CVD mortality not stratified by sex |
| 694 | Schwantes-An, T. H., Liu, S., Stedman, M., Decker, B. S., Wetherill, L., Edenberg, H. J., Vatta, M., Foroud, T. M., Chertow, G. M. and Moe, S. M. | Fibroblast growth factor 23 genotype and cardiovascular disease in patients undergoing hemodialysis | 2019 | CVD mortality not stratified by sex |
| 695 | Schwedler, S. B., Metzger, T., Schinzel, R. and Wanner, C. | Advanced glycation end products and mortality in hemodialysis patients | 2002 | CVD mortality not stratified by sex |
| 696 | Schwermer, K., Hoppe, K., Radziszewska, D., Kłysz, P., Sawatiuk, P., Nealis, J., Kałuzna, M., Kaczmarek, J., Baum, E., Lindholm, B., Pawlaczyk, K. and Oko, A. | N-terminal pro-B-type natriuretic peptide as a marker of hypervolemia and predictor of increased mortality in patients on hemodialysis | 2015 | CVD mortality not stratified by sex |
| 697 | Scialla, J. J., Kao, W. H., Crainiceanu, C., Sozio, S. M., Oberai, P. C., Shafi, T., Coresh, J., Powe, N. R., Plantinga, L. C., Jaar, B. G. and Parekh, R. S. | Biomarkers of vascular calcification and mortality in patients with ESRD | 2014 | CVD mortality not stratified by sex |
| 698 | Scrutinio, D., Agostoni, P., Gesualdo, L., Corra, U., Mezzani, A., Piepoli, M., Di Lenarda, A., Iorio, A., Passino, C., Magri, D., Masarone, D., Battaia, E., Girola, D., Re, F., Cattadori, G., Parati, G., Sinagra, G., Villani, G. Q., Limongelli, G., Pacileo, G., Guazzi, M., Metra, M., Frigerio, M., Cicoira, M., Mina, C., Malfatto, G., Caravita, S., Bussotti, M., Salvioni, E., Veglia, F., Correale, M., Scardovi, A. B., Emdin, M., Giannuzzi, P., Gargiulo, P., Giovannardi, M., Perrone-Filardi, P., Raimondo, R., Ricci, R., Paolillo, S., Farina, S., Belardinelli, R., Passantino, A., La Gioia, R., Metabolic Exercise test data combined with, C. and Kidney Indexes Score Research, G. | Renal function and peak exercise oxygen consumption in chronic heart failure with reduced left ventricular ejection fraction | 2015 | CVD mortality not stratified by sex |
| 699 | Sederholm Lawesson, S., Alfredsson, J., Szummer, K., Fredrikson, M. and Swahn, E. | Prevalence and prognostic impact of chronic kidney disease in STEMI from a gender perspective: data from the SWEDEHEART register, a large Swedish prospective cohort | 2015 | CVD mortality not stratified by sex |
| 700 | Seiler, S., Schlitt, A., Jiang, X. C., Ulrich, C., Blankenberg, S., Lackner, K. J., Girndt, M., Werdan, K., Buerke, M., Fliser, D. and Heine, G. H. | Cholesteryl ester transfer protein activity and cardiovascular events in patients with chronic kidney disease stage V | 2008 | CVD mortality not stratified by sex |
| 701 | Selim, G., Stojceva-Taneva, O., Ivanovski, N., Zafirovska, K., Sikole, A., Trajcevska, L., Asani, A. and Polenakovic, M. | Inflammation and anaemia as predictors of cardiovascular mortality in hemodialysis patients | 2007 | CVD mortality not stratified by sex |
| 702 | Selim, G., Stojceva-Taneva, O., Spasovski, G., Georgievska-Ismail, L., Zafirovska-Ivanovska, B., Gelev, S., Dzekova, P., Trajcevska, L., Trojacanec-Piponska, S. and Sikole, A. | Brain natriuretic peptide between traditional and nontraditional risk factors in hemodialysis patients: analysis of cardiovascular mortality in a two-year follow-up | 2011 | CVD mortality not stratified by sex |
| 703 | Selim, G. N., Spasovski, G., Tozija, L., Georgievska-Ismail, L., Zafirova-Ivanovska, B., Masin-Spasovska, J., Rambabova-Busletic, I., Petronijevic, Z., Dzekova-Vidimliski, P., Ristovska, V., Pusevski, V. and Stojceva-Taneva, O. | Hypomagnesemia and cause-specific mortality in hemodialysis patients: 5-year follow-up analysis | 2017 | CVD mortality not stratified by sex |
| 704 | Serrano, A., Garcia, F., Serrano, M., Ramirez, E., Alfaro, F. J., Lora, D., de la Camara, A. G., Paz-Artal, E., Praga, M. and Morales, J. M. | IgA antibodies against beta2 glycoprotein I in hemodialysis patients are an independent risk factor for mortality | 2012 | CVD mortality not stratified by sex |
| 705 | Sessa, M., Mascolo, A., Andersen, M. P., Rosano, G., Rossi, F., Capuano, A. and Torp-Pedersen, C. | Effect of Chronic Kidney Diseases on Mortality among Digoxin Users Treated for Non-Valvular Atrial Fibrillation: A Nationwide Register-Based Retrospective Cohort Study | 2016 | CVD mortality not stratified by sex |
| 706 | Shafi, T., Jaar, B. G., Plantinga, L. C., Fink, N. E., Sadler, J. H., Parekh, R. S., Powe, N. R. and Coresh, J. | Association of residual urine output with mortality, quality of life, and inflammation in incident hemodialysis patients: the Choices for Healthy Outcomes in Caring for End-Stage Renal Disease (CHOICE) Study | 2010 | CVD mortality not stratified by sex |
| 707 | Shafi, T., Meyer, T. W., Hostetter, T. H., Melamed, M. L., Parekh, R. S., Hwang, S., Banerjee, T., Coresh, J. and Powe, N. R. | Free Levels of Selected Organic Solutes and Cardiovascular Morbidity and Mortality in Hemodialysis Patients: Results from the Retained Organic Solutes and Clinical Outcomes (ROSCO) Investigators | 2015 | CVD mortality not stratified by sex |
| 708 | Shafi, T., Sozio, S. M., Luly, J., Bandeen-Roche, K. J., St Peter, W. L., Ephraim, P. L., McDermott, A., Herzog, C. A., Crews, D. C., Scialla, J. J., Tangri, N., Miskulin, D. C., Michels, W. M., Jaar, B. G., Zager, P. G., Meyer, K. B., Wu, A. W. and Boulware, L. E. | Antihypertensive medications and risk of death and hospitalizations in US hemodialysis patients | 2017 | CVD mortality not stratified by sex |
| 709 | Shah, A. N., Mentz, R. J., Gheorghiade, M., Kwasny, M. J., Fought, A. J., Zannad, F., Swedberg, K., Maggioni, A. P. and Konstam, M. A. | Gender does not affect postdischarge outcomes in patients hospitalized for worsening heart failure with reduced ejection fraction (from the Efficacy of Vasopressin Antagonism in Heart Failure Outcome Study with Tolvaptan EVEREST Trial) | 2012 | CVD mortality not stratified by sex |
| 710 | Shah, N. R., Charytan, D. M., Murthy, V. L., Skali Lami, H., Veeranna, V., Cheezum, M. K., Taqueti, V. R., Kato, T., Foster, C. R., Hainer, J., Gaber, M., Klein, J., Dorbala, S., Blankstein, R. and Di Carli, M. F. | Prognostic Value of Coronary Flow Reserve in Patients with Dialysis-Dependent ESRD | 2016 | CVD mortality not stratified by sex |
| 711 | Shardlow, A., McIntyre, N. J., Fluck, R. J., McIntyre, C. W. and Taal, M. W. | Chronic Kidney Disease in Primary Care: Outcomes after Five Years in a Prospective Cohort Study | 2016 | CVD mortality not stratified by sex |
| 712 | Shen, H., Chang, X., Zheng, X., Du, W., Zhao, B., Wang, W. and Lou, X. | Association between C-reactive protein and mortality in peritoneal dialysis patients: A meta-analysis | 2016 | CVD mortality not stratified by sex |
| 713 | Shen, J. I., Saxena, A. B., Montez-Rath, M. E., Chang, T. I. and Winkelmayer, W. C. | Angiotensin-converting enzyme inhibitor/angiotensin receptor blocker use and cardiovascular outcomes in patients initiating peritoneal dialysis | 2017 | CVD mortality not stratified by sex |
| 714 | Sheng, X., Murphy, M. J., Macdonald, T. M. and Wei, L. | Effectiveness of statins in chronic kidney disease | 2012 | CVD mortality not stratified by sex |
| 715 | Shima, H., Mori, T., Ooi, M., Sonoda, M., Shoji, T., Ishimura, E., Okamura, M., Ishizaka, N. and Inaba, M. | Silent Cerebral Microbleeds and Longitudinal Risk of Renal and Cardiovascular Events in Patients with CKD | 2016 | CVD mortality not stratified by sex |
| 716 | Shimizu, T., Yoshihisa, A., Kanno, Y., Takiguchi, M., Sato, A., Miura, S., Nakamura, Y., Yamauchi, H., Owada, T., Abe, S., Sato, T., Suzuki, S., Oikawa, M., Yamaki, T., Sugimoto, K., Kunii, H., Nakazato, K., Suzuki, H., Saitoh, S. and Takeishi, Y. | Relationship of hyperuricemia with mortality in heart failure patients with preserved ejection fraction | 2015 | CVD mortality not stratified by sex |
| 717 | Shimoyama, Y., Mitsuda, Y., Tsuruta, Y., Hamajima, N. and Niwa, T. | Polymorphism of Nrf2, an antioxidative gene, is associated with blood pressure and cardiovascular mortality in hemodialysis patients | 2014 | CVD mortality not stratified by sex |
| 718 | Shimoyama, Y., Tsuruta, Y. and Niwa, T. | Coronary artery calcification score is associated with mortality in Japanese hemodialysis patients | 2012 | CVD mortality not stratified by sex |
| 719 | Shishehbor, M. H., Oliveira, L. P., Lauer, M. S., Sprecher, D. L., Wolski, K., Cho, L., Hoogwerf, B. J. and Hazen, S. L. | Emerging cardiovascular risk factors that account for a significant portion of attributable mortality risk in chronic kidney disease | 2008 | CVD mortality not stratified by sex |
| 720 | Shlipak, M. G., Fried, L. F., Cushman, M., Manolio, T. A., Peterson, D., Stehman-Breen, C., Bleyer, A., Newman, A., Siscovick, D. and Psaty, B. | Cardiovascular mortality risk in chronic kidney disease: Comparison of traditional and novel risk factors | 2005 | CVD mortality not stratified by sex |
| 721 | Shoji, T., Marubayashi, S., Shigematsu, T., Iseki, K. and Tsubakihara, Y. | Use of Vitamin D Receptor Activator, Incident Cardiovascular Disease and Death in a Cohort of Hemodialysis Patients | 2015 | CVD mortality not stratified by sex |
| 722 | Shurraw, S., Hemmelgarn, B., Lin, M., Majumdar, S. R., Klarenbach, S., Manns, B., Bello, A., James, M., Turin, T. C., Tonelli, M. and Alberta Kidney Disease, N. | Association between glycemic control and adverse outcomes in people with diabetes mellitus and chronic kidney disease: a population-based cohort study | 2011 | CVD mortality not stratified by sex |
| 723 | Silberberg, J. S., Barre, P. E., Prichard, S. S. and Sniderman, A. D. | Impact of left ventricular hypertrophy on survival in end-stage renal disease | 1989 | CVD mortality not stratified by sex |
| 724 | Silva, A. P., Fragoso, A., Pinho, A., Tavares, N., Camacho, A., Faisca, M. and Leao Neves, P. | Phosphorus as an early marker of morbidity and mortality in type 2 chronic kidney disease diabetic patients | 2013 | CVD mortality not stratified by sex |
| 725 | Sim, J. J., Bhandari, S. K., Shi, J., Reynolds, K., Calhoun, D. A., Kalantar-Zadeh, K. and Jacobsen, S. J. | Comparative risk of renal, cardiovascular, and mortality outcomes in controlled, uncontrolled resistant, and nonresistant hypertension | 2015 | CVD mortality not stratified by sex |
| 726 | Sin, H. Y. | Prospective cohort study: Cinacalcet-mediated lowering of PTH level and cardiovascular disease mortality in younger Korean patients with stage 5 CKD at a Korean secondary hospital | 2017 | CVD mortality not stratified by sex |
| 727 | Sipahioglu, M. H., Kucuk, H., Unal, A., Kaya, M. G., Oguz, F., Tokgoz, B., Oymak, O. and Utas, C. | Impact of arterial stiffness on adverse cardiovascular outcomes and mortality in peritoneal dialysis patients | 2012 | CVD mortality not stratified by sex |
| 728 | Smilowitz, N. R., Gupta, N., Guo, Y., Mauricio, R. and Bangalore, S. | Management and outcomes of acute myocardial infarction in patients with chronic kidney disease | 2017 | CVD mortality not stratified by sex |
| 729 | Smith, D. H., Johnson, E. S., Thorp, M. L., Petrik, A., Yang, X. and Blough, D. K. | Outcomes predicted by phosphorous in chronic kidney disease: a retrospective CKD-inception cohort study | 2010 | CVD mortality not stratified by sex |
| 730 | Smith, D. H., Thorp, M. L., Gurwitz, J. H., McManus, D. D., Goldberg, R. J., Allen, L. A., Hsu, G., Sung, S. H., Magid, D. J. and Go, A. S. | Chronic kidney disease and outcomes in heart failure with preserved versus reduced ejection fraction: the Cardiovascular Research Network PRESERVE Study | 2013 | CVD mortality not stratified by sex |
| 731 | Sokolski, M., Zymlinski, R., Biegus, J., Siwolowski, P., Nawrocka-Millward, S., Todd, J., Yerramilli, M. R., Estis, J., Jankowska, E. A., Banasiak, W. and Ponikowski, P. | Urinary levels of novel kidney biomarkers and risk of true worsening renal function and mortality in patients with acute heart failure | 2017 | CVD mortality not stratified by sex |
| 732 | Solak, Y., Yilmaz, M. I., Saglam, M., Demirbas, S., Verim, S., Unal, H. U., Gaipov, A., Oguz, Y., Kayrak, M., Caglar, K., Vural, A., Turk, S., Covic, A. and Kanbay, M. | Mean corpuscular volume is associated with endothelial dysfunction and predicts composite cardiovascular events in patients with chronic kidney disease | 2013 | CVD mortality not stratified by sex |
| 733 | Sonmez, A., Yilmaz, M. I., Saglam, M., Unal, H. U., Gok, M., Cetinkaya, H., Karaman, M., Haymana, C., Eyileten, T., Oguz, Y., Vural, A., Rizzo, M. and Toth, P. P. | The role of plasma triglyceride/high-density lipoprotein cholesterol ratio to predict cardiovascular outcomes in chronic kidney disease | 2015 | CVD mortality not stratified by sex |
| 734 | Sood, M. M., Akbari, A., Manuel, D. G., Ruzicka, M., Hiremath, S., Zimmerman, D., McCormick, B. and Taljaard, M. | Longitudinal blood pressure in late-stage chronic kidney disease and the risk of end-stage kidney disease or mortality (Best blood pressure in chronic kidney disease study) | 2017 | CVD mortality not stratified by sex |
| 735 | Spencer-Hwang, R., Knutsen, S. F., Soret, S., Ghamsary, M., Beeson, W. L., Oda, K., Shavlik, D. and Jaipaul, N. | Ambient air pollutants and risk of fatal coronary heart disease among kidney transplant recipients | 2011 | CVD mortality not stratified by sex |
| 736 | Spiegel, D. M., Raggi, P., Smits, G. and Block, G. A. | Factors associated with mortality in patients new to haemodialysis | 2007 | CVD mortality not stratified by sex |
| 737 | Stack, A. G., Donigiewicz, U., Abdalla, A. A., Weiland, A., Casserly, L. F., Cronin, C. J., Nguyen, H. T. and Hannigan, A. | Plasma fibrinogen associates independently with total and cardiovascular mortality among subjects with normal and reduced kidney function in the general population | 2014 | CVD mortality not stratified by sex |
| 738 | Stack, A. G., Neylon, A. M., Abdalla, A. A., Hegarty, A., Hannigan, A., Cronin, C. J., Nguyen, H. T. and Casserly, L. F. | Declining mortality rates despite increases in clinical coronary artery disease among US dialysis patients: a national registry study | 2013 | CVD mortality not stratified by sex |
| 739 | Stack, A. G. and Saran, R. | Clinical correlates and mortality impact of left ventricular hypertrophy among new ESRD patients in the United States | 2002 | CVD mortality not stratified by sex |
| 740 | Stack, A. G., Yermak, D., Roche, D. G., Ferguson, J. P., Elsayed, M., Mohammed, W., Casserly, L. F., Walsh, S. R. and Cronin, C. J. | Differential impact of smoking on mortality and kidney transplantation among adult Men and Women undergoing dialysis | 2016 | CVD mortality not stratified by sex |
| 741 | Stenvinkel, P., Gillespie, I. A., Tunks, J., Addison, J., Kronenberg, F., Drueke, T. B., Marcelli, D., Schernthaner, G., Eckardt, K. U., Floege, J., Froissart, M. and Anker, S. D. | Inflammation modifies the paradoxical association between body mass index and mortality in hemodialysis patients | 2016 | CVD mortality not stratified by sex |
| 742 | Stidley, C. A., Hunt, W. C., Tentori, F., Schmidt, D., Rohrscheib, M., Paine, S., Bedrick, E. J., Meyer, K. B., Johnson, H. K. and Zager, P. G. | Changing relationship of blood pressure with mortality over time among hemodialysis patients | 2006 | CVD mortality not stratified by sex |
| 743 | Stirnadel-Farrant, H. A., Luo, J., Kler, L., Cizman, B., Jones, D., Brunelli, S. M. and Cobitz, A. R. | Anemia and mortality in patients with nondialysis-dependent chronic kidney disease | 2018 | CVD mortality not stratified by sex |
| 744 | Stolic, R., Trajkovic, G., Jovanovic, A., Stolic, D., Peric, V., Sovtic, S. and Subaric-Gorgieva, G. | Carotid ultrasonographic parameters as markers of atherogenesis and mortality rate in patients on hemodialysis | 2010 | CVD mortality not stratified by sex |
| 745 | Straw, S., Byrom, R., Gierula, J., Paton, M. F., Koshy, A., Cubbon, R., Drozd, M., Kearney, M. and Witte, K. K. | Predicting one-year mortality in heart failure using the 'Surprise Question': a prospective pilot study | 2019 | CVD mortality not stratified by sex |
| 746 | Stubbs, J. R., Stedman, M. R., Liu, S., Long, J., Franchetti, Y., West, R. E., Prokopienko, A. J., Mahnken, J. D., Chertow, G. M. and Nolin, T. D. | Trimethylamine N-oxide and cardiovascular outcomes in patients with ESKD receiving maintenance hemodialysis | 2019 | CVD mortality not stratified by sex |
| 747 | Su, C. T., Yabes, J., Pike, F., Weiner, D. E., Beddhu, S., Burrowes, J. D., Rocco, M. V. and Unruh, M. L. | Changes in anthropometry and mortality in maintenance hemodialysis patients in the HEMO Study | 2013 | CVD mortality not stratified by sex |
| 748 | Sud, M., Tangri, N., Pintilie, M., Levey, A. S. and Naimark, D. M. | ESRD and death after heart failure in CKD | 2015 | CVD mortality not stratified by sex |
| 749 | Sugimoto, H., Ogawa, T., Iwabuchi, Y., Otsuka, K. and Nitta, K. | Relationship between serum fibroblast growth factor-23 level and mortality in chronic hemodialysis patients | 2014 | CVD mortality not stratified by sex |
| 750 | Sugiura, S., Inaguma, D., Kitagawa, A., Murata, M., Kamimura, Y., Sendo, S., Hamaguchi, K., Nagaya, H., Tatematsu, M., Kurata, K., Yuzawa, Y. and Matsuo, S. | Administration of alfacalcidol for patients with predialysis chronic kidney disease may reduce cardiovascular disease events | 2010 | CVD mortality not stratified by sex |
| 751 | Sumida, K., Molnar, M. Z., Potukuchi, P. K., Thomas, F., Lu, J. L., Jing, J., Ravel, V. A., Soohoo, M., Rhee, C. M., Streja, E., Kalantar-Zadeh, K. and Kovesdy, C. P. | Association of Slopes of Estimated Glomerular Filtration Rate With Post-End-Stage Renal Disease Mortality in Patients With Advanced Chronic Kidney Disease Transitioning to Dialysis | 2016 | CVD mortality not stratified by sex |
| 752 | Sumida, K., Molnar, M. Z., Potukuchi, P. K., Thomas, F., Lu, J. L., Obi, Y., Rhee, C. M., Streja, E., Yamagata, K., Kalantar-Zadeh, K. and Kovesdy, C. P. | Prognostic significance of pre-end-stage renal disease serum alkaline phosphatase for post-end-stage renal disease mortality in late-stage chronic kidney disease patients transitioning to dialysis | 2018 | CVD mortality not stratified by sex |
| 753 | Sumida, K., Yamagata, K., Iseki, K. and Tsubakihara, Y. | Different impact of hemodialysis vintage on cause-specific mortality in long-term hemodialysis patients | 2016 | CVD mortality not stratified by sex |
| 754 | Sun, H., Xian, W., Geng, L., Li, E., Peng, Z. and Tian, J. | Increased plasma level of catestatin might be associated with poor prognosis in hemodialysis patients | 2017 | CVD mortality not stratified by sex |
| 755 | Sun, W., Liu, D., Shi, X., Gong, P., Wang, P. and Gong, W. | Biomarkers for cardiovascular mortality in chronic kidney disease patients | 2014 | CVD mortality not stratified by sex |
| 756 | Suvakov, S., Damjanovic, T., Pekmezovic, T., Jakovljevic, J., Savic-Radojevic, A., Pljesa-Ercegovac, M., Radovanovic, S., Simic, D. V., Pljesa, S., Zarkovic, M., Mimic-Oka, J., Dimkovic, N. and Simic, T. | Associations of GSTM1 0 and GSTA1 A genotypes with the risk of cardiovascular death among hemodialyses patients | 2014 | CVD mortality not stratified by sex |
| 757 | Suzuki, A., Obi, Y., Hayashi, T., Kotani, N., Uemura, Y., Imai, E., Makino, H. and Hishida, A. | Visit-to-visit variability in estimated glomerular filtration rate predicts hospitalization and death due to cardiovascular events | 2019 | CVD mortality not stratified by sex |
| 758 | Suzuki, H., Kanno, Y., Sugahara, S., Ikeda, N., Shoda, J., Takenaka, T., Inoue, T. and Araki, R. | Effect of angiotensin receptor blockers on cardiovascular events in patients undergoing hemodialysis: an open-label randomized controlled trial | 2008 | CVD mortality not stratified by sex |
| 759 | Suzuki, S., Shino, M., Fujikawa, T., Itoh, Y., Ueda, E., Hashimoto, T., Kuji, T., Kobayashi, N., Ohnishi, T., Hirawa, N., Tamura, K. and Toya, Y. | Plasma Cystine Levels and Cardiovascular and All-Cause Mortality in Hemodialysis Patients | 2018 | CVD mortality not stratified by sex |
| 760 | Szeto, C. C., Wong, T. Y., Chow, K. M., Leung, C. B. and Li, P. K. | Are peritoneal dialysis patients with and without residual renal function equivalent for survival study? Insight from a retrospective review of the cause of death | 2003 | CVD mortality not stratified by sex |
| 761 | Takagi, A., Iwama, Y., Yamada, A., Aihara, K. and Daida, H. | Estimated glomerular filtration rate is an independent predictor for mortality of patients with acute heart failure | 2010 | CVD mortality not stratified by sex |
| 762 | Takahashi, R., Ito, Y., Takahashi, H., Ishii, H., Kasuga, H., Mizuno, M., Suzuki, Y., Yuzawa, Y., Maruyama, S., Murohara, T., Imai, E. and Matsuo, S. | Combined values of serum albumin, C-reactive protein and body mass index at dialysis initiation accurately predicts long-term mortality | 2012 | CVD mortality not stratified by sex |
| 763 | Takeda, A., Toda, T., Fujii, T., Shinohara, S., Sasaki, S. and Matsui, N. | Discordance of influence of hypertension on mortality and cardiovascular risk in hemodialysis patients | 2005 | CVD mortality not stratified by sex |
| 764 | Takenaka, T., Sato, T., Hoshi, H., Kato, N., Sueyoshi, K., Tsuda, M., Watanabe, Y., Takane, H., Ohno, Y. and Suzuki, H. | Height constitutes an important predictor of mortality in end-stage renal disease | 2011 | CVD mortality not stratified by sex |
| 765 | Taki, F. and Komatsu, Y. | Serum beta 2 microglobulin on initiation of dialysis and mortality | 2017 | CVD mortality not stratified by sex |
| 766 | Tan, J., Bae, S., Segal, J. B., Zhu, J., Alexander, G. C., Segev, D. L. and McAdams-DeMarco, M. | Warfarin use and the risk of stroke, bleeding, and mortality in older adults on dialysis with incident atrial fibrillation | 2019 | CVD mortality not stratified by sex |
| 767 | Tan, S. H., Prowant, B. F., Khanna, R., Nolph, K. D. and Twardowski, Z. J. | Cardiovascular comorbidity and mortality in patients starting peritoneal dialysis: an American midwestern center experience | 2001 | CVD mortality not stratified by sex |
| 768 | Tanaka, A., Inaguma, D., Shinjo, H., Murata, M. and Takeda, A. | Presence of Atrial Fibrillation at the Time of Dialysis Initiation Is Associated with Mortality and Cardiovascular Events | 2016 | CVD mortality not stratified by sex |
| 769 | Tanaka, K., Watanabe, T., Takeuchi, A., Ohashi, Y., Nitta, K., Akizawa, T., Matsuo, S., Imai, E., Makino, H., Hishida, A. and Investigators, C. J. | Cardiovascular events and death in Japanese patients with chronic kidney disease | 2017 | CVD mortality not stratified by sex |
| 770 | Tanaka, M., Ishii, H., Aoyama, T., Takahashi, H., Toriyama, T., Kasuga, H., Takeshita, K., Yoshikawa, D., Amano, T. and Murohara, T. | Ankle brachial pressure index but not brachial-ankle pulse wave velocity is a strong predictor of systemic atherosclerotic morbidity and mortality in patients on maintenance hemodialysis | 2011 | CVD mortality not stratified by sex |
| 771 | Tanno, K., Ohsawa, M., Itai, K., Kato, K., Turin, T. C., Onoda, T., Sakata, K., Okayama, A. and Fujioka, T. | Associations of marital status with mortality from all causes and mortality from cardiovascular disease in Japanese haemodialysis patients | 2013 | CVD mortality not stratified by sex |
| 772 | Temgoua, M. N., Danwang, C., Agbor, V. N. and Noubiap, J. J. | Prevalence, incidence and associated mortality of cardiovascular disease in patients with chronic kidney disease in low- and middle-income countries: a protocol for a systematic review and meta-analysis | 2017 | CVD mortality not stratified by sex |
| 773 | Teng, T. H., Katzenellenbogen, J. M., Hung, J., Knuiman, M., Sanfilippo, F. M., Geelhoed, E., Hobbs, M. and Thompson, S. C. | Rural-urban differentials in 30-day and 1-year mortality following first-ever heart failure hospitalisation in Western Australia: a population-based study using data linkage | 2014 | CVD mortality not stratified by sex |
| 774 | Tentori, F., Karaboyas, A., Robinson, B. M., Morgenstern, H., Zhang, J., Sen, A., Ikizler, T. A., Rayner, H., Fissell, R. B., Vanholder, R., Tomo, T. and Port, F. K. | Association of dialysate bicarbonate concentration with mortality in the Dialysis Outcomes and Practice Patterns Study (DOPPS) | 2013 | CVD mortality not stratified by sex |
| 775 | Tepel, M., Giet, M. V., Park, A. and Zidek, W. | Association of calcium channel blockers and mortality in haemodialysis patients | 2002 | CVD mortality not stratified by sex |
| 776 | Tereshchenko, L. G., Kim, E. D., Oehler, A., Meoni, L. A., Ghafoori, E., Rami, T., Maly, M., Kabir, M., Hawkins, L., Tomaselli, G. F., Lima, J. A., Jaar, B. G., Sozio, S. M., Estrella, M., Kao, W. H. and Parekh, R. S. | Electrophysiologic Substrate and Risk of Mortality in Incident Hemodialysis | 2016 | CVD mortality not stratified by sex |
| 777 | Testa, A., Leonardis, D., Spoto, B., Sanguedolce, M. C., Parlongo, R. M., Pisano, A., Tripepi, G., Mallamaci, F. and Zoccali, C. | A polymorphism in a major antioxidant gene (Kelch-like ECH-associated protein 1) predicts incident cardiovascular events in chronic kidney disease patients: an exploratory study | 2016 | CVD mortality not stratified by sex |
| 778 | Testa, A., Torino, C., Postorino, M., Spoto, B., Sanguedolce, M. C., Parlongo, R. M., Tripepi, G., Mallamaci, F. and Zoccali, C. | The nature of the association between FGF23 and mortality and cardiovascular disease in end stage kidney disease patients: a mendelian randomization study | 2017 | CVD mortality not stratified by sex |
| 779 | Thompson, S., James, M., Wiebe, N., Hemmelgarn, B., Manns, B., Klarenbach, S. and Tonelli, M. | Cause of Death in Patients with Reduced Kidney Function | 2015 | CVD mortality not stratified by sex |
| 780 | Thorp, M. L., Johnson, E. S., Yang, X., Petrik, A. F., Platt, R. and Smith, D. H. | Effect of anaemia on mortality, cardiovascular hospitalizations and end-stage renal disease among patients with chronic kidney disease | 2009 | CVD mortality not stratified by sex |
| 781 | Tian, S. L., Tian, X. K., Han, Q. F. and Wang, T. | Peripheral arterial disease predicts overall and cardiovascular mortality in peritoneal dialysis patients | 2012 | CVD mortality not stratified by sex |
| 782 | Toida, T., Iwakiri, T., Sato, Y., Komatsu, H., Kitamura, K. and Fujimoto, S. | Relationship between Hemoglobin Levels Corrected by Interdialytic Weight Gain and Mortality in Japanese Hemodialysis Patients: Miyazaki Dialysis Cohort Study | 2017 | CVD mortality not stratified by sex |
| 783 | Tonelli, M., Keech, A., Shepherd, J., Sacks, F., Tonkin, A., Packard, C., Pfeffer, M., Simes, J., Isles, C., Furberg, C. and et al. | Effect of pravastatin in people with diabetes and chronic kidney disease | 2005 | CVD mortality not stratified by sex |
| 784 | Torino, C., Mattace-Raso, F., van Saase, J. L., D'Arrigo, G., Tripepi, R., Tripepi, G. L., Postorino, M., Mallamaci, F. and Zoccali, C. | Snoring amplifies the risk of heart failure and mortality in dialysis patients | 2014 | CVD mortality not stratified by sex |
| 785 | Torino, C., Pizzini, P., Cutrupi, S., Postorino, M., Tripepi, G., Mallamaci, F., Reiser, J., Zoccali, C., Alati, G., Barreca, E., Boito, R., Bovino, M., Bruzzese, V., Capria, M., Cassani, S., Chiarella, S., Chippari, A., Cicchetti, T., Crifò-Gasparro, E., Curti, C., D'Agostino, F., D'Anello, E., De Gaudio, M., Foscaldi, A., Fornaciari, C., Franco, C., Gaglioti, A., Galati, D., Grandinetti, F., Gullo, M., La Gamba, M. R., Logozzo, D., Maimone, I., Mannino, M. L., Mazzuca, E., Mellace, A., Natale, G., Panuccio, V., Plutino, D., Pugliese, A., Reina, A., Roberti, R., Santangelo, M., Sellaro, A., Scicchitano, R., Vardè, C. and Zingone, F. | Soluble Urokinase Plasminogen Activator Receptor (suPAR) and All-Cause and Cardiovascular Mortality in Diverse Hemodialysis Patients | 2018 | CVD mortality not stratified by sex |
| 786 | Torraca, S., Sirico, M. L., Guastaferro, P., Morrone, L. F., Nigro, F., Blasio, A. D., Romano, P., Russo, D., Bellasi, A. and Di Iorio, B. | Variability of pulse wave velocity and mortality in chronic hemodialysis patients | 2011 | CVD mortality not stratified by sex |
| 787 | Townsend, R. R., Anderson, A. H., Chirinos, J. A., Feldman, H. I., Grunwald, J. E., Nessel, L., Roy, J., Weir, M. R., Wright, J. T., Jr., Bansal, N. and Hsu, C. Y. | Association of Pulse Wave Velocity With Chronic Kidney Disease Progression and Mortality: Findings From the CRIC Study (Chronic Renal Insufficiency Cohort) | 2018 | CVD mortality not stratified by sex |
| 788 | Tripepi, G., Mallamaci, F. and Zoccali, C. | Inflammation markers, adhesion molecules, and all-cause and cardiovascular mortality in patients with ESRD: searching for the best risk marker by multivariate modeling | 2005 | CVD mortality not stratified by sex |
| 789 | Tripepi, G., Pannier, B., D'Arrigo, G., Mallamaci, F., Zoccali, C. and London, G. | Reappraisal in two European cohorts of the prognostic power of left ventricular mass index in chronic kidney failure | 2017 | CVD mortality not stratified by sex |
| 790 | Tsai, M. T., Hu, F. H., Lien, T. J., Chen, P. J., Huang, T. P. and Tarng, D. C. | Interaction between geriatric nutritional risk index and decoy receptor 3 predicts mortality in chronic hemodialysis patients | 2014 | CVD mortality not stratified by sex |
| 791 | Tsai, Y. C., Lee, C. S., Chiu, Y. W., Kuo, H. T., Lee, S. C., Hwang, S. J., Kuo, M. C. and Chen, H. C. | Angiopoietin-2 as a Prognostic Biomarker of Major Adverse Cardiovascular Events and All-Cause Mortality in Chronic Kidney Disease | 2015 | CVD mortality not stratified by sex |
| 792 | Tsai, Y. C., Lee, C. S., Chiu, Y. W., Lee, J. J., Lee, S. C., Hsu, Y. L. and Kuo, M. C. | Angiopoietin-2, Renal Deterioration, Major Adverse Cardiovascular Events and All-Cause Mortality in Patients with Diabetic Nephropathy | 2018 | CVD mortality not stratified by sex |
| 793 | Tseng, W. C., Liu, J. S., Hung, S. C., Kuo, K. L., Chen, Y. H., Tarng, D. C. and Hsu, C. C. | Effect of spironolactone on the risks of mortality and hospitalization for heart failure in pre-dialysis advanced chronic kidney disease: A nationwide population-based study | 2017 | CVD mortality not stratified by sex |
| 794 | Tuegel, C., Katz, R., Alam, M., Bhat, Z., Bellovich, K., de Boer, I., Brosius, F., Gadegbeku, C., Gipson, D., Hawkins, J., Himmelfarb, J., Ju, W., Kestenbaum, B., Kretzler, M., Robinson-Cohen, C., Steigerwalt, S. and Bansal, N. | GDF-15, Galectin 3, Soluble ST2, and Risk of Mortality and Cardiovascular Events in CKD | 2018 | CVD mortality not stratified by sex |
| 795 | Turakhia, M. P., Blankestijn, P. J., Carrero, J. J., Clase, C. M., Deo, R., Herzog, C. A., Kasner, S. E., Passman, R. S., Pecoits-Filho, R., Reinecke, H., Shroff, G. R., Zareba, W., Cheung, M., Wheeler, D. C., Winkelmayer, W. C. and Wanner, C. | Chronic kidney disease and arrhythmias: Conclusions from a Kidney Disease: Improving Global Outcomes (KDIGO) Controversies Conference | 2018 | CVD mortality not stratified by sex |
| 796 | Tziomalos, K., Giampatzis, V., Baltatzi, M., Efthymiou, E., Psianou, K., Papastergiou, N., Magkou, D., Bougatsa, V., Savopoulos, C. and Hatzitolios, A. I. | Sex-specific differences in cardiovascular risk factors and blood pressure control in hypertensive patients | 2014 | CVD mortality not stratified by sex |
| 797 | Ulrich, C., Heine, G. H., Seibert, E., Fliser, D. and Girndt, M. | Circulating monocyte subpopulations with high expression of angiotensin-converting enzyme predict mortality in patients with end-stage renal disease | 2010 | CVD mortality not stratified by sex |
| 798 | Undas, A., Kolarz, M., Kopec, G. and Tracz, W. | Altered fibrin clot properties in patients on long-term haemodialysis: relation to cardiovascular mortality | 2008 | CVD mortality not stratified by sex |
| 799 | Untersteller, K., Meissl, S., Trieb, M., Emrich, I. E., Zawada, A. M., Holzer, M., Knuplez, E., Fliser, D., Heine, G. H. and Marsche, G. | HDL functionality and cardiovascular outcome among nondialysis chronic kidney disease patients | 2018 | CVD mortality not stratified by sex |
| 800 | Usvyat, L. A., Carter, M., Thijssen, S., Kooman, J. P., van der Sande, F. M., Zabetakis, P., Balter, P., Levin, N. W. and Kotanko, P. | Seasonal variations in mortality, clinical, and laboratory parameters in hemodialysis patients: A 5-year cohort study | 2012 | CVD mortality not stratified by sex |
| 801 | van der Sman-de Beer, F., Verhagen, C., Rombach, S. M., Boorsma, P., van Manen, J. G., Korevaar, J. C., van den Bogaard, R., Boeschoten, E. W., Krediet, R. T., Navis, G. J., Vandenbroucke, J. P. and Dekker, F. W. | ACE I/D polymorphism is associated with mortality in a cohort study of patients starting with dialysis | 2005 | CVD mortality not stratified by sex |
| 802 | van der Velde, M., Matsushita, K., Coresh, J., Astor, B. C., Woodward, M., Levey, A., de Jong, P., Gansevoort, R. T., Chronic Kidney Disease Prognosis, C., van der Velde, M., Matsushita, K., Coresh, J., Astor, B. C., Woodward, M., Levey, A. S., de Jong, P. E., Gansevoort, R. T., Levey, A., El-Nahas, M., Eckardt, K. U., Kasiske, B. L., Ninomiya, T., Chalmers, J., Macmahon, S., Tonelli, M., Hemmelgarn, B., Sacks, F., Curhan, G., Collins, A. J., Li, S., Chen, S. C., Hawaii Cohort, K. P., Lee, B. J., Ishani, A., Neaton, J., Svendsen, K., Mann, J. F., Yusuf, S., Teo, K. K., Gao, P., Nelson, R. G., Knowler, W. C., Bilo, H. J., Joosten, H., Kleefstra, N., Groenier, K. H., Auguste, P., Veldhuis, K., Wang, Y., Camarata, L., Thomas, B. and Manley, T. | Lower estimated glomerular filtration rate and higher albuminuria are associated with all-cause and cardiovascular mortality. A collaborative meta-analysis of high-risk population cohorts | 2011 | CVD mortality not stratified by sex |
| 803 | van Dijk, S., van den Beukel, T. O., Kaptein, A. A., Honig, A., le Cessie, S., Siegert, C. E., Boeschoten, E. W., Krediet, R. T. and Dekker, F. W. | How baseline, new-onset, and persistent depressive symptoms are associated with cardiovascular and non-cardiovascular mortality in incident patients on chronic dialysis | 2013 | CVD mortality not stratified by sex |
| 804 | Van Pottelbergh, G., Vaes, B., Adriaensen, W., Mathei, C., Legrand, D., Wallemacq, P. and Degryse, J. M. | The glomerular filtration rate estimated by new and old equations as a predictor of important outcomes in elderly patients | 2014 | CVD mortality not stratified by sex |
| 805 | Vashistha, T., Kalantar-Zadeh, K., Molnar, M. Z., Torlen, K. and Mehrotra, R. | Dialysis modality and correction of uremic metabolic acidosis: relationship with all-cause and cause-specific mortality | 2013 | CVD mortality not stratified by sex |
| 806 | Vega, A., Abad, S., Macías, N., Aragoncillo, I., García-Prieto, A., Linares, T., Torres, E., Hernández, A. and Luño, J. | Any grade of relative overhydration is associated with long-term mortality in patients with Stages 4 and 5 non-dialysis chronic kidney disease | 2018 | CVD mortality not stratified by sex |
| 807 | Verbeke, F., Marechal, C., Van Laecke, S., Van Biesen, W., Devuyst, O., Van Bortel, L. M., Jadoul, M. and Vanholder, R. | Aortic stiffness and central wave reflections predict outcome in renal transplant recipients | 2011 | CVD mortality not stratified by sex |
| 808 | Verbeke, F., Van Biesen, W., Honkanen, E., Wikström, B., Jensen, P. B., Krzesinski, J. M., Rasmussen, M., Vanholder, R. and Rensma, P. L. | Prognostic value of aortic stiffness and calcification for cardiovascular events and mortality in dialysis patients: Outcome of the Calcification Outcome in Renal Disease (CORD) study | 2011 | CVD mortality not stratified by sex |
| 809 | Vlagopoulos, P. T., Tighiouart, H., Weiner, D. E., Griffith, J., Pettitt, D., Salem, D. N., Levey, A. S. and Sarnak, M. J. | Anemia as a risk factor for cardiovascular disease and all-cause mortality in diabetes: the impact of chronic kidney disease | 2005 | CVD mortality not stratified by sex |
| 810 | Voskamp, P. W. M., Rookmaaker, M. B., Verhaar, M. C., Dekker, F. W. and Ocak, G. | Vitamin K antagonist use and mortality in dialysis patients | 2018 | CVD mortality not stratified by sex |
| 811 | Wagner, S., Metzger, M., Flamant, M., Houillier, P., Haymann, J. P., Vrtovsnik, F., Thervet, E., Boffa, J. J., Massy, Z. A., Stengel, B. and Rossignol, P. | Association of plasma potassium with mortality and end-stage kidney disease in patients with chronic kidney disease under nephrologist care - The NephroTest study | 2017 | CVD mortality not stratified by sex |
| 812 | Waheeb, M. Q. | Investigation serum uric acid in cardiovascular mortality and all-cause mortality men and women in Al-Muthanna Province-Iraq | 2019 | CVD mortality not stratified by sex |
| 813 | Waheed, S., Malik, R., Waheed, S., Parashara, D. and Perez, J. | Association of QT interval with mortality by kidney function: Results from the National Health and Nutrition Examination Survey (NHANES) | 2017 | CVD mortality not stratified by sex |
| 814 | Waks, J. W., Tereshchenko, L. G. and Parekh, R. S. | Electrocardiographic predictors of mortality and sudden cardiac death in patients with end stage renal disease on hemodialysis | 2016 | CVD mortality not stratified by sex |
| 815 | Waldum-Grevbo, B., Leivestad, T., Reisaeter, A. V. and Os, I. | Impact of initial dialysis modality on mortality: a propensity-matched study | 2015 | CVD mortality not stratified by sex |
| 816 | Wang, A. Y., Wang, M., Lam, C. W., Chan, I. H., Lui, S. F. and Sanderson, J. E. | Heart failure with preserved or reduced ejection fraction in patients treated with peritoneal dialysis | 2013 | CVD mortality not stratified by sex |
| 817 | Wang, A. Y. M., Sea, M. M. M., Ng, K., Wang, M., Chan, I. H. S., Lam, C. W. K., Sanderson, J. E. and Woo, J. | Dietary Fiber Intake, Myocardial Injury, and Major Adverse Cardiovascular Events Among End-Stage Kidney Disease Patients: A Prospective Cohort Study | 2019 | CVD mortality not stratified by sex |
| 818 | Wang, A. Y. M., Wang, M., Woo, J., Lam, C. W. K., Li, P. K. T., Lui, S. F. and Sanderson, J. E. | Cardiac valve calcification as an important predictor for all-cause mortality and cardiovascular mortality in long-term peritoneal dialysis patients: A prospective study | 2003 | CVD mortality not stratified by sex |
| 819 | Wang, C., Li, Y., Zhang, J., Ye, Z., Zhang, Q., Ma, X., Peng, H. and Lou, T. | Prognostic Effect of Isolated Nocturnal Hypertension in Chinese Patients With Nondialysis Chronic Kidney Disease | 2016 | CVD mortality not stratified by sex |
| 820 | Wang, D., Liu, M., Hao, Z. and Tao, W. | Association between reduced kidney function and clinical outcomes after ischaemic stroke with atrial fibrillation | 2014 | CVD mortality not stratified by sex |
| 821 | Wang, H., Dai, D. F., Zeng, X. Z., Yang, J. F., Liu, D. P. and Yang, J. H. | Reduced estimated glomerular filtration rate and proteinuria are associated with increased cardiovascular events rate in octogenarian population | 2013 | CVD mortality not stratified by sex |
| 822 | Wang, J., Wang, F., Liu, S., Zhou, M., Zhang, L. and Zhao, M. | Reduced Kidney Function, Albuminuria, and Risks for All-cause and Cardiovascular Mortality in China: A Population-based Cohort Study | 2017 | CVD mortality not stratified by sex |
| 823 | Wang, J. Y., Wang, C. Y., Juang, S. Y., Huang, K. Y., Chou, P., Chen, C. W. and Lee, C. C. | Low socioeconomic status increases short-term mortality of acute myocardial infarction despite universal health coverage | 2014 | CVD mortality not stratified by sex |
| 824 | Wang, S. M., Cheng, S. Y., Chou, C. Y., Liu, J. H., Lin, H. H., Tseng, Y. H., Liu, Y. L., Chen, W. and Huang, C. C. | Association between mean arterial pressure and mortality in chronic hemodialysis patients | 2009 | CVD mortality not stratified by sex |
| 825 | Wang, Y., Guo, X., Li, J., Hu, D., Zhao, D., Ma, H., Mou, Q., Liu, J. and Xu, Y. | Predictive value of ankle-brachial index to all-cause mortality and cardiovascular mortality in Chinese patients with chronic kidney disease | 2012 | CVD mortality not stratified by sex |
| 826 | Wang, Y., Xiong, L., Xu, Q., Li, W., Peng, X., Shen, J., Qiu, Y., Yu, X. and Mao, H. | Association of left ventricular systolic dysfunction with mortality in incident peritoneal dialysis patients | 2018 | CVD mortality not stratified by sex |
| 827 | Wang, Y., Xu, Y., Hu, D., Guo, X., Zhao, D. and Li, J. | Joint association of ankle-brachial index and serum uric acid on the outcomes of six-year all-cause mortality and cardiovascular mortality in Chinese patients | 2012 | CVD mortality not stratified by sex |
| 828 | Wang, Z., Jiang, A., Wei, F. and Chen, H. | Cardiac valve calcification and risk of cardiovascular or all-cause mortality in dialysis patients: a meta-analysis | 2018 | CVD mortality not stratified by sex |
| 829 | Wanner, C., Krane, V., März, W., Olschewski, M., Mann, J. F. E., Ruf, G. and Ritz, E. | Atorvastatin in patients with type 2 diabetes mellitus undergoing hemodialysis | 2005 | CVD mortality not stratified by sex |
| 830 | Wasse, H., Speckman, R. A. and McClellan, W. M. | Arteriovenous fistula use is associated with lower cardiovascular mortality compared with catheter use among ESRD patients | 2008 | CVD mortality not stratified by sex |
| 831 | Wassertheurer, S. and Baumann, M. | Assessment of systolic aortic pressure and its association to all cause mortality critically depends on waveform calibration | 2015 | CVD mortality not stratified by sex |
| 832 | Wattanakit, K., Coresh, J., Muntner, P., Marsh, J. and Folsom, A. R. | Cardiovascular risk among adults with chronic kidney disease, with or without prior myocardial infarction | 2006 | CVD mortality not stratified by sex |
| 833 | Wei, T., Wang, M., Wang, M., Gan, L. Y. and Li, X. | Relationship of sRANKL level and vascular calcification score to cardiovascular events in maintenance hemodialysis patients | 2009 | CVD mortality not stratified by sex |
| 834 | Weidmann, Z. M., Breidthardt, T., Twerenbold, R., Zusli, C., Nowak, A., von Eckardstein, A., Erne, P., Rentsch, K., de Oliveira, M. T., Jr., Gualandro, D., Maeder, M. T., Rubini Gimenez, M., Pershyna, K., Stallone, F., Haas, L., Jaeger, C., Wildi, K., Puelacher, C., Honegger, U., Wagener, M., Wittmer, S., Schumacher, C., Krivoshei, L., Hillinger, P., Osswald, S. and Mueller, C. | Prediction of mortality using quantification of renal function in acute heart failure | 2015 | CVD mortality not stratified by sex |
| 835 | Weiner, D. E., Krassilnikova, M., Tighiouart, H., Salem, D. N., Levey, A. S. and Sarnak, M. J. | CKD classification based on estimated GFR over three years and subsequent cardiac and mortality outcomes: a cohort study | 2009 | CVD mortality not stratified by sex |
| 836 | Weiner, D. E., Tabatabai, S., Tighiouart, H., Elsayed, E., Bansal, N., Griffith, J., Salem, D. N., Levey, A. S. and Sarnak, M. J. | Cardiovascular outcomes and all-cause mortality: exploring the interaction between CKD and cardiovascular disease | 2006 | CVD mortality not stratified by sex |
| 837 | Weiner, D. E., Tighiouart, H., Stark, P. C., Amin, M. G., MacLeod, B., Griffith, J. L., Salem, D. N., Levey, A. S. and Sarnak, M. J. | Kidney disease as a risk factor for recurrent cardiovascular disease and mortality | 2004 | CVD mortality not stratified by sex |
| 838 | Weiner, D. E., Tighiouart, H., Vlagopoulos, P. T., Griffith, J. L., Salem, D. N., Levey, A. S. and Sarnak, M. J. | Effects of anemia and left ventricular hypertrophy on cardiovascular disease in patients with chronic kidney disease | 2005 | CVD mortality not stratified by sex |
| 839 | Weir, M. A., Dixon, S. N., Fleet, J. L., Roberts, M. A., Hackam, D. G., Oliver, M. J., Suri, R. S., Quinn, R. R., Ozair, S., Beyea, M. M., Kitchlu, A. and Garg, A. X. | beta-Blocker dialyzability and mortality in older patients receiving hemodialysis | 2015 | CVD mortality not stratified by sex |
| 840 | Wen, C. P., Matsushita, K., Coresh, J., Iseki, K., Islam, M., Katz, R., McClellan, W., Peralta, C. A., Wang, H., de Zeeuw, D., Astor, B. C., Gansevoort, R. T., Levey, A. S. and Levin, A. | Relative risks of chronic kidney disease for mortality and end-stage renal disease across races are similar | 2014 | CVD mortality not stratified by sex |
| 841 | Wetmore, J. B., Li, S., Yan, H., Xu, H., Peng, Y., Sinsakul, M. V., Liu, J. and Gilbertson, D. T. | Predialysis anemia management and outcomes following dialysis initiation: A retrospective cohort analysis | 2018 | CVD mortality not stratified by sex |
| 842 | Winkelmayer, W. C., Hurley, M. P., Liu, J. and Brookhart, M. A. | Altitude and the risk of cardiovascular events in incident US dialysis patients | 2012 | CVD mortality not stratified by sex |
| 843 | Winkelmayer, W. C., Kramar, R., Sunder-Plassmann, G. and Födinger, M. | Effects of single-nucleotide polymorphisms in MTHFR and MTRR on mortality and allograft loss in kidney transplant recipients | 2005 | CVD mortality not stratified by sex |
| 844 | Winkler, K., Hoffmann, M. M., Krane, V., Marz, W., Drechsler, C. and Wanner, C. | Apolipoprotein E genotype predicts cardiovascular endpoints in dialysis patients with type 2 diabetes mellitus | 2010 | CVD mortality not stratified by sex |
| 845 | Wright, J. R., Shurrab, A. E., Cheung, C., Waldek, S., O'Donoghue, D. J., Foley, R. N., Mamtora, H. and Kalra, P. A. | A prospective study of the determinants of renal functional outcome and mortality in atherosclerotic renovascular disease | 2002 | CVD mortality not stratified by sex |
| 846 | Wu, C. C., Liou, H. H., Su, P. F., Chang, M. Y., Wang, H. H., Chen, M. J. and Hung, S. Y. | Abdominal obesity is the most significant metabolic syndrome component predictive of cardiovascular events in chronic hemodialysis patients | 2011 | CVD mortality not stratified by sex |
| 847 | Wu, C. K., Wu, C. L., Lin, C. H., Leu, J. G., Kor, C. T. and Tarng, D. C. | Association of vascular access flow with short-term and long-term mortality in chronic haemodialysis patients: a retrospective cohort study | 2017 | CVD mortality not stratified by sex |
| 848 | Wu, D. Y., Shinaberger, C. S., Regidor, D. L., McAllister, C. J., Kopple, J. D. and Kalantar-Zadeh, K. | Association between serum bicarbonate and death in hemodialysis patients: is it better to be acidotic or alkalotic? | 2006 | CVD mortality not stratified by sex |
| 849 | Wu, H., Xiong, L., Xu, Q., Wu, J., Huang, R., Guo, Q., Mao, H., Yu, X. and Yang, X. | Higher serum triglyceride to high-density lipoprotein cholesterol ratio was associated with increased cardiovascular mortality in female patients on peritoneal dialysis | 2015 | CVD mortality not stratified by sex |
| 850 | Wu, H. C., Lee, L. C. and Wang, W. J. | Associations among time-average mineral values, mortality and cardiovascular events in hemodialysis patients | 2015 | CVD mortality not stratified by sex |
| 851 | Wu, M., Wu, H., Huang, X., Ye, H., Huang, F., Yu, X. and Yang, X. | Associations between serum mineral metabolism parameters and mortality in patients on peritoneal dialysis | 2018 | CVD mortality not stratified by sex |
| 852 | Xia, X., He, F., Wu, X., Peng, F., Huang, F. and Yu, X. | Relationship between serum uric acid and all-cause and cardiovascular mortality in patients treated with peritoneal dialysis | 2014 | CVD mortality not stratified by sex |
| 853 | Xie, Q., Ge, X., Da, Shang, Li, Y., Yan, H., Tian, J., Hao, C. M. and Zhu, T. | Coronary artery calcification score as a predictor of all-cause mortality and cardiovascular outcome in peritoneal dialysis patients | 2016 | CVD mortality not stratified by sex |
| 854 | Xie, X., Zhang, X., Xiang, S., Yan, X., Huang, H., Tian, Y., Shou, Z. and Chen, J. | Association of very low-density lipoprotein cholesterol with all-cause and cardiovascular mortality in peritoneal dialysis | 2017 | CVD mortality not stratified by sex |
| 855 | Xiong, L., Cao, S., Xu, F., Zhou, Q., Fan, L., Xu, Q., Yu, X. and Mao, H. | Association of body mass index and body mass index change with mortality in incident peritoneal dialysis patients | 2015 | CVD mortality not stratified by sex |
| 856 | Xiong, L., Fan, L., Xu, Q., Zhou, Q., Li, H., Peng, X., Yang, Y., Wang, Y., Yu, X. and Mao, H. | Faster Transport Status and Mortality in Anuric Patients Undergoing Continuous Ambulatory Peritoneal Dialysis | 2015 | CVD mortality not stratified by sex |
| 857 | Xu, Q., Guo, H., Cao, S., Zhou, Q., Chen, J., Su, M., Chen, S., Jiang, S., Shi, X. and Wen, Y. | Associations of vitamin K status with mortality and cardiovascular events in peritoneal dialysis patients | 2019 | CVD mortality not stratified by sex |
| 858 | Xu, Q., Xiong, L., Fan, L., Xu, F., Yang, Y., Li, H., Peng, X., Cao, S., Zheng, Z., Yang, X., Yu, X. and Mao, H. | Association of Pulmonary Hypertension with Mortality in Incident Peritoneal Dialysis Patients | 2015 | CVD mortality not stratified by sex |
| 859 | Xu, Q., Xu, F., Fan, L., Xiong, L., Li, H., Cao, S., Lin, X., Zheng, Z., Yu, X. and Mao, H. | Serum potassium levels and its variability in incident peritoneal dialysis patients: Associations with mortality | 2014 | CVD mortality not stratified by sex |
| 860 | Xu, R., Han, Q. F., Zhu, T. Y., Ren, Y. P., Chen, J. H., Zhao, H. P., Chen, M. H., Dong, J., Wang, Y., Hao, C. M., Zhang, R., Zhang, X. H., Wang, M., Tian, N. and Wang, H. Y. | Impact of individual and environmental socioeconomic status on peritoneal dialysis outcomes: a retrospective multicenter cohort study | 2012 | CVD mortality not stratified by sex |
| 861 | Yakupoglu, U., Ozdemir, F. N., Arat, Z., Haberal, A., Agca, E. and Bilgin, N. | Can troponin-I predict cardiovascular mortality due to myocardial injury in hemodialysis patients? | 2002 | CVD mortality not stratified by sex |
| 862 | Yamaguchi, S., Gohda, T., Gotoh, H., Omote, K., Furukawa, M., Ishikawa, Y. and Tomino, Y. | Factors associated with cardiovascular death and events in patients with end stage renal disease | 2013 | CVD mortality not stratified by sex |
| 863 | Yamamoto, T., Shoji, S., Yamakawa, T., Wada, A., Suzuki, K., Iseki, K. and Tsubakihara, Y. | Predialysis and Postdialysis pH and Bicarbonate and Risk of All-Cause and Cardiovascular Mortality in Long-term Hemodialysis Patients | 2015 | CVD mortality not stratified by sex |
| 864 | Yamashita, K., Mizuiri, S., Nishizawa, Y., Shigemoto, K., Doi, S. and Masaki, T. | Addition of Novel Biomarkers for Predicting All-Cause and Cardiovascular Mortality in Prevalent Hemodialysis Patients | 2018 | CVD mortality not stratified by sex |
| 865 | Yamashita, Y., Takagi, D., Hamatani, Y., Iguchi, M., Masunaga, N., Esato, M., Chun, Y. H., Itoh, H., Nishimura, M., Wada, H. and et al. | Clinical characteristics and outcomes of dialysis patients with atrial fibrillation: the Fushimi AF Registry | 2016 | CVD mortality not stratified by sex |
| 866 | Yang, J. G., Li, J., Lu, C., Hasimu, B., Yang, Y. and Hu, D. | Chronic kidney disease, all-cause mortality and cardiovascular mortality among Chinese patients with established cardiovascular disease | 2010 | CVD mortality not stratified by sex |
| 867 | Yang, X., Zhang, H., Shi, Y., Yu, Z., Yan, H., Ni, Z., Qian, J. and Fang, W. | Association of serum angiopoietin-2 with malnutrition, inflammation, atherosclerosis and valvular calcification syndrome and outcome in peritoneal dialysis patients: A prospective cohort study | 2018 | CVD mortality not stratified by sex |
| 868 | Yang, Z. K., Han, Q. F., Zhu, T. Y., Ren, Y. P., Chen, J. H., Zhao, H. P., Chen, M. H., Dong, J., Wang, Y., Hao, C. M., Zhang, R., Zhang, X. H., Wang, M., Tian, N. and Wang, H. Y. | The associations between the family education and mortality of patients on peritoneal dialysis | 2014 | CVD mortality not stratified by sex |
| 869 | Yen, T. H., Lin, J. L., Lin-Tan, D. T. and Hsu, C. W. | Association between body mass and mortality in maintenance hemodialysis patients | 2010 | CVD mortality not stratified by sex |
| 870 | Yilmaz, H., Celik, H. T., Gurel, O. M., Bilgic, M. A., Namuslu, M., Bozkurt, H., Ayyildiz, A., Inan, O., Bavbek, N. and Akcay, A. | Increased serum levels of GDF-15 associated with mortality and subclinical atherosclerosis in patients on maintenance hemodialysis | 2015 | CVD mortality not stratified by sex |
| 871 | Yin, Z., Fang, Z., Yang, M., Du, X., Nie, B. and Gao, K. | Predictive value of serum uric acid levels on mortality in acute coronary syndrome patients with chronic kidney disease after drug-eluting stent implantation | 2013 | CVD mortality not stratified by sex |
| 872 | Yoda, S., Nakanishi, K., Tano, A., Kasamaki, Y., Kunimoto, S., Matsumoto, N., Sato, Y. and Hirayama, A. | Risk stratification of cardiovascular events in patients at all stages of chronic kidney disease using myocardial perfusion SPECT | 2012 | CVD mortality not stratified by sex |
| 873 | Yoo, H. H., Martin, L. C., Kochi, A. C., Rodrigues-Telini, L. S., Barretti, P., Caramori, J. T., Matsubara, B. B., Zannati-Bazan, S. G., Franco, R. J. and Queluz, T. T. | Could albumin level explain the higher mortality in hemodialysis patients with pulmonary hypertension? | 2012 | CVD mortality not stratified by sex |
| 874 | Yoshihara, F., Horio, T., Nakamura, S., Yoshii, M., Ogata, C., Nakahama, H., Inenaga, T., Kangawa, K. and Kawano, Y. | Adrenomedullin reflects cardiac dysfunction, excessive blood volume, and inflammation in hemodialysis patients | 2005 | CVD mortality not stratified by sex |
| 875 | Yoshitomi, R., Nakayama, M., Sakoh, T., Fukui, A., Katafuchi, E., Seki, M., Tsuda, S., Nakano, T., Tsuruya, K. and Kitazono, T. | High neutrophil/lymphocyte ratio is associated with poor renal outcomes in Japanese patients with chronic kidney disease | 2019 | CVD mortality not stratified by sex |
| 876 | Yu, D., Cai, Y., Chen, Y., Chen, T., Qin, R., Simmons, D. and Zhao, Z. | Development and validation of risk prediction models for cardiovascular mortality in Chinese people initialising peritoneal dialysis: A cohort study | 2018 | CVD mortality not stratified by sex |
| 877 | Yu, L., Li, H. and Wang, S. X. | Serum Magnesium and Mortality in Maintenance Hemodialysis Patients | 2017 | CVD mortality not stratified by sex |
| 878 | Yu, T. M., Chuang, Y. W. and Chen, C. H. | Young-adult polycystic kidney disease is significantly associated with major cardiovascular complications | 2017 | CVD mortality not stratified by sex |
| 879 | Yu, Z. Z., Ni, Z. H., Gu, L. Y., Lin, A. W., Fang, W., Yao, Q., Lindholm, B. and Qian, J. Q. | Adiponectin is related to carotid artery plaque and a predictor of cardiovascular outcome in a cohort of non-diabetic peritoneal dialysis patients | 2008 | CVD mortality not stratified by sex |
| 880 | Yun, Y. S., Choi, S. J., Lee, J. Y., Kim, Y. S., Yoon, S. A., Park, S. C., Shin, O. R., Jang, E. J. and Kim, Y. O. | Impact of arterial microcalcification of the vascular access on cardiovascular mortality in hemodialysis patients | 2014 | CVD mortality not stratified by sex |
| 881 | Zamora, E., Lupon, J., de Antonio, M., Galan, A., Domingo, M., Urrutia, A., Troya, M. and Bayes-Genis, A. | Renal function largely influences Galectin-3 prognostic value in heart failure | 2014 | CVD mortality not stratified by sex |
| 882 | Zhan, X., Chen, Y., Yan, C., Liu, S., Deng, L., Yang, Y., Qiu, P., Pan, D., Zeng, B. and Chen, Q. | Apolipoprotein B/apolipoprotein A1 ratio and mortality among incident peritoneal dialysis patients | 2018 | CVD mortality not stratified by sex |
| 883 | Zhang, A., Wang, S., Li, H., Yang, J. and Wu, H. | Aortic arch calcification and risk of cardiovascular or all-cause and mortality in dialysis patients: A meta-analysis | 2016 | CVD mortality not stratified by sex |
| 884 | Zhang, J., Jiang, H., Sun, M. and Chen, J. | Association between periodontal disease and mortality in people with CKD: A meta-analysis of cohort studies | 2017 | CVD mortality not stratified by sex |
| 885 | Zhang, Q., Ren, H., Xie, J., Li, X., Huang, X. and Chen, N. | Causes of death in peritoneal dialysis patients with different kidney diseases and comorbidities: a retrospective clinical analysis in a Chinese center | 2014 | CVD mortality not stratified by sex |
| 886 | Zhang, X., Jing, J., Zhao, X., Liu, L., Wang, C., Pan, Y., Meng, X., Wang, Y. and Wang, Y. | Statin Use during Hospitalization and Short-Term Mortality in Acute Ischaemic Stroke with Chronic Kidney Disease | 2018 | CVD mortality not stratified by sex |
| 887 | Zhang, X., Yu, D., Cai, Y., Shang, J., Qin, R., Tian, X., Zhao, Z. and Simmons, D. | Derivation and Validation of Risk Scores to Predict Cerebrovascular Mortality Among Incident Peritoneal Dialysis Patients | 2018 | CVD mortality not stratified by sex |
| 888 | Zhang, X., Yu, D., Cai, Y., Shang, J., Qin, R., Xiao, J., Tian, X., Zhao, Z. and Simmons, D. | Dose-Response Between Cardiovascular Risk Factors and Cardiovascular Mortality Among Incident Peritoneal Dialysis Patients | 2018 | CVD mortality not stratified by sex |
| 889 | Zhang, Y. F., Wang, Q., Su, Y. Y., Yang, S., Guo, J., Luo, J., Tang, J. M. and Li, H. Y. | Potassium supplementation and long-term outcomes in chronic peritoneal dialysis patients with end-stage renal disease: a propensity score matching study | 2016 | CVD mortality not stratified by sex |
| 890 | Zhang, Z., Shen, B., Cao, X., Liu, Z., Chen, X., Nie, Y., Yu, J., Zou, J. and Ding, X. | Increased Soluble Suppression of Tumorigenicity 2 Level Predicts All-Cause and Cardiovascular Mortality in Maintenance Hemodialysis Patients: A Prospective Cohort Study | 2017 | CVD mortality not stratified by sex |
| 891 | Zhao, X., Wang, M. and Zuo, L. | Early mortality risk in incident Chinese hemodialysis patients: a retrospective cohort study | 2017 | CVD mortality not stratified by sex |
| 892 | Zheng, Z., Shi, H., Jia, J., Li, D. and Lin, S. | Vitamin D supplementation and mortality risk in chronic kidney disease: A meta-analysis of 20 observational studies | 2013 | CVD mortality not stratified by sex |
| 893 | Zhu, J. G., Chen, J. B., Cheng, B. C., Lee, C. H., Long, G. and Chien, Y. S. | Association between Extreme Values of Markers of Chronic Kidney Disease: Mineral and Bone Disorder and 5-Year Mortality among Prevalent Hemodialysis Patients | 2018 | CVD mortality not stratified by sex |
| 894 | Zhu, J. G., Cheng, B. C., Lee, W. C., Li, L. C., Lee, C. H., Long, G. and Chen, J. B. | Serum Alkaline Phosphatase Levels are Not Associated with Increased Death Risk in Prevalent Hemodialysis Patients: 5-Year Experience in a Single Hemodialysis Center | 2016 | CVD mortality not stratified by sex |
| 895 | Zhu, M., Dou, L., Zhu, M., Liu, S., Zhan, Y., Lu, J., Ni, Z., Qian, J., Cai, H. and Zhang, W. | Variability of serum phosphorus and its association with mortality among hemodialysis patients | 2018 | CVD mortality not stratified by sex |
| 896 | Zhu, Y., Peng, F., Chen, Y., Chen, W., Zhou, W., Li, P., Niu, H. and Long, H. | Mean platelet volume/platelet count ratio and mortality in patients on peritoneal dialysis | 2018 | CVD mortality not stratified by sex |
| 897 | Zitt, E., Sturm, G., Kronenberg, F., Neyer, U., Knoll, F., Lhotta, K. and Weiss, G. | Iron supplementation and mortality in incident dialysis patients: An observational study | 2014 | CVD mortality not stratified by sex |
| 898 | Zoccali, C., Bode-Boger, S., Mallamaci, F., Benedetto, F., Tripepi, G., Malatino, L., Cataliotti, A., Bellanuova, I., Fermo, I., Frolich, J. and Boger, R. | Plasma concentration of asymmetrical dimethylarginine and mortality in patients with end-stage renal disease: a prospective study | 2001 | CVD mortality not stratified by sex |
| 899 | Zoccali, C., Mallamaci, F., Benedetto, F. A., Tripepi, G., Parlongo, S., Cataliotti, A., Cutrupi, S., Giacone, G., Bellanuova, I., Cottini, E. and Malatino, L. S. | Cardiac natriuretic peptides are related to left ventricular mass and function and predict mortality in dialysis patients | 2001 | CVD mortality not stratified by sex |
| 900 | Zoccali, C., Mallamaci, F., Tripepi, G., Benedetto, F. A., Cutrupi, S., Parlongo, S., Malatino, L. S., Bonanno, G., Seminara, G., Rapisarda, F., Fatuzzo, P., Buemi, M., Nicocia, G., Tanaka, S., Ouchi, N., Kihara, S., Funahashi, T. and Matsuzawa, Y. | Adiponectin, metabolic risk factors, and cardiovascular events among patients with end-stage renal disease | 2002 | CVD mortality not stratified by sex |
| 901 | Zoccali, C., Mallamaci, F., Tripepi, G., Cutrupi, S., Parlongo, S., Malatino, L. S., Bonanno, G., Rapisarda, F., Fatuzzo, P., Seminara, G., Stancanelli, B., Nicocia, G. and Buemi, M. | Fibrinogen, mortality and incident cardiovascular complications in end-stage renal failure | 2003 | CVD mortality not stratified by sex |
| 902 | Zoccali, C., Postorino, M., Marino, C., Pizzini, P., Cutrupi, S. and Tripepi, G. | Waist circumference modifies the relationship between the adipose tissue cytokines leptin and adiponectin and all-cause and cardiovascular mortality in haemodialysis patients | 2011 | CVD mortality not stratified by sex |
| 903 | Zoungas, S., Cameron, J. D., Kerr, P. G., Wolfe, R., Muske, C., McNeil, J. J. and McGrath, B. P. | Association of carotid intima-medial thickness and indices of arterial stiffness with cardiovascular disease outcomes in CKD | 2007 | CVD mortality not stratified by sex |
| 904 | Zoungas, S., Lui, M., Kerr, P. G., Teede, H. J., McNeil, J. J., McGrath, B. P. and Polkinghorne, K. R. | Advanced chronic kidney disease, cardiovascular events and the effect of diabetes: data from the Atherosclerosis and Folic Acid Supplementation Trial | 2011 | CVD mortality not stratified by sex |
| 905 | Zoungas, S., McGrath, B. P., Branley, P., Kerr, P. G., Muske, C., Wolfe, R., Atkins, R. C., Nicholls, K., Fraenkel, M., Hutchison, B. G. and et al. | Cardiovascular morbidity and mortality in the Atherosclerosis and Folic Acid Supplementation Trial (ASFAST) in chronic renal failure: a multicenter, randomized, controlled trial | 2006 | CVD mortality not stratified by sex |
| 942 | Ulusoy, S., Ozkan, G., Guvercin, B. and Yavuz, A. | The Relation Between Variability of Intact Parathyroid Hormone, Calcium, and Cardiac Mortality in Hemodialysis Patients | 2016 | CVD mortality not stratified by sex |
| 914 | Assimon MM, Brookhart MA, Fine JP, Heiss G, Layton JB, Flythe JE | A Comparative Study of Carvedilol Versus Metoprolol Initiation and 1-Year Mortality Among Individuals Receiving Maintenance Hemodialysis. | 2018 | Ineligible study design |
| 915 | Depner T, Daugirdas J, Greene T, Allon M, Beck G, Chumlea C, et al. | Dialysis dose and the effect of gender and body size on outcome in the HEMO Study | 2004 | Ineligible study design |
| 916 | Fernández-Juárez G, Luño J, Barrio V, de Vinuesa SG, Praga M, Goicoechea M, et al. | 25 (OH) vitamin D levels and renal disease progression in patients with type 2 diabetic nephropathy and blockade of the renin-angiotensin system | 2013 | Ineligible study design |
| 917 | Cai Q, Serrano R, Kalyanasundaram A, Shirani J. | A preoperative echocardiographic predictive model for assessment of cardiovascular outcome after renal transplantation. Journal of the American Society of Echocardiography. 2010;23(5):560-6. | 2010 | Ineligible study design |
| 918 | Sapir-Pichhadze R, Tinckam KJ, Laupacis A, Logan AG, Beyene J, Kim SJ. | Immune Sensitization and Mortality in Wait-Listed Kidney Transplant Candidates. 2016;1(2):570-8. | 2016 | Ineligible study design |
| 919 | Lanaro E, Caixeta A, Soares JA, Alves CMR, Barbosa AHP, Souza JAM, et al. | Influence of gender on the risk of death and adverse events in patients with acute myocardial infarction undergoing pharmacoinvasive strategy. Journal of Thrombosis and Thrombolysis. 2014;38(4):510-6. | 2014 | Ineligible study design |
| 920 | Molnar MZ, Gosmanova EO, Sumida K, Potukuchi PK, Lu JL, Jing J, et al. | Predialysis Cardiovascular Disease Medication Adherence and Mortality After Transition to Dialysis. American Journal of Kidney Diseases. 2016;68(4):609-18. | 2016 | Ineligible study design |
| 921 | Soohoo M, Streja E, Obi Y, Rhee CM, Gillen DL, Sumida K, et al. | Predialysis Kidney Function and Its Rate of Decline Predict Mortality and Hospitalizations After Starting Dialysis. Mayo Clinic Proceedings. 2018;93(8):1074-85. | 2018 | Ineligible study design |
| 922 | Sumida K, Molnar MZ, Potukuchi PK, Thomas F, Lu JL, Yamagata K, et al. | Pre-end-stage renal disease visit-To-visit systolic blood pressure variability and post-end-stage renal disease mortality in incident dialysis patients. Journal of Hypertension. 2017;35(9):1816-24. | 2017 | Ineligible study design |
| 923 | Kramann R, Erpenbeck J, Schneider RK, Rohl AB, Hein M, Brandenburg VM, et al. | Speckle tracking echocardiography detects uremic cardiomyopathy early and predicts cardiovascular mortality in ESRD. Journal of the American Society of Nephrology. 2014;25(10):2351-65. | 2014 | Ineligible study design |
| 906 | Euctr, C. Z. | A randomised, double blind, placebo controlled, parallel group study to assess the effect of the endothelin receptor antagonist avosentan on time to doubling of serum creatinine, end stage renal disease or death in patients with type 2 diabetes mellitus and diabetic nephropathy - ASCEND | 2005 | Ineligible study population |
| 907 | Hirata, A., Okamura, T., Sugiyama, D., Kuwabara, K., Kadota, A., Fujiyoshi, A., Miura, K., Okuda, N., Ohkubo, T., Okayama, A. and Ueshima, H. | Impacts of chronic kidney disease and diabetes on cardiovascular mortality in a general Japanese population: A 20-year follow-up of the NIPPON DATA90 study | 2017 | Ineligible study population |
| 908 | Kacso, I. M., Potra, A. R., Bondor, C. I., Moldovan, D., Rusu, C., Patiu, I. M., Racasan, S., Orasan, R., Moldovan, R., Ghigolea, B., Vladutiu, D., Spanu, C., Nita, C. and Rusu, A. | ESAM predicts cardiovascular mortality in diabetic hemodialysis patients | 2015 | Ineligible study population |
| 909 | Peng, F., Xia, X., He, F., Li, Z., Huang, F. and Yu, X. | The effect of glycated hemoglobin and albumin-corrected glycated serum protein on mortality in diabetic patients receiving continuous peritoneal dialysis | 2015 | Ineligible study population |
| 910 | Poulter, N., Mann, J., Fonseca, V., Mosenzon, O., Raz, I., Frimer-Larsen, H., Von Scholten, B. and Idorn, T. | Liraglutide reduces major cardiovascular events in patients with chronic kidney disease: results from the LEADER trial | 2018 | Ineligible study population |
| 911 | Salinero-Fort, M. A., San Andres-Rebollo, F. J., de Burgos-Lunar, C., Abanades-Herranz, J. C., Carrillo-de-Santa-Pau, E., Chico-Moraleja, R. M., Jimenez-Garcia, R., Lopez-de-Andres, A. and Gomez-Campelo, P. | Cardiovascular and all-cause mortality in patients with type 2 diabetes mellitus in the MADIABETES Cohort Study: Association with chronic kidney disease | 2016 | Ineligible study population |
| 912 | Seferovic, J. P., Pfeffer, M. A., Claggett, B., Desai, A. S., de Zeeuw, D., Haffner, S. M., McMurray, J. J. V., Parving, H. H., Solomon, S. D. and Chaturvedi, N. | Three-question set from Michigan Neuropathy Screening Instrument adds independent prognostic information on cardiovascular outcomes: analysis of ALTITUDE trial | 2018 | Ineligible study population |
| 913 | Wanner, C., Lachin, J. M., Inzucchi, S. E., Fitchett, D., Mattheus, M., George, J., Woerle, H. J., Broedl, U. C., von Eynatten, M. and Zinman, B. | Empagliflozin and Clinical Outcomes in Patients With Type 2 Diabetes Mellitus, Established Cardiovascular Disease, and Chronic Kidney Disease | 2018 | Ineligible study population |
| 924 | Navaneethan, S. D. | Cause-specific deaths in non-dialysis-dependent CKD | 2015 | Ineligible study population |
| 925 | Kato, A. | Association between seroprevalence of anti-chlamydial antibodies and long-term cardiovascular mortality in chronic hemodialysis patients | 2006 | Ineligible study population |
| 926 | Caravaca, F., Chavez, E., Alvarado, R., Garcia-Pino, G. and Luna, E. | Sudden cardiac death in non-dialysis chronic kidney disease patients | 2016 | Not published in English |
| 927 | Deschamps, A., Grunfeld, J. P., Drueke, T., Zingraff, J. and Jungers, P. | Arterial hypertension and mortality due to cardiovascular complications in patients on chronic hemodialysis | 1978 | Not published in English |
| 928 | El Hadj Othmane, T., Kiss, I., Nemcsik, J., Fekete, C. B., Deak, G., Egresits, J., Fodor, E., Nemeth, K. Z., Szabo, T., Szathmari, M. and Tisler, A. | Significance of arterial stiffness parameters for predicting cardiovascular mortality in hemodialysis patients: a prospective cohort study | 2010 | Not published in English |
| 929 | Heras, M., Fernandez-Reyes, M. J., Sanchez, R., Guerrero, M. T., Molina, A., Rodriguez, M. A. and Alvarez-Ude, F. | Elderly patients with chronic kidney disease: outcomes after 5 years of follow-up | 2012 | Not published in English |
| 930 | Meng, L., Ding, W. H., Shi, L. B., Jiang, J., Liu, Z. P. and Gong, Y. J. | Cardiovascular events in patients with chronic kidney disease | 2009 | Not published in English |
| 931 | Prado-Uribe, M. D. C., Ventura, M. D., Avila-Diaz, M., Mora, C. J., Mendez-Duran, A., Villanueva-Noches, D., Cisneros, A., Ilabaca, B., Cueto-Manzano, A., Garcia-Contreras, F., Lindholm, B., Garcia-Lopez, E. and Paniagua, R. | Low triiodothyronine is associated with elevation of N-terminal pro-brain natriuretic peptide (NT-proBNP) and mortality in dialysis patients | 2017 | Not published in English |
| 932 | Sanchez-Perales, C., Vazquez Ruiz de Castroviejo, E., Garcia-Cortes, M. J., Biechy Mdel, M., Gil-Cunquero, J. M., Borrego-Hinojosa, J., del Barrio, P. P., Borrego-Utiel, F. and Liebana, A. | Valvular calcifications at the start of dialysis predict the onset of cardiovascular events in the course of follow-up | 2015 | Not published in English |
| 933 | Schiele, F. | Chronic renal failure: an independent factor of mortality after myocardial infarction | 2005 | Not published in English |
| 934 | Terrier-Lenglet, A., Nollet, A., Liabeuf, S., Barreto, D. V., Brazier, M., Lemke, H. D., Vanholder, R., Choukroun, G. and Massy, Z. A. | Plasma malondialdehyde may not predict mortality in patient with chronic kidney disease | 2011 | Not published in English |
| 935 | Ozkan, G., Ulusoy, S., Mentese, A., Guvercin, B., Karahan, S. C., Yavuz, A., Altay, D. U. and Ocal, M. | Can be galectin-3 a novel marker in determining mortality in hemodialysis patients? | 2015 | Other (Comments, letters) |
| 936 | Einollahi, B., Taghipour, M. and Motalebi, M. | Re: Association of leptin with mortality in patients on maintenance hemodialysis: a prospective study | 2014 | Other (Comments, letters) |
| 937 | Gholamrezaei, A., Amra, B. and Mortazavi, M. | Cardiovascular risk and mortality in end-stage renal disease patients with restless legs syndrome; need for further investigation and looking for underlying mechanisms | 2013 | Other (Comments, letters) |
| 938 | Kannan, A., Poongkunran, C. and Balamuthusamy, S. | Effect of spironolactone in CV Mortality in hemodialysis patients | 2014 | Other (Comments, letters) |
| 939 | Olszewska, M., Schwermer, K., Hoppe, K., Misian, M., Baum, E., Pawlaczyk, K. and Oko, A. | Overhydration as a modifiable cardiovascular and all-cause mortality risk factor in hemodialysis patients | 2017 | Other (Comments, letters) |
| 940 | Providencia, R., Barra, S. and Paiva, L. | Chronic renal disease is associated with stroke and thromboembolism in atrial fibrillation independently from gender | 2013 | Other (Comments, letters) |
| 941 | Sato, A. | Predicting cardiac and all-cause death in asymptomatic patients on hemodialysis: Importance of training in interpretation of β-methyl iodophenyl-pentadecanoic acid single-photon emission computed tomography (BMIPP SPECT) imaging | 2014 | Other (Comments, letters) |
| 943 | Chowdhury, E. K., Langham, R. G., Ademi, Z., Owen, A., Krum, H., Wing, L. M., Nelson, M. R. and Reid, C. M. | Comparison of predictive performance of renal function estimation equations for all-cause and cardiovascular mortality in an elderly hypertensive population | 2015 | Population data from before 2004 |
| 944 | Global Burden of Metabolic Risk Factors for Chronic Diseases Collaboration. | Cardiovascular disease, chronic kidney disease, and diabetes mortality burden of cardiometabolic risk factors from 1980 to 2010: a comparative risk assessment | 2014 | Population data from before 2004 |
| 945 | Adragao, T., Pires, A., Lucas, C., Birne, R., Magalhaes, L., Goncalves, M. and Negrao, A. P. | A simple vascular calcification score predicts cardiovascular risk in haemodialysis patients | 2004 | Population data from before 2004 |
| 946 | Akbas, T., Mulazimoglu, L., Aksu, B. and Akoglu, E. | A prospective study: inflammation, infection and comorbidity in patients on long-term dialysis | 2014 | Population data from before 2004 |
| 947 | Amar, J., Vernier, I., Rossignol, E., Bongard, V., Arnaud, C., Conte, J. J., Salvador, M. and Chamontin, B. | Nocturnal blood pressure and 24-hour pulse pressure are potent indicators of mortality in hemodialysis patients | 2000 | Population data from before 2004 |
| 948 | Avram, M. M., Sreedhara, R., Patel, N., Chattopadhyay, J., Thu, T. and Fein, P. | Is an elevated level of serum lipoprotein (a) a risk factor for cardiovascular disease in CAPD patients? | 1996 | Population data from before 2004 |
| 949 | Beddhu, S., Baird, B., Ma, X., Cheung, A. K. and Greene, T. | Serum alkaline phosphatase and mortality in hemodialysis patients | 2010 | Population data from before 2004 |
| 950 | Beddhu, S., Pappas, L. M., Ramkumar, N. and Samore, M. | Effects of body size and body composition on survival in hemodialysis patients | 2003 | Population data from before 2004 |
| 951 | Benedetto, F. A., Mallamaci, F., Tripepi, G. and Zoccali, C. | Prognostic value of ultrasonographic measurement of carotid intima media thickness in dialysis patients | 2001 | Population data from before 2004 |
| 952 | Blacher, J., Guerin, A. P., Pannier, B., Marchais, S. J., Safar, M. E. and London, G. M. | Impact of aortic stiffness on survival in end-stage renal disease | 1999 | Population data from before 2004 |
| 953 | Blacher, J., Pannier, B., Guerin, A. P., Marchais, S. J., Safar, M. E. and London, G. M. | Carotid arterial stiffness as a predictor of cardiovascular and all-cause mortality in end-stage renal disease | 1998 | Population data from before 2004 |
| 954 | Bloembergen, W. E., Stannard, D. C., Port, F. K., Wolfe, R. A., Pugh, J. A., Jones, C. A., Greer, J. W., Golper, T. A. and Held, P. J. | Relationship of dose of hemodialysis and cause-specific mortality | 1996 | Population data from before 2004 |
| 955 | Boger, C. A., Fischereder, M., Deinzer, M., Aslanidis, C., Schmitz, G., Stubanus, M., Banas, B., Kruger, B., Riegger, G. A. and Kramer, B. K. | RANTES gene polymorphisms predict all-cause and cardiac mortality in type 2 diabetes mellitus hemodialysis patients | 2005 | Population data from before 2004 |
| 956 | Boulier, A., Jaussent, I., Terrier, N., Maurice, F., Rivory, J. P., Chalabi, L., Boularan, A. M., Delcourt, C., Dupuy, A. M., Canaud, B. and Cristol, J. P. | Measurement of circulating troponin Ic enhances the prognostic value of C-reactive protein in haemodialysis patients | 2004 | Population data from before 2004 |
| 957 | Braatvedt, G. D., Rosie, B., Bagg, W. and Collins, J. | Current and former smoking increases mortality in patients on peritoneal dialysis | 2006 | Population data from before 2004 |
| 958 | Brown, J. H., Hunt, L. P., Vites, N. P., Short, C. D., Gokal, R. and Mallick, N. P. | Comparative mortality from cardiovascular disease in patients with chronic renal failure | 1994 | Population data from before 2004 |
| 959 | Carrero, J. J., de Jager, D. J., Verduijn, M., Ravani, P., De Meester, J., Heaf, J. G., Finne, P., Hoitsma, A. J., Pascual, J., Jarraya, F., Reisaeter, A. V., Collart, F., Dekker, F. W. and Jager, K. J. | Cardiovascular and noncardiovascular mortality among men and women starting dialysis | 2011 | Population data from before 2004 |
| 960 | Carrero, J. J., Nakashima, A., Qureshi, A. R., Lindholm, B., Heimburger, O., Barany, P. and Stenvinkel, P. | Protein-energy wasting modifies the association of ghrelin with inflammation, leptin, and mortality in hemodialysis patients | 2011 | Population data from before 2004 |
| 961 | Chan, K. E., Ikizler, T. A., Gamboa, J. L., Yu, C., Hakim, R. M. and Brown, N. J. | Combined angiotensin-converting enzyme inhibition and receptor blockade associate with increased risk of cardiovascular death in hemodialysis patients | 2011 | Population data from before 2004 |
| 962 | Chang, T. I., Nam, J. Y., Shin, S. K. and Kang, E. W. | Low Triiodothyronine Syndrome and Long-Term Cardiovascular Outcome in Incident Peritoneal Dialysis Patients | 2015 | Population data from before 2004 |
| 963 | Chen, Y. H., Hung, S. C. and Tarng, D. C. | Serum bilirubin links UGT1A1*28 polymorphism and predicts long-term cardiovascular events and mortality in chronic hemodialysis patients | 2011 | Population data from before 2004 |
| 964 | Chiang, C. K., Ho, T. I., Hsu, S. P., Peng, Y. S., Pai, M. F., Yang, S. Y., Hung, K. Y. and Tsai, T. J. | Low-density lipoprotein cholesterol: association with mortality and hospitalization in hemodialysis patients | 2005 | Population data from before 2004 |
| 965 | Chien, S. C., Li, S. Y., Chen, Y. T., Tsai, L. W., Chen, T. J., Chen, T. W. and Lin, Y. C. | Folic acid supplementation in end-stage renal disease patients reduces total mortality rate | 2013 | Population data from before 2004 |
| 966 | Chow, K. M., Szeto, C. C., Law, M. C., Kwan, B. C., Leung, C. B. and Li, P. K. | Impact of early nephrology referral on mortality and hospitalization in peritoneal dialysis patients | 2008 | Population data from before 2004 |
| 967 | Choy, J. B., Armstrong, P. W., Ulan, R. A., Campbell, P. M., Gourishankar, S., Prosser, C. I. and Tymchak, W. J. | Do cardiac troponins provide prognostic insight in hemodialysis patients? | 2003 | Population data from before 2004 |
| 968 | Chung, S. H., Han, D. C., Noh, H., Jeon, J. S., Kwon, S. H., Lindholm, B. and Lee, H. B. | Risk factors for mortality in diabetic peritoneal dialysis patients | 2010 | Population data from before 2004 |
| 969 | Cice, G., Di Benedetto, A., D'Andrea, A., D'Isa, S., De Gregorio, P., Marcelli, D., Gatti, E. and Calabro, R. | Heart rate as independent prognostic factor for mortality in normotensive hemodialysed patients | 2008 | Population data from before 2004 |
| 970 | Coronel, F., Cigarran, S. and Herrero, J. A. | Morbidity and mortality in diabetic patients on peritoneal dialysis. Twenty-five years of experience at a single centre | 2010 | Population data from before 2004 |
| 971 | de Jager, D. J., Grootendorst, D. C., Jager, K. J., van Dijk, P. C., Tomas, L. M., Ansell, D., Collart, F., Finne, P., Heaf, J. G., De Meester, J., Wetzels, J. F., Rosendaal, F. R. and Dekker, F. W. | Cardiovascular and noncardiovascular mortality among patients starting dialysis | 2009 | Population data from before 2004 |
| 972 | De Lima, J. J., Sesso, R., Abensur, H., Lopes, H. F., Giorgi, M. C., Krieger, E. M. and Pileggi, F. | Predictors of mortality in long-term haemodialysis patients with a low prevalence of comorbid conditions | 1995 | Population data from before 2004 |
| 973 | Deicher, R., Ziai, F., Bieglmayer, C., Schillinger, M. and Horl, W. H. | Low total vitamin C plasma level is a risk factor for cardiovascular morbidity and mortality in hemodialysis patients | 2005 | Population data from before 2004 |
| 974 | den Elzen, W. P. J., van Manen, J., Boeschoten, E. W., Krediet, R. T. and Dekker, F. W. | The effect of single and repeatedly high concentrations of C-reactive protein on cardiovascular and non-cardiovascular mortality in patients starting with dialysis | 2006 | Population data from before 2004 |
| 975 | Diez, J. J., Bossola, M., Fernandez-Reyes, M. J., Di Stasio, E., Tazza, L., Luciani, G., Codoceo, R., Iglesias, P., Rodriguez, A., Gonzalez, E. and Selgas, R. | Relationship between leptin and all-cause and cardiovascular mortality in chronic hemodialysis patients | 2011 | Population data from before 2004 |
| 976 | Dimkovic, N., Schlieper, G., Jankovic, A., Djuric, Z., Ketteler, M., Damjanovic, T., Djuric, P., Marinkovic, J., Radojcic, Z., Markovic, N. and Floege, J. | Prognostic value of cardiovascular calcifications in hemodialysis patients: a longitudinal study | 2018 | Population data from before 2004 |
| 977 | Dogan, U., Ozdemir, K., Akilli, H., Aribas, A. and Turk, S. | Evaluation of echocardiographic indices for the prediction of major adverse events during long-term follow-up in chronic hemodialysis patients with normal left ventricular ejection fraction | 2012 | Population data from before 2004 |
| 978 | Dohi, T., Kasai, T., Miyauchi, K., Takasu, K., Kajimoto, K., Kubota, N., Amano, A. and Daida, H. | Prognostic impact of chronic kidney disease on 10-year clinical outcomes among patients with acute coronary syndrome | 2012 | Population data from before 2004 |
| 979 | Dong, J., Li, Y., Yang, Z. and Luo, J. | Low dietary sodium intake increases the death risk in peritoneal dialysis | 2010 | Population data from before 2004 |
| 980 | Dong, J., Li, Y. J., Yang, Z. K. and Xu, R. | Prognostic value of serum von Willebrand factor, but not soluble ICAM and VCAM, for mortality and cardiovascular events is independent of residual renal function in peritoneal dialysis patients | 2014 | Population data from before 2004 |
| 981 | Drechsler, C., Verduijn, M., Pilz, S., Dekker, F. W., Krediet, R. T., Ritz, E., Wanner, C., Boeschoten, E. W. and Brandenburg, V. | Vitamin D status and clinical outcomes in incident dialysis patients: results from the NECOSAD study | 2011 | Population data from before 2004 |
| 982 | Dumaine, R. L., Montalescot, G., Steg, P. G., Ohman, E. M., Eagle, K. and Bhatt, D. L. | Renal function, atherothrombosis extent, and outcomes in high-risk patients | 2009 | Population data from before 2004 |
| 983 | Duong, U., Kalantar-Zadeh, K., Molnar, M. Z., Zaritsky, J. J., Teitelbaum, I., Kovesdy, C. P. and Mehrotra, R. | Mortality associated with dose response of erythropoiesis-stimulating agents in hemodialysis versus peritoneal dialysis patients | 2012 | Population data from before 2004 |
| 984 | Duong, U., Mehrotra, R., Molnar, M. Z., Noori, N., Kovesdy, C. P., Nissenson, A. R. and Kalantar-Zadeh, K. | Glycemic control and survival in peritoneal dialysis patients with diabetes mellitus | 2011 | Population data from before 2004 |
| 985 | Eddington, H., Hoefield, R., Sinha, S., Chrysochou, C., Lane, B., Foley, R. N., Hegarty, J., New, J., O'Donoghue, D. J., Middleton, R. J. and Kalra, P. A. | Serum phosphate and mortality in patients with chronic kidney disease | 2010 | Population data from before 2004 |
| 986 | Efrati, S., Zaidenstein, R., Dishy, V., Beberashvili, I., Sharist, M., Averbukh, Z., Golik, A. and Weissgarten, J. | ACE inhibitors and survival of hemodialysis patients | 2002 | Population data from before 2004 |
| 987 | Ekart, R., Hojs, R., Hojs-Fabjan, T. and Balon, B. P. | Predictive value of carotid intima media thickness in hemodialysis patients | 2005 | Population data from before 2004 |
| 988 | Ekart, R., Kanic, V., Pecovnik Balon, B., Bevc, S. and Hojs, R. | Prognostic value of 48-hour ambulatory blood pressure measurement and cardiovascular mortality in hemodialysis patients | 2012 | Population data from before 2004 |
| 989 | Ekundayo, O. J., Muchimba, M., Aban, I. B., Ritchie, C., Campbell, R. C. and Ahmed, A. | Multimorbidity due to diabetes mellitus and chronic kidney disease and outcomes in chronic heart failure | 2009 | Population data from before 2004 |
| 990 | Espe, K. M., Raila, J., Henze, A., Krane, V., Schweigert, F. J., Hocher, B., Wanner, C., Drechsler, C., German, D. and Dialysis Study, I. | Impact of vitamin A on clinical outcomes in haemodialysis patients | 2011 | Population data from before 2004 |
| 991 | Fabbian, F., Pala, M., De Giorgi, A., Manfredini, F., Mallozzi Menegatti, A., Salmi, R., Portaluppi, F., Gallerani, M. and Manfredini, R. | In-hospital mortality in patients with renal dysfunction admitted for myocardial infarction: the Emilia-Romagna region of Italy database of hospital admissions | 2013 | Population data from before 2004 |
| 992 | Fauchier, L., Villejoubert, O., Clementy, N., Bernard, A., Pierre, B., Angoulvant, D., Ivanes, F., Babuty, D. and Lip, G. Y. H. | Causes of Death and Influencing Factors in Patients with Atrial Fibrillation | 2016 | Population data from before 2004 |
| 993 | Fried, L. F., Shlipak, M. G., Crump, C., Bleyer, A. J., Gottdiener, J. S., Kronmal, R. A., Kuller, L. H. and Newman, A. B. | Renal insufficiency as a predictor of cardiovascular outcomes and mortality in elderly individuals | 2003 | Population data from before 2004 |
| 994 | Fujishima, Y., Ohsawa, M., Itai, K., Kato, K., Tanno, K., Turin, T. C., Onoda, T., Endo, S., Okayama, A. and Fujioka, T. | Serum selenium levels are inversely associated with death risk among hemodialysis patients | 2011 | Population data from before 2004 |
| 995 | Fukuta, H., Hayano, J., Ishihara, S., Sakata, S., Mukai, S., Ohte, N., Ojika, K., Yagi, K., Matsumoto, H., Sohmiya, S. and Kimura, G. | Prognostic value of heart rate variability in patients with end-stage renal disease on chronic haemodialysis | 2003 | Population data from before 2004 |
| 996 | Fukuta, H., Hayano, J., Ishihara, S., Sakata, S., Ohte, N., Takahashi, H., Yokoya, M., Toriyama, T., Kawahara, H., Yajima, K., Kobayashi, K. and Kimura, G. | Prognostic value of nonlinear heart rate dynamics in hemodialysis patients with coronary artery disease | 2003 | Population data from before 2004 |
| 997 | Fung, F., Sherrard, D. J., Gillen, D. L., Wong, C., Kestenbaum, B., Seliger, S., Ball, A. and Stehman-Breen, C. | Increased risk for cardiovascular mortality among malnourished end-stage renal disease patients | 2002 | Population data from before 2004 |
| 998 | Gabbai, F. B., Rahman, M., Hu, B., Appel, L. J., Charleston, J., Contreras, G., Faulkner, M. L., Hiremath, L., Jamerson, K. A., Lea, J. P., Lipkowitz, M. S., Pogue, V. A., Rostand, S. G., Smogorzewski, M. J., Wright, J. T., Greene, T., Gassman, J., Wang, X. and Phillips, R. A. | Relationship between ambulatory BP and clinical outcomes in patients with hypertensive CKD | 2012 | Population data from before 2004 |
| 999 | Gadallah, M. F., el-Shahawy, M., Andrews, G., Ibrahim, M., Ramdeen, G., Hanna, D., Gorospe, W., Morkos, A., Abbassian, M. and Moles, K. | Factors modulating cytosolic calcium. Role in lipid metabolism and cardiovascular morbidity and mortality in peritoneal dialysis patients | 2001 | Population data from before 2004 |
| 1000 | Garg, A. X., Clark, W. F., Haynes, R. B. and House, A. A. | Moderate renal insufficiency and the risk of cardiovascular mortality: Results from the NHANES I | 2002 | Population data from before 2004 |
| 1001 | Gayoso-Diz, P., Otero-Gonzalez, A., Rodriguez-Alvarez, M. X., Garcia, F., Gonzalez-Quintela, A. and Martin de Francisco, A. L. | Strategy to estimate risk progression of chronic kidney disease, cardiovascular risk, and referral to nephrology: the EPIRCE Study | 2013 | Population data from before 2004 |
| 1002 | Glynn, L. G., Reddan, D., Newell, J., Hinde, J., Buckley, B. and Murphy, A. W. | Chronic kidney disease and mortality and morbidity among patients with established cardiovascular disease: a West of Ireland community-based cohort study | 2007 | Population data from before 2004 |
| 1003 | Go, A. S., Yang, J., Ackerson, L. M., Lepper, K., Robbins, S., Massie, B. M. and Shlipak, M. G. | Hemoglobin level, chronic kidney disease, and the risks of death and hospitalization in adults with chronic heart failure: the Anemia in Chronic Heart Failure: Outcomes and Resource Utilization (ANCHOR) Study | 2006 | Population data from before 2004 |
| 1004 | Goldfarb-Rumyantzev, A. S., Baird, B. C., Leypoldt, J. K. and Cheung, A. K. | The association between BP and mortality in patients on chronic peritoneal dialysis | 2005 | Population data from before 2004 |
| 1005 | Goldfarb-Rumyantzev, A. S., Habib, A. N., Baird, B. C., Barenbaum, L. L. and Cheung, A. K. | The association of lipid-modifying medications with mortality in patients on long-term peritoneal dialysis | 2007 | Population data from before 2004 |
| 1006 | Gouya, G., Sturm, G., Lamina, C., Zitt, E., Freistatter, O., Struck, J., Wolzt, M., Knoll, F., Lins, F., Lhotta, K., Neyer, U. and Kronenberg, F. | The association of mid-regional pro-adrenomedullin and mid-regional pro-atrial natriuretic peptide with mortality in an incident dialysis cohort | 2011 | Population data from before 2004 |
| 1007 | Guerin, A. P., Blacher, J., Pannier, B., Marchais, S. J., Safar, M. E. and London, G. M. | Impact of aortic stiffness attenuation on survival of patients in end-stage renal failure | 2001 | Population data from before 2004 |
| 1008 | Haire, H. M., Sherrard, D. J. and Scardapane, D. | Smoking, hypertension, and mortality in a maintenance dialysis population | 1978 | Population data from before 2004 |
| 1009 | Hakeem, A., Bhatti, S., Dillie, K. S., Cook, J. R., Samad, Z., Roth-Cline, M. D. and Chang, S. M. | Predictive value of myocardial perfusion single-photon emission computed tomography and the impact of renal function on cardiac death | 2008 | Population data from before 2004 |
| 1010 | Held, P. J., Levin, N. W., Bovbjerg, R. R., Pauly, M. V. and Diamond, L. H. | Mortality and duration of hemodialysis treatment | 1991 | Population data from before 2004 |
| 1011 | Henry, R. M., Kostense, P. J., Bos, G., Dekker, J. M., Nijpels, G., Heine, R. J., Bouter, L. M. and Stehouwer, C. D. | Mild renal insufficiency is associated with increased cardiovascular mortality: The Hoorn Study | 2002 | Population data from before 2004 |
| 1012 | Hocher, B., Kalk, P., Godes, M., Liefeldt, L., Ziebig, R., Stasch, J. P., Quaschning, T. and Pfab, T. | Gender-dependent impact of risk factors for cardiovascular and non-cardiovascular mortality in end-stage renal disease patients on haemodialysis | 2008 | Population data from before 2004 |
| 1013 | Hocher, B., Liefeldt, L., Quaschning, T., Kalk, P., Ziebig, R., Godes, M., Relle, K., Asmus, G. and Stasch, J. P. | Soluble CD154 is a unique predictor of nonfatal and fatal atherothrombotic events in patients who have end-stage renal disease and are on hemodialysis | 2007 | Population data from before 2004 |
| 1014 | Holzmann, M. J., Carlsson, A. C., Hammar, N., Ivert, T., Walldius, G., Jungner, I., Wandell, P. and Arnlov, J. | Chronic kidney disease and 10-year risk of cardiovascular death | 2016 | Population data from before 2004 |
| 1015 | Holzmann, M. J., Ivert, T., Jungner, I., Nordqvist, T., Walldius, G., Ostergren, J. and Hammar, N. | Renal function assessed by two different formulas and incidence of myocardial infarction and death in middle-aged men and women | 2010 | Population data from before 2004 |
| 1016 | Holzmann, M. J., Janszky, I., Al-Khalili, F. and Schenck-Gustafsson, K. | Renal dysfunction as a predictor of long-term mortality in middle-aged women following an acute coronary syndrome | 2010 | Population data from before 2004 |
| 1017 | Hoy, W. E., Mathews, J. D., McCredie, D. A., Pugsley, D. J., Hayhurst, B. G., Rees, M., Kile, E., Walker, K. A. and Wang, Z. | The multidimensional nature of renal disease: rates and associations of albuminuria in an Australian Aboriginal community | 1998 | Population data from before 2004 |
| 1018 | Hoy, W. E., Wang, Z., VanBuynder, P., Baker, P. R. A., McDonald, S. M. and Mathews, J. D. | The natural history of renal disease in Australian Aborigines. Part 2. Albuminuria predicts natural death and renal failure | 2001 | Population data from before 2004 |
| 1019 | Hsieh, Y. P., Chang, C. C., Yang, Y., Wen, Y. K., Chiu, P. F. and Lin, C. C. | The role of uric acid in chronic kidney disease patients | 2017 | Population data from before 2004 |
| 1020 | Huang, J. T., Cheng, H. M., Yu, W. C., Lin, Y. P., Sung, S. H., Wang, J. J., Wu, C. L. and Chen, C. H. | Value of Excess Pressure Integral for Predicting 15-Year All-Cause and Cardiovascular Mortalities in End-Stage Renal Disease Patients | 2017 | Population data from before 2004 |
| 1021 | Iff, S., Wong, G., Webster, A. C., Flood, V., Wang, J. J., Mitchell, P. and Craig, J. C. | Relative energy balance, CKD, and risk of cardiovascular and all-cause mortality | 2014 | Population data from before 2004 |
| 1022 | Iliou, M. C., Fumeron, C., Benoit, M. O., Tuppin, P., Calonge, V. M., Moatti, N., Buisson, C. and Jacquot, C. | Prognostic value of cardiac markers in ESRD: Chronic Hemodialysis and New Cardiac Markers Evaluation (CHANCE) study | 2003 | Population data from before 2004 |
| 1023 | Inaguma, D., Nagaya, H., Hara, K., Tatematsu, M., Shinjo, H., Suzuki, S., Mishima, T. and Kurata, K. | Relationship between serum 1,25-dihydroxyvitamin D and mortality in patients with pre-dialysis chronic kidney disease | 2008 | Population data from before 2004 |
| 1024 | Ishii, J., Nomura, M., Okuma, T., Minagawa, T., Naruse, H., Mori, Y., Ishikawa, T., Kurokawa, H., Hirano, T., Kondo, T., Nagamura, Y., Ezaki, K. and Hishida, H. | Risk stratification using serum concentrations of cardiac troponin T in patients with end-stage renal disease on chronic maintenance dialysis | 2001 | Population data from before 2004 |
| 1025 | Ishimitsu, T., Nakano, N., Sudo, Y., Akashiba, A., Takahashi, T., Ohta, S., Minami, J. and Matsuoka, H. | Predictive significance of blood pressure values for the incidence of cardiovascular events in chronic hemodialysis patients | 2008 | Population data from before 2004 |
| 1026 | Joki, N., Hase, H., Saijyo, T., Tanaka, Y., Takahashi, Y., Ishikawa, H., Nakamura, R., Fukazawa, M., Inishi, Y., Nakamura, M. and Imamura, Y. | Combined assessment of cardiac systolic dysfunction and coronary atherosclerosis used to predict future cardiac deaths after starting hemodialysis | 2003 | Population data from before 2004 |
| 1027 | Joki, N., Hase, H., Takahashi, Y., Ishikawa, H., Nakamura, R., Imamura, Y., Tanaka, Y., Saijyo, T., Fukazawa, M., Inishi, Y., Nakamura, M. and Yamaguchi, T. | Angiographical severity of coronary atherosclerosis predicts death in the first year of hemodialysis | 2003 | Population data from before 2004 |
| 1028 | Jorsal, A., Tarnow, L., Flyvbjerg, A., Parving, H. H., Rossing, P. and Rasmussen, L. M. | Plasma osteoprotegerin levels predict cardiovascular and all-cause mortality and deterioration of kidney function in type 1 diabetic patients with nephropathy | 2008 | Population data from before 2004 |
| 1029 | Kagiyama, S., Matsumura, K., Ansai, T., Soh, I., Takata, Y., Awano, S., Sonoki, K., Yoshida, A., Takehara, T. and Iida, M. | Chronic kidney disease increases cardiovascular mortality in 80-year-old subjects in Japan | 2008 | Population data from before 2004 |
| 1030 | Kalantar-Zadeh, K., Kilpatrick, R. D., McAllister, C. J., Greenland, S. and Kopple, J. D. | Reverse epidemiology of hypertension and cardiovascular death in the hemodialysis population: The 58th annual fall conference and scientific sessions | 2005 | Population data from before 2004 |
| 1031 | Kalantar-Zadeh, K., Regidor, D. L., McAllister, C. J., Michael, B. and Warnock, D. G. | Time-dependent associations between iron and mortality in hemodialysis patients | 2005 | Population data from before 2004 |
| 1032 | Kalousová, M., Benáková, H., Kuběna, A. A., Dusilová-Sulková, S., Tesař, V. and Zima, T. | Pregnancy-associated plasma protein A as an independent mortality predictor in long-term hemodialysis patients | 2012 | Population data from before 2004 |
| 1033 | Kalousová, M., Kuběna, A. A., Koštířová, M., Vinglerová, M., Ing, O. M., Dusilová-Sulková, S., Tesař, V. and Zima, T. | Lower retinol levels as an independent predictor of mortality in long-term hemodialysis patients: A prospective observational cohort study | 2010 | Population data from before 2004 |
| 1034 | Kanaan, N., Goffin, E., Maisin, D., Struyven, J. and Jadoul, M. | CRP measurement: does the assay matter in hemodialysis patients? | 2008 | Population data from before 2004 |
| 1035 | Kang, E. W., Pike, F., Ramer, S., Abdel-Kader, K., Myaskovsky, L., Dew, M. A. and Unruh, M. | The association of mental health over time with cardiac outcomes in HEMO study patients | 2012 | Population data from before 2004 |
| 1036 | Kessler, M., Zannad, F., Lehert, P., Grunfeld, J. P., Thuilliez, C., Leizorovicz, A. and Lechat, P. | Predictors of cardiovascular events in patients with end-stage renal disease: an analysis from the Fosinopril in Dialysis study | 2007 | Population data from before 2004 |
| 1037 | Kestenbaum, B., Gillen, D. L., Sherrard, D. J., Seliger, S., Ball, A. and Stehman-Breen, C. | Calcium channel blocker use and mortality among patients with end-stage renal disease | 2002 | Population data from before 2004 |
| 1038 | Ketteler, M., Bongartz, P., Westenfeld, R., Wildberger, J. E., Mahnken, A. H., Bohm, R., Metzger, T., Wanner, C., Jahnen-Dechent, W. and Floege, J. | Association of low fetuin-A (AHSG) concentrations in serum with cardiovascular mortality in patients on dialysis: a cross-sectional study | 2003 | Population data from before 2004 |
| 1039 | Kircelli, F., Asci, G., Yilmaz, M., Sevinc Ok, E., Demirci, M. S., Toz, H., Akcicek, F., Ok, E. and Ozkahya, M. | The impact of strict volume control strategy on patient survival and technique failure in peritoneal dialysis patients | 2011 | Population data from before 2004 |
| 1040 | Kitterer, D., Segerer, S., Braun, N., Alscher, M. D. and Latus, J. | Gender-Specific Differences in Peritoneal Dialysis | 2017 | Population data from before 2004 |
| 1041 | Koc, Y., Unsal, A., Ahbap, E., Sakaci, T. and Yilmaz, M. | Clinical outcome of diabetic peritoneal dialysis patients and evaluation of factors affecting mortality: a single centre's experience from Turkey | 2011 | Population data from before 2004 |
| 1042 | Koch, M., Thomas, B., Tschope, W. and Ritz, E. | Survival and predictors of death in dialysed diabetic patients | 1993 | Population data from before 2004 |
| 1043 | Koda, Y., Nishi, S. I., Suzuki, M. and Hirasawa, Y. | Lipoprotein(a) is a predictor for cardiovascular mortality of hemodialysis patients | 1999 | Population data from before 2004 |
| 1044 | Koyama, H., Shoji, T., Fukumoto, S., Shinohara, K., Shoji, T., Emoto, M., Mori, K., Tahara, H., Ishimura, E., Kakiya, R., Tabata, T., Yamamoto, H. and Nishizawa, Y. | Low circulating endogenous secretory receptor for AGEs predicts cardiovascular mortality in patients with end-stage renal disease | 2007 | Population data from before 2004 |
| 1045 | Kumakura, H., Kanai, H., Aizaki, M., Mitsui, K., Araki, Y., Kasama, S., Iwasaki, T. and Ichikawa, S. | The influence of the obesity paradox and chronic kidney disease on long-term survival in a Japanese cohort with peripheral arterial disease | 2010 | Population data from before 2004 |
| 1046 | Kuo, C. F., See, L. C., Yu, K. H., Chou, I. J., Chiou, M. J. and Luo, S. F. | Significance of serum uric acid levels on the risk of all-cause and cardiovascular mortality | 2013 | Population data from before 2004 |
| 1047 | Kurnatowska, I., Grzelak, P., Kaczmarska, M., Stefańczyk, L. and Nowicki, M. | Relations between serum sex hormone levels and biomarkers of atherosclerosis and mineral disturbances in postmenopausal chronic haemodialysis women | 2010 | Population data from before 2004 |
| 1048 | Lajer, M., Jorsal, A., Tarnow, L., Parving, H. H. and Rossing, P. | Plasma growth differentiation factor-15 independently predicts all-cause and cardiovascular mortality as well as deterioration of kidney function in type 1 diabetic patients with nephropathy | 2010 | Population data from before 2004 |
| 1049 | Langston, R. D., Presley, R., Flanders, W. D. and McClellan, W. M. | Renal insufficiency and anemia are independent risk factors for death among patients with acute myocardial infarction | 2003 | Population data from before 2004 |
| 1050 | Latif, W., Karaboyas, A., Tong, L., Winchester, J. F., Arrington, C. J., Pisoni, R. L., Marshall, M. R., Kleophas, W., Levin, N. W., Sen, A. and et al. | Uric acid levels and all-cause and cardiovascular mortality in the hemodialysis population | 2011 | Population data from before 2004 |
| 1051 | Lee, C. C., Sun, C. Y. and Wu, M. S. | Long-term modality-related mortality analysis in incident dialysis patients | 2009 | Population data from before 2004 |
| 1052 | Lee, C. T., Huang, C. C., Hsu, C. Y., Chiou, T. T. Y., Ng, H. Y., Wu, C. H., Kuo, W. H. and Lee, Y. T. | Calcification of the aortic arch predicts cardiovascular and all-cause mortality in chronic hemodialysis patients | 2014 | Population data from before 2004 |
| 1053 | Leeder, S. R., Mitchell, P., Liew, G., Rochtchina, E., Smith, W. and Wang, J. J. | Low hemoglobin, chronic kidney disease, and risk for coronary heart disease-related death: the Blue Mountains Eye Study | 2006 | Population data from before 2004 |
| 1054 | Leinig, C. E., Moraes, T., Ribeiro, S., Riella, M. C., Olandoski, M., Martins, C. and Pecoits-Filho, R. | Predictive value of malnutrition markers for mortality in peritoneal dialysis patients | 2011 | Population data from before 2004 |
| 1055 | Leung, J., Larive, B., Dwyer, J., Hibberd, P., Jacques, P. and Rand, W. | Folic acid supplementation and cardiac and stroke mortality among hemodialysis patients | 2010 | Population data from before 2004 |
| 1056 | Li, S. and Collins, A. J. | Association of hematocrit value with cardiovascular morbidity and mortality in incident hemodialysis patients | 2004 | Population data from before 2004 |
| 1057 | Li, Y. H., Lin, G. M., Lin, C. L., Wang, J. H. and Han, C. L. | Relation of estimated glomerular filtration rate and body mass index to mortality in non-dialysis patients with coronary artery disease: a report from the ET-CHD registry, 1997-2003 | 2013 | Population data from before 2004 |
| 1058 | Liao, C. T., Kao, T. W., Chou, Y. H., Wu, M. S., Chen, Y. M., Chuang, H. F., Hung, K. Y., Chu, T. S., Wu, K. D. and Tsai, T. J. | Associations of metabolic syndrome and its components with cardiovascular outcomes among non-diabetic patients undergoing maintenance peritoneal dialysis | 2011 | Population data from before 2004 |
| 1059 | Liu, M., Takahashi, H., Morita, Y., Maruyama, S., Mizuno, M., Yuzawa, Y., Watanabe, M., Toriyama, T., Kawahara, H. and Matsuo, S. | Non-dipping is a potent predictor of cardiovascular mortality and is associated with autonomic dysfunction in haemodialysis patients | 2003 | Population data from before 2004 |
| 1060 | Lopes, A. A., Bragg-Gresham, J. L., Ramirez, S. P., Andreucci, V. E., Akiba, T., Saito, A., Jacobson, S. H., Robinson, B. M., Port, F. K., Mason, N. A. and Young, E. W. | Prescription of antihypertensive agents to haemodialysis patients: time trends and associations with patient characteristics, country and survival in the DOPPS | 2009 | Population data from before 2004 |
| 1061 | Ma, J. Z., Ebben, J., Xia, H. and Collins, A. J. | Hematocrit level and associated mortality in hemodialysis patients | 1999 | Population data from before 2004 |
| 1062 | MacHowska, A., Sun, J., Qureshi, A. R., Isoyama, N., Leurs, P., Anderstam, B., Heimburger, O., Barany, P., Stenvinkel, P. and Lindholm, B. | Plasma pentosidine and its association with mortality in patients with chronic kidney disease | 2016 | Population data from before 2004 |
| 1063 | Madero, M., Sarnak, M. J., Wang, X., Greene, T., Beck, G. J., Kusek, J. W., Collins, A. J., Levey, A. S. and Menon, V. | Uric acid and long-term outcomes in CKD | 2009 | Population data from before 2004 |
| 1064 | Mallamaci, F., Zoccali, C., Parlongo, S., Tripepi, G., Benedetto, F. A., Cutrupi, S., Bonanno, G., Fatuzzo, P., Rapisarda, F., Seminara, G., Stancanelli, B., Bellanuova, I., Cataliotti, A. and Malatino, L. S. | Troponin is related to left ventricular mass and predicts all-cause and cardiovascular mortality in hemodialysis patients | 2002 | Population data from before 2004 |
| 1065 | Marks, A., Macleod, C., McAteer, A., Murchie, P., Fluck, N., Smith, W. C., Prescott, G. J., Clark, L. E., Ali, T. and Black, C. | Chronic kidney disease, a useful trigger for proactive primary care? Mortality results from a large U.K. cohort | 2013 | Population data from before 2004 |
| 1066 | Matsubara, K., Stenvinkel, P., Qureshi, A. R., Carrero, J. J., Axelsson, J., Heimburger, O., Barany, P., Alvestrand, A., Lindholm, B. and Suliman, M. E. | Inflammation modifies the association of osteoprotegerin with mortality in chronic kidney disease | 2009 | Population data from before 2004 |
| 1067 | Meerwaldt, R., Hartog, J. W., Graaff, R., Huisman, R. J., Links, T. P., den Hollander, N. C., Thorpe, S. R., Baynes, J. W., Navis, G., Gans, R. O. and Smit, A. J. | Skin autofluorescence, a measure of cumulative metabolic stress and advanced glycation end products, predicts mortality in hemodialysis patients | 2005 | Population data from before 2004 |
| 1068 | Meisinger, C., Doring, A. and Lowel, H. | Chronic kidney disease and risk of incident myocardial infarction and all-cause and cardiovascular disease mortality in middle-aged men and women from the general population | 2006 | Population data from before 2004 |
| 1069 | Melamed, M. L., Plantinga, L., Shafi, T., Parekh, R., Meyer, T. W., Hostetter, T. H., Coresh, J. and Powe, N. R. | Retained organic solutes, patient characteristics and all-cause and cardiovascular mortality in hemodialysis: results from the retained organic solutes and clinical outcomes (ROSCO) investigators | 2013 | Population data from before 2004 |
| 1070 | Menon, V., Greene, T., Pereira, A. A., Wang, X., Beck, G. J., Kusek, J. W., Collins, A. J., Levey, A. S. and Sarnak, M. J. | Relationship of phosphorus and calcium-phosphorus product with mortality in CKD | 2005 | Population data from before 2004 |
| 1071 | Menon, V., Li, L., Wang, X., Greene, T., Balakrishnan, V., Madero, M., Pereira, A. A., Beck, G. J., Kusek, J. W., Collins, A. J. and et al. | Adiponectin and mortality in patients with chronic kidney disease | 2006 | Population data from before 2004 |
| 1072 | Millet, C., Bosson, J. L., Pernod, G., Wauters, J. P., Couturier, P., Quesada, J. L. and Zaoui, P. | Cardiovascular mortality and C-reactive protein in elderly patients beginning dialysis: reverse epidemiology? | 2011 | Population data from before 2004 |
| 1073 | Mocks, J. | Cardiovascular mortality in haemodialysis patients treated with epoetin beta - a retrospective study | 2000 | Population data from before 2004 |
| 1074 | Mok, Y., Matsushita, K., Sang, Y., Ballew, S. H., Grams, M., Shin, S. Y., Jee, S. H. and Coresh, J. | Association of kidney disease measures with cause-specific mortality: The Korean Heart Study | 2016 | Population data from before 2004 |
| 1075 | Morduchowicz, G., Winkler, J., Derazne, E., Van Dyk, D. J., Wittenberg, C., Zabludowski, J. R., Shohat, J., Rosenfeld, J. B. and Boner, G. | Causes of death in patients with end-stage renal disease treated by dialysis in a center in Israel | 1992 | Population data from before 2004 |
| 1076 | Morena, M., Terrier, N., Jaussent, I., Leray-Moragues, H., Chalabi, L., Rivory, J. P., Maurice, F., Delcourt, C., Cristol, J. P., Canaud, B. and Dupuy, A. M. | Plasma osteoprotegerin is associated with mortality in hemodialysis patients | 2006 | Population data from before 2004 |
| 1077 | Mostovaya, I. M., Bots, M. L., van den Dorpel, M. A., Goldschmeding, R., den Hoedt, C. H., Kamp, O., Levesque, R., Mazairac, A. H., Penne, E. L., Swinkels, D. W. and et al. | Left ventricular mass in dialysis patients, determinants and relation with outcome. Results from the COnvective TRansport STudy (CONTRAST) | 2014 | Population data from before 2004 |
| 1078 | Nabais, S., Rocha, S., Joao, C., Marques, J., Torres, M., Magalhaes, S., Pereira, M. A. and Correia, A. | Prognostic impact of moderate renal dysfunction in acute coronary syndromes | 2008 | Population data from before 2004 |
| 1079 | Nagai, K., Sairenchi, T., Irie, F., Watanabe, H., Ota, H. and Yamagata, K. | Relationship between Estimated Glomerular Filtration Rate and Cardiovascular Mortality in a Japanese Cohort with Long-Term Follow-Up | 2016 | Population data from before 2004 |
| 1080 | Naganuma, T., Sugimura, K., Wada, S., Yasumoto, R., Sugimura, T., Masuda, C., Uchida, J. and Nakatani, T. | The prognostic role of brain natriuretic peptides in hemodialysis patients | 2002 | Population data from before 2004 |
| 1081 | Nakagawa, K., Hirai, T., Takashima, S., Fukuda, N., Ohara, K., Sasahara, E., Taguchi, Y., Dougu, N., Nozawa, T., Tanaka, K. and Inoue, H. | Chronic kidney disease and CHADS(2) score independently predict cardiovascular events and mortality in patients with nonvalvular atrial fibrillation | 2011 | Population data from before 2004 |
| 1082 | Nakamura, K., Nakagawa, H., Murakami, Y., Kitamura, A., Kiyama, M., Sakata, K., Tsuji, I., Miura, K., Ueshima, H. and Okamura, T. | Smoking increases the risk of all-cause and cardiovascular mortality in patients with chronic kidney disease | 2015 | Population data from before 2004 |
| 1083 | Nakamura, S., Sasaki, O., Nakahama, H., Inenaga, T. and Kawano, Y. | Clinical characteristics and survival in end-stage renal disease patients with arteriosclerosis obliterans | 2002 | Population data from before 2004 |
| 1084 | Nakashima, A., Carrero, J. J., Qureshi, A. R., Hirai, T., Takasugi, N., Ueno, T., Taniguchi, Y., Lindholm, B. and Yorioka, N. | Plasma osteoprotegerin, arterial stiffness, and mortality in normoalbuminemic Japanese hemodialysis patients | 2011 | Population data from before 2004 |
| 1085 | Nishimura, M., Tsukamoto, K., Hasebe, N., Tamaki, N., Kikuchi, K. and Ono, T. | Prediction of cardiac death in hemodialysis patients by myocardial fatty acid imaging | 2008 | Population data from before 2004 |
| 1086 | Nishizawa, Y., Shoji, T., Kakiya, R., Tsujimoto, Y., Tabata, T., Ishimura, E., Nakatani, T., Miki, T. and Inaba, M. | Non-high-density lipoprotein cholesterol (non-HDL-C) as a predictor of cardiovascular mortality in patients with end-stage renal disease | 2003 | Population data from before 2004 |
| 1087 | Nishizawa, Y., Shoji, T., Maekawa, K., Nagasue, K., Okuno, S., Kim, M., Emoto, M., Ishimura, E., Nakatani, T., Miki, T. and Inaba, M. | Intima-media thickness of carotid artery predicts cardiovascular mortality in hemodialysis patients | 2003 | Population data from before 2004 |
| 1088 | Nitsch, D., Grams, M., Sang, Y., Black, C., Cirillo, M., Djurdjev, O., Iseki, K., Jassal, S. K., Kimm, H., Kronenberg, F., Oien, C. M., Levey, A. S., Levin, A., Woodward, M. and Hemmelgarn, B. R. | Associations of estimated glomerular filtration rate and albuminuria with mortality and renal failure by sex: a meta-analysis | 2013 | Population data from before 2004 |
| 1089 | Nitsch, D., Lawlor, D. A., Patel, R., Carson, C. and Ebrahim, S. | The association of renal impairment with all-cause and cardiovascular disease mortality | 2010 | Population data from before 2004 |
| 1090 | Noordzij, M., Cranenburg, E. M., Engelsman, L. F., Hermans, M. M., Boeschoten, E. W., Brandenburg, V. M., Bos, W. J., Kooman, J. P., Dekker, F. W., Ketteler, M., Schurgers, L. J., Krediet, R. T. and Korevaar, J. C. | Progression of aortic calcification is associated with disorders of mineral metabolism and mortality in chronic dialysis patients | 2011 | Population data from before 2004 |
| 1091 | Noordzij, M., Korevaar, J. C., Bos, W. J., Boeschoten, E. W., Dekker, F. W., Bossuyt, P. M. and Krediet, R. T. | Mineral metabolism and cardiovascular morbidity and mortality risk: peritoneal dialysis patients compared with haemodialysis patients | 2006 | Population data from before 2004 |
| 1092 | Noori, N., Caulfield, M. P., Salameh, W. A., Reitz, R. E., Nicholas, S. B., Molnar, M. Z., Nissenson, A. R., Kovesdy, C. P. and Kalantar-Zadeh, K. | Novel lipoprotein subfraction and size measurements in prediction of mortality in maintenance hemodialysis patients | 2011 | Population data from before 2004 |
| 1093 | Obi, Y., Kalantar-Zadeh, K., Streja, E., Rhee, C. M., Reddy, U. G., Soohoo, M., Wang, Y., Ravel, V., You, A. S., Jing, J., Sim, J. J., Nguyen, D. V., Gillen, D. L., Saran, R., Robinson, B. and Kovesdy, C. P. | Seasonal variations in transition, mortality and kidney transplantation among patients with end-stage renal disease in the USA | 2017 | Population data from before 2004 |
| 1094 | Ocak, G., Halbesma, N., le Cessie, S., Hoogeveen, E. K., van Dijk, S., Kooman, J., Dekker, F. W., Krediet, R. T., Boeschoten, E. W. and Verduijn, M. | Haemodialysis catheters increase mortality as compared to arteriovenous accesses especially in elderly patients | 2011 | Population data from before 2004 |
| 1095 | Ocak, G., Van Stralen, K. J., Rosendaal, F. R., Verduijn, M., Ravani, P., Palsson, R., Leivestad, T., Hoitsma, A. J., Ferrer-Alamar, M., Finne, P., De Meester, J., Wanner, C., Dekker, F. W. and Jager, K. J. | Mortality due to pulmonary embolism, myocardial infarction, and stroke among incident dialysis patients | 2012 | Population data from before 2004 |
| 1096 | Odden, M. C., Amadu, A. R., Smit, E., Lo, L. and Peralta, C. A. | Uric acid levels, kidney function, and cardiovascular mortality in US adults: National Health and Nutrition Examination Survey (NHANES) 1988-1994 and 1999-2002 | 2014 | Population data from before 2004 |
| 1097 | Ohya, M., Otani, H., Kimura, K., Saika, Y., Fujii, R., Yukawa, S. and Shigematsu, T. | Vascular calcification estimated by aortic calcification area index is a significant predictive parameter of cardiovascular mortality in hemodialysis patients | 2011 | Population data from before 2004 |
| 1098 | Okuno, S., Ishimura, E., Kitatani, K., Fujino, Y., Kohno, K., Maeno, Y., Maekawa, K., Yamakawa, T., Imanishi, Y., Inaba, M. and Nishizawa, Y. | Presence of Abdominal Aortic Calcification Is Significantly Associated With All-Cause and Cardiovascular Mortality in Maintenance Hemodialysis Patients | 2007 | Population data from before 2004 |
| 1099 | Okuno, S., Ishimura, E., Kohno, K., Fujino-Katoh, Y., Maeno, Y., Yamakawa, T., Inaba, M. and Nishizawa, Y. | Serum beta2-microglobulin level is a significant predictor of mortality in maintenance haemodialysis patients | 2009 | Population data from before 2004 |
| 1100 | Okuyama, C., Nakajima, K., Hatta, T., Nishimura, S., Kusuoka, H., Yamashina, A. and Nishimura, T. | Incremental prognostic value of myocardial perfusion single photon emission computed tomography for patients with diabetes and chronic kidney disease | 2011 | Population data from before 2004 |
| 1101 | Paniagua, R., Amato, D., Mujais, S., Vonesh, E., Ramos, A., Correa-Rotter, R. and Horl, W. H. | Predictive value of brain natriuretic peptides in patients on peritoneal dialysis: results from the ADEMEX trial | 2008 | Population data from before 2004 |
| 1102 | Panichi, V., Maggiore, U., Taccola, D., Migliori, M., Rizza, G. M., Consani, C., Bertini, A., Sposini, S., Perez-Garcia, R., Rindi, P., Palla, R. and Tetta, C. | Interleukin-6 is a stronger predictor of total and cardiovascular mortality than C-reactive protein in haemodialysis patients | 2004 | Population data from before 2004 |
| 1103 | Panichi, V., Taccola, D., Migliori, M., Consani, C., Giovannini, L. and Tetta, C. | The role of chronic inflammation in cardiovascular mortality of uremic patients | 2003 | Population data from before 2004 |
| 1104 | Panichi, V., Taccola, D., Rizza, G. M., Consani, C., Ghiadoni, L., Filippi, C., Cristofani, R., Panicucci, E., Migliori, M., Sidoti, A., Biagioli, M., Boracelli, D., Barsotti, G. and Tetta, C. | Interleukin-8 is a powerful prognostic predictor of all-cause and cardiovascular mortality in dialytic patients | 2006 | Population data from before 2004 |
| 1105 | Pannier, B., Guerin, A. P., Marchais, S. J., Safar, M. E. and London, G. M. | Stiffness of capacitive and conduit arteries: prognostic significance for end-stage renal disease patients | 2005 | Population data from before 2004 |
| 1106 | Panuccio, V., Tripepi, R., Tripepi, G., Mallamaci, F., Benedetto, F. A., Cataliotti, A., Bellanuova, I., Giacone, G., Malatino, L. S. and Zoccali, C. | Heart valve calcifications, survival, and cardiovascular risk in hemodialysis patients | 2004 | Population data from before 2004 |
| 1107 | Parekh, R. S., Carroll, C. E., Wolfe, R. A. and Port, F. K. | Cardiovascular mortality in children and young adults with end-stage kidney disease | 2002 | Population data from before 2004 |
| 1108 | Parving, H. H., Brenner, B. M., Cooper, M. E., de Zeeuw, D., Keane, W. F., Mitch, W. E., Remuzzi, G., Snapinn, S. M., Zhang, Z. and Shahinfar, S. | Effect of losartan on renal and cardiovascular complications of patients with type 2 diabetes and nephropathy | 2001 | Population data from before 2004 |
| 1109 | Perino, G. C., Ragni, R. and Salomone, M. | Analysis of cause of death in the 1st and last periods of the Piedmontese Dialysis and Transplantation Registry | 1998 | Population data from before 2004 |
| 1110 | Peterson, G. E., de Backer, T., Contreras, G., Wang, X., Kendrick, C., Greene, T., Appel, L. J., Randall, O. S., Lea, J., Smogorzewski, M., Vagaonescu, T., Phillips, R. A. and African American Study of Kidney Disease, I. | Relationship of left ventricular hypertrophy and diastolic function with cardiovascular and renal outcomes in African Americans with hypertensive chronic kidney disease | 2013 | Population data from before 2004 |
| 1111 | Pilz, S., Tomaschitz, A., Friedl, C., Amrein, K., Drechsler, C., Ritz, E., Boehm, B. O., Grammer, T. B. and Marz, W. | Vitamin D status and mortality in chronic kidney disease | 2011 | Population data from before 2004 |
| 1112 | Pizzarelli, F., Lauretani, F., Bandinelli, S., Windham, G. B., Corsi, A. M., Giannelli, S. V., Ferrucci, L. and Guralnik, J. M. | Predictivity of survival according to different equations for estimating renal function in community-dwelling elderly subjects | 2009 | Population data from before 2004 |
| 1113 | Poletti, R., Vergaro, G., Zyw, L., Prontera, C., Passino, C. and Emdin, M. | Prognostic value of plasma renin activity in heart failure patients with chronic kidney disease | 2013 | Population data from before 2004 |
| 1114 | Quinn, M. P., Cardwell, C. R., Kee, F., Maxwell, A. P., Savage, G., McCarron, P. and Fogarty, D. G. | The finding of reduced estimated glomerular filtration rate is associated with increased mortality in a large UK population | 2011 | Population data from before 2004 |
| 1115 | Ramirez, S. P., Albert, J. M., Blayney, M. J., Tentori, F., Goodkin, D. A., Wolfe, R. A., Young, E. W., Bailie, G. R., Pisoni, R. L. and Port, F. K. | Rosiglitazone is associated with mortality in chronic hemodialysis patients | 2009 | Population data from before 2004 |
| 1116 | Ramkumar, N., Murtaugh, M. A., Cheung, A. K. and Beddhu, S. | Lack of synergistic effects of metabolic syndrome and plasma fibrinogen on coronary events and mortality in moderate CKD | 2007 | Population data from before 2004 |
| 1117 | Rao, M., Guo, D., Perianayagam, M. C., Tighiouart, H., Jaber, B. L., Pereira, B. J. and Balakrishnan, V. S. | Plasma interleukin-6 predicts cardiovascular mortality in hemodialysis patients | 2005 | Population data from before 2004 |
| 1118 | Rashidi, A., Sehgal, A. R., Rahman, M. and O'Connor, A. S. | The case for chronic kidney disease, diabetes mellitus, and myocardial infarction being equivalent risk factors for cardiovascular mortality in patients older than 65 years | 2008 | Population data from before 2004 |
| 1119 | Ravani, P., Tripepi, G., Pecchini, P., Mallamaci, F., Malberti, F. and Zoccali, C. | Urotensin II is an inverse predictor of death and fatal cardiovascular events in chronic kidney disease | 2008 | Population data from before 2004 |
| 1120 | Regidor, D. L., Kovesdy, C. P., Mehrotra, R., Rambod, M., Jing, J., McAllister, C. J., Van Wyck, D., Kopple, J. D. and Kalantar-Zadeh, K. | Serum alkaline phosphatase predicts mortality among maintenance hemodialysis patients | 2008 | Population data from before 2004 |
| 1121 | Ricardo, A. C., Grunwald, J. E., Parvathaneni, S., Goodin, S., Ching, A. and Lash, J. P. | Retinopathy and CKD as predictors of all-cause and cardiovascular mortality: National Health and Nutrition Examination Survey (NHANES) 1988-1994 | 2014 | Population data from before 2004 |
| 1122 | Rizk, D. V., Gutierrez, O., Levitan, E. B., McClellan, W. M., Safford, M., Soliman, E. Z., Warnock, D. G. and Muntner, P. | Prevalence and prognosis of unrecognized myocardial infarctions in chronic kidney disease | 2012 | Population data from before 2004 |
| 1123 | Roderick, P. J., Atkins, R. J., Smeeth, L., Mylne, A., Nitsch, D. D., Hubbard, R. B., Bulpitt, C. J. and Fletcher, A. E. | CKD and mortality risk in older people: a community-based population study in the United Kingdom | 2009 | Population data from before 2004 |
| 1124 | Rusinaru, D., Buiciuc, O., Houpe, D. and Tribouilloy, C. | Renal function and long-term survival after hospital discharge in heart failure with preserved ejection fraction | 2011 | Population data from before 2004 |
| 1125 | Ryan, T. P., Fisher, S. G., Elder, J. L., Winters, P. C., Beckett, W., Tacci, J. and Sloand, J. A. | Increased cardiovascular risk associated with reduced kidney function | 2009 | Population data from before 2004 |
| 1126 | Safar, M. E., Blacher, J., Pannier, B., Guerin, A. P., Marchais, S. J., Guyonvarc'h, P. M. and London, G. M. | Central pulse pressure and mortality in end-stage renal disease | 2002 | Population data from before 2004 |
| 1127 | Sakaci, T., Ahbap, E., Koc, Y., Basturk, T., Ucar, Z. A., Sinangil, A., Sevinc, M., Kara, E., Akgol, C., Kayalar, A. O., Caglayan, F. B., Sahutoglu, T. and Unsal, A. | Clinical outcomes and mortality in elderly peritoneal dialysis patients | 2015 | Population data from before 2004 |
| 1128 | Satyan, S., Light, R. P. and Agarwal, R. | Relationships of N-terminal pro-B-natriuretic peptide and cardiac troponin T to left ventricular mass and function and mortality in asymptomatic hemodialysis patients | 2007 | Population data from before 2004 |
| 1129 | Scialla, J. J., Plantinga, L. C., Kao, W. H., Jaar, B., Powe, N. R. and Parekh, R. S. | Soluble P-selectin levels are associated with cardiovascular mortality and sudden cardiac death in male dialysis patients | 2011 | Population data from before 2004 |
| 1130 | Selamet, U., Tighiouart, H., Sarnak, M. J., Beck, G., Levey, A. S., Block, G. and Ix, J. H. | Relationship of dietary phosphate intake with risk of end-stage renal disease and mortality in chronic kidney disease stages 3-5: The Modification of Diet in Renal Disease Study | 2016 | Population data from before 2004 |
| 1131 | Selim, G., Stojceva-Taneva, O., Zafirovska, K., Sikole, A., Gelev, S., Dzekova, P., Stefanovski, K., Koloska, V. and Polenakovic, M. | Inflammation predicts all-cause and cardiovascular mortality in haemodialysis patients | 2006 | Population data from before 2004 |
| 1132 | Shafi, T., Hostetter, T. H., Meyer, T. W., Hwang, S., Hai, X., Melamed, M. L., Banerjee, T., Coresh, J. and Powe, N. R. | Serum Asymmetric and Symmetric Dimethylarginine and Morbidity and Mortality in Hemodialysis Patients | 2016 | Population data from before 2004 |
| 1133 | Shafi, T., Matsushita, K., Selvin, E., Sang, Y., Astor, B. C., Inker, L. A. and Coresh, J. | Comparing the association of GFR estimated by the CKD-EPI and MDRD study equations and mortality: the third national health and nutrition examination survey (NHANES III) | 2012 | Population data from before 2004 |
| 1134 | Shafi, T., Parekh, R. S., Jaar, B. G., Plantinga, L. C., Oberai, P. C., Eckfeldt, J. H., Levey, A. S., Powe, N. R. and Coresh, J. | Serum beta-trace protein and risk of mortality in incident hemodialysis patients | 2012 | Population data from before 2004 |
| 1135 | Shafi, T., Sirich, T. L., Meyer, T. W., Hostetter, T. H., Plummer, N. S., Hwang, S., Melamed, M. L., Banerjee, T., Coresh, J. and Powe, N. R. | Results of the HEMO Study suggest that p-cresol sulfate and indoxyl sulfate are not associated with cardiovascular outcomes | 2017 | Population data from before 2004 |
| 1136 | Shih, C. J., Ou, S. M., Chao, P. W., Kuo, S. C., Lee, Y. J., Yang, C. Y., Tarng, D. C., Lin, C. C., Huang, P. H., Li, S. Y. and Chen, Y. T. | Risks of Death and Stroke in Patients Undergoing Hemodialysis with New-Onset Atrial Fibrillation: A Competing-Risk Analysis of a Nationwide Cohort | 2016 | Population data from before 2004 |
| 1137 | Shinohara, K., Shoji, T., Emoto, M., Tahara, H., Koyama, H., Ishimura, E., Miki, T., Tabata, T. and Nishizawa, Y. | Insulin resistance as an independent predictor of cardiovascular mortality in patients with end-stage renal disease | 2002 | Population data from before 2004 |
| 1138 | Shoji, T., Emoto, M., Shinohara, K., Kakiya, R., Tsujimoto, Y., Kishimoto, H., Ishimura, E., Tabata, T. and Nishizawa, Y. | Diabetes mellitus, aortic stiffness, and cardiovascular mortality in end-stage renal disease | 2001 | Population data from before 2004 |
| 1139 | Shoji, T., Fukumoto, M., Kimoto, E., Shinohara, K., Emoto, M., Tahara, H., Koyama, H., Ishimura, E., Nakatani, T., Miki, T., Tsujimoto, Y., Tabata, T. and Nishizawa, Y. | Antibody to oxidized low-density lipoprotein and cardiovascular mortality in end-stage renal disease | 2002 | Population data from before 2004 |
| 1140 | Shoji, T., Maekawa, K., Emoto, M., Okuno, S., Yamakawa, T., Ishimura, E., Inaba, M. and Nishizawa, Y. | Arterial stiffness predicts cardiovascular death independent of arterial thickness in a cohort of hemodialysis patients | 2010 | Population data from before 2004 |
| 1141 | Shroff, G. R., Li, S. and Herzog, C. A. | Trends in Mortality Following Acute Myocardial Infarction Among Dialysis Patients in the United States Over 15 Years | 2015 | Population data from before 2004 |
| 1142 | Siemensen, H., Schlamp, R., Tachezy, H., Bischoff, K. and Remmecke, J. | Survival time and causes of death in dialysis patients | 1984 | Population data from before 2004 |
| 1143 | Sood, M. M., Larkina, M., Thumma, J. R., Tentori, F., Gillespie, B. W., Fukuhara, S., Mendelssohn, D. C., Chan, K., de Sequera, P., Komenda, P., Rigatto, C. and Robinson, B. M. | Major bleeding events and risk stratification of antithrombotic agents in hemodialysis: results from the DOPPS | 2013 | Population data from before 2004 |
| 1144 | Stallworthy, E. J., Pilmore, H. L., Webster, M. W., Sidhu, K. K., Curry, E. M., Brown, P. and Scaria, A. | Do echocardiographic parameters predict mortality in patients with end-stage renal disease? | 2013 | Population data from before 2004 |
| 1145 | Staplin, N., Haynes, R., Herrington, W. G., Reith, C., Cass, A., Fellstrom, B., Jiang, L., Kasiske, B. L., Krane, V., Levin, A., Walker, R., Wanner, C., Wheeler, D. C., Landray, M. J., Baigent, C. and Emberson, J. | Smoking and Adverse Outcomes in Patients With CKD: The Study of Heart and Renal Protection (SHARP) | 2016 | Population data from before 2004 |
| 1146 | Stehouwer, C. D., Gall, M. A., Hougaard, P., Jakobs, C. and Parving, H. H. | Plasma homocysteine concentration predicts mortality in non-insulin-dependent diabetic patients with and without albuminuria | 1999 | Population data from before 2004 |
| 1147 | Stengel, B., Metzger, M., Froissart, M., Rainfray, M., Berr, C., Tzourio, C. and Helmer, C. | Epidemiology and prognostic significance of chronic kidney disease in the elderly--the Three-City prospective cohort study | 2011 | Population data from before 2004 |
| 1148 | Stenvinkel, P., Wanner, C., Metzger, T., Heimburger, O., Mallamaci, F., Tripepi, G., Malatino, L. and Zoccali, C. | Inflammation and outcome in end-stage renal failure: does female gender constitute a survival advantage? | 2002 | Population data from before 2004 |
| 1149 | Stojceva-Taneva, O., Selim, G. J., Tozija, L. and Polenakovic, M. | Early mortality rate in end-stage renal disease patients initiating hemodialysis | 2006 | Population data from before 2004 |
| 1150 | Sud, M., Tangri, N., Pintilie, M., Levey, A. S. and Naimark, D. | Risk of end-stage renal disease and death after cardiovascular events in chronic kidney disease | 2014 | Population data from before 2004 |
| 1151 | Sun, J., Axelsson, J., Machowska, A., Heimburger, O., Barany, P., Lindholm, B., Lindstrom, K., Stenvinkel, P. and Qureshi, A. R. | Biomarkers of Cardiovascular Disease and Mortality Risk in Patients with Advanced CKD | 2016 | Population data from before 2004 |
| 1152 | Sun, L., Sun, Y., Zhao, X., Xu, C., Chen, D., Li, L., Ma, Y., Rong, S. and Mei, C. | Predictive role of BNP and NT-proBNP in hemodialysis patients | 2008 | Population data from before 2004 |
| 1153 | Suto, Y., Itoh, A., Otsuka, M., Yamashita, H., Ehara, S., Kawarai, H., Naruko, T., Tojo, O. and Haze, K. | Prognosis for patients with angina pectoris accompanied by chronic renal failure | 1999 | Population data from before 2004 |
| 1154 | Szpakowicz, A., Pepinski, W., Waszkiewicz, E., Maciorkowska, D., Skawronska, M., Niemcunowicz-Janica, A., Dobrzycki, S., Musial, W. J. and Kaminski, K. A. | The influence of renal function on the association of rs854560 polymorphism of paraoxonase 1 gene with long-term prognosis in patients after myocardial infarction | 2016 | Population data from before 2004 |
| 1155 | Takahashi, H., Ishii, H., Aoyama, T., Kamoi, D., Kasuga, H., Ito, Y., Yasuda, K., Tanaka, M., Yoshikawa, D., Maruyama, S., Matsuo, S., Murohara, T. and Yuzawa, Y. | Association of cardiac valvular calcifications and C-reactive protein with cardiovascular mortality in incident hemodialysis patients: a Japanese cohort study | 2013 | Population data from before 2004 |
| 1156 | Takahashi, H., Ito, Y., Ishii, H., Aoyama, T., Kamoi, D., Kasuga, H., Yasuda, K., Maruyama, S., Matsuo, S., Murohara, T. and Yuzawa, Y. | Geriatric nutritional risk index accurately predicts cardiovascular mortality in incident hemodialysis patients | 2014 | Population data from before 2004 |
| 1157 | Tang, C. H., Chen, T. H., Wang, C. C., Hong, C. Y., Huang, K. C. and Sue, Y. M. | Renin-angiotensin system blockade in heart failure patients on long-term haemodialysis in Taiwan | 2013 | Population data from before 2004 |
| 1158 | Tang, S. C. W., Lam, B., Yao, T. J., Leung, W. S., Chu, C. M., Ho, Y. W., Ip, M. S. M. and Lai, K. N. | Sleep apnea is a novel risk predictor of cardiovascular morbidity and death in patients receiving peritoneal dialysis | 2010 | Population data from before 2004 |
| 1159 | Teng, G. G., Ang, L. W., Saag, K. G., Yu, M. C., Yuan, J. M. and Koh, W. P. | Mortality due to coronary heart disease and kidney disease among middle-aged and elderly men and women with gout in the Singapore Chinese Health Study | 2012 | Population data from before 2004 |
| 1160 | Tentori, F., Blayney, M. J., Albert, J. M., Gillespie, B. W., Kerr, P. G., Bommer, J., Young, E. W., Akizawa, T., Akiba, T., Pisoni, R. L., Robinson, B. M. and Port, F. K. | Mortality risk for dialysis patients with different levels of serum calcium, phosphorus, and PTH: the Dialysis Outcomes and Practice Patterns Study (DOPPS) | 2008 | Population data from before 2004 |
| 1161 | Tepel, M., van der Giet, M., Statz, M., Jankowski, J. and Zidek, W. | The antioxidant acetylcysteine reduces cardiovascular events in patients with end-stage renal failure: a randomized, controlled trial | 2003 | Population data from before 2004 |
| 1162 | Terrier, N., Jaussent, I., Dupuy, A. M., Morena, M., Delcourt, C., Chalabi, L., Rouanet, C., Canaud, B. and Cristol, J. P. | Creatinine index and transthyretin as additive predictors of mortality in haemodialysis patients | 2008 | Population data from before 2004 |
| 1163 | Tofik, R., Torffvit, O., Rippe, B. and Bakoush, O. | Increased urine IgM excretion predicts cardiovascular events in patients with type 1 diabetes nephropathy | 2009 | Population data from before 2004 |
| 1164 | Tokmakova, M. P., Skali, H., Kenchaiah, S., Braunwald, E., Rouleau, J. L., Packer, M., Chertow, G. M., Moye, L. A., Pfeffer, M. A. and Solomon, S. D. | Chronic kidney disease, cardiovascular risk, and response to angiotensin-converting enzyme inhibition after myocardial infarction: the Survival And Ventricular Enlargement (SAVE) study | 2004 | Population data from before 2004 |
| 1165 | Tong, M., Carrero, J. J., Qureshi, A. R., Anderstam, B., Heimburger, O., Barany, P., Axelsson, J., Alvestrand, A., Stenvinkel, P., Lindholm, B. and Suliman, M. E. | Plasma pentraxin 3 in patients with chronic kidney disease: associations with renal function, protein-energy wasting, cardiovascular disease, and mortality | 2007 | Population data from before 2004 |
| 1166 | Torle ́n, K., Kalantar-Zadeh, K., Molnar, M. Z., Vashistha, T. and Mehrotra, R. | Serum potassium and cause-specific mortality in a large peritoneal dialysis cohort | 2012 | Population data from before 2004 |
| 1167 | Tripepi, G., Fagugli, R. M., Dattolo, P., Parlongo, G., Mallamaci, F., Buoncristiani, U. and Zoccali, C. | Prognostic value of 24-hour ambulatory blood pressure monitoring and of night/day ratio in nondiabetic, cardiovascular events-free hemodialysis patients | 2005 | Population data from before 2004 |
| 1168 | Trivedi, H., Xiang, Q. and Klein, J. P. | Risk factors for non-fatal myocardial infarction and cardiac death in incident dialysis patients | 2009 | Population data from before 2004 |
| 1169 | Tsagalis, G., Akrivos, T., Alevizaki, M., Manios, E., Stamatellopoulos, K., Laggouranis, A. and Vemmos, K. N. | Renal dysfunction in acute stroke: an independent predictor of long-term all combined vascular events and overall mortality | 2009 | Population data from before 2004 |
| 1170 | Tsai, T. H., Chen, Y. L., Chen, S. M., Yang, C. H., Fang, C. Y., Hsieh, Y. K., Wu, C. J., Yip, H. K., Hang, C. L., Fu, M. and Chen, M. C. | Uric Acid is not an independent predictor of cardiovascular death in patients with angiographically proven coronary artery disease | 2009 | Population data from before 2004 |
| 1171 | Tschope, W., Koch, M., Thomas, B. and Ritz, E. | Serum lipids predict cardiac death in diabetic patients on maintenance hemodialysis. Results of a prospective study. The German Study Group Diabetes and Uremia | 1993 | Population data from before 2004 |
| 1172 | Tzamaloukas, A. H., Zager, P. G., Harford, A. M., Nevarez, M., Quintana, B. J., Avasthi, P. S. and Gibel, L. J. | Vascular disease: the critical risk factor for mortality in older patients on CAPD | 1990 | Population data from before 2004 |
| 1173 | Ueda, H., Hayashi, T., Tsumura, K., Yoshimaru, K., Nakayama, Y. and Yoshikawa, J. | Inflection point of ascending aortic waveform is a predictive factor for all-cause and cardiovascular mortality in patients with chronic renal failure on hemodialysis | 2004 | Population data from before 2004 |
| 1174 | Unsal, A., Koc, Y., Basturk, T., Sakaci, T., Ahbap, E., Sinangil, A., Budak, S. K., Sevinc, M., Kara, E. and Doner, B. | Clinical outcomes and mortality in peritoneal dialysis patients: a 10-year retrospective analysis in a single center | 2013 | Population data from before 2004 |
| 1175 | Van Biesen, W., De Bacquer, D., Verbeke, F., Delanghe, J., Lameire, N. and Vanholder, R. | The glomerular filtration rate in an apparently healthy population and its relation with cardiovascular mortality during 10 years | 2007 | Population data from before 2004 |
| 1176 | van Dijk, S., van den Beukel, T. O., Dekker, F. W., le Cessie, S., Kaptein, A. A., Honig, A., Siegert, C. E., Boeschoten, E. W., Krediet, R. T. and Verduijn, M. | Short-term versus long-term effects of depressive symptoms on mortality in patients on dialysis | 2012 | Population data from before 2004 |
| 1177 | van Kuijk, J. P., Flu, W. J., Chonchol, M., Welten, G. M., Verhagen, H. J., Bax, J. J. and Poldermans, D. | The prevalence and prognostic implications of polyvascular atherosclerotic disease in patients with chronic kidney disease | 2010 | Population data from before 2004 |
| 1178 | Varma, R., Aronow, W. S., McClung, J. A., Garrick, R., Vistainer, P. F., Weiss, M. B. and Belkin, R. N. | Prevalence of valve calcium and association of valve calcium with coronary artery disease, atherosclerotic vascular disease, and all-cause mortality in 137 patients undergoing hemodialysis for chronic renal failure | 2005 | Population data from before 2004 |
| 1179 | Vicari, A. M., Taglietti, M. V., Pellegatta, F., Spotti, D., Melandri, M., Galli, L., Ronchi, P. and Folli, F. | Deranged platelet calcium homeostasis in diabetic patients with end-stage renal failure. A possible link to increased cardiovascular mortality? | 1996 | Population data from before 2004 |
| 1180 | Wagner, Z., Molnar, M., Molnar, G. A., Tamasko, M., Laczy, B., Wagner, L., Csiky, B., Heidland, A., Nagy, J. and Wittmann, I. | Serum carboxymethyllysine predicts mortality in hemodialysis patients | 2006 | Population data from before 2004 |
| 1181 | Wakasugi, M., Kazama, J. J. and Narita, I. | Mortality trends among Japanese dialysis patients, 1988-2013: A joinpoint regression analysis | 2016 | Population data from before 2004 |
| 1182 | Wallen, M. D., Radhakrishnan, J., Appel, G., Hodgson, M. E. and Pablos-Mendez, A. | An analysis of cardiac mortality in patients with new-onset end-stage renal disease in New York State | 2001 | Population data from before 2004 |
| 1183 | Wang, A. Y., Lam, C. W., Wang, M., Chan, I. H., Goggins, W. B., Yu, C. M., Lui, S. F. and Sanderson, J. E. | Prognostic value of cardiac troponin T is independent of inflammation, residual renal function, and cardiac hypertrophy and dysfunction in peritoneal dialysis patients | 2007 | Population data from before 2004 |
| 1184 | Wang, A. Y., Lam, C. W., Yu, C. M., Wang, M., Chan, I. H., Zhang, Y., Lui, S. F. and Sanderson, J. E. | N-terminal pro-brain natriuretic peptide: an independent risk predictor of cardiovascular congestion, mortality, and adverse cardiovascular outcomes in chronic peritoneal dialysis patients | 2007 | Population data from before 2004 |
| 1185 | Wang, A. Y., Sea, M. M., Tang, N., Sanderson, J. E., Lui, S. F., Li, P. K. and Woo, J. | Resting energy expenditure and subsequent mortality risk in peritoneal dialysis patients | 2004 | Population data from before 2004 |
| 1186 | Wang, A. Y., Wang, M., Woo, J., Lam, C. W., Lui, S. F., Li, P. K. and Sanderson, J. E. | Inflammation, residual kidney function, and cardiac hypertrophy are interrelated and combine adversely to enhance mortality and cardiovascular death risk of peritoneal dialysis patients | 2004 | Population data from before 2004 |
| 1187 | Wang, Z. and Hoy, W. E. | The predictive value of albuminuria for renal and nonrenal natural deaths over 14 years follow-up in a remote aboriginal community | 2012 | Population data from before 2004 |
| 1188 | Wanner, C. and Metzger, T. | C-reactive protein a marker for all-cause and cardiovascular mortality in haemodialysis patients | 2002 | Population data from before 2004 |
| 1189 | Wetmore, J. B., Liu, J., Li, S., Hu, Y., Peng, Y., Gilbertson, D. T. and Collins, A. J. | The Healthy People 2020 Objectives for Kidney Disease: How Far Have We Come, and Where Do We Need to Go? | 2017 | Population data from before 2004 |
| 1190 | Xiong, Z., Xu, H., Huang, X., Arnlov, J., Qureshi, A. R., Cederholm, T., Sjogren, P., Lindholm, B., Riserus, U. and Carrero, J. J. | Nonesterified fatty acids and cardiovascular mortality in elderly men with CKD | 2015 | Population data from before 2004 |
| 1191 | Yahalom, G., Schwartz, R., Schwammenthal, Y., Merzeliak, O., Toashi, M., Orion, D., Sela, B. A. and Tanne, D. | Chronic kidney disease and clinical outcome in patients with acute stroke | 2009 | Population data from before 2004 |
| 1192 | Yoshino, M., Kuhlmann, M. K., Kotanko, P., Greenwood, R. N., Pisoni, R. L., Port, F. K., Jager, K. J., Homel, P., Augustijn, H., De Charro, F. T., Collart, F., Erek, E., Finne, P., Garcia-Garcia, G., Grönhagen-Riska, C., Ioannidis, G. A., Ivis, F., Leivestad, T., Løkkegaard, H., Lopot, F., Jin, D. C., Kramar, R., Nakao, T., Nandakumar, M., Ramirez, S., Van Der Sande, F. M., Schön, S., Simpson, K., Walker, R. G., Zaluska, W. and Levin, N. W. | International differences in dialysis mortality reflect background general population atherosclerotic cardiovascular mortality | 2006 | Population data from before 2004 |
| 1193 | Young, E. W., Albert, J. M., Satayathum, S., Goodkin, D. A., Pisoni, R. L., Akiba, T., Akizawa, T., Kurokawa, K., Bommer, J., Piera, L. and Port, F. K. | Predictors and consequences of altered mineral metabolism: the Dialysis Outcomes and Practice Patterns Study | 2005 | Population data from before 2004 |
| 1194 | Yu, W. C., Lin, Y. P., Chuang, S. Y., Lin, I. F. and Chenb, C. H. | Cardiovascular determinants of prognosis in normotensive hemodialysis patients | 2012 | Population data from before 2004 |
| 1195 | Xiang (2018) | Monocyte/lymphocyte ratio as a better predictor of cardiovascular and all-cause mortality in hemodialysis patients: A prospective cohort study | 2018 | Reports with duplicate data |
| 1196 | Hannedouche (2016) | Multiphasic effects of blood pressure on survival in hemodialysis patients | 2016 | Reports with duplicate data |
| 1197 | Jiang (2018) | Serum Albumin at Start of Peritoneal Dialysis Predicts Long-Term Outcomes in Anhui Han Patients on Continuous Ambulatory Peritoneal Dialysis: A Retrospective Cohort Study | 2018 | Reports with duplicate data |
| 1198 | Wen (2015) | High glucose concentrations in peritoneal dialysate are associated with all-cause and cardiovascular disease mortality in continuous ambulatory peritoneal dialysis patients | 2015 | Reports with duplicate data |
| 1199 | Ye (2018) | Serum magnesium and cardiovascular mortality in peritoneal dialysis patients: A 5-year prospective cohort study | 2018 | Reports with duplicate data |
| 1200 | Li (2017) | Association of baseline, longitudinal serum high-sensitive C-reactive protein and its change with mortality in peritoneal dialysis patients | 2017 | Reports with duplicate data |
| 1201 | Echida (2012) | Serum non-high-density lipoprotein cholesterol (non-HDL-C) levels and cardiovascular mortality in chronic hemodialysis patients | 2012 | Reports with duplicate data |
| 1202 | Antunovic (2013) | High uric acid and low superoxide dismutase as possible predictors of all-cause and cardiovascular mortality in hemodialysis patients | 2013 | Reports with duplicate data |
| 1203 | Park (2015) | Serum Ferritin Predicts Mortality Regardless of Inflammatory and Nutritional Status in Patients Starting Dialysis: A Prospective Cohort Study | 2015 | Reports with duplicate data |
| 1204 | Benjamin, E. J., Blaha, M. J., Chiuve, S. E., Cushman, M., Das, S. R., Deo, R., De Ferranti, S. D., Floyd, J., Fornage, M., Gillespie, C., Isasi, C. R., Jim'nez, M. C., Jordan, L. C., Judd, S. E., Lackland, D., Lichtman, J. H., Lisabeth, L., Liu, S., Longenecker, C. T., MacKey, R. H., Matsushita, K., Mozaffarian, D., Mussolino, M. E., Nasir, K., Neumar, R. W., Palaniappan, L., Pandey, D. K., Thiagarajan, R. R., Reeves, M. J., Ritchey, M., Rodriguez, C. J., Roth, G. A., Rosamond, W. D., Sasson, C., Towfghi, A., Tsao, C. W., Turner, M. B., Virani, S. S., Voeks, J. H., Willey, J. Z., Wilkins, J. T., Wu, J. H. Y., Alger, H. M., Wong, S. S. and Muntner, P. | Heart Disease and Stroke Statistics'2017 Update: A Report from the American Heart Association | 2017 | Review/Meta-analysis |
| 1205 | Courivaud, C. and Davenport, A. | Magnesium and the risk of all-cause and cardiac mortality in hemodialysis patients: agent provocateur or innocent bystander? | 2014 | Review/Meta-analysis |
| 1206 | Eknoyan, G. | Cardiovascular mortality and morbidity in dialysis patients | 1999 | Review/Meta-analysis |
| 1207 | Fabbian, F., Dentali, F., Ageno, W. and Manfredini, R. | Mortality due to pulmonary embolism, myocardial infarction, and stroke among incident dialysis patients: A rebuttal | 2013 | Review/Meta-analysis |
| 1208 | Herzog, C. A. | Sudden cardiac death and acute myocardial infarction in dialysis patients: perspectives of a cardiologist | 2005 | Review/Meta-analysis |
| 1209 | Kuhlmann, M. K., Yoshino, M. and Levin, N. W. | Differences in cardiovascular mortality rates among hemodialysis patients in the United States and Japan: The importance of background cardiovascular mortality | 2004 | Review/Meta-analysis |
| 1210 | Remon Rodriguez, C. and Quiros Ganga, P. L. | Current evidence shows that survival outcomes are equivalent for dialysis techniques | 2011 | Review/Meta-analysis |
| 1211 | Talbot, B., Sukkar, L., Smyth, B., Jun, M., Jardine, M., Cass, A., Walker, R., Reith, C., Hooi, L. and Gallagher, M. | Cause of death varies across australia, new zealand and malaysia in those on renal replacement therapy - Results from the study of heart and renal protection-extended review (sharp-er) | 2018 | Review/Meta-analysis |
| 1212 | Zoccali, C., Tripepi, G. and Mallamaci, F. | Predictors of cardiovascular death in ESRD | 2005 | Review/Meta-analysis |
| 1213 | Brancaccio, D., Biondi, M. L., Gallieni, M., Turri, O., Galassi, A., Cecchini, F., Russo, D., Andreucci, V. and Cozzolino, M. | Matrix GLA protein gene polymorphisms: clinical correlates and cardiovascular mortality in chronic kidney disease patients | 2005 | Study duration undefined |
| 1214 | Cafka, M., Rroji, M., Seferi, S., Barbullushi, M., Burazeri, G., Spahia, N., Idrizi, A., Likaj, E., Seiti, J., Lazaj, J. and Goda, A. | Inflammation, Left Ventricular Hypertrophy, and Mortality in End-stage Renal Disease | 2016 | Study duration undefined |
| 1215 | Chen, K. H., Lin-Tan, D. T., Huang, W. H., Hung, C. C., Chang, C. T., Huang, J. Y. and Lin, J. L. | Cardiothoracic ratio, malnutrition, inflammation, and two-year mortality in non-diabetic patients on maintenance hemodialysis | 2008 | Study duration undefined |
| 1216 | Chmielewski, M., Bragfors-Helin, A. C., Stenvinkel, P., Lindholm, B. and Anderstam, B. | Serum soluble CD36, assessed by a novel monoclonal antibody-based sandwich ELISA, predicts cardiovascular mortality in dialysis patients | 2010 | Study duration undefined |
| 1217 | Cuevas, X., Garcia, F., Martin-Malo, A., Fort, J., Llados, F., Lozano, J. and Perez-Garcia, R. | Risk factors associated with cardiovascular morbidity and mortality in Spanish incident hemodialysis patients: two-year results from the ANSWER study | 2012 | Study duration undefined |
| 1218 | Dong, J., Han, Q. F., Zhu, T. Y., Ren, Y. P., Chen, J. H., Zhao, H. P., Chen, M. H., Xu, R., Wang, Y., Hao, C. M., Zhang, R., Zhang, X. H., Wang, M., Tian, N. and Wang, H. Y. | The associations of uric acid, cardiovascular and all-cause mortality in peritoneal dialysis patients | 2014 | Study duration undefined |
| 1219 | Duman, D., Tokay, S., Toprak, A., Duman, D., Oktay, A., Ozener, I. C. and Unay, O. | Elevated cardiac troponin T is associated with increased left ventricular mass index and predicts mortality in continuous ambulatory peritoneal dialysis patients | 2005 | Study duration undefined |
| 1220 | Furuhashi, M., Ishimura, S., Ota, H., Hayashi, M., Nishitani, T., Tanaka, M., Yoshida, H., Shimamoto, K., Hotamisligil, G. S. and Miura, T. | Serum fatty acid-binding protein 4 is a predictor of cardiovascular events in end-stage renal disease | 2011 | Study duration undefined |
| 1221 | Hocher, B., Ziebig, R., Krause, R., Asmus, G., Neumayer, H. H., Liefeldt, L. and Stasch, J. P. | Relaxin is an independent risk factor predicting death in male patients with end-stage kidney disease | 2004 | Study duration undefined |
| 1222 | Janda, K., Krzanowski, M., Dumnicka, P., Kusnierz-Cabala, B., Sorysz, D. and Sulowicz, W. | Hepatocyte growth factor as a long-term predictor for total and cardiovascular mortality in patients on peritoneal dialysis | 2013 | Study duration undefined |
| 1223 | Jaroszynski, A., Jaroszynska, A., Siebert, J., Dabrowski, W., Niedzialek, J., Bednarek-Skublewska, A., Zapolski, T., Wysokinski, A., Zaluska, W., Ksiazek, A. and Schlegel, T. T. | The prognostic value of positive T-wave in lead aVR in hemodialysis patients | 2015 | Study duration undefined |
| 1224 | Jaroszyński, A. J., Jaroszyńska, A., Przywara, S., Zaborowski, T., Ksiazek, A. and Dabrowski, W. | Syndecan-4 is an independent predictor of all-cause as well as cardiovascular mortality in hemodialysis patients | 2016 | Study duration undefined |
| 1225 | Karakitsos, D., Wachtel, M., Zerefos, N., Valis, D., Patrianakos, A., Saranteas, T., Daphnis, E., Boletis, J., Stefanadis, C. and Karabinis, A. | Prognostic utility of impedance cardiography measurements in elderly hemodialysis patients with coronary artery disease | 2009 | Study duration undefined |
| 1226 | Kimura, H., Tanaka, K., Kanno, M., Watanabe, K., Hayashi, Y., Asahi, K., Suzuki, H., Sato, K., Sakaue, M., Terawaki, H., Nakayama, M., Miyata, T. and Watanabe, T. | Skin Autofluorescence Predicts Cardiovascular Mortality in Patients on Chronic Hemodialysis | 2014 | Study duration undefined |
| 1227 | Lobo, J. C., Stockler-Pinto, M. B., Farage, N. E., Faulin Tdo, E., Abdalla, D. S., Torres, J. P., Velarde, L. G. and Mafra, D. | Reduced plasma zinc levels, lipid peroxidation, and inflammation biomarkers levels in hemodialysis patients: implications to cardiovascular mortality | 2013 | Study duration undefined |
| 1228 | London, G. M., Safar, M. E. and Pannier, B. | Aortic Aging in ESRD: Structural, Hemodynamic, and Mortality Implications | 2016 | Study duration undefined |
| 1229 | Ortega, O., Rodriguez, I., Cobo, G., Hinostroza, J., Gallar, P., Mon, C., Ortiz, M., Herrero, J. C., Di Gioia, C., Oliet, A. and Vigil, A. | Lack of influence of serum magnesium levels on overall mortality and cardiovascular outcomes in patients with advanced chronic kidney disease | 2013 | Study duration undefined |
| 1230 | Paniagua, R., Ventura, M. D., Avila-Diaz, M., Hinojosa-Heredia, H., Mendez-Duran, A., Cueto-Manzano, A., Cisneros, A., Ramos, A., Madonia-Juseino, C., Belio-Caro, F., Garcia-Contreras, F., Trinidad-Ramos, P., Vazquez, R., Ilabaca, B., Alcantara, G. and Amato, D. | NT-proBNP, fluid volume overload and dialysis modality are independent predictors of mortality in ESRD patients | 2010 | Study duration undefined |
| 1231 | Sevinc Ok, E., Kircelli, F., Asci, G., Altunel, E., Ertilav, M., Sipahi, S., Bozkurt, D., Duman, S., Ozkahya, M., Toz, H. and Ok, E. | Neither oxidized nor anti-oxidized low-density lipoprotein level is associated with atherosclerosis or mortality in hemodialysis patients | 2012 | Study duration undefined |
| 1232 | Spoto, B., Mattace-Raso, F., Sijbrands, E., Pizzini, P., Cutrupi, S., D'Arrigo, G., Tripepi, G., Zoccali, C. and Mallamaci, F. | Resistin and all-cause and cardiovascular mortality: effect modification by adiponectin in end-stage kidney disease patients | 2013 | Study duration undefined |
| 1233 | Stenvinkel, P., Diczfalusy, U., Lindholm, B. and Heimburger, O. | Phospholipid plasmalogen, a surrogate marker of oxidative stress, is associated with increased cardiovascular mortality in patients on renal replacement therapy | 2004 | Study duration undefined |
| 1234 | Stenvinkel, P., Heimburger, O. and Lindholm, B. | Wasting, but not malnutrition, predicts cardiovascular mortality in end-stage renal disease | 2004 | Study duration undefined |
| 1235 | Suliman, M. E., Qureshi, A. R., Heimburger, O., Lindholm, B. and Stenvinkel, P. | Soluble adhesion molecules in end-stage renal disease: a predictor of outcome | 2006 | Study duration undefined |
| 1236 | Testa, A., Spoto, B., Tripepi, G., Mallamaci, F., Malatino, L., Fatuzzo, P., Maas, R., Boeger, R. and Zoccali, C. | The GLU298ASP variant of nitric oxide synthase interacts with asymmetric dimethyl arginine in determining cardiovascular mortality in patients with end-stage renal disease | 2005 | Study duration undefined |
| 1237 | Vichairuangthum, K., Leowattana, W., Ong-Ajyooth, L. and Pokum, S. | The relationship between serum concentration of cardiac troponin I in chronic renal failure patients and cardiovascular events | 2006 | Study duration undefined |
| 1238 | Wang, A. Y., Lam, C. W., Chan, I. H., Wang, M., Lui, S. F. and Sanderson, J. E. | Long-term mortality and cardiovascular risk stratification of peritoneal dialysis patients using a combination of inflammation and calcification markers | 2009 | Study duration undefined |
| 1239 | Wei, S. Y., Huang, J. C., Chen, S. C., Chang, J. M. and Chen, H. C. | Unequal arterial stiffness with overall and cardiovascular mortality in patients receiving hemodialysis | 2016 | Study duration undefined |
| 1240 | Weng, C. H., Hu, C. C., Yen, T. H., Hsu, C. W. and Huang, W. H. | Uremic Pruritus is Associated with Two-Year Cardiovascular Mortality in Long Term Hemodialysis Patients | 2018 | Study duration undefined |
| 1241 | Winther, S., Christensen, J. H., Flyvbjerg, A., Schmidt, E. B., Jorgensen, K. A., Skou-Jorgensen, H. and Svensson, M. | Osteoprotegerin and mortality in hemodialysis patients with cardiovascular disease | 2013 | Study duration undefined |
| 1242 | Ashley, J., McArthur, E., Bota, S., Harel, Z., Battistella, M., Molnar, A. O., Jun, M., Badve, S. V., Garg, A. X., Manuel, D., Tanuseputro, P., Wells, P., Mavrakanas, T., Rhodes, E., Sood, M. M. | Risk of Cardiovascular Events and Mortality Among Elderly Patients With Reduced GFR Receiving Direct Oral Anticoagulants | 2020 | Unpublished results |
| 1243 | Shardlow, A., McIntyre, N. J., Kolhe, N. V., Nellums, L. B., Fluck, R. J., McIntyre, C. W., Taal, M. W. | The association of skin autofluorescence with cardiovascular events and all-cause mortality in persons with chronic kidney disease stage 3: A prospective cohort study | 2020 | Unpublished results |
| 1244 | Yang, K., Yang, J., Bi, X., Yu, Z., Xiao, T., Huang, Y., Liu, Y., Xiong, J., Zhao, J. | Serum Klotho, Cardiovascular Events, and Mortality in Nondiabetic Chronic Kidney Disease | 2020 | Unpublished results |
| 1245 | Zhan, X., Pan, D., Wei, X. Wen, D., Yan, C., Xiao, J. | Monocyte to high-density lipoprotein ratio and cardiovascular events in patients on peritoneal dialysis | 2020 | Unpublished results |
| 1246 | Zhong, Z., Peng, F., Shi, D., Peng, Y., Li, B., Xiao, M., Feng, S., Mao, H. Huang, F., Yang, X., Li, J., Li, Z. | Serum lipoprotein(a) and risk of mortality in patients on peritoneal dialysis | 2020 | Unpublished results |
| 1247 | Euctr, I. T. | Intensive Phosphate Control in Development of Renal End Points and Mortality in CKD (the IPeR Study) | 2012 | Unpublished results |
| 1248 | Kalantar-Zadeh, K., Johansson, J., Kulikowski, E., Halliday, C., Lebioda, K., Sweeney, M., Wong, N., Nicholls, S., Schwartz, G. and Ray, K. | Design features of the betonmace chronic kidney disease sub-study; Effects of the selective betinhibitor apabetalone on kidney function and mace in post-ACS patients with estimated glomerular filtration rate below 60 and diabetes | 2018 | Unpublished results |
| 1249 | Nakamura, S., Kawano, Y., Hase, H., Hatta, T., Nishimura, S., Moroi, M., Nakagawa, S., Kasai, T., Kusuoka, H., Takeishi, Y., Nakajima, K., Momose, M., Takehana, K., Nanasato, M., Yoda, S., Nishina, H., Matsumoto, N. and Nishimura, T. | Prognostic study of cardiac and renal events in Japanese patients with chronic kidney disease and cardiovascular risk using myocardial perfusion SPECT: J-ACCESS 3 study design | 2010 | Unpublished results |
| 1250 |  | A Study to Evaluate the Effect of Dapagliflozin on Renal Outcomes and Cardiovascular Mortality in Patients with Chronic Kidney Disease | 2017 | Unpublished results |
| 1251 | Thethi, I., Bansal, V., Khan, H., Hoppensteadt, D. and Fareed, J. | Assessment of levels of vascular endothelial growth factor in patients with ESRD and its possible role in cardiovascular morbidity and mortality | 2012 | Unpublished results |
