# Supplementary material for: Relationship between sex and cardiovascular mortality in chronic kidney disease: A systematic review and meta-analysis
Source: PLoS One. 2021 Jul 12;16(7):e0254554. doi: 10.1371/journal.pone.0254554 (PMC8274915; doi:10.1371/journal.pone.0254554)
Supplement: S2 Table — (DOCX) [file pone.0254554.s006.docx]

**S2 Table. Risk of Bias summary for individual studies**

| **Study** | **Similar distribution of men and women in the study population** | **Study controlled for age and diabetes mellitus** | **Study controlled for other confounders** | **Source of outcome data** | **Was comprehensive data reported to estimate sex differences?** | **Adequate length of follow-up for outcomes to occur** | **Adequacy of follow-up of study population** | **NOS score out of 7** | **Quality** |
| --- | --- | --- | --- | --- | --- | --- | --- | --- | --- |
| Wang 2020 | 0 | 0 | 0 | 1 | 0 | 1 | 1 | 3 | Low |
| Zhang 2020 | 0 | 0 | 0 | 1 | 0 | 1 | 0 | 2 | Low |
| Yu 2020 | 1 | 1 | 1 | 1 | 0 | 1 | 1 | 6 | High |
| Lee 2020 | 1 | 0 | 0 | 1 | 0 | 1 | 1 | 4 | High |
| Tsai 2020 | 1 | 0 | 0 | 1 | 0 | 1 | 1 | 4 | High |
| Simsek 2020 | 0 | 1 | 1 | 1 | 0 | 1 | 1 | 5 | High |
| Toyama 2019 | 0 | 0 | 0 | 1 | 0 | 1 | 1 | 3 | Low |
| Mizuiri 2019 | 0 | 0 | 0 | 1 | 0 | 1 | 1 | 3 | Low |
| Chen 2019 | 0 | 1 | 1 | 1 | 0 | 1 | 1 | 5 | High |
| Yadav 2019 | 0 | 0 | 0 | 1 | 0 | 1 | 1 | 3 | Low |
| Cano-Megias 2019 | 1 | 0 | 0 | 1 | 0 | 1 | 0 | 3 | Low |
| Wu 2019 | 1 | 0 | 0 | 1 | 0 | 1 | 1 | 4 | High |
| Saglimbene 2019 | 1 | 1 | 1 | 1 | 0 | 1 | 1 | 6 | High |
| Gong 2018 | 1 | 0 | 0 | 1 | 0 | 1 | 0 | 3 | Low |
| Zhang 2017 | 0 | 1 | 1 | 1 | 0 | 0 | 1 | 4 | High |
| Wu 2017 | 1 | 1 | 1 | 1 | 0 | 1 | 1 | 6 | High |
| Peng 2017 | 0 | 0 | 0 | 1 | 0 | 1 | 1 | 3 | Low |
| Jeng 2017 | 1 | 0 | 0 | 1 | 0 | 1 | 1 | 4 | High |
| Isla 2016 | 1 | 0 | 0 | 1 | 1 | 1 | 0 | 4 | High |
| Lu 2016 | 1 | 1 | 1 | 1 | 0 | 1 | 1 | 6 | High |
| Merle 2016 | 0 | 1 | 1 | 1 | 0 | 1 | 0 | 4 | High |
| Chen 2015 | 1 | 0 | 0 | 1 | 0 | 1 | 0 | 3 | Low |
| Flythe 2015 | 1 | 1 | 1 | 1 | 0 | 1 | 0 | 5 | High |
| Tsai 2015 | 1 | 1 | 1 | 1 | 0 | 1 | 1 | 6 | High |
| Oh 2015 | 0 | 1 | 1 | 1 | 0 | 0 | 1 | 4 | High |
| Yoshitomi 2014 | 0 | 0 | 0 | 1 | 0 | 0 | 0 | 1 | Low |
| Okamoto 2014 | 0 | 0 | 0 | 1 | 0 | 1 | 0 | 2 | Low |
| Li 2014 | 1 | 1 | 1 | 1 | 0 | 0 | 1 | 5 | High |
| Honneger Bloch 2014 | 1 | 0 | 0 | 1 | 0 | 1 | 1 | 4 | High |
| Oh 2014 | 0 | 1 | 1 | 1 | 0 | 0 | 0 | 3 | Low |
| Arsov 2013 | 0 | 0 | 0 | 1 | 0 | 1 | 1 | 3 | Low |
| Lim 2013 | 1 | 0 | 0 | 1 | 0 | 1 | 1 | 4 | High |
| Li 2013 | 1 | 0 | 0 | 1 | 0 | 1 | 1 | 4 | High |
| Murthy 2012 | 1 | 0 | 0 | 1 | 1 | 0 | 0 | 3 | Low |
| An 2012 | 1 | 1 | 1 | 1 | 0 | 1 | 1 | 6 | High |
| Wu 2012 | 0 | 0 | 0 | 1 | 0 | 1 | 0 | 2 | Low |
| Lee 2012 | 1 | 1 | 1 | 1 | 0 | 1 | 1 | 6 | High |
| Ogawa 2010 | 0 | 0 | 0 | 1 | 0 | 1 | 1 | 3 | Low |
| Yayar 2018^27^ | 1 | 0 | 0 | 1 | 1 | 1 | 1 | 5 | High |
| Kawagoe 2018^28^ | 1 | 0 | 0 | 1 | 1 | 1 | 1 | 5 | High |
| Kon 2018^49^ | 0 | 0 | 0 | 1 | 1 | 1 | 0 | 3 | Low |
| Navaneethan 2018^19^ | 1 | 0 | 0 | 1 | 1 | 1 | 0 | 4 | High |
| Antunovic 2017^18^ | 1 | 0 | 0 | 1 | 1 | 0 | 0 | 3 | Low |
| Ulusoy 2015^31^ | 1 | 0 | 0 | 1 | 1 | 1 | 0 | 4 | High |
| Avramovski 2014^23^ | 0 | 0 | 0 | 1 | 1 | 1 | 0 | 3 | Low |
| Genovesi 2013^24^ | 0 | 0 | 0 | 1 | 1 | 1 | 0 | 3 | Low |
| den Hoedt 2013^38^ | 0 | 0 | 0 | 1 | 1 | 1 | 1 | 4 | High |
| Kakiya 2012^40^ | 0 | 0 | 0 | 1 | 1 | 1 | 1 | 4 | High |

Note: NOS = Newcastle-Ottawa Scale, 1 = the study satisfied the criteria, 0 = the study did not satisfy the criteria or it was unclear whether the study satisfied the crietria.
